# Structural Modification and Development of *N*‑(1,2,3,4-Tetrahydro-3-isoquinolinylmethyl)benzamide, BPR1M492, as a Potent and Rapid-Onset Opioid Analgesic with Reduced Withdrawal Symptoms

**DOI:** 10.1021/acs.jmedchem.6c00215

**Published:** 2026-06-01

**Authors:** Po-Wei Chang, Yung-Chiao Chang, Ya-Wen Tien, Hsiao-Fu Chang, Sheng-Ren Chen, Ya-Ping Chen, Wei-Cheng Huang, Chih-Chien Hsieh, Ling Chang, Hong Zhuang, Teng-Kuang Yeh, Chun-Wei Tung, Shiu-Hwa Yeh, Shau-Hua Ueng

**Affiliations:** † Institute of Biotechnology and Pharmaceutical Research, 427103 National Health Research Institutes, Miaoli County 35053, Taiwan, R.O.C.; ‡ School of Pharmacy, College of Medicine, National Cheng Kung University, Tainan 701, Taiwan, R.O.C; § The Ph.D. Program in Medical Neuroscience, College of Medical Science and Technology, Taipei Medical University and National Health Research Institutes, Taipei 110, Taiwan, ROC

## Abstract

A series of *N*-(1,2,3,4-tetrahydro-3-isoquinolinylmethyl)­benzamides, which are potent μ-opioid receptor (MOR) agonists, has been discovered. The most promising compound, compound **56** (BPR1M492), is an MOR agonist without a clear signaling bias between cAMP and β-arrestin-2 pathways, a cAMP-biased nociceptin-orphanin FQ opioid peptide agonist, and a weak cAMP-biased δ/κ-opioid receptor agonist. Compound **56** demonstrated potent *in vivo* antinociception at 0.027 mg/kg, offering rapid pain relief within 5 min of subcutaneous injection. It produced markedly milder withdrawal symptoms than TRV130 in mice, whereas differences in respiratory, gastrointestinal, reward-related, and tolerance-related measures were less pronounced and should be interpreted cautiously in light of the substantially lower dose required for antinociception. Compound **56** is a highly stable, slightly hygroscopic, low-moisture-containing crystalline solid that is safe at its *in vivo* effective dose.

## Graphical abstract

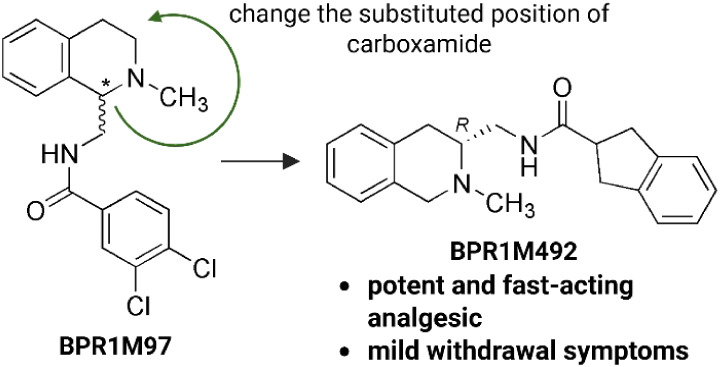

## Introduction

Opioids are an ancient and widely used class of pain relief medicines. The opioid analgesic market size is estimated to grow at a compound annual rate of 1.4% from 2023 to 2030, and the global market in 2022 was valued at 22.8 billion.^1^ However, unfavorable side effects of opioids, such as constipation, respiratory suppression, heart rate suppression, tolerance, withdrawal symptoms, and addiction,^2–5^ limit their use in pain treatment. Currently, one strategy that uses multiopioid receptor modulators is employed to resolve these unfavorable side effects.^6–20^ In addition, nonmorphine-like ligands^21–23^ may activate the mu-opioid receptor (MOR) through a different mechanism of action from that of morphine, which may alleviate the side effects caused by MOR activation, also known as agonist-selective signaling.^24^ Moreover, activation of MOR by biased ligands via the cAMP signaling pathway^25–32^ may ameliorate the side effects caused by MOR activation. A recently approved drug, Olinvyk (also known as TRV130), is a cAMP-biased MOR agonist used for postoperative pain control.^30–32^

In our previous study, we discovered a series of *N*-(1,2,3,4-tetrahydro-3-isoquinolinylmethyl)­benzamides that activated the MOR and exhibited potent antinociceptive effects in mouse models.^19,23^ The most effective compound among them, 1M97, shown in Figure 1, is a potent MOR and nociceptin-orphanin FQ opioid peptide (NOP) receptor dual agonist that exhibits an antinociceptive effect comparable to that of morphine, with less constipation. 1M97 is a racemic mixture and a noncrystalline solid. In this study, we aimed to discover a nonmorphine-like ligand with potent MOR activation ability that is more synthetically feasible for preparing enantiomeric isomers. We changed the substituted position of the carboxamide moiety from the 1- to the 3-position, and enantiomeric isomers were prepared using optically pure amino acids without chiral resolution or asymmetric synthesis (Figure 1). A structure–activity relationship study was conducted using racemic mixtures of *N*-(1,2,3,4-tetrahydro-3-isoquinolinylmethyl)­benzamide analogs. We then synthesized and evaluated the *in vitro* activity of enantiomeric isomers of *N*-(1,2,3,4-tetrahydro-3-isoquinolinylmethyl)­benzamide racemates with potent MOR agonistic activity. Among these enantiomeric isomers, *R*-configuration isomers usually exhibited better MOR agonism than *S*-configuration isomers. Compound **56**, the most potent *R*-configuration isomer of compound **53**, is a crystalline solid with good aqueous solution stability and solid-state stability.

**Figure 1:**
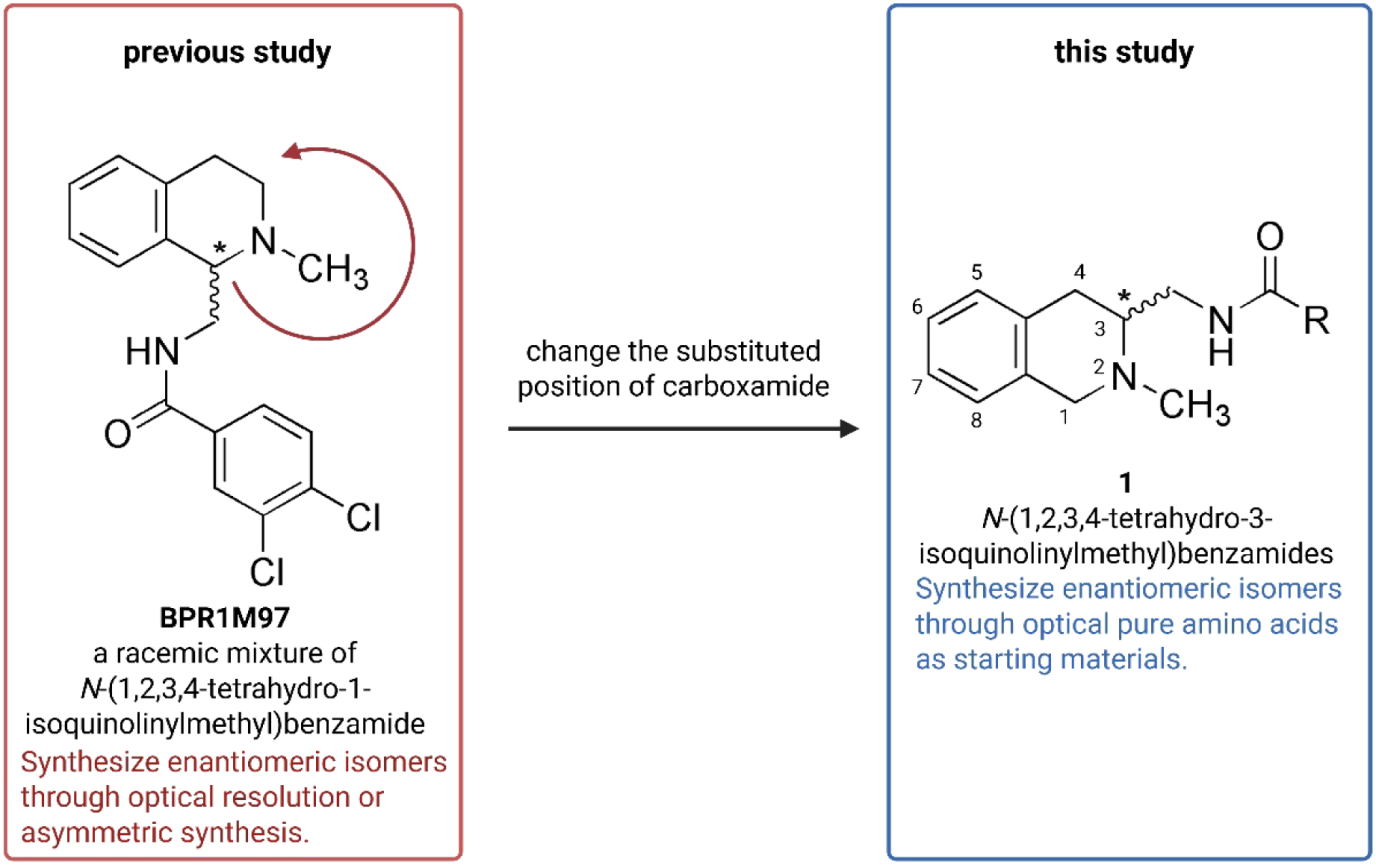
*N*-(1,2,3,4-tetrahydro-3-isoquinolinylmethyl)­benzamides were designed by changing the substituted position of the carboxamide moiety of *N*-(1,2,3,4-tetrahydro-1-isoquinolinylmethyl)­benzamides (1M97 series).

The molecular mechanism and animal pharmacology profiles of compound **56** were evaluated. The animal pharmacology study of compound **56**, including the potency of antinociception induced by this compound in the tail-flick test, antinociception in a disease-related pain model, and various side effects such as constipation, tolerance, reward, and withdrawal, was evaluated after treatment with compound **56**.

## Chemistry

The most important *N*-(1,2,3,4-tetrahydro-3-isoquinolinylmethyl)­benzamide intermediates were the substituted (2-methyl-1,2,3,4-tetrahydroisoquinolin-3-yl)­methanamines. The synthesis of the substituted (2-methyl-1,2,3,4-tetrahydroisoquinolin-3-yl)­methanamines and the final products is depicted in Schemes 1–3.^33–35^ As shown in Schemes 1 and 3, (2-methyl-1,2,3,4-tetrahydroisoquinolin-3-yl)­methanamines **2c** and **10c** were synthesized by employing 1,2,3,4-tetrahydroisoquinoline-3-carboxamides (compounds **2a** and **10a**), and methylation was performed at the N^2^-position via a reductive amination reaction, followed by the lithium aluminum hydride (LAH)-mediated reduction of amides **2b** and **10b**. In Schemes 1 and 3, the preparation of substituted (2-methyl-1,2,3,4-tetrahydroisoquinolin-3-yl)­methanamines **3**–**6i**, **8**–**9i**, and **12**–**15i** was carried out using various amino acids **3**–**6a**, **8**–**9a**, and **12**–**15a**, respectively. The carboxylic acid groups of these amino acids were converted to methyl esters **3**–**6b**, **8**–**9b**, and **12**–**15b** by thionyl chloride-mediated methyl esterification. The amine groups of these compounds were then protected with either ethyl or methyl carbamates using ethyl chloroformate or methyl chloroformate. The formation of tetrahydroisoquinoline was performed using carbamates, paraformaldehyde, sulfuric acid, and acetic acid, followed by the hydrolysis of ester and carbamate groups with 6–12 N hydrochloric acid_(aq)_. The carboxylic acid groups of compounds **3**–**6e**, **8**–**9e**, and **12**–**15e** were transformed to methyl esters by sulfuric acid-mediated esterification or thionyl chloride-mediated esterification. Then, amide formation was performed with aqueous 28–30% ammonia solution to give compounds **3**–**6g**, **8**–**9g**, and **12**–**15g**. Methylation at N^2^-position and amide reduction to produce key substituted (2-methyl-1,2,3,4-tetrahydroisoquinolin-3-yl)­methanamines **3**–**6i**, **8**–**9i**, and **12**–**15i** were performed following the same reductive amination and amide reduction conditions used to prepare compounds **2c** and **10c**. The preparation of tetrahydroisoquinoline **7b** in Scheme 2 was carried out with 2-amino-3-(4-methylphenyl)­propanoic acid **7a**, formaldehyde, and hydrobromic acid in water. (2,7-Dimethyl-1,2,3,4-tetrahydroisoquinolin-3-yl)­methanamine **7f** was synthesized by the same route as described above for the preparation of **3**–**6i**, **8**–**9i**, and **12**–**15i**. In Scheme 3, [(3*R*)-2-methyl-1,2,3,4-tetrahydroisoquinolin-3-yl]­methanamine **11e** was synthesized from commercially available (3*R*)-1,2,3,4-tetrahydroisoquinoline-3-carboxylic acid hydrochloride (1:1) (**11a**) via the same synthetic route, including methyl esterification, amide formation, reductive amination, and amide reduction, as mentioned above. In the cyclization of 6-substituted tetrahydroisoquinoline cores, a mixture of the desired 6-substituted isomer and the undesired 8-substituted isomer was obtained from the paraformaldehyde-, sulfuric acid-, and acetic acid-mediated cyclization reaction. This mixture of 6- and 8-substituted isomers could be completely separated during the purification of the crude product in subsequent steps by flash column chromatography (details are described in the Supporting Information). Final products **16**–**62** in each scheme were synthesized either via an amide coupling reagent-mediated reaction with various carboxylic acids or through an acyl chloride-mediated amide coupling reaction.

**Scheme 1:**
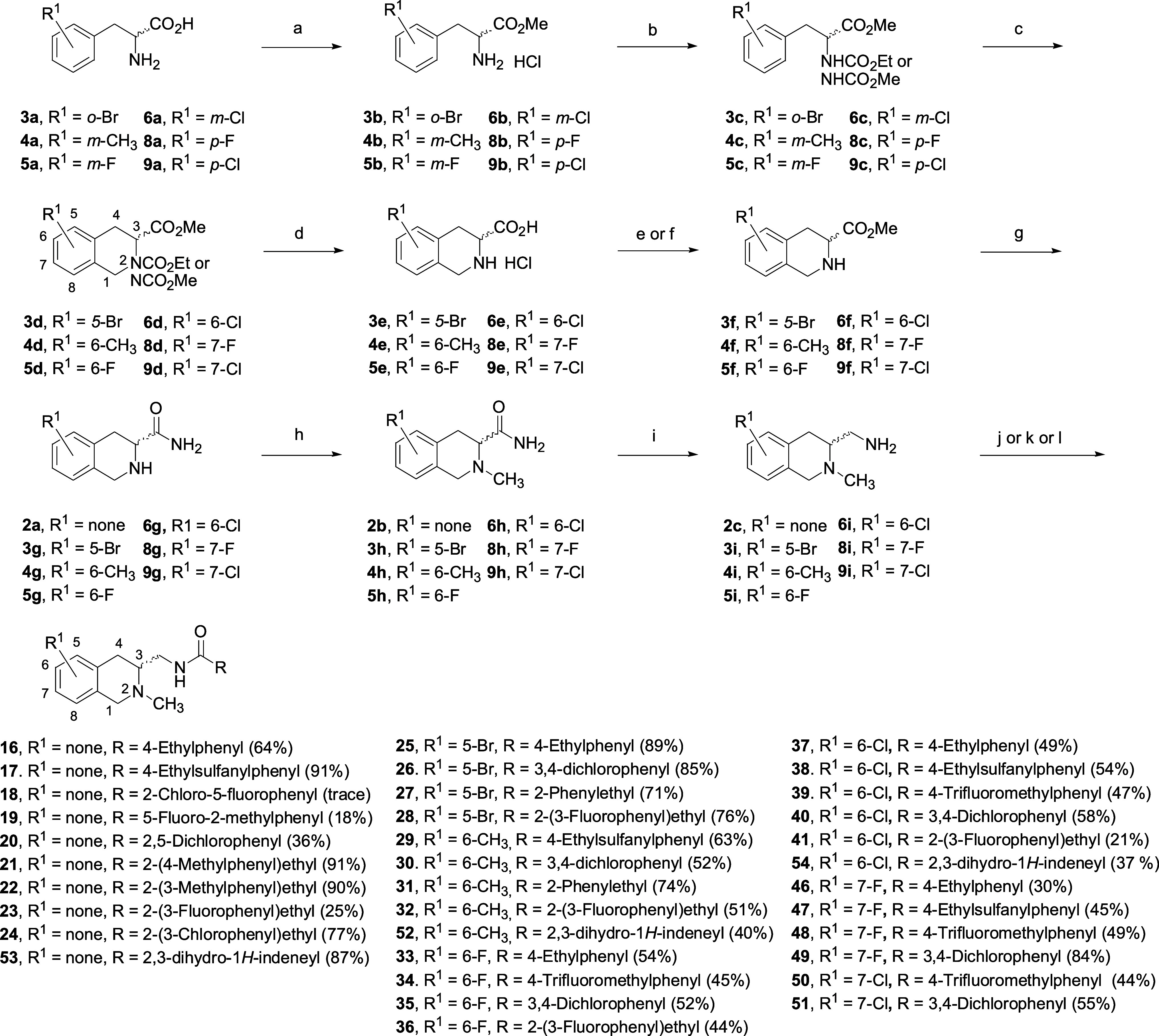
Synthesis of *N*-(1,2,3,4-tetrahydro-3-isoquinolinylmethyl)­benzamides **16**–**41** and **46**–**54** ^a^

a Reagents and conditions: (a) SOCl_2_, MeOH, 60 °C to reflux, 16–18 h, 78% to quantitative yield; (b) ethyl chloroformate, pyridine, CH_2_Cl_2_, 0 °C to rt, 1–2 h, 73% to quantitative yield; (c) paraformaldehyde, H_2_SO_4_, CH_3_COOH , rt, 16–18 h, 44% to quantitative yield; (d) aqueous 6 N–12 N HCl, reflux, 17–48 h, 36–98%; (e) SOCl_2_, NEt_3_, anhydrous MeOH, 55 °C to reflux, 16–18 h, 79–98%; (f) H_2_SO_4_, MeOH, reflux, 16−18 h, 88−90% (g) aqueous 28–30% NH_4_OH, rt, 16–18 h, 69–98%; (h) 1. CH_2_O, CH_3_CN, THF, rt, 1 h. 2. NaBH­(OAc)_3_, CH_3_COOH, rt, 2–16 h, 27–98%, 2 steps; (i) LiAlH_4_, THF, 60 °C to reflux, 4–48 h, 36–98%; (j) R–COOH, EDCI-HCl, HOBt, DIPEA, CH_2_Cl_2_, rt, 16–18 h, 21–89%; (k) R–COCl, NEt_3_, CH_2_Cl_2_, 0 °C to rt, 1 h, 45–85%; (l) R–COOH, NEt_3_, T_3_P (50 wt % in ethyl acetate), CH_2_Cl_2_, rt, 16 h, 55%. (**4d**–**4i**, **5d**, **6d**, and **6e** contained inseparable mixtures of 6- and 8-substituted isomers, percent yields were determined by ^1^H NMR spectra).

**Scheme 2:**
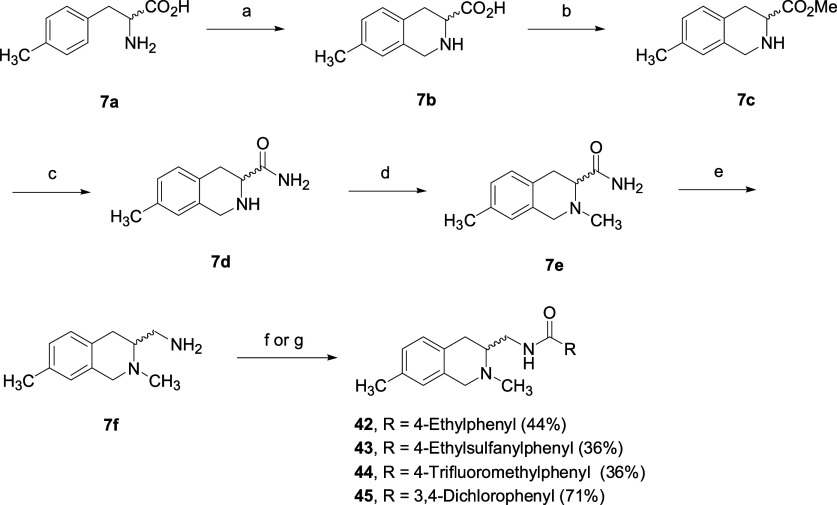
Synthesis of the *N*-(1,2,3,4-tetrahydro-3-isoquinolinylmethyl)­benzamides **42**–**45** ^a^

a Reagents and conditions: (a) CH_2_O, 48% HBr, H_2_O, rt to 75 °C, 5 h, 99%; (b) SOCl_2_, NEt_3_, anhydrous MeOH, 50 °C, 17 h, 94%; (c) 28–30% NH_4_OH, rt, 17 h, 57%; (d) 1. CH_2_O, CH_3_CN, THF, rt, 1 h. 2. NaBH­(OAc)_3_, CH_3_COOH, rt, 17 h, 79%, 2 steps; (e) LiAlH_4_, THF, 50 °C, 17 h, 72%; (f) R_2_-COOH, EDCI·HCl, HOBt, DIPEA, CH_2_Cl_2_, rt, 17 h, 36–71%; (g) R–COCl, NEt_3_, CH_2_Cl_2_, 0 °C to rt, 1 h, 36%.

**Scheme 3:**
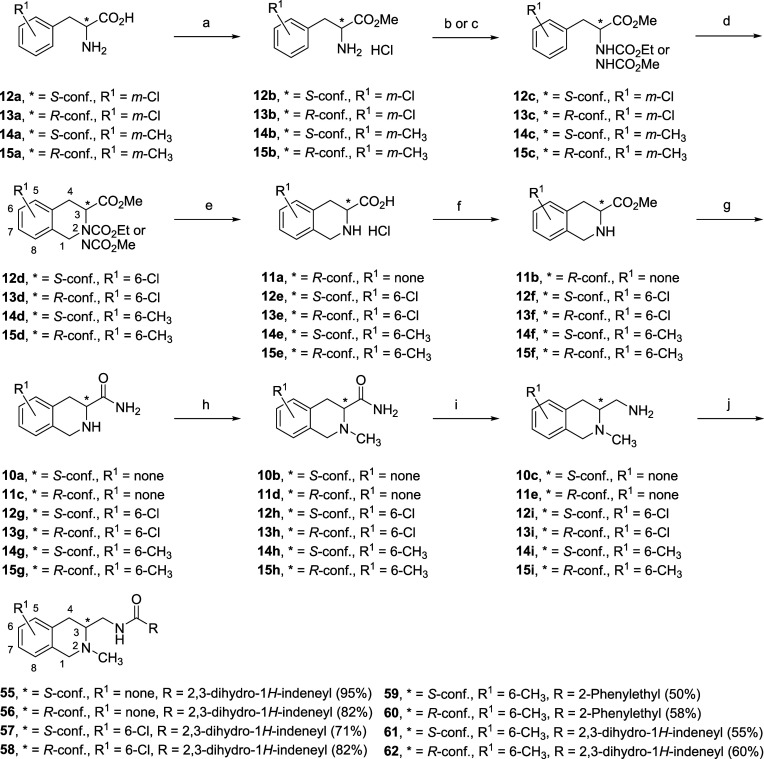
Synthesis of *N*-(1,2,3,4-tetrahydro-3-isoquinolinylmethyl)­benzamides **55**–**62** ^a^

a Reagents and conditions: (a) SOCl_2_, MeOH, 50 °C to reflux, 16–18 h, 34% to quantitative yield; (b) ethyl chloroformate, pyridine, CH_2_Cl_2_, 0 °C, 1–2 h, 99% to quantitative yield; (c) methyl chloroformate, NaHCO_3_, CH_2_Cl_2_, rt, 16 h, 91–97%; (d) paraformaldehyde, H_2_SO_4_, CH_3_COOH , rt, 16–18 h, 43–88% (an unseparated mixture of 6- and 8-substituted isomers); (e) aqueous 6–8 N HCl, reflux, 17–48 h, 37–72%; (f) SOCl_2_, NEt_3_, anhydrous MeOH, 50 °C to reflux, 16–18 h, 36–99%; (g) aqueous 28–30% NH_4_OH, rt, 16–18 h, 60–92%; (h) 1. CH_2_O, CH_3_CN, THF, rt, 1 h. 2. NaBH­(OAc)_3_, CH_3_COOH, rt, 2–16 h, 82% to quantitative yield, 2 steps; (i) LiAlH_4_, THF, 60 °C to reflux, 17–48 h, 32–84%; (j) R–COOH, EDCI-HCl, HOBt, DIPEA, CH_2_Cl_2_, rt, 16–18 h, 50–95%. (**14d**–**14f** and **15d** contained inseparable mixtures of 6- and 8-substituted isomers; percent yields were determined by ^1^H NMR spectra).

## Results and Discussion

### Structure–Activity Relationship (SAR) Study of *N*-(1,2,3,4-Tetrahydro-3-isoquinolinylmethyl)­benzamides

The FLIPR Ca^2+^ assay was performed in CHO-K1 cells expressing human MOR and Gα15 (CHO-K1/hMOR/Gα15) and measured their real-time responsive fluorescence (RFU) caused by the intracellular calcium release during MOR activation, indicating MOR activation via a G_αi_ protein-coupled mechanism.^21^ The real-time RFU variation curve was integrated over time and reported as the area under the curve (AUC) at different concentrations of the test compound. The maximum AUC of the test compound, divided by the maximum AUC of BPR1M97, was reported as % *E*_max_. The reference compound BPR1M97 acted as a full MOR agonist, displaying potent activity (EC_50_ = 0.16 nM, % *E*_max_ = 100).

As shown in Table 1, all analogs were racemic mixtures, and the *para*-ethyl-substituted benzene of the benzamide moiety (compound **16**) showed 100-fold less potent MOR agonism than BPR1M97. The introduction of ethylsulfanylbenzene into the benzamide moiety did not substantially improve activity, and compound **17** exhibited a 31-fold less potent EC_50_ value than that of BPR1M97. Disubstituted benzene at *ortho*- and *meta*-positions of the benzamide moiety resulted in much poorer activity than BPR1M97; compounds **18**–**20** showed EC_50_ values 199- to 281-fold higher than that of BPR1M97. The maximum efficacy of MOR activation produced by compounds **16**–**20** was approximately 72.0 to 90.0%. Extension of the benzamide linker to phenylethyl benzamides and analogs, including *para*-methyl phenylethyl (**21**), *meta*-methyl phenylethyl (**22**), *meta*-fluoro phenylethyl (**23**), and *meta*-chloro phenylethyl (**24**), led to lower EC_50_ values than BPR1M97 (52- to 5.9-fold). The maximum efficacy of MOR activation produced by compounds **21**–**24** was 88.5 to 114%, revealing that the ethylene linker of the benzamide moiety could benefit MOR agonism. Regarding the substituent position on the benzene ring of phenylethyl benzamide, the *meta*-substituent was more potent than the *para*-substituent. The introduction of a 5-bromo substituent on the benzene ring of tetrahydroisoquinoline decreased MOR agonism. Compounds **25** and **26,** possessed *para*-ethyl and 3,4-dichloro-substituted benzene of benzamide moieties, respectively, exhibited 1363- and 1794-fold less potent EC_50_ values than BPR1M97, respectively. Phenylethyl (**27**) and *meta*-fluoro phenylethyl (**28**) benzamide analogs displayed 42.6- and 68.8-fold higher EC_50_ values than BPR1M97, respectively. These four analogs (**25**–**28**) exhibited 45.8–92.5% of maximum efficacies in MOR activation. The 6-methyl tetrahydroisoquinoline core with *para*-thioethyl and 3,4-dichloro-substituted benzamide analogs, compounds **29** and **30**, exhibited 675- and 173-fold less potent EC_50_ than BPR1M97, respectively. Notably, both analogs displayed maximum efficacies of 108–124%, respectively, in MOR activation, exceeding that of BPR1M97. Remarkably, the 6-methyl tetrahydroisoquinoline core with phenylethyl benzamide (**31**) exhibited 1.45-fold more potent EC_50_ than BPR1M97 and 80.6% maximum MOR activation efficacy; however, the *meta*-fluoro phenylethyl benzamide (**32**) exhibited 31-fold weaker EC_50_ than BPR1M97 with 84.4% maximum MOR activation efficacy. Replacement of 6-methyl tetrahydroisoquinoline with 6-fluoro tetrahydroisoquinoline did not improve the ability of the compounds to exhibit MOR agonism. The *para*-ethyl, *para*-trifluoromethyl, 3,4-dichloro, and *meta*-fluoro phenylethyl benzamide analogs **33**–**36** showed 126- to 769-fold less potent EC_50_ values than that of BPR1M97, with 86.1–102% of maximum efficacy in MOR activation. Furthermore, the 6-chloro tetrahydroisoquinoline core with *para*-ethyl, *para*-sulfanylethyl, *para*-trifluoromethyl, 3,4-dichloro, and *meta*-fluoro phenylethyl benzamide analogs **37**–**41** displayed 9.1- to 288-fold weaker EC_50_ values than that of BPR1M97, and 96.3 to 111% of maximum efficacy in MOR activation. The results for the 6-methyl-substituted tetrahydroisoquinoline and phenylethyl benzamide analogs implied that the 6-methyl substitutents at the tetrahydroisoquinoline core and phenylethyl benzamides might improve the efficacy of MOR activation. Shifting the substituent from C6 to C7 in the tetrahydroisoquinoline core gave 7-methyl, 7-fluoro, and 7-chloro tetrahydroisoquinolines with *para*-ethyl, *para*-sulfanylethyl, *para*-trifluoromethyl, and 3,4-dichloro benzamide analogs **42**–**51**. None of these analogs showed improved MOR agonistic activity and exhibited 85.6- to 2619-fold weaker EC_50_ values than that of BPR1M97. Most of the *N*-(7-substituted tetrahydroisoquinolinemethyl) benzamides showed strong efficacy in terms of MOR activation, with 81.9 to 102% of maximum efficacy, except for compound **43** (73.0%).

**Table 1:**
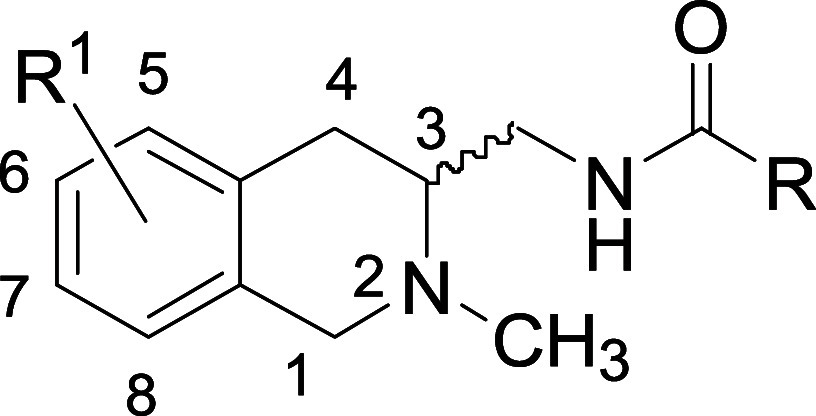

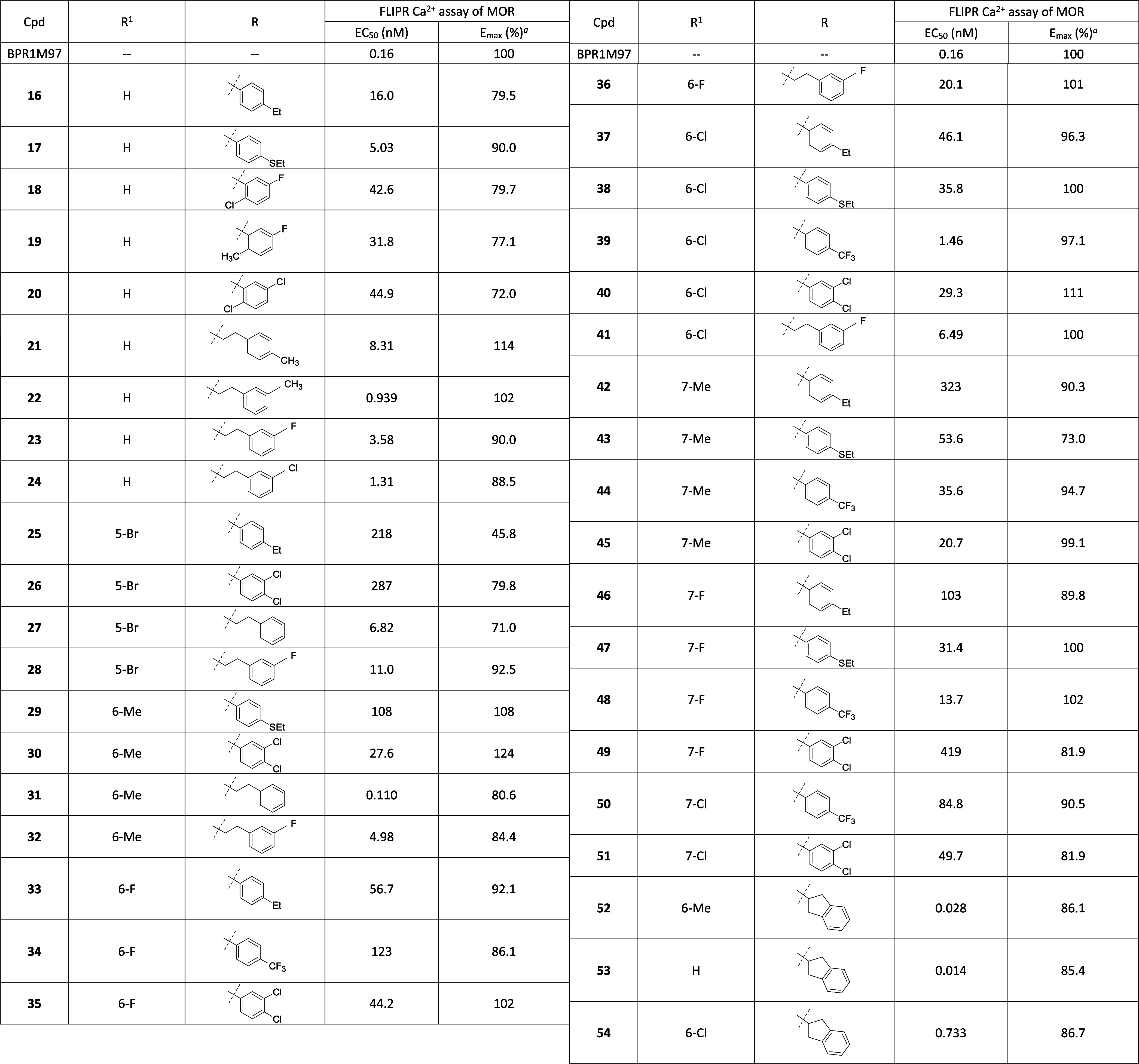
Results of FLIPR Ca^2+^ Assay of *N*-(1,2,3,4-Tetrahydro-3-isoquinolinylmethyl)­benzamides **16**–**54** in MOR

a % of BPR1M97 *E*_max_.

The results for compounds **16**–**51** revealed a general trend that the nonsubstituted, 6-chloro, and 6-methyl tetrahydroisoquinolines with a phenylethyl benzamide moiety showed more potent activity than the rest. We decided to fix the ethylene linker of phenylethyl benzamide to indanyl carboxamide to increase the rigidity of the amide moiety. Compounds **52**–**54**, which possess a 6-methyl, nonsubstituted, and 6-chloro tetrahydroisoquinoline core, respectively, showed 5.7- to 11.4-fold stronger EC_50_ values than that of BPR1M97, with 85.4–86.1% maximum efficacies, except for compound **54** (4.6-fold less potent than BPR1M97 with 86.7% maximum efficacy). These results revealed that a rigid amide substituent could improve MOR agonistic activity, which inspired us to prepare two enantiomeric isomers of each of compounds **31** and **52**–**54** to understand the influence of the configuration at C3 of these compounds on MOR agonism.

Both the *R*- and *S*-configured C3 enantiomeric isomers of compounds **31** and **52**–**54** were evaluated for their MOR activation abilities using a FLIPR Ca^2+^ assay. As shown in Table 2, all *R*-configuration C3 isomers exhibited better MOR agonistic activity than the *S*-configuration C3 isomers, with both potency and maximum efficacy (*R*-configuration isomers were 3.4- to 59.8-fold more potent than *S*-configuration isomers). These results suggest that the *R*-configuration isomers of the *N*-(1,2,3,4-tetrahydro-3-isoquinolinyl)­methyl core are preferred for MOR activation. Analogs with a rigid indanyl carboxamide showed superior activity to the linear phenylethyl benzamide, and compounds **61** and **62** were 40.8- and 8.5-fold more potent than **59** and **60**, respectively. Among these eight analogs, compound **56** was the most potent and efficacious compound, showing a 40-fold more potent EC_50_ than BPR1M97, with 91.7% maximum efficacy. Because of their superior potency and efficacy in MOR activation compared with BPR1M97, compounds **56**, **60,** and **62** were selected for further evaluation in animal pharmacology experiments.

**Table 2:**
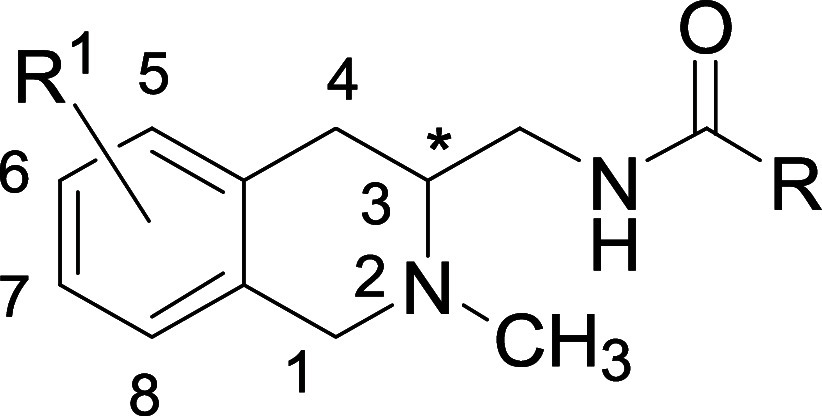

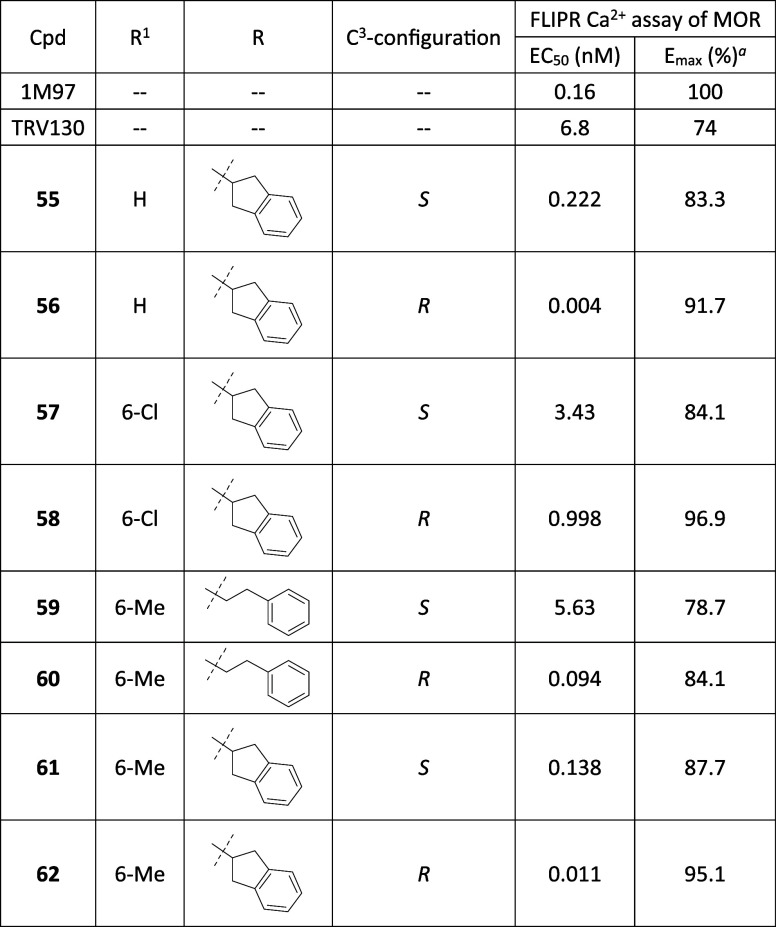
Results of FLIPR Ca^2+^ Assay of Enantiomeric *N*-(1,2,3,4-Tetrahydro-3-Isoquinolinylmethyl)­benzamides **55**–**62** in MOR

a = % of BPR1M97 *E*_max_.

### Antinociceptive Effect Produced by Compounds **56**, **60**, and **62**

The antinociceptive ED_50_ values of compounds **56**, **60**, and **62,** when administered subcutaneously, were measured using the up–down method^36^ in a thermally stimulated mouse tail-flick test with WT (for **60** and **62**) and hMOR^+^; mMOR^–/–^ (for **56**) mice.^37^ The dose-response curves of these three compounds are shown in Figure S1, and the ED_50_ values of **56**, **60**, and **62** were measured using the up–down method, showing 0.027 ± 0.002, 0.172 ± 0.036, and 0.97 ± 0.069 mg/kg, respectively. Among these three compounds, **56** exhibited the most potent antinociceptive activity mediated by MOR activation, as this antinociceptive effect was abolished in the mMOR^–/–^ mouse model (Figure S2A). In addition, the synthetic route for synthesizing **56** requires fewer steps than those for compounds **60** or **62**. The onset time of antinociception of **56** was approximately 5 min after subcutaneous (s.c.) administration (Figure S2B), which indicated that compound **56** shows potential as an analgesic for postoperative patients. Olinvyk (TRV130), developed by Trevena Inc. and approved in 2020, is used to treat moderate to severe acute pain in postoperative patients.^30–32^ TRV130 was used as a positive control in the animal pharmacology studies described below.

### Antinociceptive Activity of Compound **56** in Mouse Pain Models and its Effects on Oxygen Saturation

Figure 2 presents the evaluation of the antinociceptive efficacy and side effects of the novel analgesic compound **56** and the known MOR agonist TRV130 in mouse models of thermally induced pain and postoperative pain. The results show that both compounds increased pain thresholds in a dose-dependent manner in response to thermal (Figure 2A) and mechanical stimuli (Figure 2B,C), indicating significant antinociceptive effects. At low and medium doses, pulse oximetry measurements (Figure 2D) showed that compound **56** groups exhibited a smaller decrease in oxygen saturation than TRV130 at 20 and 30 min (Figure 2E). In addition, all compound **56** groups demonstrated a smaller reduction in respiratory rate compared with TRV130 at all time points (Figure 2F). In contrast, the heart rate showed a compensatory increase (Figure 2G). Further experiments demonstrated that these adverse effects were abolished in MOR knockout (mMOR^–/–^) mice, suggesting that the side effects are indeed mediated through the MOR (Figure S3A–S3C). Overall, compound **56** produced potent antinociception and showed smaller reductions in oxygen saturation and respiratory rate than TRV130 under the present dosing conditions. However, because compound **56** was substantially more potent than TRV130 in the antinociceptive assay, these differences should be interpreted cautiously and may partly reflect the lower doses required to achieve antinociceptive effects.

**Figure 2:**
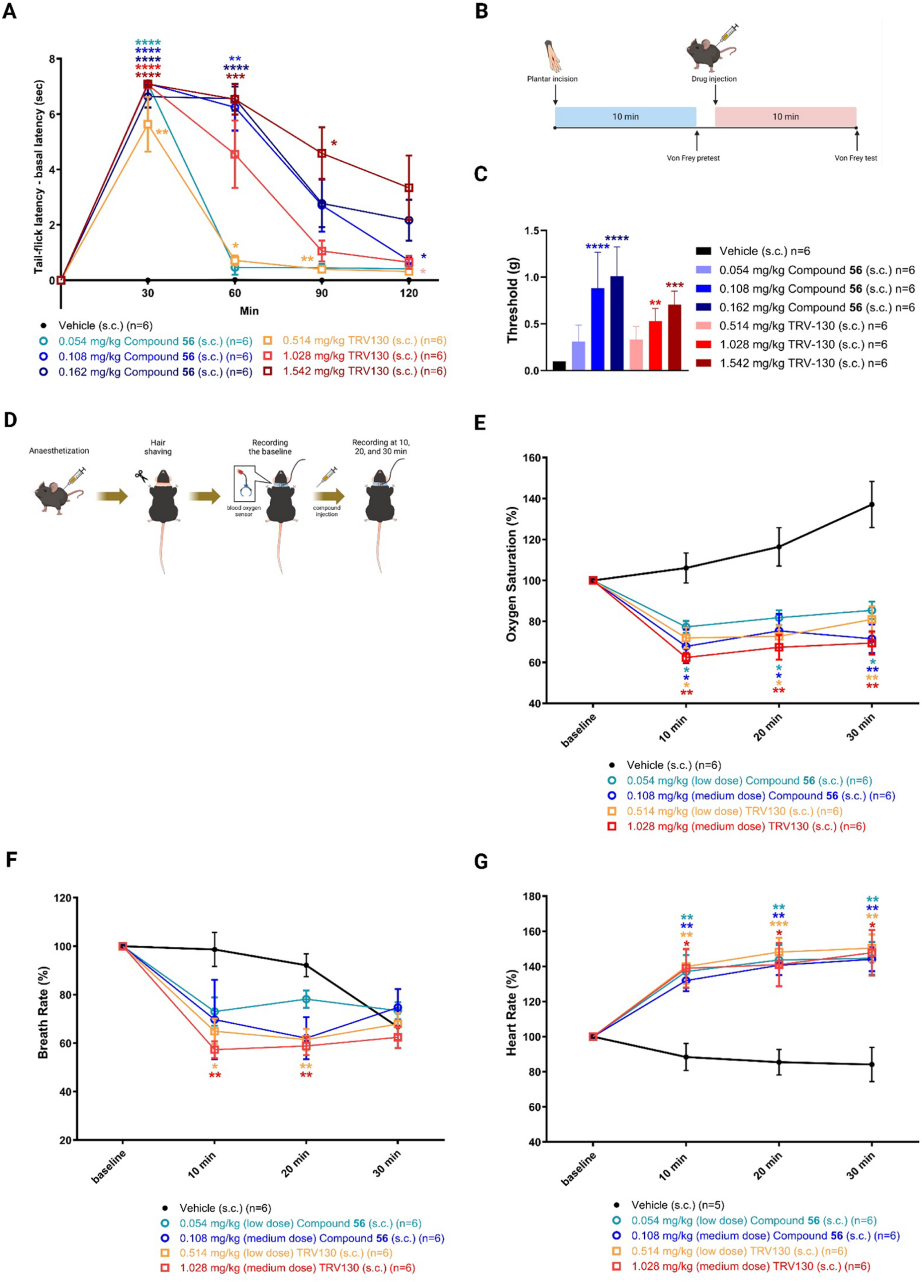
Acute thermal and postoperative antinociceptive effects, and the cardiopulmonary impact of compound **56** and TRV130. (A) Time-course analysis of acute thermal antinociceptive effects of compound **56** and TRV130 in wild-type mice using the tail-flick test. *p < 0.05, **p < 0.01, ***p < 0.001, ****p < 0.0001 vs vehicle group. (B) An experimental flowchart illustrating the protocol for assessing postoperative incision pain. (C) Evaluation of mechanical allodynia using von Frey hair filaments following each treatment. **p < 0.01, ***p < 0.001, ****p < 0.0001 vs vehicle. (D) Experimental timeline for investigating the effects of each treatment on cardiopulmonary function. (E) Effects on oxygen saturation levels assessed using a pulse oximeter. (F, G) Respiratory rate (F) and heart rate (G) were measured at specified time points to assess the respiratory and circulatory effects of compound **56** and TRV130. Data in panels E–G are presented as percentages relative to baseline values. *p < 0.05, **p < 0.01, ***p < 0.001 vs vehicle. All values are expressed as mean ± SEM.

### Comparison of Antinociceptive Efficacy, Gastrointestinal Effects, Tolerance, and Reward Liability of Compound **56** and TRV130 in Mouse Models

We further investigated the antinociceptive efficacy, tolerance development, side effect profile, and addiction potential of the novel antinociceptive compound **56** compared to those of TRV130 in mouse models. Figure 3A and B shows the dose–response curves of both compounds in the tail-flick test, along with their effects on gastrointestinal transit. In the TRV130-treated group, gastrointestinal (GI) transit inhibition ranged from 42–69%, and the antinociceptive ED_50_ was 0.295 mg/kg (Figure 3B). In contrast, compound **56** exhibited a markedly lower antinociceptive ED_50_ of 0.027 mg/kg, while GI transit inhibition ranged from 28 to 67%, which was significantly lower than that observed in the TRV130-treated group at low to medium doses (Figure 3A). Compound **56** showed less gastrointestinal transit inhibition than TRV130 at low to medium doses under the present experimental conditions. However, because compound **56** achieved antinociception at a substantially lower ED_50_ than TRV130, this difference may partly reflect dose sparing associated with its higher antinociceptive potency. This gastrointestinal disruption was mediated by MOR activation, as it was abolished in mMOR^–/–^ mice treated with compound **56** (Figure S2C). Figure 3C and 3D compares the development of antinociceptive tolerance after repeated dosing, indicating that both compounds exhibit similar tolerance shifts, with post-treatment ED_50_ values being close to their original ED_50_. Figure 3E and F shows the results of the conditioned place preference (CPP) test. Compound **56** showed lower CPP responses than TRV130 at some of the tested dose levels (1× and 8× ED_50_). Notably, compound **56** elicited significant place preference only at medium to high doses (2× and 4× ED_50_); however, these findings do not by themselves establish an intrinsically lower reward liability, because the difference may also be influenced by the substantially lower doses required for compound **56** to produce antinociception. Figure 3G and H shows the evaluation of withdrawal behaviors, revealing that TRV130-treated mice exhibited pronounced withdrawal symptoms, including paw tremors, wet-dog shakes, tooth chattering, diarrhea, and ptosis, after cessation, whereas compound **56** elicited only mild withdrawal signs. Taken together, compound **56** retained effective antinociception and showed the clearest improvement in withdrawal-related behaviors relative to TRV130. In contrast, differences in gastrointestinal transit, cardiopulmonary parameters, tolerance, and CPP were less pronounced and should be interpreted cautiously, as they may partly reflect the substantially lower antinociceptive dose required for compound **56**.

**Figure 3:**
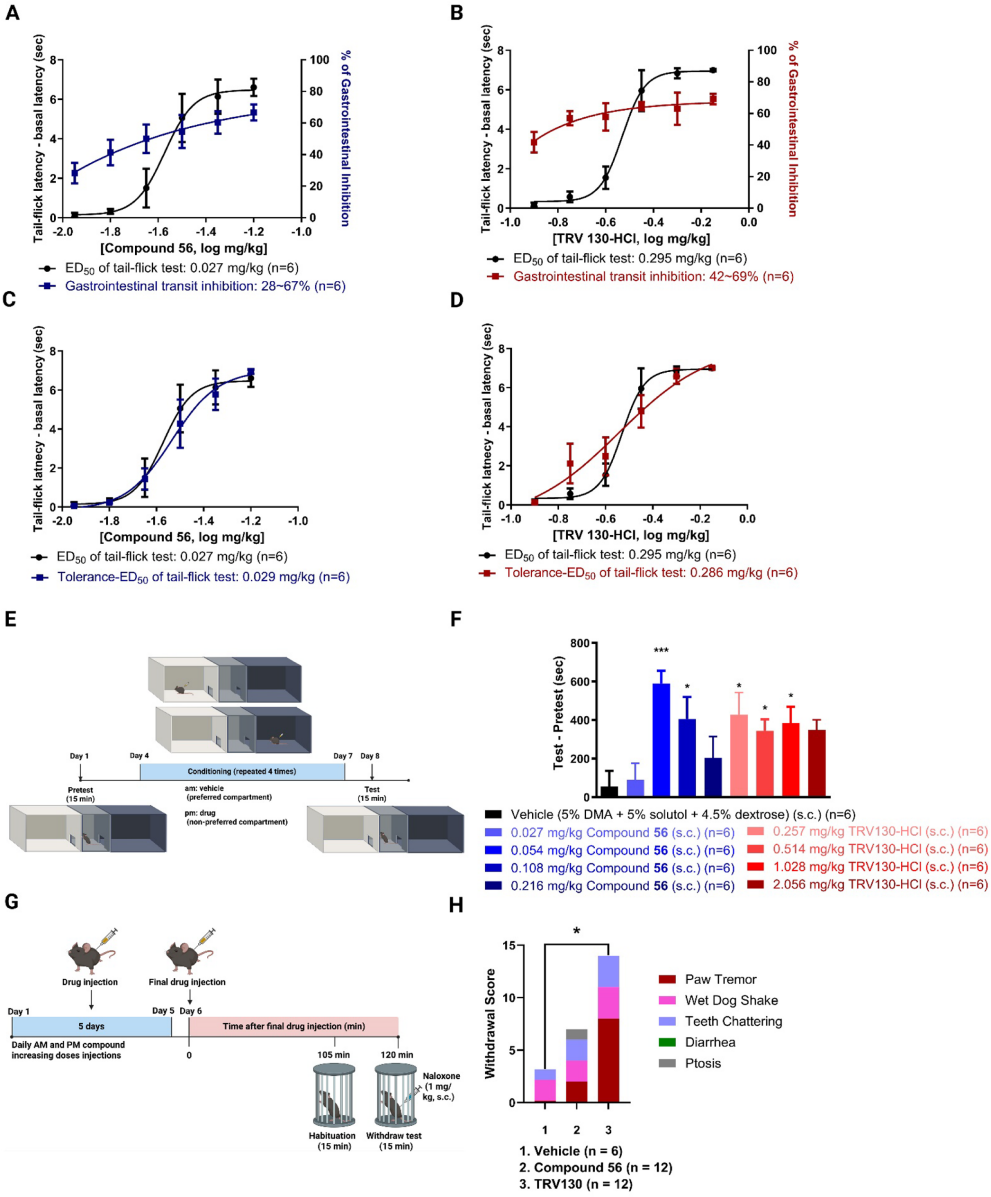
Assessment of antinociceptive potency, tolerance, gastrointestinal side effects, reward behavior, and withdrawal signs of compound **56** and TRV130 in mice. (A, B) Dose–response curves of tail-flick latency and gastrointestinal transit inhibition following the administration of compound **56** (A) or TRV130 (B). (C, D) Tail-flick dose–response curves before and after repeated dosing to evaluate antinociceptive tolerance for compound **56** (C) and TRV130 (D). (E) A schematic of the CPP protocol. (F) CPP test results show the time difference spent in the drug-paired compartment (test–pretest). *p < 0.05, ***p < 0.001 vs vehicle. (G) Timeline for withdrawal assessment following repeated escalating-dose administration. (H) Stacked bar plot summarizes withdrawal scores based on observed symptoms. *p < 0.05 vs vehicle group. Data are shown as mean ± SEM.

### Mechanism of Action between Compound **56** and Opioid Receptors

The cAMP assays of MOR, NOP, κ-opioid receptor (KOR), and the FLIPR Ca^2+^ assay of δ-opioid receptor (DOR) for compound **56** were performed by an outsourced research organization (Eurofins DiscoverX Co.), and the results are shown in Table 3. Compound **56** produced full agonism of the MOR and NOP. The EC_50_ value of **56** in MOR was comparable to that of DAMGO (a reference agonist of MOR), and that in NOP was 715-fold higher than that of orphanin FQ (a reference agonist of NOP). Compound **56** produced weak agonism in DOR and KOR, with EC_50_ values that were 663-fold and 2025-fold less potent than DPDPE (a reference agonist of DOR) and U-69593 (a reference agonist of KOR), respectively. The β-arrestin-2 signaling pathway would be activated for opioid receptor internalization.^38–40^ Thus, we utilized an assay for the secondary messenger, that is, a β-arrestin-2 recruitment assay, to investigate the action of compound **56** in opioid receptor internalization on MOR, NOP, DOR, and KOR. On MOR, compound **56** effectively recruited β-arrestin-2 with 1.8-fold less potency than Met-5 (a reference compound of MOR). Compound **56** exhibited nearly no effect on β-arrestin-2 recruitment for NOP and KOR, and a partial effect for DOR with an EC_50_ value that was 481-fold higher than that of DADLE (a reference compound of DOR). The ratio of EC_50_ values of compound **56** in the MOR and KOR cAMP assays was >2591, in the MOR and DOR β-arrestin-2 recruitment assays was 17.9, and in the MOR and DOR FLIPR Ca^2+^ assays was >2.5 × 10^5^. These results suggested that compound **56** is a potent MOR agonist, with both cAMP and β-arrestin-2 pathways activated without evidence of preferential signaling in our assay setting.

**Table 3:** Mechanisms by which Compound **56** is Involved in Opioid Receptor Signaling Pathways

cAMP or FLIPR Ca^2+^assay on opioid receptors							
MOR		NOP		DOR^a^		**KOR**	
EC_50_ (nM)	% of *E*_max_ ^b^	EC_50_ (nM)	% of *E*_max_ ^c^	EC_50_ (nM)	% of *E*_max_ ^d^	EC_50_ (nM)	% of *E*_max_ ^e^
0.93	100	52.89	95.79	730	104.1	2410	88

β-arrestin-2 recruitment on opioid receptors							
EC_50_ (nM)	% of *E*_max_ ^f^	EC_50_ (nM)	% of *E*_max_ ^g^	EC_50_ (nM)	% of *E*_max_ ^h^	EC_50_ (nM)	% of *E*_max_ ^i^
161	105.8	>1 × 10^5^	9	2883	59	>1 × 10^5^	3

a FLIPR Ca^2+^ Assay on DOR.

b = % of DAMGO *E*_max_, EC_50_ of DAMGO = 1.4 nM.

c = % of orphanin FQ *E*_max_, EC_50_ of orphanin FQ = 0.074 nM.

d = % of DPDPE *E*_max_, EC_50_ of DPDPE = 1.1 nM.

e = % of U-69593 *E*_max_, EC_50_ of U-69593 = 1.19 nM.

f = % of Met-5 *E*_max_, EC_50_ of Met-5 = 91 nM.

g = % of orphanin FQ *E*_max_, EC_50_ of orphanin FQ = 7 nM.

h = % of DADLE *E*_max_, EC_50_ of DADLE = 6 nM.

i = % of dynorphin A *E*_max_, EC_50_ of dynorphin A = 23 nM.

### Molecular Dynamics to Predict Interactions between Compound **56** and MOR

To explore the binding positions of chiral compounds **55** (*S*-configuration on C3) and **56** (*R*-configuration on C3) with MOR, we performed protein–ligand docking with MOR (PDB ID: 4DKL) using AutoDock Vina^41^ and followed by 1.5 μs molecular dynamics (MD) simulations using AMBER (Figure 4). The binding of **56** and **55** to MOR exhibited distinctly different preferences. Compound **56** predominantly interacted with Tyr150, Met153, and Lys235 in the crevice of transmembrane helix 3 (TM3) and TM5 (Figure 4A, B). Tyr150 and Lys235 formed hydrogen bonds with the oxygen of the amide moiety of **56**, and Met153 formed an aryl–sulfur interaction with the indanyl group of **56**. In contrast to compound **56**, compound **55** interacted with MOR by binding to Gln126, Trp135, and Tyr328, which is the crevice between TM2 and TM3 (Figure 4C, D). Gln126 and Tyr328 stabilized the nitrogen of the amide moiety of **55** through hydrogen-bonding interactions, whereas Trp135 formed an edge-to-face π-interaction with the benzene ring of the tetrahydroisoquinoline core of **55**. In the molecular dynamics simulated models, compound **55** remained near extracellular loop 1 (Trp135) toward the top of the binding pocket (Figure 4B), whereas compound **56** adopted a deeper binding position closer to the bottom of the crevice formed by TM3 and TM5 (Figure 4A).

**Figure 4:**
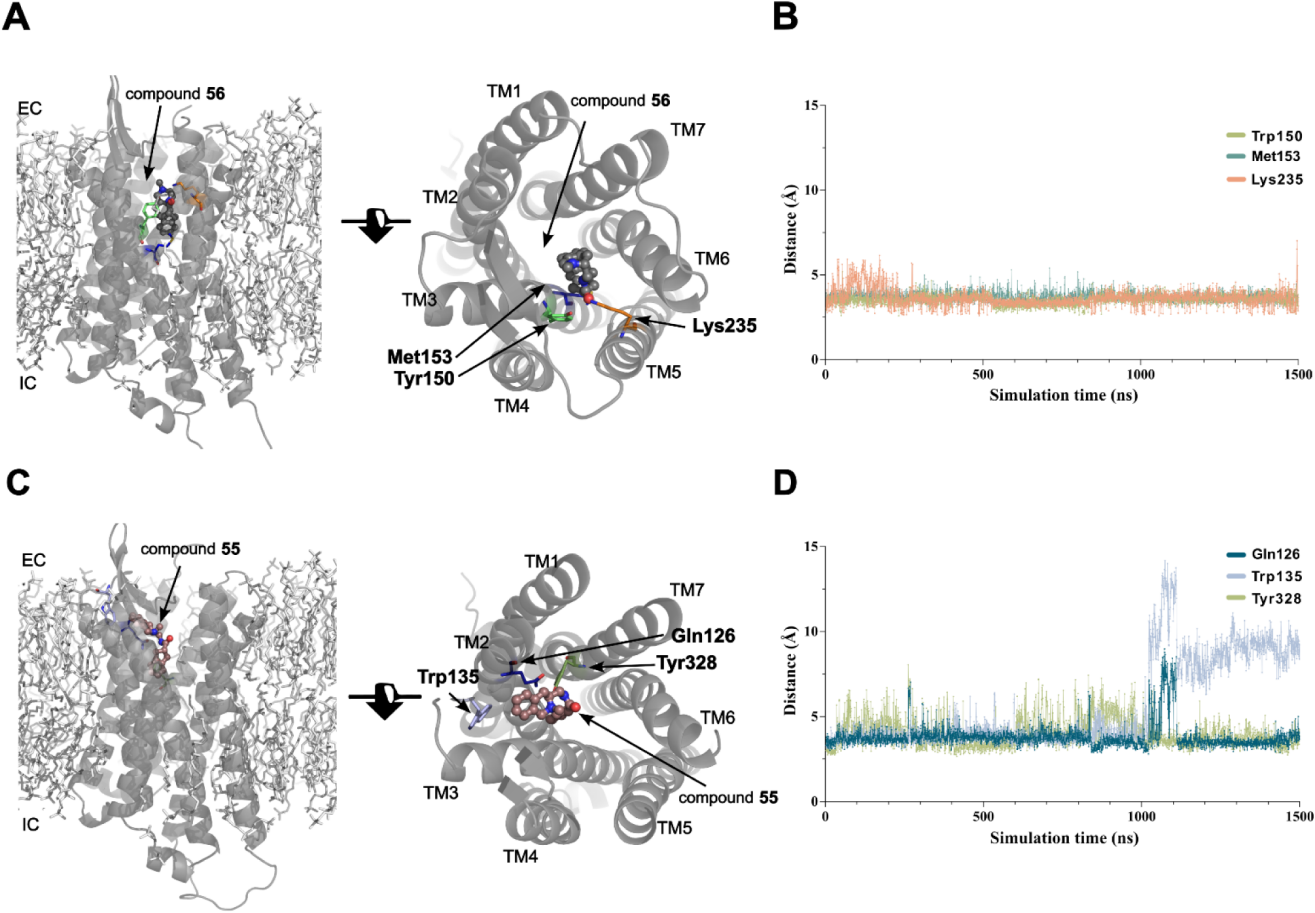
Docking and molecular dynamics simulations of compounds **55** and **56** with MOR (PDB ID: 4DKL). (A) Binding pose of compound **56** in MOR after a 1.5 μs molecular dynamics simulation. Compound **56** is shown as gray sticks with gray carbon atoms; nitrogen atoms are shown in blue, and oxygen atoms in red. MOR is shown in gray. (B) Molecular dynamics trajectory analysis of the interactive residues Tyr150, Lys235, and Met153 with compound **56** during the 1.5 μs dynamics simulation shows the time-dependent minimal distance (Å) between the indicated residues and **56**. (C) The binding pose of compound **55** in MOR after a 1.5 μs molecular dynamics simulation. Compound **55** is shown as brown sticks with brown carbon atoms; nitrogen atoms are shown in blue, and oxygen atoms in red. MOR is shown in gray. (D) Molecular dynamics trajectory analysis of the interactive residues Gln126, Trp135, and Tyr328 with compound **55** over a 1.5 μs simulation shows the time-responsive minimal distance between these residues and **55**. Abbreviations: EC, extracellular; IC, intracellular.

### Pharmacokinetic Profile of Compound **56**

Brain and plasma concentrations of compound **56** were measured after s.c. administration. Mice were treated with 1 mg/kg of compound **56**, and plasma and brain tissue samples were collected at each time point (15 min, 30 min, 1, 2, 4, 6, 8, 16, and 24 h), and the concentrations of compound **56** were measured by liquid chromatography/tandem mass spectrometry. The analysis of the pharmacokinetic profile of compound **56** is listed in Table 4, showing that the maximum concentration (*C*_max_) of **56** in the brain was 3.4-fold higher than it is in plasma. Also, the integration of the concentration–time response curve of **56** (AUC_(0–inf)_) in the brain was 4.7-fold higher than in plasma. In addition, the *C*_max_ value of **56** was approximately 1.13 × 10^6^- and 4839-fold of the EC_50_ values in the FLIPR Ca^2+^ and cAMP assays, respectively. These results indicate that compound **56** effectively penetrated the blood–brain barrier and produced potent *in vivo* antinociception. A small dose of compound **56** (0.1 mg/kg), which is 3.7-fold of the ED_50_ value of compound **56** in the thermally stimulated antinociceptive mouse model, was administered subcutaneously to mice, and the concentration of compound **56** in plasma and brain was measured at 0.5 and 1 h. The concentrations of compound **56** in the brain at 30 min and 1 h were 72.4 and 21.2 ng/mg, respectively, both of which were higher than those in plasma (11.7 and 2.1 ng/mL). The data are shown in Table S1 in the Supporting Information. These results indicated that the brain exposure of compound **56** was sufficient for MOR activation in most of the pharmacological studies of this work.

**Table 4:** Plasma and Brain Concentration Profiles of Compound 56^a^

	*T*_1/2_ (h)	*C*_max_ (ng/mL)	*T*_max_ (h)	AUC_(0‑inf)_ (ng·h/mL)
**56** in plasma	0.2 ± 0.0	429 ± 98	0.1 ± 0.0	181 ± 21
	*T*_1/2_ (h)	*C*_max_ (ng/g)	*T*_max_ (h)	AUC_(0‑inf)_ (ng·h/g)
**56** in brain	0.3 ± 0.0	1457 ± 214	0.3 ± 0.0	856 ± 235

a After a single injection of compound **56** (1 mg/kg, s.c.), plasma and brain samples were collected from each mouse at the indicated time points (15 and 30 min; 1, 2, 4, 6, 8, 16, and 24 h). The indicated parameters were calculated using a noncompartmental method. *n* = 3 per group. Values indicate the mean ± SD. SD, standard deviation.

### Physicochemical Properties of Compound **56**

The physicochemical properties of compound **56** were evaluated using polarized light microscopy (PLM), dynamic vapor sorption (DVS), Karl Fischer (KF) titration, pH solution stability, and solid-state stability. As shown in Figure 5A, PLM analysis showed that compound **56** appeared as birefringent crystalline particles under crossed polarized light. The crystals exhibited irregular plate- to prismatic-like morphologies, with sizes ranging from approximately 20 to 40 μm. In the DVS experiment (Figure 5B), compound **56** was placed in a 25 °C temperature-controlled chamber, and the relative humidity (RH) of this chamber was increased from 5% to 95% with a 5% gradient change, and vice versa, for each cycle. Compound **56** was subjected to a two-cycle isotherm DVS study, and the results indicated that compound **56** was a slightly hygroscopic crystal with a 0.92% weight change of water sorption at 80% RH. The water content of compound **56** was determined by KF titration at RH 64% in three independent tests, with sodium tartrate dihydrate used as the control standard. The results indicated that the water content of compound **56** was 0.64% (Figure 5C). Compound **56** was incubated in 40 °C aqueous solutions at various pH values with or without an oxidizing agent for 24 h, including 0.1 N HCl, pH 2, 4, 6, 8, pH 2 plus 0.3% H_2_O_2_, and pH 8 plus 0.3% H_2_O_2_. The remaining compounds were monitored by high-performance liquid chromatography (HPLC) every 4 h. The results are shown in Figure 5D (the percentage remaining at each time point for all tested conditions is shown in Table S2); compound **56** remained stable in 0.1 N HCl, pH 2, 4, 6, 8, pH 2 plus 0.3% H_2_O_2_ conditions, and approximately 88% remained at pH 8 with 0.3% H_2_O_2_ condition.Compound **56** was subjected to 7- and 14-day solid-state stability tests at 25 °C with light, 40 °C, 40 °C with RH 75%, and 70 °C conditions. Significant degradation of compound **56** was observed at Day 7 and Day 14 in 25 °C with light and 70 °C conditions. Compound **56** was about 93.3% and 86.7% remaining in these conditions at Day 14, respectively. The solid-state stability and HPLC conditions for compound **56** monitoring are listed in the Supporting Information (Tables S3 and S4).

**Figure 5:**
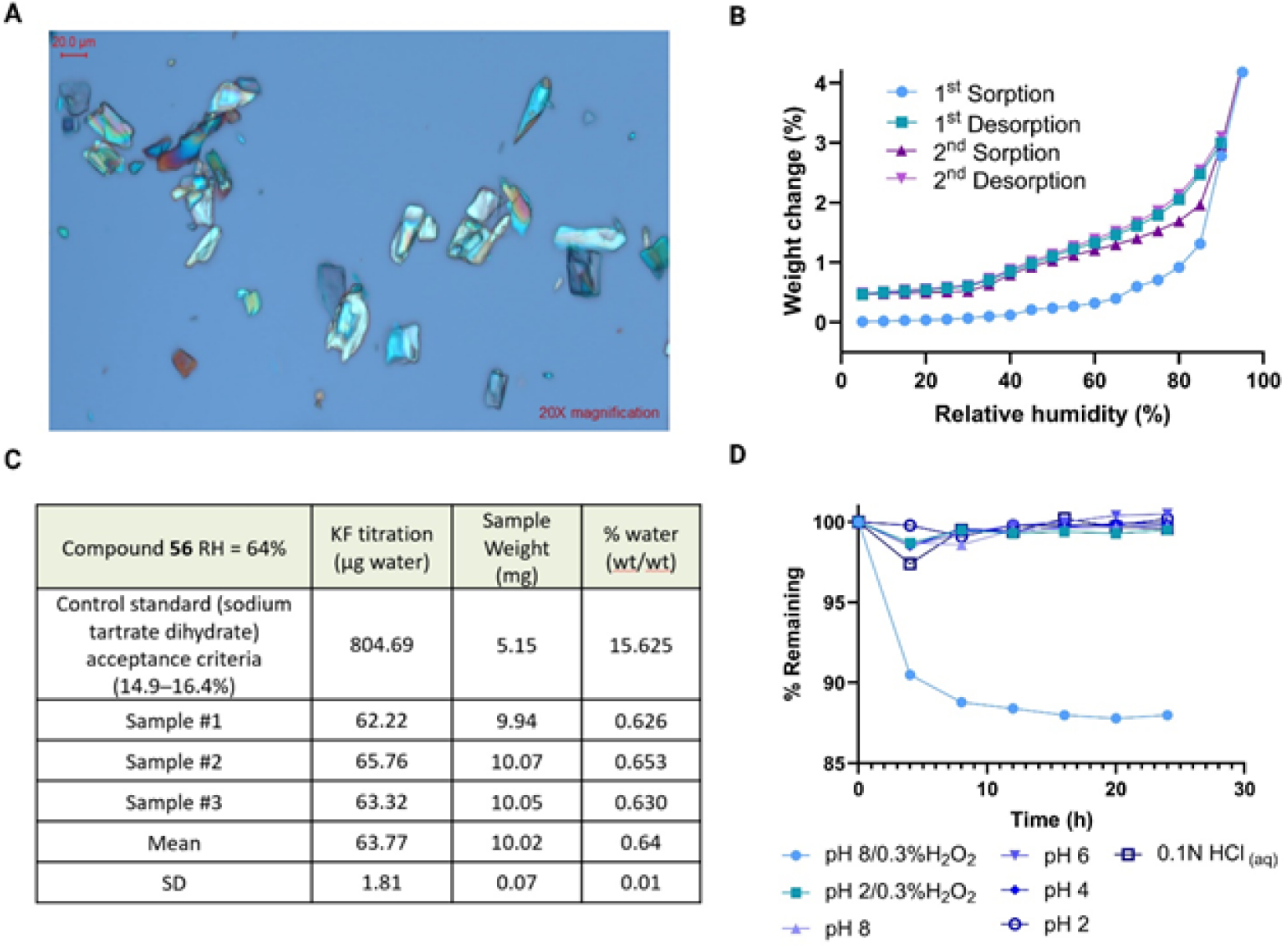
(A) PLM graph of compound **56**. (B) DVS isotherm plot of compound **56** in a two-cycle DVS experiment. (C) Three independent KF titration experiments to determine the water content of compound **56**. KF titration (μg water) = sample weight (mg) × %water × 1000 (μg/mg). (D) Aqueous solution stability of compound **56**. Test compounds were incubated in pH 1 (0.1 N HCl), 2, 4, 6, and 8 solutions, and 0.3% H_2_O_2_ aqueous solutions at pH 2 and 8.

### *In Vitro* Safety Profile of Compound **56**

The *in vitro* safety profiling of compound **56** was performed by evaluating *N*-methyl-*D*-aspartate receptor (NMDAR) ion channel blockage, cytochrome P450 (CYP) enzyme inhibition, and human ether-à-go-go-related gene (hERG) channel inhibition. NMDAR and hERG channels modulate the dynamics of essential ions in cells, such as calcium and potassium. Thus, inhibition of NMDAR or hERG channels usually causes neurotoxicity or acute cardiovascular toxicity, respectively.^42,43^ CYP enzymes are crucial for drug metabolism, and their inhibition may interfere with the metabolism of other drugs, causing potential toxicity. Assays were conducted by an outsourced research organization (Eurofins DiscoverX Co.). Compound **56** failed to block the NMDAR ion channel at 3 μM (Figure S4) and inhibited CYP1A, 2B6, 2C8, 2C9, and 3A with IC_50_ values > 100 μM (Figure S5). Compound **56** inhibited CYP2C19 and 2D6 with IC_50_ values of 26 and 2.1 μM, respectively (Figure S5). These corresponded to 27,956-fold and 2258-fold higher values than its MOR agonist EC_50_ in the cAMP assay, and 6.5 × 10^6^- and 5.25 × 10^5^-fold higher than its EC_50_ in the FLIPR Ca^2+^ assay, respectively. Compound **56** inhibited hERG with an IC_50_ of 1.92 μM (Figure S6), which was 2064-fold and 4.8 × 10^5^-fold higher than its EC_50_ values in the cAMP and FLIPR Ca^2+^ assays, respectively. These results revealed that compound **56** has a wide therapeutic window for potential clinical use.

## Discussion and Conclusion

The core structure of **56** differed from that of our previously developed compound, BPR1M97, by shifting the carboxamide moiety from C1 to C3. A SAR study of *N*-(1,2,3,4-tetrahydro-3-isoquinolinylmethyl)­benzamides revealed that the unsubstituted, 5-bromo, and 6-methyl tetrahydroisoquinoline cores with an indanyl or phenylethyl benzamide moiety exhibited potent and effective MOR agonism. Further SAR studies of compounds **55**–**62**, the enantiomeric isomers of **31** and **52**–**54** indicated that the *R*-configuration at C3 was favorable for MOR agonism. Compound **56** was the most potent compound in the FLIPR Ca^2+^ assay and had the most potent *in vivo* antinociceptive effect among compounds **56**, **60,** and **62**. Compound **56** exhibited full MOR agonism through both cAMP and β-arrestin-2 pathways, full NOP agonism through the cAMP pathway, weak DOR agonism through both cAMP and β-arrestin-2 pathways, and weak KOR agonism via the cAMP pathway in the *in vitro* assays. Molecular dynamics simulation of compounds **55** and **56** showed that the binding positions of compounds **55** and **56** within the MOR binding pocket were distinctly different. The interacting residues of MOR/**56** were Tyr150, Met153, and Lys235 at the bottom of the crevices formed by TM3 and TM5, and those of MOR/**55** were Gln126, Trp135, and Tyr328 toward the top of the crevices formed by TM2 and TM3. In the 1.5 μs molecular dynamics simulation, the trajectory analysis showed that the interactive residues of MOR maintained smaller fluctuations with **56** than with **55**.

Although compound **56** acted as an MOR agonist with no clear preference between cAMP and β-arrestin-2 signaling in our assays, the present data do not support a generalized conclusion that it possesses an intrinsically broader safety profile than TRV130. TRV130 (Olinvyk), a clinically approved MOR G protein-biased agonist, was developed to reduce β-arrestin-mediated adverse effects such as respiratory depression and constipation. TRV130 activates MOR with an EC_50_ of 8 nM in a cAMP assay, demonstrating 6.25-fold greater potency than morphine.^31^ The antinociceptive ED_50_ of TRV130 was 0.295 mg/kg, which was higher than that of compound **56** (ED_50_ = 0.027 mg/kg). The difference in antinociceptive potency may be related to the lower *in vitro* MOR potency of TRV130, as reflected by its higher EC_50_ values in the cAMP and FLIPR Ca^2+^ assays. Specifically, the EC_50_ values of TRV130 were 3.1 nM in the cAMP assay^44^ and 6.8 nM in the FLIPR Ca^2+^ assay (Table 2). Because compound **56** displayed an approximately 11-fold lower antinociceptive ED_50_ than TRV130, some apparent differences in nonwithdrawal adverse-effect readouts may reflect dose sparing associated with its higher antinociceptive potency, rather than an intrinsically wider therapeutic window. While early trials showed promise in acute pain management,^31^ later clinical data revealed that TRV130 causes severe side effects, including dose-dependent respiratory suppression and abuse potential, limiting its therapeutic application.^45^ In contrast, compound **56**, which showed no clear preference between cAMP and β-arrestin-2 signaling at MOR in our assays, consistently produced milder withdrawal signs than TRV130; this was the most prominent adverse-effect difference observed in the present study. Although compound **56** also showed smaller changes in oxygen saturation, gastrointestinal transit, CPP, and tolerance-related measures in some comparisons, these differences were less pronounced and should be interpreted cautiously because they may be influenced, at least in part, by the substantially lower doses required for compound **56** to achieve antinociception. Pharmacological profiling further showed that compound **56** exhibited a 56.8-fold preference for MOR over NOP activation in the cAMP assay. At antinociceptive doses, it was also capable of engaging NOP signaling and functioned as a G protein-biased NOP agonist, with minimal β-arrestin pathway activation. Emerging research supports the strategy of NOP-biased or mixed MOR/NOP agonism as a promising approach for opioid development. For example, Ding et al. reported that dual MOR/NOP agonists, such as cebranopadol and AT-121, provided potent antinociception with significantly fewer side effects in preclinical studies.^46^ Similarly, Bird et al. demonstrated MOR/NOP receptor interactions and the potential of dual acting or biased ligands to achieve antinociception with minimized liabilities.^47^ Thus, compound **56**, which showed no clear preference between cAMP and β-arrestin-2 signaling at MOR in our assays and partial NOP activation, may represent an alternative approach to pain management beyond conventional MOR-biased strategies. While compound **56** showed partial NOP activation in the cAMP assay and minimal β-arrestin recruitment, the present study was designed primarily to characterize its *in vitro* signaling properties and *in vivo* pharmacological profile, rather than to establish a direct causal relationship between individual signaling pathways and the observed behavioral outcomes. Therefore, although the reduced side-effect profile of compound **56** may be associated with its mixed MOR/NOP pharmacology, the specific contribution of NOP receptor activation to these effects was not directly demonstrated in the current work. Further mechanistic studies, such as pharmacological blockade or genetic disruption of NOP signaling, will be required to clarify the extent to which NOP engagement contributes to the improved safety profile of compound **56**.

The pharmacokinetic profile of compound **56** showed that its distributed concentration in the brain was higher than that in the plasma and much higher than the EC_50_ values of compound **56** in the FLIPR Ca^2+^ and cAMP assays. The physicochemical study of compound **56** validated it as a highly stable, slightly hygroscopic, low-moisture-containing, crystalline solid that can tolerate 40 °C aqueous buffer solutions with various pH values from 1 to 8 and pH 2 plus 0.03% H_2_O_2_ for 24 h. The *in vitro* safety screens of NMDAR ion channel blockage, CYP enzyme inhibition, and hERG channel inhibition suggested that compound **56** was safe at its *in vivo* effective dose. Together, these properties demonstrated that compound **56** merits further development and was designated as BPR1M492.

## Experimental Section

### Chemistry

#### General Remarks

Chemical reagents and organic solvents were purchased from commercial vendors and used as received without purification unless indicated otherwise. The reactions were carried out under argon and monitored using thin-layer chromatography plates (Merck 60 F_254_ glass-backed plates precoated with silica gel). A Teledyne Isco CombiFlash Automated Flash Chromatography system was used for automatic column chromatography, eluting with organic solvents as indicated. NMR spectra were recorded by Varian Mercury-300, Varian Mercury-400, Bruker AVNEO 600 MHz, and Bruker AVNEO 400 MHz spectrometers in deuterated solvents. Chemical shifts (δ) are reported in ppm relative to the residual solvent peaks. Multiplicities: s (singlet), br s (broad singlet), d (doublet), t (triplet), br t (broad triplet), q (quartet), quin (quintet), dd (doublet of doublets), tt (triplet of triplets), ddd (doublet of doublet of doublets), dt (doublet of triplets), and m (multiplet). Low-resolution mass spectrometry-electrospray ionization (ESI) spectra were recorded using an Agilent 1260 Infinity II UPLC/MSD 6125 B. ESI ionization time-of-flight mass spectrometry (ESI-TOF MS) data were collected using an Impact HD Q-TOF mass spectrometer (Bruker, Germany) equipped with an ESI source to detect positive and negative ions. Mass spectra of the final product solutions at pH 7.5 were acquired by direct infusion (2 μL). ESI (+) parameters included an ion spray voltage of 4.5 kV, a capillary temperature of 200 °C, and a 6 L/min sheath gas flow rate. Mass spectra were collected over a mass range of 50–1500 *m*/*z* at a resolving power of 40,000. The collected data were analyzed using Compass DataAnalysis 4.1 (Bruker, Germany). The specific optical rotation was recorded using a JASCO P-1020 polarimeter equipped with a sodium lamp. HPLC Purity: Waters Alliance e2695 HPLC separation module using a C_18_ column (XBridge 5 μm, 4.6 mm × 150 mm), operating at 25 °C. Elution was carried out using water containing 0.1% trifluoroacetic acid as mobile phase A and acetonitrile as mobile phase B. Elution conditions: 0–23.0 min, 10% phase A + 90% phase B; at 23.5–34.0 min, 90% phase A + 10% phase B. The flow rate of the mobile phase was 0.7 mL/min, unless otherwise indicated. The injection volume of the sample was 5 μL. The peaks were detected at 266 nm. UPLC Purity: Waters Acquity UPLC H-class system using an ACQUITY UPLC BEH-C18 column (1.7 μm, 2.1 mm × 50 mm). Elution was carried out using water containing 0.1% formic acid + 10 mM NH_4_OAc as mobile phase A and acetonitrile as mobile phase B. Elution conditions: 0.00–0.15 min, 90% phase A + 10% phase B; at 0.15–5.15 min, a gradient condition from 90% phase A + 10% phase B to 10% phase A + 90% phase B; at 5.15–6.35 min, 10% phase A + 90% phase B; at 6.35–6.50 min, a gradient condition from 10% phase A + 90% phase B to 90% phase A + 10% phase B; at 6.50–8.50 min, 90% phase A + 10% phase B. The flow rate of the mobile phase was 0.5 mL/min. The injection volume of the sample was 5 μL. Peaks were detected at 254 nm. The purity of all tested compounds was >95%, except for **22** (94%), **23** (94%), **24** (90%), **28** (90%), and **41** (94%).

#### General Procedure I for Amide Bond Formation (For Preparation of **16–25**, **27–44**, **46**, **49**, **50–62**)

To a stirring solution of the amine intermediate (1.00 equiv) in dichloromethane (the concentration of the intermediate was 0.05–0.2 M) were added the acid (0.90–1.00 equiv), 1-(3-(dimethylamino)­propyl)-3-ethylcarbodiimide hydrochloride (1.50 equiv), 1-hydroxybenzotriazole hydrate (1.50 equiv), and *N*,*N*-diisopropylethylamine (3.50 equiv) at rt. This reaction mixture was then stirred at rt for 16–18 h. After the completion of the reaction, the crude mixture was diluted with dichloromethane, extracted with saturated brine, and the organic layer was concentrated under reduced pressure. The crude product was purified using Isco Combi-Flash Companion column chromatography or silica gel flash column chromatography to obtain the desired product.

#### General Procedure II for Amide Bond Formation (For Preparation of **26**, **45**)

To a stirring solution of the amine intermediate (1.00 equiv) and triethylamine (1.20 equiv) in dichloromethane (the concentration of the intermediate was 0.1 M), acyl chloride (1.10 equiv) was added, and the reaction mixture was stirred at rt for 1 h. After the completion of the reaction, the crude mixture was diluted with dichloromethane, extracted with saturated brine, and the organic layer was concentrated under reduced pressure. The crude product was purified using an Isco Combi-Flash Companion column chromatograph or silica gel flash column chromatography to obtain the desired product.

#### 4-Ethyl-*N*-[(2-methyl-1,2,3,4-tetrahydroisoquinolin-3-Yl)­methyl]­benzamide (**16**)

The compound was prepared by general procedure I with (2-methyl-1,2,3,4-tetrahydroisoquinolin-3-yl)­methanamine **2c** (95.0 mg, 0.539 mmol), 4-ethylbenzoic acid (81.0 mg, 0.539 mmol), 1-(3-(dimethylamino)­propyl)-3-ethylcarbodiimide hydrochloride (207 mg, 1.08 mmol), 1-hydroxybenzotriazole hydrate (148 mg, 1.08 mmol), and *N*,*N*-diisopropylethylamine (235 μL, 1.35 mmol) in dichloromethane (10.8 mL). The crude product was purified by flash column chromatography (SiO_2_, 3% methanol in dichloromethane) to give the desired product, 4-ethyl-*N*-[(2-methyl-1,2,3,4-tetrahydroisoquinolin-3-yl)­methyl]­benzamide **16,** as a pink syrup (107 mg, 64%). ^1^H NMR (400 MHz, DMSO-*d*_6_) δ: 8.35 (t, 1H), 7.77 (d, 2H), 7.29 (d, 2H), 7.14–7.08 (m, 3H), 7.07–7.03 (m, 1H), 3.78 (d, 1H), 3.63–3.50 (m, 2H), 3.24–3.12 (m, 1H), 2.92 (quin, 1H), 2.82 (dd, 1H), 2.73–2.60 (m, 3H), 2.41 (s, 3H), 1.19 (t, 3H); ^13^C NMR (150 MHz, DMSO-*d*_6_) δ: 166.22, 147.11, 134.26, 133.40, 132.05, 128.70, 127.58, 127.29, 126.09, 126.00, 125.54, 57.54, 54.87, 40.86, 30.23, 28.00, 15.39. LC-MS (ESI) *m*/*z* 309.1 [M + H]^+^; HRMS–ESI (*m*/*z*): [M + H]^+^ calcd for C_20_H_25_N_2_O, 309.1961; found, 309.1968; HPLC purity = 95% (*t*_R_ = 12.2 min).

#### 4-(Ethylsulfanyl)-*N*-[(2-methyl-1,2,3,4-tetrahydroisoquinolin-3-yl)­methyl]­benzamide (**17**)

The compound was prepared by general procedure I with (2-methyl-1,2,3,4-tetrahydroisoquinolin-3-yl)­methanamine **2c** (95.0 mg, 0.539 mmol), 4-(ethylthio)­benzoic acid (98.0 mg, 0.539 mmol), 1-(3-(dimethylamino)­propyl)-3-ethylcarbodiimide hydrochloride (207 mg, 1.08 mmol), 1-hydroxybenzotriazole hydrate (148 mg, 1.08 mmol), and *N*,*N*-diisopropylethylamine (235 μL, 1.35 mmol) in dichloromethane (10.8 mL). The crude product was purified by flash column chromatography (SiO_2_, 3% methanol in dichloromethane) to give the desired product, 4-(ethylsulfanyl)-*N*-[(2-methyl-1,2,3,4-tetrahydroisoquinolin-3-yl)­methyl]­benzamide **17,** as a pink syrup (166 mg, 91%). ^1^H NMR (400 MHz, DMSO-*d*_6_) δ: 8.40 (t, 1H), 7.78 (d, 2H), 7.35 (d, 2H), 7.15–7.08 (m, 3H), 7.08–7.01 (m, 1H), 3.78 (d, 1H), 3.65–3.48 (m, 2H), 3.24–3.11 (m, 1H), 3.04 (q, 2H), 2.92 (quin, 1H), 2.82 (dd, 1H), 2.68 (dd, 1H), 2.42 (s, 3H), 1.26 (t, 3H); ^13^C NMR (150 MHz, Acetone-*d*_6_) δ: 167.09, 142.31, 135.48, 134.71, 132.71, 129.63, 128.66, 127.67, 127.13, 127.03, 126.61, 59.07, 56.59, 41.46, 41.24, 31.22, 26.76, 14.52. LC-MS (ESI) *m*/*z* 341.1 [M + H]^+^; HRMS–ESI (*m*/*z*): [M + H]^+^ calcd for C_20_H_25_N_2_OS, 341.1682; found, 341.1690; HPLC purity = 95% (*t*_R_ = 15.0 min).

#### 2-Chloro-5-fluoro-*N*-[(2-methyl-1,2,3,4-tetrahydroisoquinolin-3-yl)­methyl]­benzamide (**18**)

The compound was prepared by general procedure I with (2-methyl-1,2,3,4-tetrahydroisoquinolin-3-yl)­methanamine **2c** (60.0 mg, 0.340 mmol), 2-chloro-5-fluorobenzoic acid (59.4 mg, 0.340 mmol), 1-(3-(dimethylamino)­propyl)-3-ethylcarbodiimide hydrochloride (84.6 mg, 0.443 mmol), 1-hydroxybenzotriazole hydrate (50.6 mg, 0.374 mmol), and *N*,*N*-diisopropylethylamine (80.0 μL, 0.511 mmol) in dichloromethane (5.0 mL). The crude product was purified by Isco Combi-Flash Companion column chromatography (SiO_2_, 0–5% methanol in dichloromethane) to give 2-chloro-5-fluoro-*N*-[(2-methyl-1,2,3,4-tetrahydroisoquinolin-3-yl)­methyl]­benzamide **18** (trace). ^1^H NMR (400 MHz, CDCl_3_) δ: 7.41–7.30 (m, 2H), 7.19–7.00 (m, 5H), 6.97 (br s, 1H), 3.89 (d, 1H), 3.78–3.62 (m, 2H), 3.55 (dt, 1H), 3.07–2.94 (m, 1H), 2.85 (d, 2H), 2.43 (s, 3H); ^13^C NMR (150 MHz, Acetone*-d*_6_) δ: 165.13, 161.01 (d, *J*_C–F_ = 244.8 Hz), 138.70 (d, *J*_C–F_ = 6.9 Hz), 134.57, 133.73, 131.59 (d, *J*_C–F_ = 8.3 Hz), 128.62, 126.12, 126.06, 125.71 (d, *J*_C–F_ = 3.5 Hz), 125.65, 117.58 (d, *J*_C–F_ = 23.1 Hz), 116.08 (d, *J*_C–F_ = 24.3 Hz), 57.97, 55.78, 40.54, 40.29, 30.30. LC-MS (ESI) *m*/*z* 333.1 [M + H]^+^; HRMS–ESI (*m*/*z*): [M + H]^+^ calcd for C_18_H_19_ClFN_2_O, 333.1164; found, 333.1173; UPLC purity = 97% (*t*_R_ = 1.9 min).

#### 5-Fluoro-2-methyl-*N*-[(2-methyl-1,2,3,4-tetrahydroisoquinolin-3-yl)­methyl]­benzamide (**19**)

The compound was prepared by general procedure I with (2-methyl-1,2,3,4-tetrahydroisoquinolin-3-yl)­methanamine **2c** (60.0 mg, 0.340 mmol), 5-fluoro-2-methylbenzoic acid (52.5 mg, 0.340 mmol), 1-(3-(dimethylamino)­propyl)-3-ethylcarbodiimide hydrochloride (84.6 mg, 0.443 mmol), 1-hydroxybenzotriazole hydrate (50.6 mg, 0.374 mmol), and *N*,*N*-diisopropylethylamine (80.0 μL, 0.511 mmol) in dichloromethane (5.0 mL). The crude product was purified by Isco Combi-Flash Companion column chromatography (SiO_2_, 0–5% methanol in dichloromethane) to give 5-fluoro-2-methyl-*N*-[(2-methyl-1,2,3,4-tetrahydroisoquinolin-3-yl)­methyl]­benzamide **19** (18.6 mg, 17.5%). ^1^H NMR (400 MHz, CDCl_3_) δ: 7.23–6.91 (m, 8H), 3.97 (d, 1H), 3.87 (d, 1H), 3.75 (dt, 1H), 3.57 (ddd, 1H), 3.18 (br s, 1H), 2.94 (dd, 1H), 2.86 (dd, 1H), 2.50 (s, 3H), 2.40 (s, 3H); ^13^C NMR (150 MHz, Acetone*-d*_6_) δ: 168.06, 160.52 (d, *J*_C–F_ = 241.5 Hz), 138.96 (d, *J*_C–F_ = 6.2 Hz), 134.62, 133.75, 132.26 (d, *J*_C–F_ = 7.5 Hz), 131.7 (d, *J*_C–F_ = 3.3 Hz), 128.65, 126.15, 126.04, 125.65, 115.74 (d, *J*_C–F_ = 20.7 Hz), 113.72 (d, *J*_C–F_ = 22.4 Hz), 58.11, 55.72, 40.32, 40.22, 30.22, 18.26. LC-MS (ESI) *m*/*z* 313.1 [M + H]^+^; HRMS–ESI (*m*/*z*): [M + H]^+^ calcd for C_19_H_22_FN_2_O, 313.1711; found, 313.1713; HPLC purity = 99% (*t*_R_ = 10.9 min).

#### 2,5-Dichloro-*N*-[(2-methyl-1,2,3,4-tetrahydroisoquinolin-3-yl)­methyl]­benzamide (**20**)

The compound was prepared by general procedure I with (2-methyl-1,2,3,4-tetrahydroisoquinolin-3-yl)­methanamine **2c** (60.0 mg, 0.340 mmol), 2,5-dichlorobenzoic acid (65.0 mg, 0.340 mmol), 1-(3-(dimethylamino)­propyl)-3-ethylcarbodiimide hydrochloride (84.6 mg, 0.443 mmol), 1-hydroxybenzotriazole hydrate (50.6 mg, 0.374 mmol), and *N*,*N*-diisopropylethylamine (80.0 μL, 0.511 mmol) in dichloromethane (5.0 mL). The crude product was purified by Isco Combi-Flash Companion column chromatography (SiO_2_, 0–5% methanol in dichloromethane) to give 2,5-dichloro-*N*-[(2-methyl-1,2,3,4-tetrahydroisoquinolin-3-yl)­methyl]­benzamide **20** (42.4 mg, 36%) as a white solid. ^1^H NMR (400 MHz, CDCl_3_) δ: 8.35 (br s, 1H), 7.53 (d, 1H), 7.36–7.07 (m overlapped with CDCl_3_ at 7.24, 6H), 4.19 (d, 1H), 4.10 (d, 1H), 3.88 (dt, 1H), 3.69 (ddd, 1H), 3.53 (br s, 1H), 3.14–2.90 (m, 2H), 2.67 (s, 3H); ^13^C NMR (150 MHz, Acetone*-d*_6_) δ: 165.11, 138.53, 134.57, 133.72, 132.25, 131.37, 130.50, 129.18, 128.91, 128.63, 126.14, 126.08, 125.67, 57.98, 55.79, 40.56, 40.34, 30.35. LC-MS (ESI) *m*/*z* 349.1 [M + H]^+^; HRMS–ESI (*m*/*z*): [M + H]^+^ calcd for C_18_H_19_Cl_2_N_2_O, 349.0869; found, 349.0868; HPLC purity = 98% (*t*_R_ = 11.7 min).

#### 3-(4-Methylphenyl)-*N*-[(2-methyl-1,2,3,4-tetrahydroisoquinolin-3-yl)­methyl]­propanamide (**21**)

The compound was prepared by general procedure I with (2-methyl-1,2,3,4-tetrahydroisoquinolin-3-yl)­methanamine **2c** (60.0 mg, 0.340 mmol), 3-(4-methylphenyl)­propanoic acid (55.9 mg, 0.340 mmol), 1-(3-(dimethylamino)­propyl)-3-ethylcarbodiimide hydrochloride (84.6 mg, 0.443 mmol), 1-hydroxybenzotriazole hydrate (50.6 mg, 0.374 mmol), and *N*,*N*-diisopropylethylamine (80.0 μL, 0.511 mmol) in dichloromethane (5.0 mL). The crude product was purified by Isco Combi-Flash Companion column chromatography (SiO_2_, 0–5% methanol in dichloromethane) to give 3-(4-methylphenyl)-*N*-[(2-methyl-1,2,3,4-tetrahydroisoquinolin-3-yl)­methyl]­propanamide **21** (100 mg, 91%). ^1^H NMR (400 MHz, CDCl_3_) δ: 7.17–7.11 (m, 2H), 7.10–6.99 (m, 6H), 5.93 (br s, 1H), 3.79 (d, 1H), 3.64 (d, 1H), 3.47–3.27 (m, 2H), 2.92 (t, 2H), 2.81–2.71 (m, 1H), 2.71–2.58 (m, 2H), 2.48 (t, 2H), 2.30 (s, 3H), 2.29 (s, 3H); ^13^C NMR (150 MHz, Acetone*-d*_6_) δ: 171.53, 138.50, 135.03, 134.60, 133.92, 128.87, 128.56, 128.26, 126.04, 125.97, 125.54, 58.20, 56.19, 40.28, 40.00, 37.71, 31.11, 30.37, 20.15. LC-MS (ESI) *m*/*z* 323.1 [M + H]^+^; HRMS–ESI (*m*/*z*): [M + H]^+^ calcd for C_21_H_27_N_2_O, 323.2118; found, 323.2117; HPLC purity = 95% (*t*_R_ = 12.2 min).

#### 3-(3-Methylphenyl)-*N*-[(2-methyl-1,2,3,4-tetrahydroisoquinolin-3-yl)­methyl]­propanamide (**22**)

The compound was prepared by general procedure I with (2-methyl-1,2,3,4-tetrahydroisoquinolin-3-yl)­methanamine **2c** (60.0 mg, 0.340 mmol), 3-(3-methylphenyl)­propanoic acid (55.9 mg, 0.340 mmol), 1-(3-(dimethylamino)­propyl)-3-ethylcarbodiimide hydrochloride (84.6 mg, 0.443 mmol), 1-hydroxybenzotriazole hydrate (50.6 mg, 0.374 mmol), and *N*,*N*-diisopropylethylamine (80.0 μL, 0.511 mmol) in dichloromethane (5.0 mL). The crude product was purified by Isco Combi-Flash Companion column chromatography (SiO_2_, 0–5% methanol in dichloromethane) to give 3-(3-methylphenyl)-*N*-[(2-methyl-1,2,3,4-tetrahydroisoquinolin-3-yl)­methyl]­propanamide **22** (98.7 mg, 90%). ^1^H NMR (400 MHz, CDCl_3_) δ: 7.19–7.10 (m, 3H), 7.08–6.97 (m, 5H), 5.94 (br s, 1H), 3.79 (d, 1H), 3.64 (d, 1H), 3.42 (dt, 1H), 3.32 (dt, 1H), 2.93 (t, 2H), 2.82–2.71 (m, 1H), 2.71–2.58 (m, 2H), 2.49 (t, 2H), 2.30 (s, 3H), 2.29 (s, 3H); ^13^C NMR (150 MHz, Acetone*-d*_6_) δ: 171.54, 141.51, 137.52, 134.59, 133.90, 129.08, 128.57, 128.16, 126.55, 126.03, 125.97, 125.55, 125.38, 58.20, 56.17, 40.30, 40.02, 37.61, 31.47, 30.38, 20.55. LC-MS (ESI) *m*/*z* 323.1 [M + H]^+^; HRMS–ESI (*m*/*z*): [M + H]^+^ calcd for C_21_H_27_N_2_O, 323.2118; found, 323.2122; HPLC purity = 94% (*t*_R_ = 12.1 min).

#### 3-(3-Fluorophenyl)-*N*-[(2-methyl-1,2,3,4-tetrahydroisoquinolin-3-yl)­methyl]­propanamide (**23**)

The compound was prepared by general procedure I with (2-methyl-1,2,3,4-tetrahydroisoquinolin-3-yl)­methanamine **2c** (60.0 mg, 0.340 mmol), 3-(3-fluorophenyl)­propanoic acid (57.2 mg, 0.340 mmol), 1-(3-(dimethylamino)­propyl)-3-ethylcarbodiimide hydrochloride (84.6 mg, 0.443 mmol), 1-hydroxybenzotriazole hydrate (50.6 mg, 0.374 mmol), and *N*,*N*-diisopropylethylamine (80.0 μL, 0.511 mmol) in dichloromethane (5.0 mL). The crude product was purified by Isco Combi-Flash Companion column chromatography (SiO_2_, 0–5% methanol in dichloromethane) to give 3-(3-fluorophenyl)-*N*-[(2-methyl-1,2,3,4-tetrahydroisoquinolin-3-yl)­methyl]­propanamide **23** (27.3 mg, 25%). ^1^H NMR (400 MHz, CDCl_3_) δ: 7.22–7.18 (m, 1H), 7.17–7.10 (m, 2H), 7.09–6.99 (m, 2H), 6.97 (d, 1H), 6.94–6.83 (m, 2H), 6.03 (br s, 1H), 3.80 (d, 1H), 3.67 (d, 1H), 3.45 (dt, 1H), 3.32 (ddd, 1H), 2.96 (t, 2H), 2.87–2.74 (m, 1H), 2.73–2.60 (m, 2H), 2.49 (t, 2H), 2.32 (s, 3H); ^13^C NMR (150 MHz, Acetone*-d*_6_) δ: 171.21, 162.82 (d, *J*_C–F_ = 241.7 Hz), 144.62 (d, *J*_C–F_ = 7.5 Hz), 134.57, 133.85, 129.93 (d, *J*_C–F_ = 8.4 Hz), 128.55, 126.05, 125.98, 125.56, 124.39 (d, *J*_C–F_ = 2.7 Hz), 115.05 (d, *J*_C–F_ = 20.9 Hz), 112.53 (d, *J*_C–F_ = 20.9 Hz), 58.17, 56.10, 40.25, 40.00, 37.05, 31.06, 30.30. LC-MS (ESI) *m*/*z* 327.1 [M + H]^+^; HRMS–ESI (*m*/*z*): [M + H]^+^ calcd for C_20_H_24_FN_2_O, 327.1867; found, 327.1878; HPLC purity = 94% (*t*_R_ = 11.4 min).

#### 3-(3-Chlorophenyl)-*N*-[(2-methyl-1,2,3,4-tetrahydroisoquinolin-3-yl)­methyl]­propanamide (**24**)

The compound was prepared by general procedure I with (2-methyl-1,2,3,4-tetrahydroisoquinolin-3-yl)­methanamine **2c** (60.0 mg, 0.340 mmol), 3-(3-chlorophenyl)­propanoic acid (62.8 mg, 0.340 mmol), 1-(3-(dimethylamino)­propyl)-3-ethylcarbodiimide hydrochloride (84.6 mg, 0.443 mmol), 1-hydroxybenzotriazole hydrate (50.6 mg, 0.374 mmol), and *N*,*N*-diisopropylethylamine (80.0 μL, 0.511 mmol) in dichloromethane (5.0 mL). The crude product was purified by Isco Combi-Flash Companion column chromatography (SiO_2_, 0–5% methanol in dichloromethane) to give 3-(3-chlorophenyl)-*N*-[(2-methyl-1,2,3,4-tetrahydroisoquinolin-3-yl)­methyl]­propanamide **24** (90.2 mg, 77%). ^1^H NMR (400 MHz, CDCl_3_) δ: 7.22–6.99 (m, 8H), 5.97 (br s, 1H), 3.79 (d, 1H), 3.65 (d, 1H), 3.43 (dt, 1H), 3.31 (ddd, 1H), 2.94 (t, 2H), 2.84–2.72 (m, 1H), 2.72–2.59 (m, 2H), 2.52–2.45 (m, 2H), 2.32 (S, 3H); ^13^C NMR (150 MHz, Acetone*-d*_6_) δ: 171.12, 144.18, 134.58, 133.85, 133.51, 129.86, 128.56, 128.42, 127.05, 126.03, 125.97, 125.94, 125.55, 58.16, 56.11, 40.27, 39.98, 37.04, 30.98, 30.31. LC-MS (ESI) *m*/*z* 343.0 [M + H]^+^; HRMS–ESI (*m*/*z*): [M + H]^+^ calcd for C_20_H_24_ClN_2_O, 343.1572; found, 343.1580; HPLC purity = 90% (*t*_R_ = 12.5 min).

#### *N*-[(5-Bromo-2-methyl-1,2,3,4-tetrahydroisoquinolin-3-yl)­methyl]-4-ethylbenzamide (**25**)

The compound was prepared by general procedure I with (5-bromo-2-methyl-1,2,3,4-tetrahydroisoquinolin-3-yl)­methanamine **3i** (47.0 mg, 0.184 mmol), 4-ethylbenzoic acid (27.7 mg, 0.184 mmol), 1-(3-(dimethylamino)­propyl)-3-ethylcarbodiimide hydrochloride (45.8 mg, 0.239 mmol), 1-hydroxybenzotriazole hydrate (27.4 mg, 0.203 mmol), and *N*,*N*-diisopropylethylamine (50.0 μL, 0.276 mmol) in dichloromethane (5.0 mL). The crude product was purified by Isco Combi-Flash Companion column chromatography (SiO_2_, 0–3% methanol in dichloromethane) to give *N*-[(5-bromo-2-methyl-1,2,3,4-tetrahydroisoquinolin-3-yl)­methyl]-4-ethylbenzamide **25** (63.6 mg, 89%). ^1^H NMR (400 MHz, DMSO-*d*_6_) δ: 8.40 (br t, 1H), 7.75 (d, 2H), 7.43 (d, 1H), 7.29 (d, 2H), 7.14–7.03 (m, 2H), 3.80 (d, 1H), 3.59 (d, 1H), 3.54 (dt, 1H), 3.26 (dt, 1H), 3.01–2.91 (m, 1H), 2.76 (dd, 1H), 2.64 (q, 2H), 2.57 (dd, 1H), 2.38 (s, 3H), 1.18 (t, 3H); ^13^C NMR (150 MHz, Acetone*-d*_6_) δ: 166.58, 147.54, 137.51, 133.54, 132.62, 130.00, 127.70, 127.29, 127.23, 125.74, 124.75, 58.04, 56.00, 40.60, 39.54, 31.14, 28.36, 14.94. LC-MS (ESI) *m*/*z* 387.1 [M + H]^+^; HRMS–ESI (*m*/*z*): [M + H]^+^ calcd for C_20_H_24_BrN_2_O, 387.1067; found, 387.1068; HPLC purity = 99% (*t*_R_ = 13.7 min).

#### *N*-[(5-Bromo-2-methyl-1,2,3,4-tetrahydroisoquinolin-3-yl)­methyl]-3,4-dichlorobenzamide (**26**)

The compound was prepared by general procedure II with (5-bromo-2-methyl-1,2,3,4-tetrahydroisoquinolin-3-yl)­methanamine **3i** (54.0 mg, 0.212 mmol), 3,4-dichlorobenzoyl chloride (44.0 mg, 0.212 mmol), and triethylamine (35.0 μL, 0.254 mmol) in dichloromethane (5.0 mL). The crude product was purified by Isco Combi-Flash Companion column chromatography (SiO_2_, 0–3% methanol in dichloromethane) to give *N*-[(5-bromo-2-methyl-1,2,3,4-tetrahydroisoquinolin-3-yl)­methyl]-3,4-dichlorobenzamide **26** (76.6 mg, 85%).^1^H NMR (400 MHz, DMSO-*d*_6_) δ: 8.69 (t, 1H), 8.06 (d, 1H), 7.81 (dd, 1H), 7.76 (d, 1H), 7.43 (dd, 1H), 7.14–7.05 (m, 2H), 3.80 (d, 1H), 3.61 (d, 1H), 3.55 (dt, 1H), 3.31–3.22 (m, 1H), 3.01–2.91 (m, 1H), 2.73 (dd, 1H), 2.58 (dd, 1H), 2.37 (s, 3H); ^13^C NMR (150 MHz, Acetone*-d*_6_) δ: 164.52, 137.41, 135.37, 134.57, 133.42, 131.94, 130.59, 130.03, 129.32, 127.34, 127.15, 125.76, 124.73, 57.94, 55.85, 40.79, 39.51, 31.02. LC-MS (ESI) *m*/*z* 426.9 [M + H]^+^; HRMS–ESI (*m*/*z*): [M + H]^+^ calcd for C_18_H_18_BrCl_2_N_2_O, 426.9974; found, 426.9984; HPLC purity = 98% (*t*_R_ = 14.3 min).

#### *N*-[(5-Bromo-2-methyl-1,2,3,4-tetrahydroisoquinolin-3-yl)­methyl]-3-phenylpropanamide (**27**)

The compound was prepared by general procedure I with (5-bromo-2-methyl-1,2,3,4-tetrahydroisoquinolin-3-yl)­methanamine **3i** (51.0 mg, 0.200 mmol), 3-phenylpropanoic acid (30.0 mg, 0.200 mmol), 1-(3-(dimethylamino)­propyl)-3-ethylcarbodiimide hydrochloride (49.7 mg, 0.260 mmol), 1-hydroxybenzotriazole hydrate (30.0 mg, 0.220 mmol), and *N*,*N*-diisopropylethylamine (50.0 μL, 0.300 mmol) in dichloromethane (5.0 mL). The crude product was purified by Isco Combi-Flash Companion column chromatography (SiO_2_, 0–3% methanol in dichloromethane) to give *N*-[(5-bromo-2-methyl-1,2,3,4-tetrahydroisoquinolin-3-yl)­methyl]-3-phenylpropanamide **27** (54.6 mg, 71%). ^1^H NMR (600 MHz, DMSO-*d*_6_) δ: 7.86 (br t, 1H), 7.43 (t, 1H), 7.28–7.17 (m, 4H), 7.14 (t, 1H), 7.11–7.06 (m, 2H), 3.75 (d, 1H), 3.53 (d, 1H), 3.36–3.27 (m, overlapped with H_2_O, 1H), 3.11 (dt, 1H), 2.80 (t, 2H), 2.70–2.60 (m, 2H), 2.54–2.46 (m, overlapped with DMSO-*d*_6_, 1H), 2.40 (t, 2H), 2.27 (s, 3H); ^13^C NMR (150 MHz, Acetone*-d*_6_) δ: 171.50, 141.66, 137.50, 133.64, 129.97, 128.31, 128.25, 127.25, 125.83, 125.63, 124.64, 58.21, 56.44, 40.09, 39.57, 37.55, 31.50, 31.33. LC-MS (ESI) *m*/*z* 387.0 [M + H]^+^; HRMS–ESI (*m*/*z*): [M + H]^+^ calcd for C_20_H_24_BrN_2_O, 387.1067; found, 387.1072; HPLC purity = 98% (*t*_R_ = 12.4 min).

#### *N*-[(5-Bromo-2-methyl-1,2,3,4-tetrahydroisoquinolin-3-yl)­methyl]-3-(3-fluorophenyl)­propanamide (**28**)

The compound was prepared by general procedure I with (5-bromo-2-methyl-1,2,3,4-tetrahydroisoquinolin-3-yl)­methanamine **3i** (46.0 mg, 0.180 mmol), 3-(3-fluorophenyl)­propanoic acid (30.0 mg, 0.180 mmol), 1-(3-(dimethylamino)­propyl)-3-ethylcarbodiimide hydrochloride (44.8 mg, 0.234 mmol), 1-hydroxybenzotriazole hydrate (26.8 mg, 0.198 mmol), and *N*,*N*-diisopropylethylamine (45.0 μL, 0.270 mmol) in dichloromethane (5.0 mL). The crude product was purified by Isco Combi-Flash Companion column chromatography (SiO_2_, 0–3% methanol in dichloromethane) to give *N*-[(5-bromo-2-methyl-1,2,3,4-tetrahydroisoquinolin-3-yl)­methyl]-3-(3-fluorophenyl)­propanamide **28** (55.4 mg, 76%).^1^H NMR (400 MHz, DMSO-*d*_6_) δ: 7.87 (t, 1H), 7.45–7.38 (m, 1H), 7.32–7.23 (m, 1H), 7.12–7.00 (m, 4H), 6.95 (t, 1H), 3.75 (d, 1H), 3.52 (d, 1H), 3.37–3.27 (m, overlapped with H_2_O, 1H), 3.17–3.05 (m, 1H), 2.83 (t, 2H), 2.70–2.57 (m, 2H), 2.52–2.45 (m, overlapped with DMSO-*d*_6_, 1H), 2.42 (t, 2H), 2.27 (s, 3H); ^13^C NMR (150 MHz, Acetone*-d*_6_) δ: 171.33, 162.82 (d, *J*_C–F_ = 243.0 Hz), 144.60 (d, *J*_C–F_ = 7.4 Hz), 137.46, 133.59, 129.98, 129.92, 127.27, 125.64, 124.65, 124.34 (d, *J*_C–F_ = 2.7 Hz), 115.02 (d, *J*_C–F_ = 21.0 Hz), 112.53 (d, *J*_C–F_ = 21.0 Hz), 58.19, 56.42, 40.14, 39.54, 37.05, 31.28, 31.08. LC-MS (ESI) *m*/*z* 405.1 [M + H]^+^; HRMS–ESI (*m*/*z*): [M + H]^+^ calcd for C_20_H_23_BrFN_2_O, 405.0972; found, 405.0979; HPLC purity = 90% (*t*_R_ = 12.8 min).

#### *N*-[(2,6-Dimethyl-1,2,3,4-tetrahydroisoquinolin-3-yl)­methyl]-4-(ethylsulfanyl)­benzamide (**29**)

The compound was prepared by general procedure I with (2,6-dimethyl-1,2,3,4-tetrahydroisoquinolin-3-yl)­methanamine **4i** (57.0 mg, 0.300 mmol), 4-(ethylthio)­benzoic acid (65.6 mg, 0.360 mmol), 1-(3-(dimethylamino)­propyl)-3-ethylcarbodiimide hydrochloride (74.7 mg, 0.390 mmol), 1-hydroxybenzotriazole hydrate (52.7 mg, 0.390 mmol), and *N*,*N*-diisopropylethylamine (160 μL, 0.918 mmol) in dichloromethane (3.0 mL). The crude product was purified by Isco Combi-Flash Companion column chromatography (SiO_2_, 0–6% methanol in dichloromethane) to give the desired product, *N*-[(2,6-dimethyl-1,2,3,4-tetrahydroisoquinolin-3-yl)­methyl]-4-(ethylsulfanyl)­benzamide **29,** as an orange solid (67.2 mg, 63%). ^1^H NMR (400 MHz, CDCl_3_) δ: 7.80 (dt, 2H), 7.30 (dd, 2H), 7.08–6.93 (m, 3H), 4.04 (t, 1H), 3.95–3.74 (m, 2H), 3.59 (ddd, 1H), 3.23 (s, 1H), 3.05–2.89 (m, 4H), 2.89–2.79 (m, 1H), 2.58 (d, 4H), 2.30 (s, 3H), 1.34 (t, 3H); ^13^C NMR (150 MHz, Acetone-*d*_6_) δ: 167.04, 142.29, 136.34, 134.45, 132.71, 132.19, 130.10, 128.67, 127.68, 127.47, 127.08, 59.21, 56.24, 41.35, 41.17, 31.08, 26.76, 21.18, 14.52. LC-MS (ESI) *m*/*z* 355.2 [M + H]^+^; HRMS–ESI (*m*/*z*): [M + H]^+^ calcd for C_21_H_27_N_2_OS, 355.1839; found, 355.1849; HPLC purity = 99% (*t* **_R_** = 13.7 min).

#### 3,4-Dichloro-*N*-[(2,6-dimethyl-1,2,3,4-tetrahydroisoquinolin-3-yl)­methyl]­benzamide (30)

The compound was prepared by general procedure I with (2,6-dimethyl-1,2,3,4-tetrahydroisoquinolin-3-yl)­methanamine **4i** (57.0 mg, 0.300 mmol), 3,4-dichlorobenzoic acid (68.7 mg, 0.360 mmol), 1-(3-(dimethylamino)­propyl)-3-ethylcarbodiimide hydrochloride (74.7 mg, 0.390 mmol), 1-hydroxybenzotriazole hydrate (52.7 mg, 0.390 mmol), and *N*,*N*-diisopropylethylamine (160 μL, 0.918 mmol) in dichloromethane (3.0 mL). The crude product was purified by Isco Combi-Flash Companion column chromatography (SiO_2_, 0–6% methanol in dichloromethane) to give the desired product, 3,4-dichloro-*N*-[(2,6-dimethyl-1,2,3,4-tetrahydroisoquinolin-3-yl)­methyl]­benzamide **30,** as an orange solid (57.1 mg, 52%). ^1^H NMR (400 MHz, CDCl_3_) δ: 7.99 (s, 1H), 7.77 (d, 1H), 7.54–7.47 (m, 1H), 7.11–6.95 (m, 3H), 4.10 (d, 1H), 3.97 (d, 1H), 3.86 (d, 1H), 3.69–3.54 (m, 1H), 3.33 (s, 1H), 3.07–2.95 (m, 1H), 2.91–2.76 (m, 1H), 2.67–2.57 (m, 3H), 2.30 (s, 3H); ^13^C NMR (150 MHz, Acetone-*d*_6_) δ: 165.45, 136.36, 136.27, 135.52, 134.41, 132.91, 132.41, 131.53, 130.29, 130.09, 128.12, 127.47, 127.05, 58.99, 56.16, 41.58, 41.29, 31.12, 21.20. LC-MS (ESI) *m*/*z* 363.1 [M + H]^+^; HRMS–ESI (*m*/*z*): [M + H]^+^ calcd for C_19_H_21_Cl_2_N_2_O, 363.1025; found, 363.1030; HPLC purity = 97% (*t* **_R_** = 13.9 min).

#### *N*-[(2,6-Dimethyl-1,2,3,4-tetrahydroisoquinolin-3-yl)­methyl]-3-phenylpropanamide (**31**)

The compound was prepared by general procedure I with (2,6-dimethyl-1,2,3,4-tetrahydroisoquinolin-3-yl)­methanamine **4i** (40.0 mg, 0.210 mmol), 3-phenylpropionic acid (30.0 mg, 0.200 mmol), 1-(3-(dimethylamino)­propyl)-3-ethylcarbodiimide hydrochloride (60.4 mg, 0.315 mmol), 1-hydroxybenzotriazole hydrate (42.6 mg, 0.315 mmol), and *N*,*N*-diisopropylethylamine (91.5 μL, 0.526 mmol) in dichloromethane (2.10 mL). The crude product was purified by flash column chromatography (SiO_2_, 2% methanol in dichloromethane) to give the desired product, *N*-[(2,6-dimethyl-1,2,3,4-tetrahydroisoquinolin-3-yl)­methyl]-3-phenylpropanamide **31,** as a yellow solid (47.7 mg, 74%). ^1^H NMR (400 MHz, CDCl_3_) δ: 7.30–7.24 (m, overlapped with CDCl_3_, 2H), 7.22–7.15 (m, 3H), 6.97–6.86 (m, 3H), 6.05 (br s, 1H), 3.76 (d, 1H), 3.62 (d, 1H), 3.42 (dt, 1H), 3.30 (ddd, 1H), 2.97 (t, 2H), 2.81–2.73 (m, 1H), 2.68–2.56 (m, 2H), 2.50 (t, 2H), 2.30 (s, 3H); ^13^C NMR (150 MHz, Acetone-*d*_6_) δ: 172.44, 142.65, 136.19, 134.59, 132.34, 130.05, 129.34, 129.24, 127.38, 126.97, 126.83, 59.23, 56.78, 41.19, 40.90, 38.55, 32.48, 31.19, 21.20. LC-MS (ESI) *m*/*z* 323.2 [M + H]^+^; HRMS–ESI (*m*/*z*): [M + H]^+^ calcd for C_21_H_27_N_2_O, 323.2118; found, 323.2128; UPLC purity = 100% (*t*_R_ = 2.32 min).

#### *N*-[(2,6-Dimethyl −1,2,3,4-tetrahydroisoquinolin-3-yl)­methyl]-3-(3-fluorophenyl)­propanamide (**32**)

The compound was prepared by general procedure I with (2,6-dimethyl-1,2,3,4-tetrahydroisoquinolin-3-yl)­methanamine **4i** (45.0 mg, 0.236 mmol), 3-(3-fluorophenyl)­propionic acid (38.0 mg, 0.225 mmol), 1-(3-(dimethylamino)­propyl)-3-ethylcarbodiimide hydrochloride (68.0 mg, 0.355 mmol), 1-hydroxybenzotriazole hydrate (48.0 mg, 0.355 mmol), and *N*,*N*-diisopropylethylamine (144 μL, 0.828 mmol) in dichloromethane (4.7 mL). The crude product was purified by flash column chromatography (SiO_2_, 2% methanol in dichloromethane) to give *N*-[(2,6-dimethyl-1,2,3,4-tetrahydroisoquinolin-3-yl)­methyl]-3-(3-fluorophenyl)­propanamide **32** as a yellow syrup (39.0 mg, 51%). ^1^H NMR (600 MHz, CDCl_3_) δ: 7.21 (td, 1H), 7.00–6.94 (m, 2H), 6.93–6.84 (m, 4H), 6.18 (br s, 1H), 3.79 (d, 1H), 3.67 (d, 1H), 3.45 (dt, 1H), 3.31 (ddd, 1H), 2.96 (t, 2H), 2.86–2.81 (m, 1H), 2.70 (dd, 1H), 2.62 (dd, 1H), 2.50 (t, 2H), 2.33 (s, 3H), 2.29 (s, 3H); ^13^C NMR (150 MHz, Acetone-*d*_6_) δ: 171.26, 162.82 (d, *J*_C–F_ = 241.8 Hz), 144.61 (d, *J*_C–F_ = 7.4 Hz), 135.24, 133.53, 131.26, 129.94 (d, *J*_C–F_ = 8.4 Hz), 129.04, 126.42, 125.99, 124.39 (d, *J*_C–F_ = 2.4 Hz), 115.05 (d, *J*_C–F_ = 21.0 Hz), 112.53 (d, *J*_C–F_ = 21.0 Hz), 58.24, 55.76, 40.14, 39.91, 37.06, 31.06, 30.14, 20.21. LC-MS (ESI) *m*/*z* 341.2 [M + H]^+^; HRMS–ESI (*m*/*z*): [M + H]^+^ calcd for C_21_H_25_FN_2_O, 341.2029; found, 341.2024; HPLC purity = 96% (*t*_R_ = 12.12 min).

#### 4-Ethyl-*N*-[(6-fluoro-2-methyl-1,2,3,4-tetrahydroisoquinolin-3-yl)­methyl]­benzamide (**33**)

The compound was prepared by general procedure I with 1-(6-fluoro-2-methyl-1,2,3,4-tetrahydroisoquinolin-3-yl)­methanamine **5i** (60.0 mg, 0.309 mmol), 4-ethylbenzoic acid (51.0 mg, 0.340 mmol), 1-(3-(dimethylamino)­propyl)-3-ethylcarbodiimide hydrochloride (77.0 mg, 0.402 mmol), 1-hydroxybenzotriazole hydrate (45.9 mg, 0.340 mmol), and *N*,*N*-diisopropylethylamine (80.7 μL, 0.463 mmol) in dichloromethane (4.6 mL). The crude product was purified by flash column chromatography (SiO_2_, 2% methanol in dichloromethane) to give the desired product, 4-ethyl-*N*-[(6-fluoro-2-methyl-1,2,3,4-tetrahydroisoquinolin-3-yl)­methyl]­benzamide **33,** as a yellow liquid (54.0 mg, 54%). ^1^H NMR (300 MHz, CDCl_3_) δ: 7.69 (d, 2H), 7.23 (d, 2H), 6.99 (dd, 1H), 6.87–6.76 (m, 3H), 3.85 (d, 1H), 3.70–3.60 (m, 2H), 3.56–3.53 (m, 1H), 2.96–2.94 (m, 1H), 2.82–2.79 (m, 2H), 2.67 (q, 2H), 2.43 (s, 3H), 1.23 (t, 3H); ^13^C NMR (150 MHz, Acetone-*d*_6_) δ: 167.52, 162.22 (d, *J*_C–F_ = 240.3 Hz), 148.55, 137.33 (d, *J*_C–F_ = 7.5 Hz), 133.60, 131.58 (d, *J*_C–F_ = 2.4 Hz), 128.87 (d, *J*_C–F_ = 8.1 Hz), 128.69, 128.22, 115.74 (d, *J*_C–F_ = 20.7 Hz), 113.52 (d, *J*_C–F_ = 21.4 Hz), 58.71, 55.91, 41.26, 41.23, 31.35, 29.37, 15.96. LC-MS (ESI) *m*/*z* 327.2 [M + H]^+^; HRMS–ESI (*m*/*z*): [M + H]^+^ calcd for C_20_H_24_FN_2_O, 327.1867; found, 327.1870; HPLC purity = 98% (*t*_R_ = 12.6 min).

#### *N*-[(6-Fluoro-2-mfthyl-1,2,3,4-tetrahydroisoquinolin-3-yl)­methyl]-4-(trifluoromethyl)­benzamide (**34**)

The compound was prepared by general procedure I with 1-(6-fluoro-2-methyl-1,2,3,4-tetrahydroisoquinolin-3-yl)­methanamine **5i** (60.0 mg, 0.309 mmol), 4-(trifluoromethyl)­benzoic acid (64.6 mg, 0.340 mmol), 1-(3-(dimethylamino)­propyl)-3-ethylcarbodiimide hydrochloride (77.0 mg, 0.402 mmol), 1-hydroxybenzotriazole hydrate (45.9 mg, 0.340 mmol), and *N*,*N*-diisopropylethylamine (80.7 μL, 0.463 mmol) in dichloromethane (4.6 mL). The crude product was purified by flash column chromatography (SiO_2_, 2% methanol in dichloromethane) to give the desired product, *N*-[(6-fluoro-2-methyl-1,2,3,4-tetrahydroisoquinolin-3-yl)­methyl]-4-(trifluoromethyl)­benzamide **34,** as a yellow liquid (51.1 mg, 45%). ^1^H NMR (300 MHz, CDCl_3_) δ: 7.89 (d, 2H), 7.68 (d, 2H), 7.05–7.00 (m, 2H), 6.90–6.80 (m, 2H), 3.90 (d, 1H), 3.77–3.68 (m, 2H), 3.53 (ddd, 1H), 3.09–3.04 (m, 1H), 2.88–2.78 (m, 2H), 2.46 (s, 3H); ^13^C NMR (150 MHz, Acetone-*d*_6_) δ: 166.52, 162.19 (d, *J*_C–F_ = 240.3 Hz), 139.72, 137.17 (d, *J*_C–F_ = 7.5 Hz), 132.99 (q, *J*_C–F_ = 32.0 Hz), 131.50 (d, *J*_C–F_ = 2.6 Hz), 128.93, 128.85 (d, *J*_C–F_ = 8.1 Hz), 126.24 (q, *J*_C–F_ = 3.6 Hz), 125.12 (q, *J*_C–F_ = 270.0 Hz), 115.71 (d, *J*_C–F_ = 20.7 Hz), 113.54 (d, *J*_C–F_ = 21.4 Hz), 58.54, 55.77, 41.43, 41.21, 31.24. LC-MS (ESI) *m*/*z* 367.1 [M + H]^+^; HRMS–ESI (*m*/*z*): [M + H]^+^ calcd for C_19_H_19_F_4_N_2_O, 367.1428; found, 367.1432; HPLC purity = 97% (*t*_R_ = 14.9 min).

#### 3,4-Dichloro-*N*-[(6-fluoro-2-methyl-1,2,3,4-tetrahydroisoquinolin-3-yl)­methyl]­benzamide (**35**)

The compound was prepared by general procedure I with 1-(6-fluoro-2-methyl-1,2,3,4-tetrahydroisoquinolin-3-yl)­methanamine **5i** (60.0 mg, 0.309 mmol), 3,4-dichlorobenzoic acid (64.9 mg, 0.340 mmol), 1-(3-(dimethylamino)­propyl)-3-ethylcarbodiimide hydrochloride (77.0 mg, 0.402 mmol), 1-hydroxybenzotriazole hydrate (45.9 mg, 0.340 mmol), and *N*,*N*-diisopropylethylamine (80.7 μL, 0.463 mmol) in dichloromethane (4.6 mL). The crude product was purified by flash column chromatography (SiO_2_, 2% methanol in dichloromethane) to give the desired product, 3,4-dichloro-*N*-[(6-fluoro-2-methyl-1,2,3,4-tetrahydroisoquinolin-3-yl)­methyl]­benzamide **35,** as a yellow liquid (59.3 mg, 52%). ^1^H NMR (300 MHz, CDCl_3_) δ: 7.83 (s, 1H), 7.58 (d, 1H), 7.48 (d, 1H), 7.02 (dd, 1H), 6.90–6.79 (m, 3H), 3.88 (d, 1H), 3.75–3.63 (m, 2H), 3.49 (ddd, 1H), 3.03–3.00 (m, 1H), 2.85–2.78 (m, 2H), 2.44 (s, 3H); ^13^C NMR (150 MHz, Acetone-*d*_6_) δ: 165.47, 162.14 (d, *J*_C–F_ = 240.3 Hz), 137.11 (d, *J*_C–F_ = 7.5 Hz), 136.25, 135.51, 132.87, 131.49, 131.43 (d, *J*_C–F_ = 2.6 Hz), 130.23, 128.80 (d, *J*_C–F_ = 8.1 Hz), 128.06, 115.66 (d, *J*_C–F_ = 20.7 Hz), 113.51 (d, *J*_C–F_ = 21.4 Hz), 58.50, 55.70, 41.41, 41.18, 31.19. LC-MS (ESI) *m*/*z* 367.1 [M + H]^+^; HRMS–ESI (*m*/*z*): [M + H]^+^ calcd for C_18_H_18_Cl_2_FN_2_O, 367.0775; found, 367.0780; HPLCpurity = 98% (*t*_R_ = 14.9 min).

#### *N*-[(6-Fluoro-2-methyl-1,2,3,4-tetrahydroisoquinolin-3-yl)­methyl]-3-(3-fluorophenyl)­propanamide (**36**)

The compound was prepared by general procedure I with 1-(6-fluoro-2-methyl-1,2,3,4-tetrahydroisoquinolin-3-yl)­methanamine **5i** (26.9 mg, 0.139 mmol), 3-(3-fluorophenyl)­propionic acid (21.0 mg, 0.125 mmol), 1-(3-(dimethylamino)­propyl)-3-ethylcarbodiimide hydrochloride (53.1 mg, 0.277 mmol), 1-hydroxybenzotriazole hydrate (37.4 mg, 0.277 mmol), and *N*,*N*-diisopropylethylamine (84.4 μL, 0.485 mmol) in dichloromethane (2.0 mL). The crude product was purified by flash column chromatography (SiO_2_, 2% methanol in dichloromethane) to give the desired product, *N*-[(6-fluoro-2-methyl-1,2,3,4-tetrahydroisoquinolin-3-yl)­methyl]-3-(3-fluorophenyl)­propanamide **36,** as a yellow liquid (18.9 mg, 44%). ^1^H NMR (300 MHz, CDCl_3_) δ: 7.22 (dd, 1H), 7.01–6.76 (m, 6H), 6.13 (br s, 1H), 3.79 (d, 1H), 3.64 (d, 1H), 3.47–3.41 (m, 1H), 3.37–3.30 (m, 1H), 2.97 (t, 2H), 2.85–2.43 (m, overlapped with t at 2.50, 5H), 2.33 (s, 3H); ^13^C NMR (150 MHz, DMSO-*d*_6_) δ: 171.22, 162.16 (d, *J*_C–F_ = 241.2 Hz), 160.50 (d, *J*_C–F_ = 239.7 Hz), 144.27 (d, *J*_C–F_ = 7.4 Hz), 136.01 (d, *J*_C–F_ = 7.4 Hz), 130.30, 130.00 (d, *J*_C–F_ = 8.4 Hz), 127.84 (d, *J*_C–F_ = 8.1 Hz), 124.44 (d, *J*_C–F_ = 2.1 Hz), 114.94 (d, *J*_C–F_ = 20.6 Hz), 114.59 (d, *J*_C–F_ = 20.4 Hz), 112.60 (d, *J*_C–F_ = 20.7 Hz), 112.55 (d, *J*_C–F_ = 21.15 Hz), 57.30, 54.76, 40.66, 39.18, 36.42, 30.67, 30.24. LC-MS (ESI) *m*/*z* 345.1 [M + H]^+^; HRMS–ESI (*m*/*z*): [M + H]^+^ calcd for C_20_H_23_F_2_N_2_O, 345.1773; found, 345.1778; UPLC purity = 99% (*t*_R_ = 2.28 min).

#### *N*-[(6-Chloro-2-methyl-1,2,3,4-tetrahydroisoquinolin-3-yl)­methyl]-4-ethylbenzamide (**37**)

The compound was prepared by general procedure I with (6-chloro-2-methyl-1,2,3,4-tetrahydroisoquinolin-3-yl)­methanamine **6i** (47.0 mg, 0.223 mmol), 4-ethylbenzoic acid (33.5 mg, 0.223 mmol), 1-(3-(dimethylamino)­propyl)-3-ethylcarbodiimide hydrochloride (85.5 mg, 0.446 mmol), 1-hydroxybenzotriazole hydrate (60.0 mg, 0.446 mmol), and *N*,*N*-diisopropylethylamine (136 μL, 0.780 mmol) in dichloromethane (4.5 mL). The crude product was purified by flash column chromatography (SiO_2_, 2% methanol in dichloromethane) to give the desired product, *N*-[(6-chloro-2-methyl-1,2,3,4-tetrahydroisoquinolin-3-yl)­methyl]-4-ethylbenzamide **37,** as a yellow syrup (37.0 mg, 49%). ^1^H NMR (300 MHz, DMSO-*d*_6_) δ: 8.33 (t, 1H), 7.74 (d, 2H), 7.27 (d, 2H), 7.19–7.12 (m, 2H), 7.11–7.04 (m, 1H), 3.74 (d, 1H), 3.61–3.45 (m, 2H), 3.20–3.08 (m, 1H), 2.91 (quin, 1H), 2.81 (dd, 1H), 2.71–2.57 (m, 3H), 2.39 (s, 3H), 1.17 (t, 3H); ^13^C NMR (150 MHz, Acetone-*d*_6_) δ: 167.53, 148.50, 137.23, 134.51, 133.52, 131.99, 129.34, 128.86, 128.64, 128.20, 126.61, 58.59, 55.73, 41.23, 41.05, 31.01, 29.32, 15.91. LC-MS (ESI) *m*/*z* 343.1 [M + H]^+^; HRMS–ESI (*m*/*z*): [M + H]^+^ calcd for C_20_H_24_ClN_2_O, 343.1572; found, 343.1582; HPLC purity = 95% (*t*_R_ = 15.8 min).

#### *N*-[(6-Chloro-2-methyl-1,2,3,4-tetrahydroisoquinolin-3-yl)­methyl]-4-(ethylsulfanyl)­benzamide (**38**)

The compound was prepared by general procedure I with (6-chloro-2-methyl-1,2,3,4-tetrahydroisoquinolin-3-yl)­methanamine **6i** (47.0 mg, 0.223 mmol), 4-(ethylthio)­benzoic acid (41.0 mg, 0.223 mmol), 1-(3-(dimethylamino)­propyl)-3-ethylcarbodiimide hydrochloride (85.5 mg, 0.446 mmol), 1-hydroxybenzotriazole hydrate (60.0 mg, 0.446 mmol), and *N*,*N*-diisopropylethylamine (136 μL, 0.780 mmol) in dichloromethane (4.5 mL). The crude product was purified by flash column chromatography (SiO_2_, 2% methanol in dichloromethane) to give the desired product, *N*-[(6-chloro-2-methyl-1,2,3,4-tetrahydroisoquinolin-3-yl)­methyl]-4-(ethylsulfanyl)­benzamide **38,** as a white solid (45.0 mg, 54%). ^1^H NMR (300 MHz, DMSO-*d*_6_) δ: 8.38 (t, 1H), 7.75 (d, 2H), 7.33 (d, 2H), 7.18–7.11 (m, 2H), 7.11–7.03 (m, 1H), 3.74 (d, 1H), 3.61–3.44 (m, 2H), 3.14 (dt, 1H), 3.03 (q, 2H), 2.91 (quin, 1H), 2.81 (dd, 1H), 2.65 (dd, 1H), 2.39 (s, 3H), 1.24 (t, 3H); ^13^C NMR (150 MHz, Acetone-*d*_6_) δ: 167.09, 142.37, 137.27, 134.58, 132.75, 132.06, 129.40, 128.93, 128.68, 127.73, 126.67, 58.64, 55.75, 41.30, 41.11, 31.04, 26.81, 14.55. LC-MS (ESI) *m*/*z* 375.0 [M + H]^+^; HRMS–ESI (*m*/*z*): [M + H]^+^ calcd for C_20_H_24_ClN_2_OS, 375.1292; found, 375.1303; HPLC purity = 97% (*t*_R_ = 16.1 min).

#### *N*-[(6-Chloro-2-methyl-1,2,3,4-tetrahydroisoquinolin-3-yl)­methyl]-4-(trifluoromethyl)­benzamide (**39**)

The compound was prepared by general procedure I with (6-chloro-2-methyl-1,2,3,4-tetrahydroisoquinolin-3-yl)­methanamine **6i** (47.0 mg, 0.223 mmol), 4-(trifluoromethyl)­benzoic acid (42.0 mg, 0.223 mmol), 1-(3-(dimethylamino)­propyl)-3-ethylcarbodiimide hydrochloride (85.5 mg, 0.446 mmol), 1-hydroxybenzotriazole hydrate (60.0 mg, 0.446 mmol), and *N*,*N*-diisopropylethylamine (136 μL, 0.780 mmol) in dichloromethane (4.5 mL). The crude product was purified by flash column chromatography (SiO_2_, 2% methanol in dichloromethane) to give the desired product, *N*-[(6-chloro-2-methyl-1,2,3,4-tetrahydroisoquinolin-3-yl)­methyl]-4-(trifluoromethyl)­benzamide **39,** as a white solid (40.0 mg, 47%). ^1^H NMR (300 MHz, DMSO-*d*_6_) δ: 8.68 (t, 1H), 8.02 (d, 2H), 7.85 (d, 2H), 7.22–7.14 (m, 2H), 7.14–7.06 (m, 1H), 3.77 (d, 1H), 3.64–3.50 (m, 2H), 3.26–3.13 (m, 1H), 2.95 (quin, 1H), 2.85 (dd, 1H), 2.69 (dd, 1H), 2.42 (s, 3H); ^13^C NMR (150 MHz, Acetone-*d*_6_) δ: 166.50, 139.78, 137.20, 134.58, 133.03 (q, *J*_C–F_ = 32.0 Hz), 132.08, 129.39, 128.97, 128.94, 126.71, 126.29 (q, *J*_C–F_ = 4.0 Hz), 125.17 (q, *J*_C–F_ = 270.0 Hz), 58.55, 55.68, 41.29, 30.98. LC-MS (ESI) *m*/*z* 383.0 [M + H]^+^; HRMS–ESI (*m*/*z*): [M + H]^+^ calcd for C_19_H_19_ClF_3_N_2_O, 383.1133; found, 383.1142; HPLC purity = 97% (*t*_R_ = 16.1 min).

#### 3,4-Dichloro-*N*-[(6-chloro-2-methyl-1,2,3,4-tetrahydroisoquinolin-3-yl)­methyl]­benzamide (**40**)

The compound was prepared by general procedure I with (6-chloro-2-methyl-1,2,3,4-tetrahydroisoquinolin-3-yl)­methanamine **6i** (47.0 mg, 0.223 mmol), 3,4-dichlorobenzoic acid (42.6 mg, 0.223 mmol), 1-(3-(dimethylamino)­propyl)-3-ethylcarbodiimide hydrochloride (85.5 mg, 0.446 mmol), 1-hydroxybenzotriazole hydrate (60.0 mg, 0.446 mmol), and *N*,*N*-diisopropylethylamine (136 μL, 0.780 mmol) in dichloromethane (4.5 mL). The crude product was purified by flash column chromatography (SiO_2_, 2% methanol in dichloromethane) to give the desired product, 3,4-dichloro-*N*-[(6-chloro-2-methyl-1,2,3,4-tetrahydroisoquinolin-3-yl)­methyl]­benzamide **40,** as a white solid (49.0 mg, 58%). ^1^H NMR (300 MHz, DMSO-*d*_6_) δ: 8.63 (t, 1H), 8.05 (d, 1H), 7.81 (dd, 1H), 7.76 (d, 1H), 7.22–7.13 (m, 2H), 7.13–7.05 (m, 1H), 3.75 (d, 1H), 3.63–3.48 (m, 2H), 3.23–3.11 (m, 1H), 2.94 (quin, 1H), 2.84 (dd, 1H), 2.67 (dd, 1H), 2.41 (s, 3H); ^13^C NMR (150 MHz, Acetone-*d*_6_) δ: 165.48, 137.18, 136.36, 135.57, 134.57, 132.94, 132.07, 131.57, 130.29, 129.36, 128.91, 128.13, 126.69, 58.52, 55.63, 41.31, 30.99. LC-MS (ESI) *m*/*z* 384.9 [M + H]^+^; HRMS–ESI (*m*/*z*): [M + H]^+^ calcd for C_18_H_18_Cl_3_N_2_O, 383.0479; found, 383.0481; HPLC purity = 99% (*t*_R_ = 16.3 min).

#### *N*-[(6-Chloro-2-methyl-1,2,3,4-tetrahydroisoquinolin-3-yl)­methyl]-3-(3-fluorophenyl)­propanamide (**41**)

The compound was prepared by general procedure I with (6-chloro-2-methyl-1,2,3,4-tetrahydroisoquinolin-3-yl)­methatamine **6i** (72.0 mg, 0.342 mmol), 3-(3-fluorophenyl)­propionic acid (16.3 mg, 0.092 mmol), 1-(3-(dimethylamino)­propyl)-3-ethylcarbodiimide hydrochloride (0.131 g, 0.683 mmol), 1-hydroxybenzotriazole hydrate (92.3 mg, 0.683 mmol), and *N*,*N*-diisopropylethylamine (0.208 mL, 1.20 mmol) in dichloromethane (5.00 mL). The crude product was purified by flash column chromatography (SiO_2_, 2% methanol in dichloromethane) to give the desired product, *N*-[(6-chloro-2-methyl-1,2,3,4-tetrahydroisoquinolin-3-yl)­methyl]-3-(3-fluorophenyl)­propanamide **41,** as a brown solid (25.5 mg, 21%). ^1^H NMR (300 MHz, CDCl_3_) δ: 7.27–7.15 (m, overlapped with CDCl_3_, 1H), 7.11 (d, 1H), 7.05 (s, 1H), 7.00–6.82 (m, 4H), 6.04 (br s, 1H), 3.77 (d, 1H), 3.61 (d, 1H), 3.42 (dt, 1H), 3.31 (dt, 1H), 2.96 (t, 2H), 2.84–2.70 (m, 1H), 2.69–2.41 (m, 4H), 2.31 (s, 3H); ^13^C NMR (150 MHz, Acetone-*d*_6_) δ: 172.19, 163.83 (d, *J*_C–F_ = 241.7 Hz), 145.62 (d, *J*_C–F_ = 7.4 Hz), 137.33, 134.52, 132.01, 130.95 (d, *J*_C–F_ = 8.4 Hz), 129.26, 128.84, 126.62, 125.42 (d, *J*_C–F_ = 2.4 Hz), 116.07 (d, *J*_C–F_ = 21.0 Hz), 113.54 (d, *J*_C–F_ = 21.0 Hz), 58.77, 56.26, 41.21, 40.65, 38.05, 32.04, 31.03. LC-MS (ESI) *m*/*z* 361.1 [M + H]^+^; HRMS–ESI (*m*/*z*): [M + H]^+^ calcd for C_20_H_23_ClFN_2_O, 361.1477; found, 361.1485; HPLC purity = 94% (*t*_R_ = 12.9 min).

#### *N*-[(2,7-Dimethyl-1,2,3,4-tetrahydroisoquinolin-3-yl)­methyl]-4-ethylbenzamide (**42**)

The compound was prepared by general procedure I with (2,7-dimethyl-1,2,3,4-tetrahydroisoquinolin-3-yl)­methanamine **7f** (50.0 mg, 0.263 mmol), 4-ethylbenzoic acid (39.5 mg, 0.263 mmol), 1-(3-(dimethylamino)­propyl)-3-ethylcarbodiimide hydrochloride (65.3 mg, 0.346 mmol), and *N*,*N*-diisopropylethylamine (70.0 μL, 0.394 mmol) in dichloromethane (5.0 mL). The crude product was purified by Isco Combi-Flash Companion column chromatography (SiO_2_, 0–5% methanol in dichloromethane) to give *N*-[(2,7-dimethyl-1,2,3,4-tetrahydroisoquinolin-3-yl)­methyl]-4-ethylbenzamide **42** (37.7 mg, 44%). ^1^H NMR (400 MHz, CDCl_3_) δ: 7.68 (d, 2H), 7.24 (d, 2H), 7.02–6.93 (m, 2H), 6.86 (s, 1H), 6.77 (br s, 1H), 3.84 (d, 1H), 3.77–3.62 (m, 2H), 3.50 (ddd, 1H), 3.02–2.90 (m, 1H), 2.83–2.75 (m, 2H), 2.68 (q, 2H), 2.42 (s, 3H), 2.30 (s, 3H), 1.24 (t, 3H); ^13^C NMR (150 MHz, Acetone*-d*_6_) δ: 166.47, 147.49, 134.81, 134.39, 132.65, 130.65, 128.56, 127.68, 127.22, 126.82, 126.61, 58.13, 55.65, 40.46, 40.18, 29.80, 28.37, 20.18, 14.96. LC-MS (ESI) *m*/*z* 323.1 [M + H]^+^; HRMS–ESI (*m*/*z*): [M + H]^+^ calcd for C_21_H_27_N_2_O, 323.2118; found, 323.2132; HPLC purity = 97% (*t*_R_ = 15.5 min).

#### *N*-[(2,7-Dimethyl-1,2,3,4-tetrahydroisoquinolin-3-yl)­methyl]-4-(ethylsulfanyl)­benzamide (**43**)

The compound was prepared by general procedure I with (2,7-dimethyl-1,2,3,4-tetrahydroisoquinolin-3-yl)­methanamine **7f** (50.0 mg, 0.263 mmol), 4-(ethylsulfanyl)­benzoic acid (47.9 mg, 0.263 mmol), 1-(3-(dimethylamino)­propyl)-3-ethylcarbodiimide hydrochloride (65.3 mg, 0.346 mmol), and *N*,*N*-diisopropylethylamine (70.0 μL, 0.394 mmol) in dichloromethane (5.0 mL). The crude product was purified by Isco Combi-Flash Companion column chromatography (SiO_2_, 0–5% methanol in dichloromethane) to give *N*-[(2,7-dimethyl-1,2,3,4-tetrahydroisoquinolin-3-yl)­methyl]-4-(ethylsulfanyl)­benzamide **43** (33.1 mg, 36%). ^1^H NMR (400 MHz, CDCl_3_) δ: 7.66 (d, 2H), 7.29 (d, 2H), 7.01–6.95 (m, 2H), 6.86 (s, 1H), 6.76 (br s, 1H), 3.84 (d, 1H), 3.75–3.60 (m, 2H), 3.49 (dt, 1H), 3.09–2.89 (m, 3H), 2.86–2.68 (m, 2H), 2.42 (s, 3H), 2.30 (s, 3H), 1.34 (t, 3H); ^13^C NMR (150 MHz, Acetone*-d*_6_) δ: 166.00, 141.28, 134.82, 134.38, 131.80, 130.62, 128.55, 127.65, 126.82, 126.72, 126.61, 58.11, 55.61, 40.47, 40.17, 29.77, 25.78, 20.18, 13.53. LC-MS (ESI) *m*/*z* 355.1 [M + H]^+^; HRMS–ESI (*m*/*z*): [M + H]^+^ calcd for C_21_H_27_N_2_OS, 355.1839; found, 355.1847; HPLC purity = 98% (*t*_R_ = 13.6 min)

#### *N*-[(2,7-Dimethyl-1,2,3,4-tetrahydroisoquinolin-3-yl)­methyl]-4-(trifluoromethyl)­benzamide (**44**)

The compound was prepared by general procedure I with (2,7-dimethyl-1,2,3,4-tetrahydroisoquinolin-3-yl)­methanamine **7f** (60.0 mg, 0.315 mmol), 4-(trifluoromethyl)­benzoic acid (59.9 mg, 0.315 mmol), 1-(3-(dimethylamino)­propyl)-3-ethylcarbodiimide hydrochloride (78.3 mg, 0.410 mmol), and *N*,*N*-diisopropylethylamine (80.0 μL, 0.473 mmol) in dichloromethane (5.0 mL). The crude product was purified by Isco Combi-Flash Companion column chromatography (SiO_2_, 0–5% methanol in dichloromethane) to give *N*-[(2,7-dimethyl-1,2,3,4-tetrahydroisoquinolin-3-yl)­methyl]-4-(trifluoromethyl)­benzamide **44** (40.9 mg, 36%). ^1^H NMR (300 MHz, CDCl_3_) δ: 7.85 (d, 2H), 7.68 (d, 2H), 7.05–6.78 (m, 4H), 3.85 (d, 1H), 3.78–3.60 (m, 2H), 3.58–3.37 (m, 1H), 3.07–2.98 (m, 1H), 2.90–2.62 (m, 2H), 2.42 (s, 3H), 2.30 (s, 3H); ^13^C NMR (150 MHz, Acetone*-d*_6_) δ: 165.42, 138.83, 134.87, 134.37, 132.00 (q, *J*_C–F_ = 31.9 Hz), 130.54, 128.55, 127.95, 126.84, 126.62, 125.26 (q, *J*_C–F_ = 3.9 Hz), 124.23 (q, *J*_C–F_ = 276.55 Hz), 58.01, 55.51, 40.63, 40.18, 29.73, 20.16. LC-MS (ESI) *m*/*z* 363.1 [M + H]^+^; HRMS–ESI (*m*/*z*): [M + H]^+^ calcd for C_20_H_22_F_3_N_2_O, 363.1679; found, 363.1686; HPLC purity = 98% (*t*_R_ = 15.8 min).

#### 3,4-Dichloro-*N*-[(2,7-dimethyl-1,2,3,4-tetrahydroisoquinolin-3-yl)­methyl]­benzamide (**45**)

The compound was prepared by general procedure II with (2,7-dimethyl-1,2,3,4-tetrahydroisoquinolin-3-yl)­methanamine **7f** (50.0 mg, 0.263 mmol), 3,4-dichlorobenzoyl chloride (55.0 mg, 0.263 mmol), and triethylamine (44.0 μL, 0.315 mmol) in dichloromethane (5.0 mL). The crude product was purified by Isco Combi-Flash Companion column chromatography (SiO_2_, 0–5% methanol in dichloromethane) to give 3,4-dichloro-*N*-[(2,7-dimethyl-1,2,3,4-tetrahydroisoquinolin-3-yl)­methyl]­benzamide **45** (67.7 mg, 71%). ^1^H NMR (400 MHz, CDCl_3_) δ: 7.80 (d, 1H), 7.55 (dd, 1H), 7.48 (d, 1H),7.02–6.97 (m, 2H), 6.88 (s, 1H), 6.78 (br s, 1H), 3.84 (d, 1H), 3.73 (d, 1H), 3.66 (dt, 1H), 3.47 (ddd, 1H), 3.06–2.96 (m, 1H), 2.83 (dd, 1H), 2.74 (dd, 1H), 2.42 (s, 3H), 2.30 (s, 3H); ^13^C NMR (150 MHz, Acetone*-d*_6_) δ: 164.41, 135.40, 134.87, 134.51, 134.33, 131.91, 130.53, 129.30, 128.53, 127.14, 126.85, 126.61, 58.01, 55.45, 40.64, 40.21, 29.73, 20.19. LC-MS (ESI) *m*/*z* 363.0 [M + H]^+^; HRMS–ESI (*m*/*z*): [M + H]^+^ calcd for C_19_H_21_Cl_2_N_2_O, 363.1025; found, 363.1027; HPLC purity = 95% (*t*_R_ = 16.2 min).

#### 4-Ethyl-*N*-[(7-fluoro-2-methyl-1,2,3,4-tetrahydroisoquinolin-3-yl)­methyl]­benzamide (**46**)

The compound was prepared by general procedure I with 1-(7-fluoro-2-methyl-1,2,3,4-tetrahydroisoquinolin-3-yl)­methanamine **8i** (80.0 mg, 0.412 mmol), 4-ethylbenzoic acid (68.0 mg, 0.453 mmol), 1-(3-(dimethylamino)­propyl)-3-ethylcarbodiimide hydrochloride (0.103 g, 0.535 mmol), 1-hydroxybenzotriazole hydrate (61.2 mg, 0.453 mmol), and *N*,*N*-diisopropylethylamine (0.108 mL, 0.618 mmol) in dichloromethane (6 mL). The crude product was purified by flash column chromatography (SiO_2_, 2% methanol in dichloromethane) to give the desired product, 4-ethyl-*N*-[(7-fluoro-2-methyl-1,2,3,4-tetrahydroisoquinolin-3-yl)­methyl]­benzamide **46,** as a yellow liquid (40.1 mg, 30%). ^1^H NMR (300 MHz, CDCl_3_) δ: 7.70 (d, 2H), 7.24 (d, 2H), 7.04 (dd, 1H), 6.88–6.82 (m, 2H), 6.75 (dd, 1H), 3.86 (d, 1H), 3.75–3.63 (m, 2H), 3.55–3.46 (m, 1H), 3.03–2.98 (m, 1H), 2.80–2.72 (m, 2H), 2.68 (q, 2H), 2.42 (s, 3H), 1.24 (t, 3H); ^13^C NMR (150 MHz, Acetone*-d*_6_) δ: 166.50, 161.00 (d, *J*_C–F_ = 240.2 Hz), 147.53, 136.87 (d, *J*_C–F_ = 6.8 Hz), 132.61, 130.36 (d, *J*_C–F_ = 8.0 Hz), 129.68, 127.69, 127.22, 112.89 (d, *J*_C–F_ = 21.2 Hz), 112.39 (d, *J*_C–F_ = 21.0 Hz), 57.95, 55.35, 40.36, 39.96, 29.30, 28.36, 14.96. LC-MS (ESI) *m*/*z* 327.1 [M + H]^+^; HRMS–ESI (*m*/*z*): [M + H]^+^ calcd for C_20_H_24_FN_2_O, 327.1867; found, 327.1873; HPLC purity = 96% (*t*_R_ = 14.2 min).

#### 4-(Ethylsulfanyl)-*N*-[(7-fluoro-2-methyl-1,2,3,4-tetrahydroisoquinolin-3-yl)­methyl]­benzamide (**47**)

The compound was prepared by general procedure I with 1-(7-fluoro-2-methyl-1,2,3,4-tetrahydroisoquinolin-3-yl)­methanamine **8i** (50.0 mg, 0.257 mmol), 4-(ethylthio)­benzoic acid (51.6 mg, 0.283 mmol), 1-(3-(dimethylamino)­propyl)-3-ethylcarbodiimide hydrochloride (64.1 mg, 0.335 mmol), 1-hydroxybenzotriazole hydrate (38.3 mg, 0.283 mmol), and *N*,*N*-diisopropylethylamine (67.3 μL, 0.386 mmol) in dichloromethane (3.8 mL). The crude product was purified by flash column chromatography (SiO_2_, 2% methanol in dichloromethane) to give the desired product, 4-(ethylsulfanyl)-*N*-[(7-fluoro-2-methyl-1,2,3,4-tetrahydroisoquinolin-3-yl)­methyl]­benzamide **47,** as a yellow liquid (41.9 mg, 45%). ^1^H NMR (300 MHz, CDCl_3_) δ: 7.68 (d, 2H), 7.29 (d, 2H), 7.03 (dd, 1H), 6.88–6.81 (m, 2H), 6.75 (dd, 1H), 3.85 (d, 1H), 3.75–3.61 (m, 2H), 3.49 (ddd, 1H), 3.02–2.94 (m, 3H), 2.79–2.74 (m, 2H), 2.42 (s, 3H), 1.34 (t, 3H); ^13^C NMR (150 MHz, Acetone*-d*_6_) δ: 166.06, 160.98 (d, *J*_C–F_ = 240.3 Hz), 141.36, 136.85 (d, *J*_C–F_ = 7.1 Hz), 131.71, 130.37 (d, *J*_C–F_ = 8.0 Hz), 129.65 (d, *J*_C–F_ = 3.0 Hz), 127.67, 126.68, 112.90 (d, *J*_C–F_ = 21.5 Hz), 112.40 (d, *J*_C–F_ = 21.0 Hz), 57.92, 55.33, 40.38, 39.96, 29.28, 25.75, 13.53. LC-MS (ESI) *m*/*z* 359.1 [M + H]^+^; HRMS–ESI (*m*/*z*): [M + H]^+^ calcd for C_20_H_24_FN_2_OS, 359.1588; found, 359.1594; HPLC purity = 97% (*t*_R_ = 13.0 min).

#### *N*-[(7-Fluoro-2-methyl-1,2,3,4-tetrahydroisoquinolin-3-yl)­methyl]-4-(trifluoromethyl)­benzamide (**48**)

To a solution of 1-(7-fluoro-2-methyl-1,2,3,4-tetrahydroisoquinolin-3-yl)­methanamine **8i** (70.0 mg, 0.360 mmol), triethylamine (75.3 μL, 0.541 mmol), and propylphosphonic anhydride solution (50 wt % in ethyl acetate) (0.344 mL, 0.541 mmol) in dichloromethane (3.1 mL) was added 4-(trifluoromethyl)­benzoic acid (75.4 mg, 0.396 mmol), and the reaction mixture was stirred at room temperature for 16 h. After the reaction was complete, the crude mixture was diluted with dichloromethane, extracted with water, and the organic layer was concentrated under reduced pressure. The crude product was purified by flash column chromatography (SiO_2_, 2% methanol in dichloromethane) to give the desired product, *N*-[(7-fluoro-2-methyl-1,2,3,4-tetrahydroisoquinolin-3-yl)­methyl]-4-(trifluoromethyl)­benzamide **48,** as a yellow liquid (64.1 mg, 49%). ^1^H NMR (300 MHz, CDCl_3_) δ: 7.87 (d, 2H), 7.67 (d, 2H), 7.05 (dd, 1H), 6.99 (br s, 1H)), 6.86 (td, 1H), 6.76 (dd, 1H), 3.86 (d, 1H), 3.79–3.64 (m, 2H), 3.49 (ddd, 1H), 3.09–2.99 (m, 1H), 2.95–2.65 (m, 2H), 2.42 (s, 3H); ^13^C NMR (150 MHz, Acetone*-d*_6_) δ: 165.46, 161.00 (d, *J*_C–F_ = 240.5 Hz), 138.77, 136.83 (d, *J*_C–F_ = 7.1 Hz), 132.00 (q, *J*_C–F_ = 31.9 Hz), 130.37 (d, *J*_C–F_ = 7.8 Hz), 129.58 (d, *J*_C–F_ = 3.0 Hz), 127.96, 125.29 (q, *J*_C–F_ = 3.9 Hz), 124.16 (q, *J*_C–F_ = 269.95 Hz), 112.93 (d, *J*_C–F_ = 21.2 Hz), 112.42 (d, *J*_C–F_ = 21.0 Hz), 57.83, 55.25, 40.54, 39.94. LC-MS (ESI) *m*/*z* 367.1 [M + H]^+^; HRMS–ESI (*m*/*z*): [M + H]^+^ calcd for C_19_H_19_F_4_N_2_O, 367.1428; found, 367.1428; HPLC purity = 98% (*t*_R_ = 13.0 min).

#### 3,4-Dichloro-*N*-[(7-fluoro-2-methyl-1,2,3,4-tetrahydroisoquinolin-3-yl)­methyl]­benzamide (**49**)

The compound was prepared by general procedure I with 1-(7-fluoro-2-methyl-1,2,3,4-tetrahydroisoquinolin-3-yl)­methanamine **8i** (80.0 mg, 0.412 mmol), 3,4-dichlorobenzoic acid (86.5 mg, 0.453 mmol), 1-(3-(dimethylamino)­propyl)-3-ethylcarbodiimide hydrochloride (0.103 g, 0.535 mmol), 1-hydroxybenzotriazole hydrate (61.2 mg, 0.453 mmol), and *N*,*N*-diisopropylethylamine (0.108 mL, 0.618 mmol) in dichloromethane (6 mL). The crude product was purified by flash column chromatography (SiO_2_, 2% methanol in dichloromethane) to give the desired product, 3,4-dichloro-*N*-[(7-fluoro-2-methyl-1,2,3,4-tetrahydroisoquinolin-3-yl)­methyl]­benzamide **49,** as a yellow liquid (0.128 g, 84%). ^1^H NMR (300 MHz, CDCl_3_) δ: 7.84 (d, 1H), 7.58 (d, 1H), 7.48 (d, 1H), 7.05 (dd, 1H), 6.91–6.82 (m, 2H), 6.77 (dd, 1H), 3.87 (d, 1H), 3.75 (d, 1H), 3.67 (dt, 1H), 3.47 (ddd, 1H), 3.09–2.98 (m, 1H), 2.90–2.68 (m, 2H), 2.43 (s, 3H); ^13^C NMR (150 MHz, Acetone*-d*_6_) δ: 164.41, 161.00 (d, *J*_C–F_ = 240.3 Hz), 136.85 (d, *J*_C–F_ = 7.1 Hz), 135.39, 134.55, 131.93, 130.58, 130.35 (d, *J*_C–F_ = 7.8 Hz), 129.58 (d, *J*_C–F_ = 2.9 Hz), 129.28, 127.12, 112.92 (d, *J*_C–F_ = 21.3 Hz), 112.40 (d, *J*_C–F_ = 21.1 Hz), 57.82, 55.20, 40.56, 39.98. LC-MS (ESI) *m*/*z* 367.0 [M + H]^+^; HRMS–ESI (*m*/*z*): [M + H]^+^ calcd for C_18_H_18_Cl_2_FN_2_O, 367.0775; found, 367.0780; HPLC purity = 96% (*t*_R_ = 13.2 min).

#### *N*-[(7-Chloro-2-methyl-1,2,3,4-tetrahydroisoquinolin-3-yl)­methyl]-4-(trifluoromethyl)­benzamide (**50**)

The compound was prepared by general procedure I with (7-chloro-2-methyl-1,2,3,4-tetrahydroisoquinolin-3-yl)­methanamine **9i** (25.0 mg, 0.119 mmol), 4-(trifluoromethyl)­benzoic acid (22.5 mg, 0.119 mmol), 1-(3-(dimethylamino)­propyl)-3-ethylcarbodiimide hydrochloride (45.6 mg, 0.238 mmol), 1-hydroxybenzotriazole hydrate (32.1 mg, 0.238 mmol), and *N*,*N*-diisopropylethylamine (50.0 μL, 0.298 mmol) in dichloromethane (2.4 mL). The crude product was purified by flash column chromatography (SiO_2_, 3% methanol in dichloromethane) to give the desired product, *N*-[(7-chloro-2-methyl-1,2,3,4-tetrahydroisoquinolin-3-yl)­methyl]-4-(trifluoromethyl)­benzamide **50,** as a yellow syrup (20.0 mg, 44%). ^1^H NMR (300 MHz, DMSO-*d*_6_) δ: 8.68 (t, 1H), 8.03 (d, 2H), 7.85 (d, 2H), 7.21–7.15 (m, 2H), 7.12 (d, 1H), 3.79 (d, 1H), 3.67–3.49 (m, 2H), 3.28–3.14 (m, overlapped with H_2_O, 1H), 2.95 (quin, 1H), 2.82 (dd, 1H), 2.65 (dd, 1H), 2.40 (s, 3H); ^13^C NMR (150 MHz, Acetone-*d*_6_) δ: 166.46, 139.76, 137.86, 133.69, 133.01 (d, *J*_C–F_ = 32.0 Hz), 131.73, 131.41, 128.95, 127.07, 127.01, 126.27 (q, *J*_C–F_ = 4.0 Hz), 125.15 (q, *J*_C–F_ = 270.1 Hz), 58.71, 55.93, 41.51, 40.93, 30.29. LC-MS (ESI) *m*/*z* 383.0 [M + H]^+^; HRMS–ESI (*m*/*z*): [M + H]^+^ calcd for C_19_H_19_ClF_3_N_2_O, 383.1133; found, 383.1142; HPLC purity = 99% (*t*_R_ = 16.2 min).

#### 3,4-Dichloro-*N*-[(7-chloro-2-methyl-1,2,3,4-tetrahydroisoquinolin-3-yl)­methyl]­benzamide (**51**)

The compound was prepared by general procedure I with (7-chloro-2-methyl-1,2,3,4-tetrahydroisoquinolin-3-yl)­methanamine **9i** (25.0 mg, 0.119 mmol), 3,4-dichlorobenzoic acid (22.7 mg, 0.119 mmol), 1-(3-(dimethylamino)­propyl)-3-ethylcarbodiimide hydrochloride (45.6 mg, 0.238 mmol), 1-hydroxybenzotriazole hydrate (32.1 mg, 0.238 mmol), and *N*,*N*-diisopropylethylamine (50.0 μL, 0.298 mmol) in dichloromethane (2.4 mL). The crude product was purified by flash column chromatography (SiO_2_, 3% methanol in dichloromethane) to give the desired product, 3,4-dichloro-*N*-[(7-chloro-2-methyl-1,2,3,4-tetrahydroisoquinolin-3-yl)­methyl]­benzamide **51,** as a white foam (25.0 mg, 55%). ^1^H NMR (DMSO-*d*_6_, 300 MHz) δ: 8.64 (t, 1H), 8.05 (d, 1H), 7.82 (dd, 1H), 7.75 (d, 1H), 7.22–7.12 (m, 2H), 7.12 (d, 1H), 3.77 (d, 1H), 3.66–3.47 (m, 2H), 3.26–3.19 (m, 1H), 2.94 (quin, 1H), 2.81 (dd, 1H), 2.64 (dd, 1H), 2.39 (s, 3H); ^13^C NMR (150 MHz, Acetone-*d*_6_) δ: 165.45, 137.87, 136.35, 135.56, 133.68, 132.93, 131.72, 131.56, 131.40, 130.29, 128.12, 127.06, 126.99, 58.68, 55.89, 41.54, 40.97, 30.31. LC-MS (ESI) *m*/*z* 383.0 [M + H]^+^; HRMS–ESI (*m*/*z*): [M + H]^+^ calcd for C_18_H_18_Cl_3_N_2_O, 383.0479; found, 383.0474; HPLC purity = 96% (*t*_R_ = 16.3 min).

#### *N*-[(2,6-Dimethyl-1,2,3,4-tetrahydroisoquinolin-3-yl)­methyl]-2,3-dihydro-1*H*-indene-2-carboxamide (**52**)

The compound was prepared by general procedure I with (2,6-dimethyl-1,2,3,4-tetrahydroisoquinolin-3-yl)­methanamine **4i** (34.5 mg, 0.181 mmol), 2-indanecarboxylic acid (28.0 mg, 0.172 mmol), 1-(3-(dimethylamino)­propyl)-3-ethylcarbodiimide hydrochloride (52.0 mg, 0.272 mmol), 1-hydroxybenzotriazole hydrate (36.7 mg, 0.272 mmol), and *N*,*N*-diisopropylethylamine (110 μL, 0.635 mmol) in dichloromethane (3.60 mL). The crude product was purified by flash column chromatography (SiO_2_, 3% methanol in dichloromethane) to give the desired product, *N*-[(2,6-dimethyl-1,2,3,4-tetrahydroisoquinolin-3-yl)­methyl]-2,3-dihydro-1*H*-indene-2-carboxamide **52,** as a colorless syrup (23.0 mg, 40%). ^1^H NMR (600 MHz, CDCl_3_) δ: 7.24–7.20 (m, 2H), 7.19–7.15 (m, 2H), 7.00 (d, 1H), 6.96 (d, 1H), 6.94 (s, 1H), 6.35 (br s, 1H), 3.86 (d, 1H), 3.73 (d, 1H), 3.53 (dt, 1H), 3.43 (ddd, 1H), 3.30–3.14 (m, 5H), 2.96–2.90 (m, 1H), 2.80 (dd, 1H), 2.74 (dd, 1H), 2.42 (s, 3H), 2.32 (s, 3H); ^13^C NMR (150 MHz, Acetone-*d*_6_) δ: 175.28, 143.32, 136.34, 134.59, 132.29, 130.06, 127.47, 127.28, 127.04, 125.10, 59.41, 56.77, 45.98, 41.25, 41.10, 37.65, 37.62, 31.28, 21.22. LC-MS (ESI) *m*/*z* 335.2 [M + H]^+^; HRMS–ESI (*m*/*z*): [M + H]^+^ calcd for C_22_H_27_N_2_O, 335.2118; found, 335.2119; HPLC purity = 97% (*t*_R_ = 12.9 min).

#### *N*-[(2-Methyl-1,2,3,4-tetrahydroisoquinolin-3-yl)­methyl]-2,3-dihydro-1*H* **-**indene-2-carboxamide (**53**)

The compound was prepared by general procedure I with (2-methyl-1,2,3,4-tetrahydroisoquinolin-3-yl)­methanamine **2c** (300 mg, 1.70 mmol), 2-indanecarboxylic acid (248 mg, 1.53 mmol), 1-(3-(dimethylamino)­propyl)-3-ethylcarbodiimide hydrochloride (489 mg, 2.55 mmol), 1-hydroxybenzotriazole hydrate (345 mg, 2.55 mmol), and *N*,*N*-diisopropylethylamine (1.04 mL, 5.96 mmol) in dichloromethane (17 mL). The crude product was purified by flash column chromatography (SiO_2_, 3% methanol in dichloromethane) to give *N*-[(2-methyl-1,2,3,4-tetrahydroisoquinolin-3-yl)­methyl]-2,3-dihydro-1*H*-indene-2-carboxamide **53** as an off-white solid (427 mg, 87%). ^1^H NMR (600 MHz, Acetone-*d*_6_) δ: 7.21–7.14 (m, 3H), 7.13–7.09 (m, 4H), 7.09–7.06 (m, 1H), 7.06–7.02 (m, 1H), 3.82 (d, 1H), 3.59 (d, 1H), 3.52 (ddd, 1H), 3.34–3.27 (m, 2H), 3.20 (ddd, 2H), 3.10 (dd, 2H), 2.84–2.72 (m, 3H), 2.41 (s, 3H); ^13^C NMR (150 MHz, Acetone-*d*_6_) δ: 175.29, 143.30, 135.58, 134.88, 129.58, 127.27, 127.10, 127.05, 126.61, 125.10, 59.30, 57.10, 45.97, 41.34, 41.18, 37.65, 37.63, 31.42. LC-MS (ESI) *m*/*z* 321.2 [M + H]^+^; HRMS–ESI (*m*/*z*): [M + H]^+^ calcd for C_21_H_25_N_2_O, 321.1961; found, 321.1968; HPLC purity = 98% (*t*_R_ = 11.7 min).

#### *N*-[(6-Chloro-2-methyl-1,2,3,4-tetrahydroisoquinolin-3-yl)­methyl]-2,3-dihydro-1*H*-indene-2-carboxamide (**54**)

The compound was prepared by general procedure I with (6-chloro-2-methyl-1,2,3,4-tetrahydroisoquinolin-3-yl)­methanamine **6i** (39.5 mg, 0.187 mmol), 2-indanecarboxylic acid (29.0 mg, 0.178 mmol), 1-(3-(dimethylamino)­propyl)-3-ethylcarbodiimide hydrochloride (54.0 mg, 0.281 mmol), 1-hydroxybenzotriazole hydrate (38.0 mg, 0.281 mmol), and *N*,*N*-diisopropylethylamine (110 μL, 0.656 mmol) in dichloromethane (3.7 mL). The crude product was purified by flash column chromatography (SiO_2_, 3% methanol in dichloromethane) to give *N*-[(6-chloro-2-methyl-1,2,3,4-tetrahydroisoquinolin-3-yl)­methyl]-2,3-dihydro-1*H*-indene-2-carboxamide **54** as a brown syrup (23.0 mg, 37%). ^1^H NMR (600 MHz, DMSO-*d*_6_) δ: 7.93 (br t, 1H), 7.20–7.15 (m, 4H), 7.13–7.10 (m, 2H), 7.10–7.08 (m, 1H), 3.75 (d, 1H), 3.53 (d, 1H), 3.40–3.36 (m, 1H), 3.21 (quin., 1H), 3.08 (dd, 1H), 3.06–3.01 (m, 4H), 2.79 (dd, 1H), 2.74 (ddd, 1H), 2.65 (dd, 1H), 2.36 (s, 3H); ^13^C NMR (150 MHz, Acetone-*d*_6_) δ: 175.16, 143.15, 137.26, 134.48, 131.91, 129.15, 128.74, 127.15, 126.52, 124.97, 58.71, 56.12, 45.85, 41.19, 40.72, 37.50, 31.05. LC-MS (ESI) *m*/*z* 355.1 [M + H]^+^; HRMS–ESI (*m*/*z*): [M + H]^+^ calcd for C_21_H_23_ClN_2_O, 355.1577; found, 355.1572; HPLC purity = 97% (*t*_R_ = 12.86 min).

#### *N*-{[(3*S*)-2-Methyl-1,2,3,4-tetrahydroisoquinolin-3-yl]­methyl}-2,3-dihydro-1*H*-indene-2-carboxamide (**55**)

The compound was prepared by general procedure I with [(3*S*)-2-methyl-1,2,3,4-tetrahydroisoquinolin-3-yl]­methanamine **10c** (755 mg, 4.28 mmol), 2-indanecarboxylic acid (625 mg, 3.86 mmol), 1-(3-(dimethylamino)­propyl)-3-ethylcarbodiimide hydrochloride (1.23 mg, 6.43 mmol), 1-hydroxybenzotriazole hydrate (868 mg, 6.43 mmol), and *N*,*N*-diisopropylethylamine (2.60 mL, 15.0 mmol) in dichloromethane (22 mL). The crude product was purified by flash column chromatography (SiO_2_, 3% methanol in dichloromethane) to give *N*-{[(3*S*)-2-methyl-1,2,3,4-tetrahydroisoquinolin-3-yl]­methyl}-2,3-dihydro-1*H*-indene-2-carboxamide **55** as an off-white solid (1.17 g, 95%). [α]_D_ ^24^ = −42.46̊ (c = 1, MeOH); ^1^H NMR (600 MHz, DMSO-*d*_6_) δ: 7.95 (br t, 1H), 7.20–7.16 (m, 2H), 7.14–7.07 (m, 5H), 7.06–7.03 (m, 1H), 3.76 (d, 1H), 3.55 (d, 1H), 3.40 (dt, 1H), 3.22 (quin., 1H), 3.12–3.06 (m, 1H), 3.06–3.01 (m, 4H), 2.77 (dd, 1H), 2.74–2.69 (m, 1H), 2.66 (dd, 1H), 2.36 (s, 3H); ^13^C NMR (150 MHz, Acetone-*d*_6_) δ: 175.28, 143.30, 135.64, 134.90, 129.58, 127.27, 127.09, 127.03, 126.60, 125.10, 59.29, 57.12, 45.97, 41.36, 41.19, 37.65, 37.63, 31.44; LC-MS (ESI) *m*/*z* 321.2 [M + H]^+^; HRMS–ESI (*m*/*z*): [M + H]^+^ calcd for C_21_H_25_N_2_O, 321.1961; found, 321.1967; HPLC purity = 99% (*t*_R_ = 11.8 min); 99.6% ee [MeOH/DEA = 100/0.1 (v/v); *t*_R1_ = 8.93 min (minor), *t*_R2_ = 9.80 min (major)].

#### *N*-{[(3*R*)-2-Methyl-1,2,3,4-tetrahydroisoquinolin-3-yl]­methyl}-2,3-dihydro-1*H*-indene-2-carboxamide (**56**)

The compound was prepared by general procedure I with [(3*R*)-2-methyl-1,2,3,4-tetrahydroisoquinolin-3-yl]­methanamine **11e** (0.525 g, 2.98 mmol), 2-indanecarboxylic acid (0.483 g, 2.98 mmol), 1-(3-(dimethylamino)­propyl)-3-ethylcarbodiimide hydrochloride (0.857 g, 4.47 mmol), 1-hydroxybenzotriazole hydrate (0.604 g, 4.47 mmol), and *N*,*N*-diisopropylethylamine (1.31 mL, 7.45 mmol) in dichloromethane (29.8 mL). The crude product was purified by flash column (SiO_2_, 3% methanol in dichloromethane) to give the desired product, *N*-{[(3*R*)-2-methyl-1,2,3,4-tetrahydroisoquinolin-3-yl]­methyl}-2,3-dihydro-1*H*-indene-2-carboxamide **56,** as a white solid (0.782 g, 82%). [α]_D_ ^24^ = +57.57̊ (c = 1, MeOH); ^1^H NMR (400 MHz, CDCl_3_) δ: 7.25–7.03 (m, 8H), 6.23 (br s, 1H), 3.86 (d, 1H), 3.73 (d, 1H), 3.52 (dt, 1H), 3.40 (ddd, 1H), 3.28–3.11 (m, 5H), 2.95–2.87 (m, 1H), 2.85–2.71 (m, 2H), 2.40 (s, 3H); ^13^C NMR (150 MHz, Acetone-*d*_6_) δ: 175.28, 143.27, 135.59, 134.86, 129.55, 127.25, 127.06, 127.01, 126.58, 125.07, 59.26, 57.09, 45.94, 41.32, 41.17, 37.62, 37.60, 31.41. LC-MS (ESI) *m*/*z* 321.2 [M + H]^+^; HRMS–ESI (*m*/*z*): [M + H]^+^ calcd for C_21_H_25_N_2_O, 321.1961; found, 321.1962; HPLC purity = 100% (*t*_R_ = 11.9 min); 100% ee [MeOH/DEA = 100/0.1 (v/v); *t*_R_ = 8.88 min].

#### *N*-{[(3*S*)-6-Chloro-2-methyl-1,2,3,4-tetrahydroisoquinolin-3-yl]­methyl}-2,3-dihydro-1*H*-indene-2-carboxamide (**57**)

The compound was prepared by general procedure I with [(3*S*)-6-chloro-2-methyl-1,2,3,4-tetrahydroisoquinolin-3-yl]­methanamine **12i** (35.0 mg, 0.166 mmol), 2-indanecarboxylic acid (26.0 mg, 0.158 mmol), 1-(3-(dimethylamino)­propyl)-3-ethylcarbodiimide hydrochloride (48.0 mg, 0.249 mmol), 1-hydroxybenzotriazole hydrate (34.0 mg, 0.249 mmol), and *N*,*N*-diisopropylethylamine (100 μL, 0.581 mmol) in dichloromethane (3.3 mL). The crude product was purified by flash column chromatography (SiO_2_, 3% methanol in dichloromethane) to give *N*-{[(3*S*)-6-chloro-2-methyl-1,2,3,4-tetrahydroisoquinolin-3-yl]­methyl}-2,3-dihydro-1*H*-indene-2-carboxamide **57** as a beige solid (40.0 mg, 71%). [α]_D_ ^25^ = −19.31̊° (c = 1, CH_3_OH); ^1^H NMR (400 MHz, CDCl_3_) δ: 7.22–7.13 (m, 4H), 7.12 (dd, 1H), 7.08 (d, 1H), 6.96 (d, 1H), 6.19 (br s, 1H), 3.81 (d, 1H), 3.65 (d, 1H), 3.48 (dt, 1H), 3.39 (ddd, 1H), 3.29–3.10 (m, 5H), 2.91–2.83 (m, 1H), 2.77 (dd, 1H), 2.69 (dd, 1H), 2.38 (s, 3H); ^13^C NMR (150 MHz, Acetone-*d*_6_) δ: 175.19, 143.15, 137.25, 134.46, 131.92, 129.15, 128.74, 127.15, 126.52, 124.97, 58.72, 56.12, 45.85, 41.18, 40.73, 37.50, 31.06. LC-MS (ESI) *m*/*z* 355.1 [M + H]^+^; HRMS–ESI (*m*/*z*): [M + H]^+^ calcd for C_21_H_23_ClN_2_O, 355.1577; found, 355.1571; HPLC purity = 97% (*t*_R_ = 13.05 min); 100% ee [MeOH/DEA = 100/0.1 (v/v); *t*_R_ = 17.55 min].

#### *N*-{[(3*R*)-6-Chloro-2-methyl-1,2,3,4-tetrahydroisoquinolin-3-yl]­methyl}-2,3-dihydro-1*H*-indene-2-carboxamide (58)

The compound was prepared by general procedure I with [(3*R*)-6-chloro-2-methyl-1,2,3,4-tetrahydroisoquinolin-3-yl]­methanamine **13i** (50.8 mg, 0.241 mmol), 2-indanecarboxylic acid (39.1 mg, 0.241 mmol), 1-(3-(dimethylamino)­propyl)-3-ethylcarbodiimide hydrochloride (69.3 mg, 0.362 mmol), 1-hydroxybenzotriazole hydrate (48.9 mg, 0.362 mmol), and *N*,*N*-diisopropylethylamine (0.105 mL, 0.603 mmol) in dichloromethane (2.4 mL), stirred at room temperature for 16 h. After workup, the crude was purified by flash column chromatography (SiO_2_, dichloromethane/methanol = 97/3) to give *N*-{[(3*R*)-6-chloro-2-methyl-1,2,3,4-tetrahydroisoquinolin-3-yl]­methyl}-2,3-dihydro-1*H*-indene-2-carboxamide **58** as a white solid (70.5 mg, 82%). [α]_D_ ^25^ = +18.00̊° (c = 1, CH_3_OH); ^1^H NMR (CDCl_3_, 400 MHz) δ: 7.22–7.06 (m, 6H), 6.96 (d, 1H), 6.16 (br s, 1H), 3.80 (d, 1H), 3.64 (d, 1H), 3.47 (dt, 1H), 3.39 (ddd, 1H), 3.27–3.11 (m, 5H), 2.90–2.82 (m, 1H), 2.77 (dd, 1H), 2.69 (dd, 1H), 2.38 (s, 3H); ^13^C NMR (150 MHz, Acetone-*d*_6_) δ: 175.16, 143.15, 137.26, 134.49, 131.91, 129.15, 128.74, 127.15, 126.51, 124.97, 58.71, 56.12, 45.85, 41.20, 40.72, 37.50, 31.05. LC-MS (ESI) *m*/*z* 355.1 [M + H]^+^; HRMS–ESI (*m*/*z*): [M + H]^+^ calcd for C_21_H_23_ClN_2_O, 355.1577; found, 355.1572; HPLC purity = 98% (*t*_R_ = 13.12 min); 95.0% ee [MeOH/DEA = 100/0.1 (v/v); *t*_R1_ = 11.90 min (major), *t*_R2_ = 17.53 min (minor)].

#### *N*-{[(3*S*)-2,6-Dimethyl-1,2,3,4-tetrahydroisoquinolin-3-yl]­methyl}-3-phenylpropanamide (59)

The compound was prepared by general procedure I with [(3*S*)-2,6-dimethyl-1,2,3,4-tetrahydroisoquinolin-3-yl]­methanamine **14i** (133 mg, 0.699 mmol), 3-phenylpropionic acid (100 mg, 0.664 mmol), 1-(3-(dimethylamino)­propyl)-3-ethylcarbodiimide hydrochloride (201 mg, 1.05 mmol), 1-hydroxybenzotriazole hydrate (142 mg, 1.05 mmol), and *N*,*N*-diisopropylethylamine (430 μL, 2.45 mmol) in dichloromethane (2.9 mL). The crude product was purified by flash column chromatography (SiO_2_, 3–4% methanol in dichloromethane) to give the desired product, *N*-{[(3*S*)-2,6-dimethyl-1,2,3,4-tetrahydroisoquinolin-3-yl]­methyl}-3-phenylpropanamide **59,** as a white solid (113 mg, 50%). [α]_D_ ^24^ = −36.60° (c = 1, MeOH); ^1^H NMR (600 MHz, CDCl_3_) δ: 7.29–7.25 (m overlapped with CDCl_3_, 2H), 7.22–7.16 (m, 3H), 6.96 (d, 1H), 6.91 (d, 1H), 6.88 (s, 1H), 6.06 (br s, 1H), 3.77 (d, 1H), 3.64 (d, 1H), 3.43 (dt, 1H), 3.30 (ddd, 1H), 2.97 (t, 2H), 2.83–2.77 (m, 1H), 2.68 (dd, 1H), 2.61 (dd, 1H), 2.53–2.48 (m, 2H), 2.31 (s, 3H), 2.30 (s, 3H); ^13^C NMR (150 MHz, Acetone-*d*_6_) δ: 172.45, 142.67, 136.29, 134.55, 132.19, 130.06, 129.37, 129.25, 127.43, 127.01, 126.85, 59.32, 56.74, 41.14, 40.89, 38.57, 32.49, 31.14, 21.21. LC-MS (ESI) *m*/*z* 323.2 [M + H]^+^; HRMS–ESI (*m*/*z*): [M + H]^+^ calcd for C_21_H_27_N_2_O, 323.2118; found, 323.2126; HPLC purity = 95% (*t*_R_ = 11.9 min)); 97.4% ee [MeOH/DEA = 100/0.1 (v/v); *t*_R1_ = 6.07 min (minor), *t*_R2_ = 6.37 min (major)].

#### *N*-{[(3*R*)-2,6-Dimethyl-1,2,3,4-tetrahydroisoquinolin-3-yl]­methyl}-3-phenylpropanamide (60)

The compound was prepared by general procedure I with [(3*R*)-2,6-dimethyl-1,2,3,4-tetrahydroisoquinolin-3-yl]­methanamine **15i** (50.0 mg, 0.263 mmol), 3-phenylpropionic acid (37.5 mg, 0.250 mmol), 1-(3-(dimethylamino)­propyl)-3-ethylcarbodiimide hydrochloride (75.6 mg, 0.395 mmol), 1-hydroxybenzotriazole hydrate (53.3 mg, 0.395 mmol), and *N*,*N*-diisopropylethylamine (160 μL, 0.921 mmol) in dichloromethane (5.3 mL). The crude product was purified by flash column chromatography (SiO_2_, 3–5% methanol in dichloromethane) to give the desired product, *N*-{[(3*R*)-2,6-dimethyl-1,2,3,4-tetrahydroisoquinolin-3-yl]­methyl}-3-phenylpropanamide **60,** as a white solid (46.0 mg, 58%). [α]_D_ ^24^ = +39.01̊° (c = 1, MeOH); ^1^H NMR (300 MHz, DMSO-*d*_6_) δ: 7.80 (t, 1H), 7.30–7.12 (m, 5H), 6.95–6.81 (m, 3H), 3.68 (d, 1H), 3.48 (d, 1H), 3.31–3.20 (m overlapped with H_2_O, 1H), 3.01 (dt, 1H), 2.81 (t, 2H), 2.69–2.53 (m, 3H), 2.40 (t, 2H), 2.31 (s, 3H), 2.23 (s, 3H); ^13^C NMR (150 MHz, Acetone-*d*_6_) δ: 172.45, 142.66, 136.25, 134.57, 132.25, 130.06, 129.36, 129.25, 127.41, 126.99, 126.84, 59.27, 56.75, 41.14, 40.89, 38.56, 32.49, 31.16, 21.21. LC-MS (ESI) *m*/*z* 323.2 [M + H]^+^; HRMS–ESI (*m*/*z*): [M + H]^+^ calcd for C_21_H_27_N_2_O, 323.2118; found, 323.2113; UPLC purity = 97% (*t*_R_ = 2.37 min); 100% ee [MeOH/DEA = 100/0.1 (v/v); *t*_R1_ = 6.05 min].

#### *N*-{[(3*S*)-2,6-Dimethyl-1,2,3,4-tetrahydroisoquinolin-3-yl]­methyl}-2,3-dihydro-1*H*-indene-2-carboxamide (61)

The compound was prepared by general procedure I with [(3*S*)-2,6-dimethyl-1,2,3,4-tetrahydroisoquinolin-3-yl]­methanamine **14i** (27.5 mg, 0.145 mmol), 2-indanecarboxylic acid (22.3 mg, 0.137 mmol), 1-(3-(dimethylamino)­propyl)-3-ethylcarbodiimide hydrochloride (41.5 mg, 0.217 mmol), 1-hydroxybenzotriazole hydrate (29.0 mg, 0.217 mmol), and *N*,*N*-diisopropylethylamine (88.0 μL, 0.506 mmol) in dichloromethane (2.9 mL). The crude product was purified by flash column chromatography (SiO_2_, 3–4% methanol in dichloromethane) to give the desired product, *N*-{[(3*S*)-2,6-dimethyl-1,2,3,4-tetrahydroisoquinolin-3-yl]­methyl}-2,3-dihydro-1*H*-indene-2-carboxamide **61,** as a white solid (25.0 mg, 55%). [α]_D_ ^24^ = −28.68̊° (c = 1, MeOH); ^1^H NMR (600 MHz, CDCl_3_) δ: 7.22–7.18 (m, 2H), 7.17–7.13 (m, 2H), 6.97 (d, 1H), 6.94 (d, 1H), 6.92 (s, 1H), 6.30 (br s, 1H), 3.84 (d, 1H), 3.71 (d, 1H), 3.52 (dt, 1H), 3.40 (ddd, 1H), 3.28–3.11 (m, 5H), 2.95–2.88 (m, 1H), 2.78 (dd, 1H), 2.72 (dd, 1H), 2.40 (s, 3H), 2.30 (s, 3H); ^13^C NMR (150 MHz, CDCl_3_) δ: 175.32, 141.98, 136.38, 133.01, 130.21, 129.27, 127.17, 126.67, 126.51, 124.43, 57.84, 56.03, 45.91, 40.94, 39.73, 36.96, 36.93, 29.21, 21.18. LC-MS (ESI) *m*/*z* 335.2 [M + H]^+^; HRMS–ESI (*m*/*z*): [M + H]^+^ calcd for C_22_H_27_N_2_O, 335.2118; found, 335.2127; HPLC purity = 98% (*t*_R_ = 12.9 min); 97.9% ee [MeOH/DEA = 100/0.1 (v/v); *t*_R1_ = 10.75 min (minor), *t*_R2_ = 14.63 min (major)].

#### *N*-{[(3*R*)-2,6-Dimethyl-1,2,3,4-tetrahydroisoquinolin-3-yl]­methyl}-2,3-dihydro-1*H*-indene-2-carboxamide (62)

The compound was prepared by general procedure I with [(3*R*)-2,6-dimethyl-1,2,3,4-tetrahydroisoquinolin-3-yl]­methanamine **15i** (0.130 g, 0.683 mmol), 2-indanecarboxylic acid (0.111 g, 0.683 mmol), 1-(3-(dimethylamino)­propyl)-3-ethylcarbodiimide hydrochloride (0.196 g, 1.02 mmol), 1-hydroxybenzotriazole hydrate (0.139 g, 1.02 mmol), and *N*,*N*-diisopropylethylamine (0.298 mL, 1.71 mmol) in dichloromethane (6.80 mL). The crude product was purified by flash column chromatography (SiO_2_, 3% methanol in dichloromethane) to give the desired product, *N*-{[(3*R*)-2,6-dimethyl-1,2,3,4-tetrahydroisoquinolin-3-yl]­methyl}-2,3-dihydro-1*H*-indene-2-carboxamide **62,** as a white solid (0.137 g, 60%). [α]_D_ ^20^ = +48.21° (c = 1, MeOH); ^1^H NMR (400 MHz, CDCl_3_) δ: 7.23–7.13 (m, 4H), 6.99**–**6.90 (m, 3H), 6.24 (br s, 1H), 3.82 (d, 1H), 3.67 (d, 1H), 3.50 (dt, 1H), 3.39 (dt, 1H), 3.29–3.11 (m, 5H), 2.91–2.83 (m, 1H), 2.80–2.67 (m, 2H), 2.38 (s, 3H), 2.30 (s, 3H); ^13^C NMR (150 MHz, CDCl_3_) δ: 175.23, 141.97, 136.16, 133.16, 130.66, 129.25, 127.04, 126.66, 126.43, 124.41, 57.58, 56.12, 45.91, 40.98, 39.84, 36.95, 36.93, 29.29, 21.16. LC-MS (ESI) *m*/*z* 335.2 [M + H]^+^; HRMS–ESI (*m*/*z*): [M + H]^+^ calcd for C_22_H_27_N_2_O, 335.2118; found, 335.2125; UPLC purity = 99% (*t*_R_ = 12.9 min); 95.4% ee [MeOH/DEA = 100/0.1 (v/v); *t*_R1_ = 10.66 min (major), *t*_R2_ = 14.75 min (minor)].

### Biology

#### FLIPR Calcium Assay

The FLIPR calcium assay was employed using CHO-K1 cells stably coexpressing the μ-opioid receptor (MOR) (CHO-K1/MOR/Gα15; GenScript). MOR activation leads to intracellular calcium mobilization, which is detected as an increase in RFU. The extent of intracellular Ca^2+^ release was quantified by calculating the AUC of the real-time FLIPR signal in response to compound treatment. Data are expressed as percentages relative to the response induced by BPR1M97, a reference MOR agonist (AUC = 19,476). Test compounds were excluded from further evaluation if their maximal efficacy failed to reach 20% of the BPR1M97 *E*_max_ under identical assay conditions. Twenty four hours prior to the assay, CHO-K1/MOR/Gα15 cells were seeded at a density of approximately 3.7 × 10^4^ cells/well in “CELL CULTURE MICROPLATE, 96 WELL, PS, HALF AREA, μCLEAR®, BLACK, CELLSTAR®” (Greiner) with F12 medium and incubated overnight at 37 °C in a humidified atmosphere containing 5% CO_2_. On the assay day, FLIPR calcium assay reagent (Cat# R8141; Molecular Devices, Sunnyvale, CA, USA) was reconstituted in 1× assay buffer composed of HBSS supplemented with 5 mM KCl, 0.3 mM KH_2_PO_4_, 138 mM NaCl, 4 mM NaHCO_3_, 0.3 mM Na_2_HPO_4_, 5.6 mM d-glucose, 20 mM HEPES, and 13 mM CaCl_2_ (pH 7.4), with the addition of 2.5 mM probenecid to prevent dye extrusion. Forty microliters of this mixture were added to each well and incubated for 1 h at 37 °C. Test compounds were diluted in assay buffer and applied to the cells using FlexStation III (Molecular Devices), which also recorded fluorescence signals at 1.52 s intervals following robotic compound injection. Fluorescence emission was monitored at 525 nm upon excitation at 485 nm, with data collection continuing for 336 s postinjection.

#### β-Arrestin-2 Recruitment Assay

The recruitment of β-arrestin-2 to the MOR was assessed using the PathHunter β-Arrestin Assay system (Eurofins DiscoverX), following the manufacturer’s instructions. CHO-K1 cells stably expressing MOR and the β-arrestin-2 enzyme acceptor fusion protein (CHO-K1-MOR) were seeded at a density of 5,000 cells per well into black 384-well assay plates containing CP2 reagent (Cat# 93–0563R2A; Eurofins DiscoverX) and incubated for 24 h at 37 °C in a humidified incubator prior to stimulation. Following compound treatment, cells were incubated at rt for 90 min to allow β-arrestin recruitment. Subsequently, 8 μL of the PathHunter Detection Reagent (Cat# 93–0001; Eurofins DiscoverX) was added to each well, and the plates were incubated for an additional 60 min at rt. Luminescence signals, indicative of β-arrestin-2 recruitment to the activated MOR, were measured using a Victor 2 plate reader (PerkinElmer, Waltham, MA, USA). All measurements were performed on the same day using cells from the same passage to ensure reproducibility and to minimize interassay variability.

#### Animals and Housing Conditions

Male C57BL/6J (B6) mice (8–10 weeks old) were purchased from the National Institutes of Applied Research (National Center for Biomodels, Taiwan), whereas male hMOR^+^; mMOR^–/–^ mice were bred in-house. All animals were housed in a temperature-controlled room (22–24 °C) under a 12 h light/dark cycle (lights on at 7:00 AM) with *ad libitum* access to food and water. The experimental protocol was approved by the Institutional Animal Care and Use Committee (IACUC) of the National Health Research Institute (NHRI) of Taiwan. All procedures were conducted in accordance with the Policies on the Use of Animals in Neuroscience Research and ethical guidelines for pain research in animals, as established by the International Association for the Study of Pain (IASP). In each experiment, animals were randomly assigned to different treatment groups to minimize bias. B6 mice were used for CPP and physical dependence tests, whereas all other behavioral experiments were conducted using hMOR^+^; mMOR^–/–^ mice. All animal experiments were performed following the protocols evaluated and approved by the Institutional Animal Care and Use Committee at the National Health Research Institutes (Ethics Approval Number: NHRI-IACUC-110124).

#### Tail-Flick Test

Antinociceptive responses to pharmacological treatments targeting acute thermal pain hypersensitivity were evaluated using a Tail-Flick Analgesia Meter (Columbia Instruments, Columbus, OH, USA). Animals exhibiting a baseline tail-flick latency of 2.5–3.0 s were selected and randomly assigned to treatment groups. The baseline latency was measured prior to drug administration. Tail-flick latencies were recorded at 30, 60, 90, and 120 min following s.c. administration of the test compounds. A cutoff latency of 10 s was imposed to prevent tissue damage. The antinociceptive effect was quantified as the difference between tail-flick latency and baseline latency at each time point. To calculate the 50% effective dose (ED_50_), six different doses of the drug were administered to mice, and the corresponding antinociceptive effects were measured. The dose–response relationship was analyzed using GraphPad Prism (version 10.6.0) under the “Dose-Response–Stimulation” module. Specifically, the data were fitted using the “log­(agonist) vs. response–variable slope (four-parameter logistic)” nonlinear regression model. This approach estimates the ED_50_ as the dose that produces 50% of the maximal antinociceptive effect, considering the Hill slope, minimum and maximum plateaus, and response variability across animals. ED_50_ values were reported with 95% confidence intervals.

#### Incisional Pain Model

The incisional pain model was established in adult male hMOR^+^; mMOR^–/–^ mice (23–28 g) as previously described by Brennan et al.,^48^ with minor modifications. Mice were anesthetized with isoflurane (2–3% for induction, 1–2% for maintenance) delivered via a nose cone. After achieving adequate anesthesia, the plantar surface of the right hind paw was sterilized with 70% ethanol, and a 5 mm longitudinal incision was made through the skin and fascia, beginning 2 mm from the proximal edge of the heel and extending toward the toes. The underlying plantaris muscle was also incised longitudinally without damaging the deeper tissues. The wound was closed using a single 6–0 nylon suture. After wound closure, the animals were allowed to recover for 10 min before the baseline mechanical sensitivity was assessed using von Frey filaments. Mechanical allodynia was measured by applying calibrated von Frey filaments to the plantar surface adjacent to the incision site. The 50% paw withdrawal threshold was determined using the up–down method. Subsequently, compound **56** or TRV130-HCl was administered subcutaneously. Ten minutes postinjection, the von Frey test was performed again to evaluate the rapid antinociceptive effect of the drug in the postoperative condition.

#### Oxygen Saturation Evaluation

Oxygen saturation was assessed under injectable anesthesia to ensure consistent physiological monitoring with minimal motion artifacts. Mice were anesthetized with a cocktail of ketamine (100 mg/kg) and xylazine (10 mg/kg) administered intraperitoneally. Following the induction of anesthesia, the fur over the neck region was carefully removed by using electric clippers to ensure optimal sensor contact. Peripheral oxygen saturation (SpO_2_) was continuously monitored using a neck collar sensor attached to a MouseSTAT Pulse Oximeter and Heart Rate Monitor (Physiosuite, Kent Scientific, CT, USA). After a 10 min stabilization period, the baseline SpO_2_ values were recorded. The mice were subsequently administered a subcutaneous injection of the vehicle, compound **56**, or TRV130-HCl. Oxygen saturation was measured at 10, 20, and 30 min after drug administration to assess the potential respiratory effects of the treatments. Oxygen saturation and the accompanying physiological parameters were measured at multiple time points after drug administration to assess the potential respiratory and cardiovascular effects associated with the treatment.

#### Colonic Transit Analysis

Male hMOR^+^; mMOR^–/–^ mice were fasted for 6 h prior to the experiment, with *ad libitum* access to water. For the charcoal meal test, the animals received either compound **56** (subcutaneous, various doses) or TRV130-HCl (subcutaneous, various doses) 30 min before the oral administration of an aqueous activated charcoal suspension (10% activated charcoal with 5% gum Arabic; 0.3 mL).

After 30 min, the mice were euthanized by an intraperitoneal injection of a ketamine/xylazine cocktail, followed by cervical dislocation. The entire small intestine, from the pylorus to the ileocecal junction, was carefully excised to measure both the total length and the distance traveled by the charcoal meal. The % of gastrointestinal (GI) transit was calculated as follows:

%ofgastrointestinaltransit=distancetraveledbycharcoalmeal/fulllengthofthesmallintestine×100%

To normalize interindividual variation, GI propulsion was quantified using the same metric. The percentage of GI inhibition relative to that in the control group was calculated by using the following formula:

%ofgastrointestinalinhibition=(controlgroup−drug−treatedgroup)/controlgroup×100%

A GI propulsion rate of <55% was considered indicative of substantial inhibition of intestinal motility.

#### Conditioned Place Preference (CPP) Test

The reward effects of compound **56** and TRV130-HCl were evaluated using a CPP apparatus (MED Associates, East Fairfield, VT, USA) equipped with automated infrared (IR) photobeam detectors for real-time tracking of animal positions. The apparatus consisted of two distinct conditioning chambers (21 × 21 cm^2^ each) connected by a neutral gray center compartment with autoguillotine doors on either side to control access. The two chambers were differentiated using multiple contextual cues. The black compartment featured a stainless-steel grid rod floor, bright LED lighting, and black walls, whereas the white compartment was equipped with a wire mesh floor, dim LED lighting, and white walls. These physical and visual contrasts enabled strong contextual association in place conditioning. On day 1 (pretest), male C57BL/6J mice were placed in the central compartment with free access to all three chambers for 15 min. The time spent in each compartment was automatically recorded using an IR detection system. Based on the pretest results, the less-preferred compartment (i.e., the chamber in which each mouse spent less time) was designated as the drug-paired compartment to avoid biased preference. During the conditioning phase (days 2–5), the mice received a subcutaneous injection of vehicle and were confined to the vehicle-paired chamber. Five hours later, the mice were administered compound **56** or TRV130-HCl (s.c.) and placed in the drug-paired chamber for 30 min with the doors closed. On day 6 (expression test), the animals received no treatment and were placed in the neutral compartment with access to both chambers for 15 min. The time spent in each compartment was automatically recorded using a photobeam system. The CPP score was calculated by subtracting the time spent in the vehicle-paired compartment from that spent in the drug-paired compartment.

$$CPP\;Score=Time_{drug-paired}-Time_{vehicle-paired}$$

An increase in the CPP score during the expression test, compared with the pretest, was interpreted as a conditioned preference for the drug-paired compartment, indicating a rewarding effect of the treatment.

#### Physical Dependence Test

To assess physical dependence liability, male C57BL/6J mice were administered subcutaneous (s.c.) injections twice daily for five consecutive days. The treatment groups included vehicle, compound **56** (increasing doses of 0.054, 0.108, 0.162, 0.216, and 0.270 mg/kg), and TRV130-HCl (increasing doses of 0.514, 1.028, 1.542, 2.056, and 2.570 mg/kg). On day 6, the mice received a single dose of vehicle, compound **56** (0.270 mg/kg), or TRV130-HCl (2.570 mg/kg). Two hours after the final dose, naloxone (1 mg/kg, subcutaneously) was administered to induce opioid withdrawal. Immediately following the naloxone injection, each mouse was placed individually in a transparent cylindrical observation chamber (10 cm in diameter × 30 cm in height), and withdrawal behaviors were monitored for 15 min. Paw tremors were counted throughout the session, with one point assigned every five tremors. Additionally, wet dog shakes, tooth chattering, diarrhea, and ptosis were recorded as discrete behavioral events. These signs were scored based on their occurrence at each 5 min interval, with a maximum of three points per behavior. For each animal, a composite withdrawal score was calculated by summing the points assigned to all observed behaviors, providing a quantitative measure of withdrawal severity.

#### Protein–Ligand Docking and Molecular Dynamics Simulations

The three-dimensional structures of the ligands used in this study were generated using the Python programming language and the open-source RDKit library.^49^ The protein structure was derived from the antagonist-bound MOR structure (Protein Data Bank [PDB] IDs: 4DKL^50^ and 5C1M^51^). After removing the bound ligand, protein structures were prepared and protonated using the AMBER-FB19SB force field.^52^ AutoDock Vina^41^ was used to dock single ligands following previously reported protocols. Once the desired ligands were docked, the explicit solvent and all-atom classical representations of the protein–ligand-membrane systems were reconstructed for further molecular dynamics simulations using the CHARMM-GUI server.^53^ The conserved disulfide bridge between Cys140 and Cys217 ^54^ was maintained, and the protein–ligand structures were embedded in a 1-palmitoyl-2-oleoyl-*sn*-glycero-3-phosphocholine (POPC) and 30% cholesterol membrane within a 67 Å membrane size containing 150 mM NaCl. Subsequently, the CHARMM-GUI files were converted to the Amber format. All simulations were conducted in Amber20, utilizing the FF19SB force field for proteins, the GAFF2 force field for ligands,^55^ the Lipid21 force field^56^ for lipid bilayers, and the TIP3P model for parametrizing water molecules.

The initial energy minimization was performed using the steepest-descent method, followed by multistage equilibration steps totaling 1875 ps across five stages at a target temperature of 303.15 K. During these stages, positional restraints on lipid phosphate atoms, water, ions, proteins, and ligands were gradually released. A final 50 ns simulation in the NPT ensemble was run, but this brief simulation was not included in the analysis. The production simulation, lasting 1.5 μs, was carried out semi-isotropically using the Langevin thermostat in the NPT ensemble at 1 bar pressure, employing the Berendsen barostat method. A nonbonded force cutoff of 9 Å was set for the van der Waals and electrostatic interactions. All the generated simulations were used for subsequent trajectory analyses.

For trajectory analysis, RMSD per residue and minimum distance analyses between all heavy atoms of the residues and ligands were conducted using CPPTRAJ.^57^ Residues in close contact with ligands were selected for minimum distance analysis using the native contact analysis in CPPTRAJ. Visualization and modeling of the images were performed using PyMOL software.

#### Pharmacokinetic Study

The animal study was conducted according to procedures approved by the NHRI Institutional Animal Care and Committee. Male ICR mice (weighing approximately 38–41 g) were obtained from BioLASCO (Taiwan Co. Ltd., Ilan, Taiwan). A single 0.1 or 1.0 mg/kg subcutaneous dose of compound **56**, formulated as DMA/Solutol-15/95% glucose water (5/5/90, v/v/v), was separately administered to the mice. At 0 (before dosing), 15 min, 30 min, 1, 2, 4, 6, 8, 16, and 24 h after dosing, blood and brain samples were collected from groups of three mice at each time point by cardiac puncture and plasma was separated from the blood by centrifugation and stored in a freezer (−80 °C) before analysis. All samples were analyzed using LC-MS/MS. The plasma and brain concentrations data were analyzed using a noncompartmental method.

#### Polarized Light Microscopy

PLM images were acquired by using a Leica DM2500P microscope. A small amount of the sample (<1 mg) was placed on a glass slide, and a drop of oil was applied before being covered with a coverslip. The specimen was examined by using the eyepiece, and representative regions were selected for image acquisition and evaluation.

#### Dynamic Vapor Sorption

DVS measurements were performed by using a Q5000 SA vapor sorption analyzer (TA Instruments). The experimental parameters are listed in Table 5.

**Table 5:** 

Parameters	Values
Drying	Temperature: 25 °C; Drying time: 360 min
Adsorption/desorption	Temperature: 25 °C; Equilibrium criterion: 0.01 wt % in 5 min; Max equilibrium time: 120 min
Relative humidity steps	RH % steps: 5, 10, 15. 20, 25, 30, 35, 40, 45, 50, 55, 60, 65, 70, 75, 80, 85, 90, 95, 90, 85, 80, 75, 70, 65, 60, 55, 50, 45, 40, 35, 30, 25, 20, 15, 10, 5 (2 cycles)

#### Karl Fischer (KF) Titration

The water content was determined using a C30 Compact Karl Fischer Coulometer (Mettler Toledo). The parameters for the KF titration are listed in the table below.


**Parameters**

**Values**
Mix Time60 sStir Speed45%End point100.0
mVControl band250.0
mVRateNormalGenerator currentAutomaticDrift<25 μg/mL

#### pH Solution Stability

The pH solution stability was evaluated to determine potential chemical degradation of the compound across varying pH conditions (pH 1–8) at 40 °C. A cosolvent was used, when necessary, to ensure solubility in the pH range. The media included pH 1 (0.1 N HCl), pH 2, 4, 6, and 8 (50 mM sodium phosphate buffer), as well as pH 2 and 8 buffers containing 0.3% H_2_O_2_. The compound solutions were stored at 40 °C for 0 (initial standard), 4, 8, 12, 16, 20, or 24 h. The percentage of the remaining compounds relative to the initial standard was determined using HPLC.

#### Solid-State Stability

Approximately 2 mg of compound **56** was weighed in glass vials. Samples were subjected to stressed conditions (vial caps removed for humidity testing), while standards were stored at −20 °C. After 7 days, one set of stressed samples was transferred to −20 °C. After 14 days, all the remaining stressed samples, standards, and 7 days samples were collected. Physical changes (e.g., discoloration) were recorded prior to dilution, and chemical stability was evaluated by HPLC.

## Supplementary Material









## Notes

The synthetic procedure of all intermediates, ^1^H and ^13^C NMR spectra of **16**–**62**, HPLC chromatograms of **56** and **62**, UPLC chromatogram of **60**, and chiral HPLC chromatograms of **55–62** (PDF)
Molecular formula strings (CSV)
PDB files of 55 with MOR (PDB)
PDB files of 56 with MOR (PDB)

## Abbreviations

AUC: area under curve
cAMP: cyclic adenosine monophosphate
CHO: Chinese hamster ovary
CPP: conditioned place preference
CYP: cytochrome P450
DIPEA: *N*,*N*-diisopropylethylamine
DOR: δ-opioid receptor
DVS: dynamic vapor sorption
*E*_max_: maximum effect
EC_50_: concentration for 50% of maximal effect
ED_50_: dose for 50% of maximal effect
EDCI: *N*-ethyl-*N*’-(3-(dimethylamino)­propyl)­carbodiimide
FLIPR: fluorescent imaging plate reader
GI: gastrointestinal
GPCR: G-protein coupled receptors
hERG: human ether-à-go-go-related gene
HOBt: hydroxybenzotriazole hydrate
KOR: κ-opioid receptor
LRMS: low-resolution mass spectra
MOR: μ-opioid receptor
NMDAR: *N*-methyl-*d*-aspartate receptor
NOP: nociception-orphanin FQ peptide
Rt: room temperature
SAR: structure–activity relationship
S.C.: subcutaneous
THF: tetrahydrofuran
