# Supplementary material for: Structural Modification and Development of N‑(1,2,3,4-Tetrahydro-3-isoquinolinylmethyl)benzamide, BPR1M492, as a Potent and Rapid-Onset Opioid Analgesic with Reduced Withdrawal Symptoms
Source: J Med Chem. 2026 Jun 1;69(11):13222–47. doi: 10.1021/acs.jmedchem.6c00215 (PMC13266996; doi:10.1021/acs.jmedchem.6c00215)
Supplement: Supplementary file 1 [file jm6c00215_si_001.pdf]

The Supporting Information of

Structural modification and development of an *N*-(1,2,3,4-tetrahydro-3-isoquinolinylmethyl)benzamide, BPR1M492, as a potent and rapid-onset opioid analgesic with reduced withdrawal symptoms

Po-Wei Chang, [†a] Yung-Chiao Chang, [†a] Ya-Wen Tien, [a] Hsiao-Fu Chang, [a] Sheng-Ren Chen, [a] Ya-Ping Chen, [a] Wei-Cheng Huang, [a] Chih-Chien Hsieh, [a] Ling Chang, [a] Hong Zhuang, [a] Teng-Kuang Yeh, [a] Chun-Wei Tung, [a] Shiu-Hwa Yeh, [a,c*] Shau-Hua Ueng [a,b*]

[a] Institute of Biotechnology and Pharmaceutical Research, National Health Research Institutes, Miaoli County 35053, Taiwan, R. O. C.

[b] School of Pharmacy, College of Medicine, National Cheng Kung University, Tainan 701, Taiwan, R. O. C.

[c] The Ph.D. Program in Medical Neuroscience, College of Medical Science and Technology, Taipei Medical University and National Health Research Institutes, Taipei, 110, Taiwan, ROC

Corresponding Authors

[*] Tel: +886-37-246166 ext. 35791. Fax: +886-37-586456. E-mail: shueng@nhri.edu.tw

[*] Tel: +886-37-246166 ext. 35759. Fax: +886-37-586456. E-mail: bau9763@nhri.edu.tw

Table of Contents

Dose-responsive curves of antinociception of compounds **56**, **60**, and **62**--S3

The on-set time of antinociception of compound **56**--S3

The antinociceptive and gastrointestinal transit inhibition effect of compound **56** in MOR$^{-/-}$ model-----------------S3

Effects on cardiopulmonology in a mMOR$^{-/-}$ mouse model treating with compound **56**-------------------------S3

NMDAR channel inhibition of compound **56**---S4

CYP enzyme (CYP1A, 2B6, 2C8, 2C9, 3A, 2C19, and 2D6) inhibition of compound **56**---------------------------S4

hERG channel inhibition of compound **56**---S5

Plasma and brain concentrations of 0.1 mg/kg compound **56** at two time points---------------------------------S5

% Remaining of compound **56** stored at 40 ^{O}C in different pH buffers for 24 hours--S6

The results of solid-state stability of compound **56**---S6

HPLC condition for monitoring of compound **56** in solution and solid-state stability-------------------------S6

Synthetic procedure of intermediates **2b–15i**--S7

^{1}H and ^{13}C NMR spectra of final compounds **16–62**---S38

HPLC chromatograms of compounds **56**, **62**, and UPLC chromatogram of **60**-------------------------------------S85

Chiral HPLC chromatograms of compounds **55–62**---S86

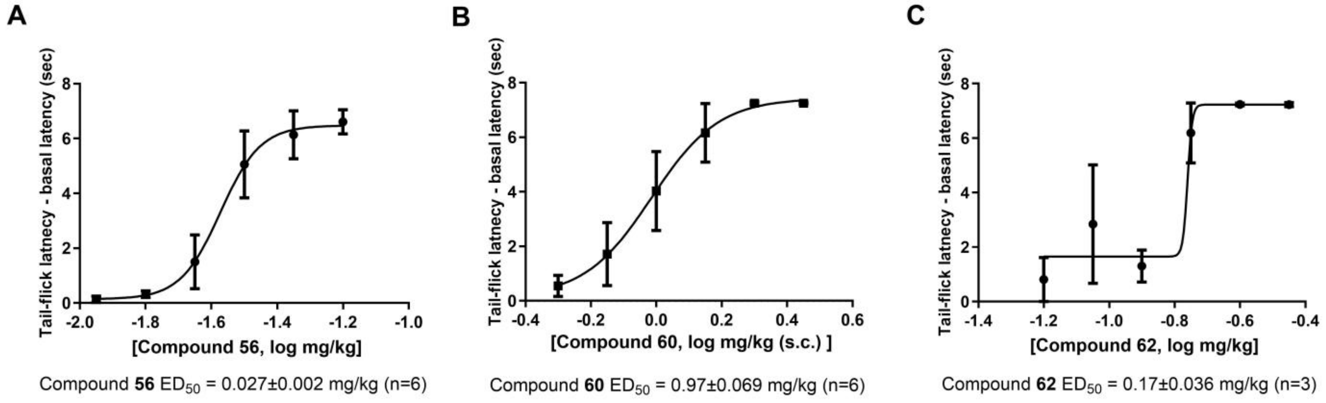

Figure S1. Dose-responsive curves of antinociception of compounds (A) **56**, (B) **60**, and (C) **62**.

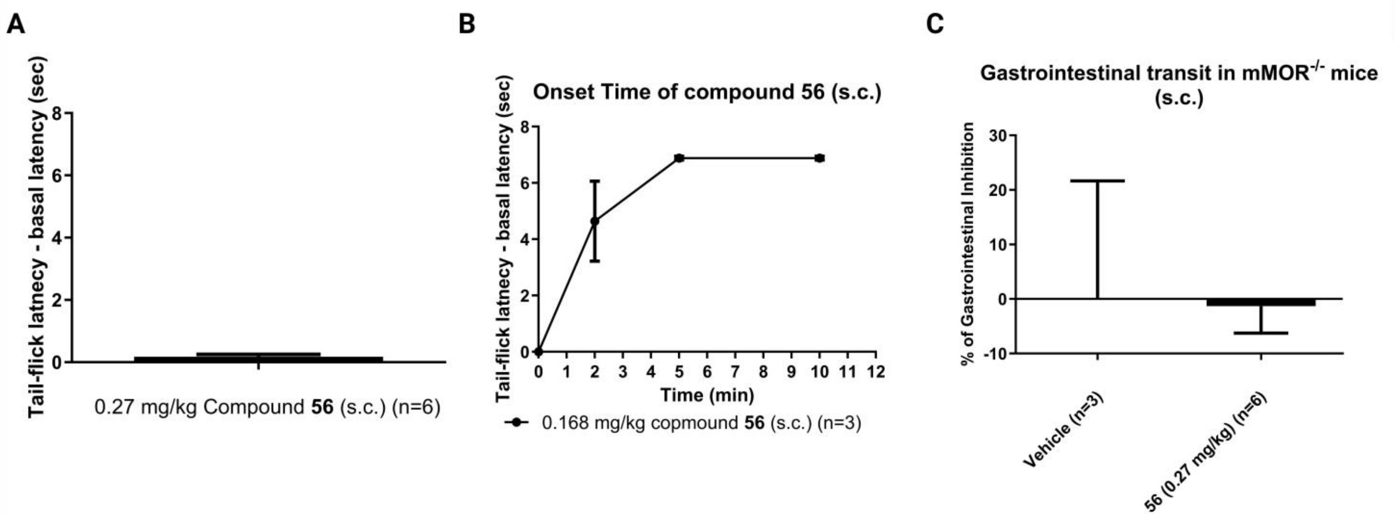

Figure S2. (A) The antinociceptive effect of 0.27 mg/kg compound **56** in the mMOR$^{-/-}$ mouse model at 30 min after administration. (B) The onset time of antinociception of compound **56**. (C) The gastrointestinal transit inhibition effect of 0.27 mg/kg compound **56** in the mMOR$^{-/-}$ mouse model.

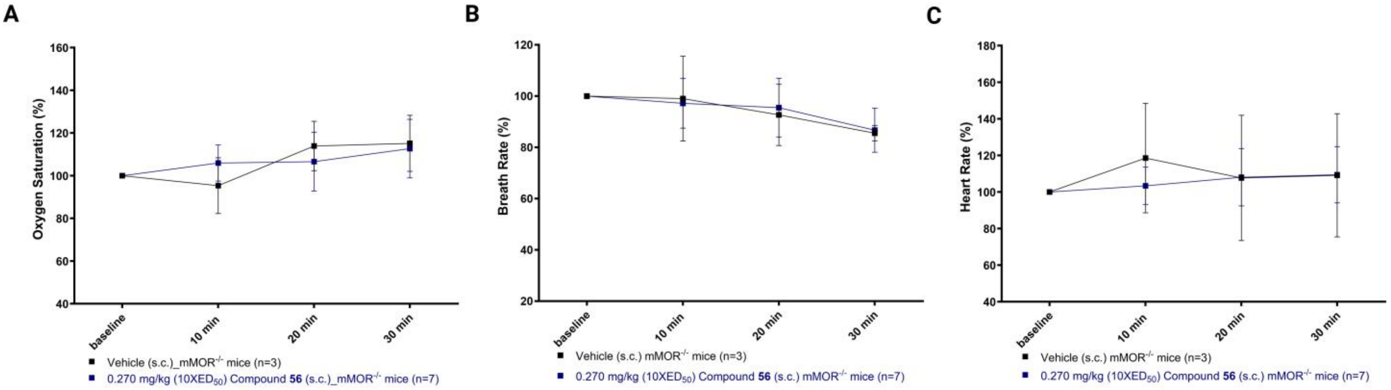

Figure S3. Effects on (A) oxygen saturation levels, (B) respiratory rate, and (C) heart rate in a mMOR$^{-/-}$ mouse model treating with compound **56**.

Results: Summary Table

Compound Name	Order ID	Target Class	Assay Name	Mode	Assay Target	Result Type	Value Prefix	RC50 (µM)	Hill	Curve Bottom	Curve Top	Max Response
BPR1M0492S0	US073TW04-0018156	Ion Channel	Ion Channel	Blocker	NMDAR (1A/2B)	IC50	>	3				0
BPR1M0492S0	US073TW04-0018156	Ion Channel	Ion Channel	Blocker	NMDAR (1A/2B)	IC50	>	3				5.16

Figure 3: Summary of compound(s) tested

Figure S4. NMDAR channel inhibition of compound **56**.

Compound I.D.	Client Compound I.D.	IC50(M)	nH	Test Concentration	%Inhibition 1st	%Inhibition 2nd	Mean %Inhibition	Flags 1st	Flags 2nd
100067708-9	1M492	> 1.0E-04	n/a	3.0E-08 M	19.1	-10.0	4.5		
				1.0E-07 M	67.1	16.3	16.3	0	
				3.0E-07 M	2.2	-6.7	-2.2		
				1.0E-06 M	18.2	1.3	9.7		
				3.0E-06 M	1.4	6.5	4.0		
				1.0E-05 M	6.2	4.3	5.2		
				3.0E-05 M	11.5	3.0	7.2		
				1.0E-04 M	34.7	26.2	30.5		

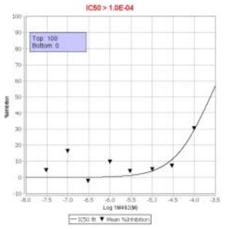

1M492 on CYP1A inhibition (HLM, phenacetin substrate) IC50

Compound I.D.	Client Compound I.D.	IC50(M)	nH	Test Concentration	%Inhibition 1st	%Inhibition 2nd	Mean %Inhibition
100067708-9	1M492	2.6E-05 M	0.6	3.0E-08 M	16.2	6.9	11.6
				1.0E-07 M	23.9	18.5	21.2
				3.0E-07 M	19.3	23.2	21.3
				1.0E-06 M	26.6	25.2	25.9
				3.0E-06 M	34.0	32.0	33.0
				1.0E-05 M	44.3	40.6	42.4
				3.0E-05 M	62.0	57.5	59.8
				1.0E-04 M	76.0	75.6	75.8

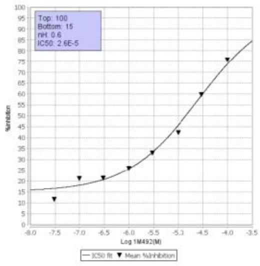

1M492 on CYP2C19 inhibition (HLM, omeprazole substrate) IC50

Compound I.D.	Client Compound I.D.	IC50(M)	nH	Test Concentration	%Inhibition 1st	%Inhibition 2nd	Mean %Inhibition
100067708-9	1M492	1.3E-04 M	0.3	3.0E-08 M	-1.3	0.0	-0.7
				1.0E-07 M	6.1	14.6	10.3
				3.0E-07 M	14.2	23.1	18.6
				1.0E-06 M	17.7	22.0	19.8
				3.0E-06 M	17.3	19.6	18.4
				1.0E-05 M	24.7	26.2	25.5
				3.0E-05 M	33.7	32.1	32.9
				1.0E-04 M	55.2	51.9	53.6

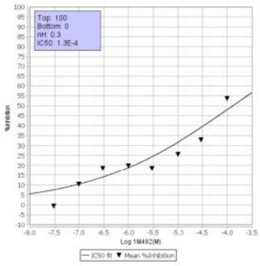

Figure 20. 1M492 on CYP2B6 inhibition (HLM, bupropion substrate) IC50

Compound I.D.	Client Compound I.D.	IC50(M)	nH	Test Concentration	%Inhibition 1st	%Inhibition 2nd	Mean %Inhibition
100067708-9	1M492	> 1.0E-04	n/a	3.0E-08 M	-6.4	-5.2	-5.8
				1.0E-07 M	-1.9	3.3	0.7
				3.0E-07 M	5.9	11.0	8.4
				1.0E-06 M	12.9	12.5	12.7
				3.0E-06 M	13.6	16.2	14.9
				1.0E-05 M	14.0	18.6	16.3
				3.0E-05 M	20.0	22.7	21.3
				1.0E-04 M	34.0	35.7	34.8

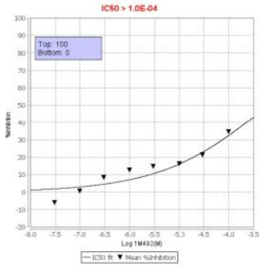

1M492 on CYP2C9 inhibition (HLM, diclofenac substrate) IC50

Compound I.D.	Client Compound I.D.	IC50(M)	nH	Test Concentration	%Inhibition 1st	%Inhibition 2nd	Mean %Inhibition	Flags 1st	Flags 2nd
100067708-9	1M492	> 1.0E-04	n/a	3.0E-08 M	17.6	12.2	14.9		
				1.0E-07 M	17.8	25.1	21.4		
				3.0E-07 M	26.8	34.9	30.8		
				1.0E-06 M	22.5	23.8	23.2		
				3.0E-06 M	11.4	11.7	11.6		
				1.0E-05 M	-0.7	-2.7	-1.7		
				3.0E-05 M	13.6	10.5	12.1		
				1.0E-04 M	54.7	50.7	52.7		

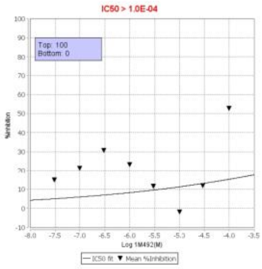

1M492 on CYP3A inhibition (HLM, testosterone substrate) IC50

Compound I.D.	Client Compound I.D.	IC50(M)	nH	Test Concentration	%Inhibition 1st	%Inhibition 2nd	Mean %Inhibition
100067708-9	1M492	2.1E-06 M	0.7	3.0E-08 M	-4.6	-7.4	-6.0
				1.0E-07 M	18.6	20.4	19.5
				3.0E-07 M	6.8	11.2	9.0
				1.0E-06 M	38.9	41.7	40.3
				3.0E-06 M	58.7	61.8	60.2
				1.0E-05 M	76.9	77.6	77.3
				3.0E-05 M	84.4	84.0	84.2
				1.0E-04 M	89.3	90.1	89.7

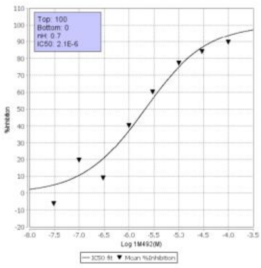

1M492 on CYP2D6 inhibition (HLM, dextromethorphan substrate)IC50

Compound I.D.	Client Compound I.D.	IC50(M)	nH	Test Concentration	%Inhibition 1st	%Inhibition 2nd	Mean %Inhibition
100067708-9	1M492	> 1.0E-04	n/a	3.0E-08 M	23.0	18.9	21.0
				1.0E-07 M	28.8	25.0	26.9
				3.0E-07 M	32.4	27.1	29.8
				1.0E-06 M	31.5	30.3	30.9
				3.0E-06 M	32.5	31.2	31.8
				1.0E-05 M	35.3	32.7	34.0
				3.0E-05 M	47.4	42.7	45.0
				1.0E-04 M	54.4	52.7	53.6

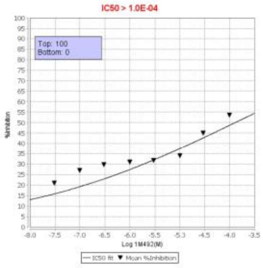

1M492 on CYP2C8 inhibition (HLM, amodiaquine substrate) IC50

Figure S5. CYP1A, 2B6, 2C8, 2C9, 3A, 2C19, and 2D6 inhibition of compound **56**.

EXPERIMENTAL RESULTS

CiPA hERG Assay Data Table

Compound ID	Client Compound ID	Batch Number	Concentration (μM)	% inhibition		
				n1	n2	mean
TW04-0015527-1	BPR1M0492S0-2	09BP-067-134C	0.0001	14.64	10.72	12.68
TW04-0015527-1	BPR1M0492S0-2	09BP-067-134C	0.001	6.76	4.99	5.88
TW04-0015527-1	BPR1M0492S0-2	09BP-067-134C	0.01	10.38	10.82	10.60
TW04-0015527-1	BPR1M0492S0-2	09BP-067-134C	0.1	13.68	14.61	14.15
TW04-0015527-1	BPR1M0492S0-2	09BP-067-134C	1	33.52	34.16	33.84
TW04-0015527-1	BPR1M0492S0-2	09BP-067-134C	10	80.39	78.73	79.56
Time-Matched Vehicle Control	0.33% DMSO Addition 1			9.70	9.84	9.77
Positive Reference Control	Verapamil		0.01	14.52	11.50	13.01
Positive Reference Control	Verapamil		0.03	16.31	13.41	14.86
Positive Reference Control	Verapamil		0.1	25.50	22.10	23.80
Positive Reference Control	Verapamil		0.3	45.03	42.36	43.69
Positive Reference Control	Verapamil		1	69.99	68.87	69.43
Positive Reference Control	Verapamil		3	84.68	85.32	85.00

Estimated IC_{50} Compound Summary Table

Compound	Target	Mode	Estimated IC_{50} (μM)
BPR1M0492S0-2	hERG	Antagonist	1.92

REFERENCE COMPOUND RESULTS

CiPA hERG Reference Compound Table

ITEM	Assay Name	Mode	Reference Compound	Estimated IC_{50} (μM)
CYL8038QB2DR	hERG Human Potassium Channel Assay Qube APC	Antagonist	Verapamil	0.371

Figure S6. Assay data of hERG channel inhibition of compound **56**.

Table S1. Plasma and brain concentrations of 0.1 mg/kg compound **56** at two time points.

Compound **56** in plasma	Time (hr)	Observed drug conc. (ng/mL)
	0.5	11.7±2.2
	1	2.1±0.5
Compound **56** in brain	Time (hr)	Observed drug conc. (ng/mg)
	0.5	72.4±11.4
	1	21.2±3.3

After a single injection of compound **56** (0.1 mg/kg, s.c.), plasma and brain samples were collected from each mouse at the indicated time points (30 min and 1 h). The observed drug concentrations were measured and calculated average. n = 3 per group. Values indicate the mean ± SD. SD, standard deviation.

Table S2. % Remaining of compound **56** stored at 40 ^{O}C in different pH buffers for 24 hours.

Time (h)	pH 8/ 0.3% H_2O_2	pH 2/ 0.3% H_2O_2	pH 8	pH 6	pH 4	pH 2	0.1N HCl (pH 1)
0	100.0	100.0	100.0	100.0	100.0	100.0	100.0
4	90.5	98.7	98.7	98.5	98.5	99.8	97.4
8	88.8	99.5	98.6	99.3	99.6	99.1	99.5
12	88.4	99.3	99.5	99.8	99.4	99.8	99.4
16	88.0	99.4	99.6	100.0	99.7	99.8	100.2
20	87.8	99.3	99.7	100.4	99.9	99.8	99.7
24	88.0	99.5	99.8	100.5	99.9	100.2	99.6

Table S3. The results of solid-state stability of compound **56**.

Condition	Time	Weight (mg)	TRS*(%)	Appearance	Conc. (μg/mL)	Peak area	Cal. Weight (mg)	Remaining (%) (%)	Remaining (%) Mean
-20°C	STD	2.0681	5.71	No change	206.8	459348	-	-	-
	CSTD	2.0768	5.59	No change	207.7	461936	2.0798	100.1	-
25°C/ light#	7d	2.0317	10.07	No change	203.2	437581	1.9701	97.0	95.0
		2.2196	10.70	No change	222.0	458572	2.0646	93.0	
	14d	2.0628	13.63	No change	206.3	426644	1.9209	93.1	93.3
		2.1627	13.14	No change	216.3	449272	2.0227	93.5	
40°C	7d	2.2434	5.79	No change	224.3	496304	2.2345	99.6	99.7
		2.1345	6.00	No change	213.5	472829	2.1288	99.7	
	14d	2.4157	6.51	No change	241.6	531640	2.3936	99.1	99.1
		2.0655	6.54	No change	206.6	454639	2.0469	99.1	
40°C/75% RH	7d	2.0972	5.51	No change	209.7	465146	2.0942	99.9	99.8
		2.0482	5.99	No change	204.8	454075	2.0444	99.8	
	14d	2.0975	6.35	No change	209.8	461325	2.0770	99.0	99.1
		2.0639	5.83	No change	206.4	454725	2.0473	99.2	
70°C	7d	2.2798	28.50	Change	228.0	434426	1.9559	85.8	85.0
		2.1022	30.84	Change	210.2	393507	1.7717	84.3	
	14d	2.0790	26.74	Change	207.9	396331	1.7844	85.8	86.7
		2.2529	23.72	Change	225.3	437979	1.9719	87.5	

*, total related substances
#, illumination of 2442 lux and an ultraviolet energy of 6 micro-watt/square centimeter (0.06 watt/square meter)

Table S4. HPLC condition for monitoring of compound **56** in solution and solid-state stability.

HPLC System	Waters e2695 HPLC coupled with 2998 PDA detector
Mobile phase	A: 0.1% TFA in water
	B: Acetonitrile (ACN)
Column	XBridge C18, 4.6 mm × 75 mm, 3.5 μm
UV detector	266 nm
Dilute solvent	ACN
Injection volume	10 μL
Column temperature	30 °C
Flow rate	1.0 mL/min
Run time	13 min
Sample compartment temperature	22 °C
Retention time	4.6 min

Gradient Elution						
Time (min)	0	3	7	11	11.1	13
A (%)	90	60	40	10	90	90
B (%)	10	40	60	90	10	10

General procedure A for esterification of amino acid (for preparation of 3–6b, 8b, 9b, and 12–15b)

To a solution of the starting material amino acid (1.00 equiv) in anhydrous methanol (the concentration of starting material at 0.20 M) was added thionyl chloride (1.10 equiv) at 0 ˚C, and the reaction mixture was stirred at 50–70 ˚C for 16–18 h. After reaction completion, the crude was concentrated under reduced pressure to give the desired intermediate as a hydrochloride salt (1:1).

General procedure B for protection of amine (for preparation of 4c, 15c)

To a solution of the intermediates **4b** and **15b** (1.00 equiv) in dichloromethane (the concentration of the intermediate at 0.30 M) was added methyl chloroformate (1.05 equiv) and sodium bicarbonate (2.20 equiv) at 0 ˚C, and the reaction mixture was stirred at room temperature for 16–18 h. After reaction completion, the crude was diluted with water, extracted with dichloromethane, then the organic layer was concentrated under reduced pressure to give the desired intermediate.

General procedure C for protection of amine (for preparation of 3c, 5c, 6c, 8c, 9c, and 12–14c)

For ethyl chloroformate:

To a solution of the intermediates **3b**, **5b**, **6b**, **8b**, **9b**, and **12–14b** (1.00 equiv) in dichloromethane (the concentration of the intermediate at 0.30 M) was added ethyl chloroformate (1.05 equiv) and pyridine (2.20 equiv) at 0 ˚C, and the reaction mixture was stirred at 0 ˚C for 2–16 h. After reaction completion, the crude was diluted with brine, extracted with dichloromethane, then the organic layer was concentrated under reduced pressure to give the desired intermediate.

For methyl chloroformate:

To a solution of the intermediate **4b** (1.00 equiv) and some case of intermediates **5b**, **6b**, **8b**, **12b and 13b** (1.00 equiv) in dichloromethane (the concentration of the intermediate at at 0.30 M) was added methyl chloroformate (1.05 equiv) and sodium bicarbonate (2.20 equiv) at 0 ˚C, and the reaction mixture was stirred at room temperature for 16–18 h. After reaction completion, the crude was diluted with water, extracted with dichloromethane, then the organic layer was concentrated under reduced pressure to give the desired intermediate.

General procedure D for cyclization (for preparation of 3–6d, 8d, 9d, and 12–15d)

To a solution of the intermediates **3–6c**, **8c**, **9c**, **and 12–15c** (1.00 equiv) in a mixture of acetic acid and

sulfuric acid (ratio: 3 / 1, the concentration of the intermediate at 0.25 M) was added paraformaldehyde (1.10 equiv), and the reaction mixture was stirred at room temperature for 16–18 h. After reaction completion, the crude was neutralized with aqueous 6 N NaOH at ice bath, extracted with brine and dichloromethane, then the organic layer was concentrated under reduced pressure. The residue was purified by Isco Combi-Flash Companion column chromatography or flash column chromatography over silica gel to give the desired intermediate.

General procedure E for deprotection (for preparation of 3–6e, 8e, 9e, and 12–15e)

A mixture of the intermediates **3**–**6d**, **8d**, **9d**, and **12**–**15d** (1.00 equiv) in aqueous 6–12 N hydrogen chloride (the concentration of the intermediate at 0.10 M) was refluxed for 16–48 h. After reaction completion, the obtained precipitate was filtered, and the solid was washed by iced acetone, dried *in vacau* to give the desired intermediate as a hydrochloride salt (1:1).

General procedure F for methyl esterification (for preparation of 5f and 8f)

To a suspension of the intermediates **5e** and **8e** (1.00 equiv) in methanol (the concentration of the intermediate at 0.10 M) was added sulfuric acid (2.00 equiv), and the suspension was refluxed for 16–18 h. After reaction completion, the crude was evaporated under reduced pressure, then the resulted residue was diluted with dichloromethane, washed with saturated aqueous $NaHCO_3$, and the organic layer was concentrated under reduced pressure. The crude product was purified by Isco Combi-Flash Companion column chromatography or flash column chromatography over silica gel to give the desired intermediate.

General procedure G for methyl esterification (for preparation of 3f, 4f, 6f, 7c, 9f, 11b, and 12–15f)

To a solution of the intermediates **3e**, **4e**, **6e**, **7b**, **9e**, **11a**, and **12**–**15e** (1.00 equiv) and triethylamine (1.10 equiv) in anhydrous methanol (the concentration of the intermediate at 0.10 M) under a ice bath was added thionyl chloride (3.50 equiv) at 0 ˚C, then the reaction mixture was stirred at 50 ˚C to reflux for 16–18 h. After reaction completion, the crude was diluted with dichloromethane, extracted with saturated aqueous $NaHCO_3$, and the organic layer was concentrated under reduced pressure. The crude product was purified by Isco Combi-Flash Companion column chromatography or flash column chromatography over silica gel to give the desired intermediate.

General procedure H for amidation of 3-methyl ester (for preparation of 3–6g, 7d, 8g, 9g, 11c, and

12–15g)

A solution of the intermediates **3**–**6f**, **7c**, **8f**, **9f**, **11b**, and **12**–**15f** (1.00 equiv) in 28–30% aqueous ammonium hydroxide (the concentration of the intermediate at 0.10 M) was stirred at room temperature for 16–18 h. After reaction completion, the obtained precipitate was filtered, washed by iced water or iced dichloromethane, and dried *in vacuo* to give the desired intermediate.

General procedure I for methylation of 2-amino of tetrahedroisoquinoline (for preparation of 2b, 3–6h, 7e, 8h, 9h, 10b, 11d, and 12–15h)

A suspension of the intermediates **2a**, **3**–**6g**, **7d**, **8g**, **9g**, **10a**, **11c**, and **12**–**15g** (1.00 equiv) and 37% aqueous formaldehyde (1.50 equiv) in acetonitrile or tetrahydrofuran (the concentration of the intermediate at 0.05 M) was stirred at room temperature for 0.5–1 h, then to the reaction mixture was added sodium triacetoxyborohydride (2.00 equiv) and acetic acid (1.00 equiv). The reaction mixture was stirred at room temperature for 1–16 h. After reaction completion, the crude mixture was diluted with dichloromethane or ethyl acetate, extracted with saturated aqueous $NaHCO_3$, and the organic layer was concentrated under reduced pressure. The crude product was purified by Isco Combi-Flash Companion column chromatography or flash column chromatography over silica gel to give the desired intermediate.

General procedure J for reduction of 3-carboxamide (for preparation of 2c, 3–6i, 7f, 8i, 9i, 10c, 11e, and 12–15i)

To a solution of the intermediates **2b**, **3**–**6h**, **7e**, **8h**, **9h**, **10b**, **11d**, and **12**–**15h** (1.00 equiv) in anhydrous tetrahydrofuran (the concentration of the intermediate at 0.05 M) at 0 ˚C was added lithium aluminum hydride (3.00 equiv, solid or 1–2 M solution in tetrahydrofuran), and the suspension was stirred at 50 ˚C to reflux for 3–72 h. After reaction completion, the crude mixture was quenched with saturated aqueous Na_2SO_4, filtered through a short pad of celite, then the filtrate was extracted with dichloromethane and brine, and the organic layer was concentrated under reduced pressure. The crude product was purified by Isco Combi-Flash Companion column chromatography or flash column chromatography over silica gel to give the desired intermediate.

2-Methyl-1,2,3,4-tetrahydroisoquinoline-3-carboxamide (2b)

The compound was prepared by general procedure I with 1,2,3,4-tetrahydroisoquinoline-3-carboxamide **2a**

(1.50 g, 8.51 mmol), 37% aqueous formaldehyde (2.22 mL, 29.8 mmol) and sodium triacetoxyborohydride (7.22 g, 34.1 mmol) in acetonitrile (100 mL) and tetrahydrofuran (200 mL), stirred at room temperature for 17 h. After workup, the crude product was purified by Isco Combi-Flash Companion column chromatography (0–5% methanol in CH_2Cl_2) to give the desired product 2-methyl-1,2,3,4-tetrahydroisoquinoline-3-carboxamide **2b** (1.39 g, 85.8%). ^{1}H NMR (300 MHz, $CDCl_3$) δ 7.24–7.12 (m, 3H), 7.12–7.05 (m, 1H), 6.94 (br s, 1H), 5.33 (br s, 1H), 3.87 (d, 1H), 3.70 (d, 1H), 3.28 (dd, 1H), 3.15–3.03 (m, 2H), 2.44 (s, 3H).

(2-Methyl-1,2,3,4-tetrahydroisoquinolin-3-yl)methanamine (2c)

The compound was prepared by general procedure J with 2-methyl-1,2,3,4-tetrahydroisoquinoline-3-carboxamide **2b** (500 mg, 2.63 mmol) and lithium aluminum hydride (300 mg, 7.88 mmol) in tetrahydrofuran (30.0 mL) and CH_2Cl_2 (30.0 mL), stirred at 50 °C for 17 h. After workup, the crude product was purified by Isco Combi-Flash Companion column chromatography (0–5% methanol with 1% ammonia hydroxide in CH_2Cl_2) to give the desired product (2-methyl-1,2,3,4-tetrahydroisoquinolin-3-yl)methanamine **2c** (0.240 g, 52%). ^{1}H NMR (400 MHz, $CDCl_3$) δ 7.22–6.95 (m, 4H), 3.85 (d, 1H), 3.70 (d, 1H), 3.02–2.74 (m, 4H), 2.68 (dq, 1H), 2.41 (s, 3H).

Methyl 2-amino-3-(2-bromophenyl)propanoate hydrochloride (1:1) (3b)

The compound was prepared by general procedure A with 2-amino-3-(4-methylphenyl)propanoic acid **3a** (0.300 g, 1.23 mmol) and thionyl chloride (0.178 mL, 2.46 mmol) in anhydrous methanol (10.0 mL), stirred at 50 °C for 17 h. The desired product methyl 2-amino-3-(2-bromophenyl)propanoate hydrochloride (1:1) **3b** was obtained as a white solid (0.280 g, 78%). ^{1}H NMR (400 MHz, DMSO-d_6) δ 8.60 (br s, 2H), 7.63 (dd, 1H), 7.41–7.34 (m, 2H), 7.30–7.20 (m, 1H), 4.19 (br s, 1H), 3.61 (s, 3H), 3.23 (d, 2H).

Methyl 3-(2-bromophenyl)-2-[(ethoxycarbonyl)amino]propanoate (3c)

The compound was prepared by general procedure C with methyl 2-amino-3-(2-bromophenyl)propanoate hydrochloride (1:1) **3b** (0.280 g, 0.956 mmol), ethyl chloroformate (0.141 mL, 1.00 mmol) and pyridine (0.169 mL, 2.10 mmol) in dichloromethane (5.0 mL), stirred at 0 °C for 2 h. After workup, the crude product was purified by Isco Combi-Flash Companion column chromatography (0–20% ethyl acetate in n-hexane) to give the desired product methyl 3-(2-bromophenyl)-2-[(ethoxycarbonyl)amino]propanoate **3c** (0.230 g,

73%). ^{1}H NMR (400 MHz, DMSO-d_6) δ 7.71 (d, 1H), 7.59 (d, 1H), 7.41–7.27 (m, 2H), 7.24–7.14 (m, 1H), 4.41–4.25 (m, 1H), 3.98–3.83 (m, 2H), 3.62 (s, 3H), 3.19 (dd, 1H), 2.94 (dd, 1H), 1.10 (t, 3H).

2-Ethyl 3-methyl 5-bromo-3,4-dihydroisoquinoline-2,3(1*H*)-dicarboxylate (3d)

The compound was prepared by general procedure D with methyl 3-(2-bromophenyl)-2-((ethoxycarbonyl) amino)propanoate **3c** (0.230 g, 0.697 mmol) and paraformaldehyde (23.0 g, 0.766 mmol) in a mixture of acetic acid (3.0 mL) and sulfuric acid (1.0 mL), stirred at room temperature for 17 h. After workup, the crude product was purified by Isco Combi-Flash Companion column chromatography (0–30% ethyl acetate in n-hexane) to give the desired product 2-ethyl 3-methyl 5-bromo-3,4-dihydroisoquinoline-2,3(1*H*)-dicarboxylate **3d** as a colorless liquid (95.6 mg, 40%). ^{1}H NMR (400 MHz, DMSO-d_6) δ 7.51 (d, 1H), 7.35–7.22 (m, 1H), 7.16 (t, 1H), 5.15–4.94 (m, 1H), 4.67 (dd, 1H), 4.45 (dd, 1H), 4.23–4.06 (m, 2H), 3.57 (s, 3H), 3.43–3.27 (m overlapped with H_2O, 1H), 3.19–2.99 (m, 1H), 1.18 (t, 1.5H), 1.17 (t, 1.5H).

5-Bromo-1,2,3,4-tetrahydroisoquinoline-3-carboxylic acid hydrochloride (1:1) (3e)

The compound was prepared by general procedure E with 2-ethyl 3-methyl 5-bromo-3,4-dihydroisoquinoline-2,3(1*H*)-dicarboxylate **3d** (2.48 g, 7.25 mmol) in aqueous 12 N hydrogen chloride (70.0 mL), refluxed for 17 h. The desired product 5-bromo-1,2,3,4-tetrahydroisoquinoline-3-carboxylic acid hydrochloride (1:1) **3e** was obtained as a white solid (1.79 g, 84%). ^{1}H NMR (400 MHz, DMSO-d_6) δ 9.93 (br s, 2H), 7.61 (dd, 1H), 7.32 (d, 1H), 7.24 (t, 1H), 4.46 (dd, 1H), 4.38 (d, 1H), 4.34 (d, 1H), 3.27 (dd, 1H), 2.96 (dd, 1H).

Methyl 5-bromo-1,2,3,4-tetrahydroisoquinoline-3-carboxylate (3f)

The compound was prepared by general procedure G with 5-bromo-1,2,3,4-tetrahydroisoquinoline-3-carboxylic acid hydrochloride (1:1) **3e** (1.79 g, 6.12 mmol), thionyl chloride (1.55 mL, 21.4 mmol) and triethylamine (0.940 mL, 6.73 mmol) in anhydrous methanol (25.0 mL), stirred at 50 ^{o}C for 17 h. After workup, the crude mixture was concentrated to give the desired product methyl 5-bromo-1,2,3,4-tetrahydroisoquinoline-3-carboxylate **3f** (1.63 g, 98%). ^{1}H NMR (400 MHz, DMSO-d_6) δ 7.43 (t, 1H), 7.14–7.03 (m, 2H), 3.93 (d, 1H), 3.86 (d, 1H), 3.72 (dd, 1H), 3.68 (s, 3H), 2.90 (dd, 1H), 2.67 (dd, 1H).

5-Bromo-1,2,3,4-tetrahydroisoquinoline-3-carboxamide (3g)

The compound was prepared by general procedure H with methyl 5-bromo-1,2,3,4-tetrahydroisoquinoline-

3-carboxylate **3f** (1.63 g, 6.03 mmol) in 28–30% aqueous ammonium hydroxide (60.0 mL), stirred at room temperature for 17 h, to give the desired product 5-bromo-1,2,3,4-tetrahydroisoquinoline-3-carboxamide **3g** (1.20 g, 78%). ^{1}H NMR (400 MHz, DMSO-d_6) δ 7.43 (t, 1H), 7.37 (br s, 1H), 7.14 (br s, 1H), 7.10–7.05 (m, 2H), 3.91 (d, 1H), 3.85 (d, 1H), 3.39 (dd, 1H), 2.87 (dd, 1H), 2.56 (dd, 1H).

5-Bromo-2-methyl-1,2,3,4-tetrahydroisoquinoline-3-carboxamide (3h)

The compound was prepared by general procedure I with 5-bromo-1,2,3,4-tetrahydroisoquinoline-3-carboxamide **3g** (1.20 g, 4.70 mmol), 37% aqueous formaldehyde (0.530 mL, 7.06 mmol), sodium triacetoxyborohydride (1.99 g, 9.41 mmol) and acetic acid (0.270 mL, 4.70 mmol) in acetonitrile (25.0 mL) and tetrahydrofuran (25.0 mL), stirred at room temperature for 2 h. After workup, the crude product was purified by Isco Combi-Flash Companion column chromatography (0–5% methanol in CH_2Cl_2) to give the desired product 5-bromo-2-methyl-1,2,3,4-tetrahydroisoquinoline-3-carboxamide **3h** (0.340 g, 27%). ^{1}H NMR (400 MHz, DMSO-d_6) δ 7.49–7.41 (m, 2H), 7.15 (br s, 1H), 7.11–7.07 (m, 2H), 3.87 (d, 1H), 3.53 (d, 1H), 3.15 (dd, 1H), 2.85–2.76 (m, 2H), 2.30 (s, 3H).

(5-Bromo-2-methyl-1,2,3,4-tetrahydroisoquinolin-3-yl)methanamine (3i)

The compound was prepared by general procedure J with 5-bromo-2-methyl-1,2,3,4-tetrahydroisoquinoline-3-carboxamide **3h** (0.570 g, 2.11 mmol) and 1M lithium aluminum hydride in THF (6.40 mL, 6.35 mmol) in tetrahydrofuran (30.0 mL) and CH_2Cl_2 (30.0 mL), stirred at 60 °C for 17 h. After workup, the crude product was purified by Isco Combi-Flash Companion column chromatography (0–5% methanol with 1% ammonia hydroxide in CH_2Cl_2) to give the desired product (5-bromo-2-methyl-1,2,3,4-tetrahydroisoquinolin-3-yl)methanamine **3i** (0.320 g, 59%). ^{1}H NMR (400 MHz, DMSO-d_6) δ 7.46–7.38 (m, 1H), 7.08–7.05 (m, 2H), 3.76 (d, 1H), 3.52 (d, 1H), 2.73 (dd, 1H), 2.68–2.60 (m, 3H), 2.53–2.43 (m overlapped with d_6-DMSO, 1H), 2.26 (s, 3H).

Methyl 2-amino-3-(3-methylphenyl)propanoate hydrochloride (1:1) (4b)

The compound was prepared by general procedure A with 2-amino-3-(3-methylphenyl)propanoic acid **4a** (8.00 g, 44.6 mmol) and thionyl chloride (3.57 mL, 49.1 mmol) in anhydrous methanol (120 mL), refluxed for 18 h. The desired product methyl 2-amino-3-(3-methylphenyl)propanoate hydrochloride (1:1) **4b** was obtained as a white solid (10.25 g, quantitative yield). ^{1}H NMR (400 MHz, CD_3OD) δ 7.25 (t, 1H), 7.14 (d,

1H), 7.09 (s, 1H), 7.04 (d, 1H), 4.30 (t, 1H), 3.81 (s, 3H), 3.23 (dd, 1H), 3.12 (dd, 1H), 2.35 (s, 3H).

Methyl 2-[(ethoxycarbonyl)amino]-3-(3-methylphenyl)propanoate (4c)

The compound was prepared by general procedure B with methyl 2-amino-3-(3-methylphenyl)propanoate hydrochloride (1:1) **4b** (10.2 g, 44.6 mmol), methyl chloroformate (4.49 mL, 46.9 mmol) and pyridine (7.91 mL, 98.2 mmol) in dichloromethane (153 mL), stirred at room temperature for 18 h. The desired product methyl 2-[(ethoxycarbonyl)amino]-3-(3-methylphenyl)propanoate **4c** was obtained as a brown oil (8.40 g, 71%). ^{1}H NMR (600 MHz, DMSO-d_6) δ 7.62 (d, 1H), 7.16 (t, 1H), 7.07–6.99 (m, 3H), 4.19 (ddd, 1H), 3.97–3.88 (m, 2H), 3.61 (s, 3H), 2.96 (dd, 1H), 2.80 (dd, 1H), 2.26 (s, 3H), 1.11 (t, 3H).

2-Ethyl 3-methyl 6-methyl-3,4-dihydroisoquinoline-2,3(1*H*)-dicarboxylate and 2-ethyl 3-methyl 8-methyl-3,4-dihydroisoquinoline-2,3(1*H*)-dicarboxylate (4d)

The compound was prepared by general procedure D with methyl 2-((ethoxycarbonyl)amino)-3-(3-methylphenyl)propanoate **4c** (8.40 g, 31.7 mmol) and paraformaldehyde (1.05 g, 34.8 mmol) in a mixture of acetic acid (48.0 mL) and sulfuric acid (16.0 mL), stirred at room temperature for 18 h. The inseparable mixture of 2-ethyl 3-methyl 6-methyl-3,4-dihydroisoquinoline-2,3(1*H*)-dicarboxylate and 2-ethyl 3-methyl 8-methyl-3,4-dihydroisoquinoline-2,3(1*H*)-dicarboxylate **4d** was obtained as a colorless oil (7.22 g, 82%). ^{1}H NMR (600 MHz, DMSO-d_6) δ 7.12–6.98 (m, 3H), 5.07–4.83 (m, 1H), 4.64–4.51 (m, 1H), 4.47–4.24 (m, 1H), 4.20–4.00 (m, 2H), 3.58–3.52 (m, 3H), 3.19–3.02 (m, 2H), 2.25 (s, 2H), 2.22–02.18 (m, 1H), 1.28–1.22 (m, 1.5H), 1.21–1.12 (m, 1.5H).

6-Methyl-1,2,3,4-tetrahydroisoquinoline-3-carboxylic acid hydrochloride (1:1) and 8-methyl-1,2,3,4-tetrahydroisoquinoline-3-carboxylic acid hydrochloride (1:1) (4e)

The compound was prepared by general procedure E with 2-ethyl 3-methyl 6-methyl-3,4-dihydroisoquinoline-2,3(1*H*)-dicarboxylate and 2-ethyl 3-methyl 8-methyl-3,4-dihydroisoquinoline-2,3(1H)-dicarboxylate **4d** (6.77 g, 13.6 mmol) in aqueous 8 N hydrogen chloride (98.0 mL), refluxed for 18 h. The inseparable mixture of 6-methyl-1,2,3,4-tetrahydroisoquinoline-3-carboxylic acid hydrochloride (1:1) and 8-methyl-1,2,3,4-tetrahydroisoquinoline-3-carboxylic acid (1:1) **4e** was obtained as a white solid (the ratio is 85:15, 3.45 g, 62%). The ^{1}H NMR spectrum of 6-methyl-1,2,3,4-tetrahydroisoquinoline-3-carboxylic acid

hydrochloride (1:1) is listed. ^{1}H NMR (400 MHz, CD_3OD) δ 7.16–7.10 (m, 3H), 4.48–4.27 (m, 3H), 3.43 (dd, 1H), 3.20 (dd, 1H), 2.33 (s, 3H).

Methyl 6-methyl-1,2,3,4-tetrahydroisoquinoline-3-carboxylate and methyl 8-methyl-1,2,3,4-tetrahydroisoquinoline-3-carboxylate (4f)

The compound was prepared by general procedure G with 6-methyl-1,2,3,4-tetrahydroisoquinoline-3-carboxylic acid hydrochloride (1:1) and 8-methyl-1,2,3,4-tetrahydroisoquinoline-3-carboxylic acid (1:1) **4e** (1.00 g, 4.39 mmol), thionyl chloride (1.12 mL, 15.4 mmol) and triethylamine (1.52 mL, 4.83 mmol) in anhydrous methanol (45.0 mL), refluxed for 18 h. After workup, the crude product was purified by flash column chromatography (SiO_2, dichloromethane/methanol = 99.5/0.5) to give an inseparable mixture of methyl 6-methyl-1,2,3,4-tetrahydroisoquinoline-3-carboxylate and methyl 8-methyl-1,2,3,4-tetrahydroisoquinoline-3-carboxylate **4f** as a yellow oil (the ratio is 82:18, 1.42 g, quantitative yield). The ^{1}H NMR spectrum of methyl 6-methyl-1,2,3,4-tetrahydroisoquinoline-3-carboxylate is listed. ^{1}H NMR (400 MHz, $CDCl_3$) δ 6.98–6.90 (m, 3H), 4.09 (d, 1H), 4.03 (d, 1H), 3.77 (s, 3H), 3.72 (dd, 1H), 3.03 (dd, 1H), 2.91 (dd, 1H), 2.30 (s, 3H).

6-Methyl-1,2,3,4-tetrahydroisoquinoline-3-carboxamide and 8-methyl-1,2,3,4-tetrahydroisoquinoline-3-carboxamide (4g)

The compound was prepared by general procedure H with methyl 6-methyl-1,2,3,4-tetrahydroisoquinoline-3-carboxylate and methyl 8-methyl-1,2,3,4-tetrahydroisoquinoline-3-carboxylate **4f** (1.42 g, 6.92 mmol) in 28–30% aqueous ammonium hydroxide (138 mL), stirred at room temperature for 18 h. The inseparable mixture of 6-methyl-1,2,3,4-tetrahydroisoquinoline-3-carboxamide and 8-methyl-1,2,3,4-tetrahydroisoquinoline-3-carboxamide **4g** was obtained as a white solid (the ratio is 85:15, 1.32 g, quantitative yield). The ^{1}H NMR spectrum of 6-methyl-1,2,3,4-tetrahydroisoquinoline-3-carboxamide is listed.^{1}H NMR (400 MHz, CD_3OD) δ 6.97–6.91 (m, 3H), 4.01 (d, 1H), 3.95 (d, 1H), 3.54 (dd, 1H), 2.99 (dd, 1H), 2.85 (dd, 1H), 2.27 (s, 3H).

2,6-Dimethyl-1,2,3,4-tetrahydroisoquinoline-3-carboxamide and 2,8-dimethyl-1,2,3,4-tetrahydroisoquinoline-3-carboxamide (4h)

The compound was prepared by general procedure I with 6-methyl-1,2,3,4-tetrahydroisoquinoline-3-carboxamide and 8-methyl-1,2,3,4-tetrahydroisoquinoline-3-carboxamide **4g** (1.32 g, 6.94 mmol), 37% aqueous formaldehyde (0.84 mL, 10.4 mmol), sodium triacetoxyborohydride (2.94 g, 13.9 mmol) and acetic acid (400 μL, 6.94 mmol) in tetrahydrofuran (35.0 mL), stirred at room temperature for 18 h. The inseparable mixture of 2,6-dimethyl-1,2,3,4-tetrahydroisoquinoline-3-carboxamide and 2,8-dimethyl-1,2,3,4-tetrahydroisoquinoline-3-carboxamide **4h** was obtained as a white solid (the ratio is 86:14, 1.32 g, 93%). The ^{1}H NMR spectrum of 2,6-dimethyl-1,2,3,4-tetrahydroisoquinoline-3-carboxamide is listed. ^{1}H NMR (400 MHz, CD_3OD) δ 6.96–6.92 (m, 3H), 3.91 (d, 1H), 3.52 (d, 1H), 3.12 (dd, 1H), 3.05 (dd, 1H), 2.94 (dd, 1H), 2.43 (s, 3H), 2.27 (s, 3H).

(2,6-Dimethyl-1,2,3,4-tetrahydroisoquinolin-3-yl)methanamine and (2,8-dimethyl-1,2,3,4-tetrahydroisoquinolin-3-yl)methanamine (4i)

The compound was prepared by general procedure J with 2,6-dimethyl-1,2,3,4-tetrahydroisoquinoline-3-carboxamide and 2,8-dimethyl-1,2,3,4-tetrahydroisoquinoline-3-carboxamide **4h** (200 mg, 0.979 mmol) and 1M lithium aluminum hydride in THF (2.94 mL, 2.94 mmol) in tetrahydrofuran (20.0 mL), stirred at 60 °C for 18 h . After workup, the crude product was purified by flash column chromatography (SiO_2, dichloromethane/methanol/tetrahydrofuran = 90/10/1 to 80/20/1) to give an inseparable mixture of (2,6-dimethyl-1,2,3,4-tetrahydroisoquinolin-3-yl)methanamine and (2,8-dimethyl-1,2,3,4-tetrahydroisoquinolin-3-yl)methanamine **4i** as a yellow oil (the ratio is 89:11, 138 mg, 74%). The ^{1}H NMR spectrum of (2,6-dimethyl-1,2,3,4-tetrahydroisoquinolin-3-yl)methanamine is listed. ^{1}H NMR (400 MHz, $CDCl_3$) δ 6.96–6.89 (m, 3H), 3.82 (d, 1H), 3.67 (d, 1H), 2.89–2.79 (m, 2H), 2.76–2.64 (m, 3H), 2.39 (s, 3H), 2.29 (s, 3H).

Methyl 3-fluorophenylalaninate hydrochloride (1:1) (5b)

The compound was prepared by general procedure A with 3-fluoro-dl-phenylalanine **5a** (0.500 g, 2.73

mmol) and thionyl chloride (0.218 mL, 3.00 mmol) in anhydrous methanol (4mL), refluxed for 16 h. The desired product methyl 3-fluorophenylalaninate hydrochloride (1:1) **5b** was obtained as a brown solid (0.538 g, quantitative yield). ^{1}H NMR (300 MHz, CD_3OD) δ 7.44–7.35 (m, 1H), 7.12–7.03 (m, 3H), 4.37 (dd, 1H), 3.81 (s, 3H), 3.34–3.16 (m, 2H).

Methyl *N*-(ethoxycarbonyl)-3-fluorophenylalaninate (5c)

The compound was prepared by general procedure C with methyl 3-fluorophenylalaninate hydrochloride (1:1) **5b** (0.538 g, 2.73 mmol), ethyl chloroformate (0.286 mL, 3.00 mmol) and pyridine (0.484 mL, 6.01 mmol) in dichloromethane (8.9 mL), stirred at room temperature for 2 h. The desired product methyl *N*-(ethoxycarbonyl)-3-fluorophenylalaninate **5c** was obtained as a brown liquid (0.654 g, 89%). ^{1}H NMR (300 MHz, $CDCl_3$) δ 7.29–7.20 (m, overlapped $CDCl_3$, 1H), 6.97–6.88 (m, 2H), 6.83 (d, 1H), 5.18 (br d, 1H), 4.63 (br q, 1H), 4.10 (q, 2H), 3.72 (s, 3H), 3.17–3.00 (m, 2H), 1.22 (t, 3H).

2-Ethyl 3-methyl 6-fluoro-3,4-dihydroisoquinoline-2,3(1*H*)-dicarboxylate and 2-ethyl 3-methyl 8-fluoro-3,4-dihydroisoquinoline-2,3(1*H*)-dicarboxylate (5d)

The compound was prepared by general procedure D with methyl *N*-(ethoxycarbonyl)-3-fluorophenylalaninate **5c** (0.654 g, 2.43 mmol) and paraformaldehyde (76.5 mg, 2.55 mmol) in a mixture of acetic acid (2.35 mL) and sulfuric acid (0.783 mL), stirred at room temperature for 16 h. The inseparable mixture of 2-ethyl 3-methyl 6-fluoro-3,4-dihydroisoquinoline-2,3(1*H*)-dicarboxylate and 2-ethyl 3-methyl 8-fluoro-3,4-dihydroisoquinoline-2,3(1*H*)-dicarboxylate **5d** was obtained as a brown liquid (0.683 g, quantitative yield). ^{1}H NMR (300 MHz, $CDCl_3$) δ 7.13–7.02 (m, 1H), 6.93–6.82 (m, 2H), 5.16 (dd, 0.5H), 4.93 (t, 0.5H), 4.72 (dd, 1H), 4.50 (dd, 1H), 4.26–4.14 (m, 2H), 3.62 (s, 3H), 3.27–3.09 (m, 2H), 1.32 (t, 1.5H), 1.24 (t, 1.5H).

6-Fluoro-1,2,3,4-tetrahydroisoquinoline-3-carboxylic acid hydrochloride (1:1) (5e)

The compound was prepared by general procedure E with 2-ethyl 3-methyl 6-fluoro-3,4-dihydroisoquinoline-2,3(1*H*)-dicarboxylate and 2-ethyl 3-methyl 8-fluoro-3,4-dihydroisoquinoline-2,3(1*H*)-dicarboxylate **5d** (2.27 g, 8.07 mmol) in aqueous 6 N hydrogen chloride (32.7 mL), refluxed for 48 h. The desired product 6-fluoro-1,2,3,4-tetrahydroisoquinoline-3-carboxylic acid hydrochloride (1:1) **5e** was obtained as a brown

solid (1.74 g, 93%). ^{1}H NMR (300 MHz, CD_3OD) δ 7.29 (dd, 1H), 7.12–7.03 (m, 2H), 4.45–4.35 (m, 3H), 3.50 (dd, 1H), 3.24 (dd, 1H).

Methyl 6-fluoro-1,2,3,4-tetrahydroisoquinoline-3-carboxylate (5f)

The compound was prepared by general procedure F with 6-fluoro-1,2,3,4-tetrahydroisoquinoline-3-carboxylic acid hydrochloride (1:1) **5e** (1.74 g, 7.51 mmol) and sulfuric acid (0.802 mL, 15.0 mmol) in anhydrous methanol (39.8 mL), refluxed for 16 h. The desired product methyl 6-fluoro-1,2,3,4-tetrahydroisoquinoline-3-carboxylate **5f** was obtained as a brown liquid (1.41 g, 90%). ^{1}H NMR (300 MHz, $CDCl_3$) δ 6.97 (dd, 1H), 6.87–6.77 (m, 2H), 4.12–3.96 (m, 2H), 3.76 (s, 3H), 3.72 (dd, 1H), 3.06 (dd, 1H), 2.94 (dd, 1H).

6-Fluoro-1,2,3,4-tetrahydroisoquinoline-3-carboxamide (5g)

The compound was prepared by general procedure H with methyl 6-fluoro-1,2,3,4-tetrahydroisoquinoline-3-carboxylate **5f** (1.36 g, 6.50 mmol) in 28–30% aqueous ammonium hydroxide (18.1 mL, 130 mmol), stirred at room temperature for 16 h. The desired product 6-fluoro-1,2,3,4-tetrahydroisoquinoline-3-carboxamide **5g** was obtained as a white solid (1.02 g, 81%). ^{1}H NMR (300 MHz, CD_3OD) δ 7.11–7.03 (m, 1H), 6.92–6.83 (m, 2H), 4.03 (d, 1H), 3.94 (d, 1H), 3.60–3.52 (m, 1H), 3.09–2.98 (m, 1H), 2.94–2.82 (m, 1H).

6-Fluoro-2-methyl-1,2,3,4-tetrahydroisoquinoline-3-carboxamide (5h)

The compound was prepared by general procedure I with 6-fluoro-1,2,3,4-tetrahydroisoquinoline-3-carboxamide **5g** (1.02 g, 5.26 mmol), 37% aqueous formaldehyde (1.38 mL, 18.4 mmol) and sodium triacetoxyborohydride (4.46 g, 21.1 mmol) in acetonitrile (76.7 mL), stirred at room temperature for 16 h. The desired product 6-fluoro-2-methyl-1,2,3,4-tetrahydroisoquinoline-3-carboxamide **5h** was obtained as a white solid (0.718 g, 66%). ^{1}H NMR (300 MHz, CD_3OD) δ 7.13–7.03 (m, 1H), 6.93–6.82 (m, 2H), 4.00–3.88 (m, 1H), 3.58–3.47 (m, 1H), 3.20–2.92 (m, 3H), 2.48–2.40 (m, 3H).

1-(6-Fluoro-2-methyl-1,2,3,4-tetrahydroisoquinolin-3-yl)methanamine (5i)

The compound was prepared by general procedure J with 6-fluoro-2-methyl-1,2,3,4-tetrahydroisoquinoline-3-carboxamide **5h** (0.300 g, 1.44 mmol) and lithium aluminum hydride (0.164 g, 4.32 mmol) in anhydrous tetrahydrofuran (28.7 mL), refluxed for 16 h. After workup, the crude product was concentrated to give the desired product

1-(6-fluoro-2-methyl-1,2,3,4-tetrahydroisoquinolin-3-yl)methanamine **5i** as a yellow liquid (0.224 g, 80%). ^{1}H NMR (300 MHz, $CDCl_3$) δ 6.97 (dd, 1H), 6.86–6.75 (m, 2H), 3.85–3.59 (m, 2H), 3.00–2.64 (m, 5H), 2.44–2.30 (br s, 3H).

Methyl 2-amino-3-(3-chlorophenyl)propanoate hydrochloride (1:1) (6b)

The compound was prepared by general procedure A with 3-chlorophenylalanine **6a** (4.00 g, 20.0 mmol) and thionyl chloride (1.61 mL, 22.0 mmol) in anhydrous methanol (24.8 mL), refluxed for 16 h. The desired product methyl 2-amino-3-(3-chlorophenyl)propanoate hydrochloride (1:1) **6b** was obtained as a brown solid (4.25 g, 85%). ^{1}H NMR (300 MHz, CD_3OD) δ 7.40–7.32 (m, 3H), 7.21 (dt, 1H), 4.36 (t, 1H), 3.81 (s, 3H), 3.28 (dd, overlapped with CD_3OD, 1H), 3.18 (dd, 1H).

Methyl 3-(3-chlorophenyl)-2-[(ethoxycarbonyl)amino]propanoate (6c)

The compound was prepared by general procedure C with methyl 2-amino-3-(3-chlorophenyl)propanoate hydrochloride (1:1) **6b** (4.25 g, 17.0 mmol), ethyl chloroformate (1.78 mL, 18.7 mmol) and pyridine (3.01 mL, 37.4 mmol) in dichloromethane (64.5 mL), stirred at room temperature for 2 h. The desired product methyl 3-(3-chlorophenyl)-2-[(ethoxycarbonyl)amino]propanoate **6c** was obtained as a brown liquid (4.74 g, 98%). ^{1}H NMR (300 MHz, $CDCl_3$) δ 7.23–7.20 (m, 2H), 7.11 (s, 1H), 7.02–6.98 (m, 1H), 5.19 (br d, 1H), 4.62 (br q, 1H), 4.10 (q, 2H), 3.72 (s, 3H), 3.15–2.97 (m, 2H), 1.22 (t, 3H).

2-Ethyl 3-methyl 6-chloro-3,4-dihydroisoquinoline-2,3(1*H*)-dicarboxylate and 2-ethyl 3-methyl 8-chloro-3,4-dihydroisoquinoline-2,3(1*H*)-dicarboxylate (6d)

The compound was prepared by general procedure D with methyl 3-(3-chlorophenyl)-2-[(ethoxycarbonyl)amino]propanoate **6c** (4.70 g, 16.4 mmol) and paraformaldehyde (0.518 g, 17.3 mmol) in a mixture of acetic acid (15.9 mL) and sulfuric acid (5.30 mL), stirred at room temperature for 16 h. The inseparable mixture of 2-ethyl 3-methyl 6-chloro-3,4-dihydroisoquinoline-2,3(1*H*)-dicarboxylate and 2-ethyl 3-methyl 8-chloro-3,4-dihydroisoquinoline-2,3(1*H*)-dicarboxylate **6d** was obtained as a colorless liquid (3.55 g, 73%). ^{1}H NMR (300 MHz, $CDCl_3$) δ 7.20–7.00 (m, 3H), 5.18 (dd, 0.7H), 4.95 (dd, 0.3H), 4.74 (dd, 1H), 4.50 (dd, 1H), 4.28–4.15 (m, 2H), 3.63 (s, 3H), 3.28–3.07 (m, 2H), 1.36–1.21 (m, 3H).

6-Chloro-1,2,3,4-tetrahydroisoquinoline-3-carboxylic acid hydrochloride (1:1) and 8-chloro-1,2,3,4-tetrahydroisoquinoline-3-carboxylic acid hydrochloride (1:1) (6e)

The compound was prepared by general procedure E with 2-ethyl 3-methyl -chloro-3,4-dihydroisoquinoline-2,3(1*H*)-dicarboxylate and 2-ethyl 3-methyl 8-chloro-3,4-dihydroisoquinoline-2,3(1*H*)-dicarboxylate **6d** (3.55 g, 11.9 mmol) in aqueous 8 N hydrogen chloride (119 mL), refluxed for 48 h. The inseparable mixture of 6-chloro-1,2,3,4-tetrahydroisoquinoline-3-carboxylic acid hydrochloride (1:1) and 8-chloro-1,2,3,4-tetrahydroisoquinoline-3-carboxylic acid hydrochloride (1:1) **6e** was obtained as a white solid (the ratio is 72:28, 2.26 g, 76%). The spectrum of 6-chloro-1,2,3,4-tetrahydroisoquinoline-3-carboxylic acid hydrochloride (1:1) is listed. ^{1}H NMR (300 MHz, CD_3OD) δ 7.36 (s, 1H), 7.32 (dd, 1H), 7.26 (d, 1H), 4.50–4.37 (m, 3H), 3.49 (dd, 1H), 3.23 (dd, 1H).

Methyl 6-chloro-1,2,3,4-tetrahydroisoquinoline-3-carboxylate (6f)

The compound was prepared by general procedure G with 6-chloro-1,2,3,4-tetrahydroisoquinoline-3-carboxylic acid hydrochloride (1:1) and 8-chloro-1,2,3,4-tetrahydroisoquinoline-3-carboxylic acid hydrochloride (1:1) **6e** (1.97 g, 7.94 mmol), thionyl chloride (2.02 mL, 27.8 mmol) and triethylamine (1.22 mL, 8.73 mmol) in anhydrous methanol (79.0 mL), stirred at 50 °C for 18 h. After workup, the crude product was purified by flash column chromatography (SiO_2, 3% methanol in dichloromethane) to give the desired product methyl 6-chloro-1,2,3,4-tetrahydroisoquinoline-3-carboxylate **6f** as a colorless oil (1.42 mg, 73%). ^{1}H NMR (300 MHz, DMSO-d_6) δ 7.20 (s, 1H), 7.16 (dd, 1H), 7.06 (d, 1H), 3.98–3.78 (m, 2H), 3.70–3.63 (m, 1H), 3.66 (s, 3H), 2.96 (dd, 1H), 2.88–2.73 (m, 2H).

6-Chloro-1,2,3,4-tetrahydroisoquinoline-3-carboxamide (6g)

The compound was prepared by general procedure H with methyl 6-chloro-1,2,3,4-tetrahydroisoquinoline-3-carboxylate **6f** (775 mg, 2.96 mmol) in 28–30% aqueous ammonium hydroxide (30.0 mL), stirred at room temperature for 18 h. The desired product 6-chloro-1,2,3,4-tetrahydroisoquinoline-3-carboxamide **6g** was obtained as a white solid (908 mg, 80%). ^{1}H NMR (300 MHz, DMSO-d_6) δ 7.33 (br s, 1H), 7.18 (s, 1H), 7.18–7.01 (m, 3H), 3.95–3.76 (m, 2H), 3.40–3.29

(m overlapped with H_2O, 1H), 2.89 (dd, 1H), 2.71 (dd, 1H), 2.60 (br s, 1H).

6-Chloro-2-methyl-1,2,3,4-tetrahydroisoquinoline-3-carboxamide (6h)

The compound was prepared by general procedure I with 6-chloro-1,2,3,4-tetrahydroisoquinoline-3-carboxamide **6g** (908 mg, 4.31 mmol), 37% aqueous formaldehyde (1.22 mL, 15.1 mmol), sodium triacetoxyborohydride (3.65 g, 17.3 mmol) and acetic acid (0.517 mL, 8.62 mmol) in acetonitrile (21.5 mL) and tetrahydrofuran (21.5 mL), stirred at room temperature for 2 h. After workup, the crude product was purified by flash column chromatography (SiO_2, 3–5% methanol in dichloromethane) to give the desired product 6-chloro-2-methyl-1,2,3,4-tetrahydroisoquinoline-3-carboxamide **6h** as a white solid (936 mg, 97%). ^1H NMR (300 MHz, DMSO-d_6) δ 7.40 (br s, 1H), 7.20 (s, 1H), 7.16 (dd, 1H), 7.14–7.04 (m, 2H), 3.86 (d, 1H), 3.47 (d, 1H), 3.08 (dd, 1H), 2.99–2.80 (m, 2H), 2.31 (s, 3H).

(6-Chloro-2-methyl-1,2,3,4-tetrahydroisoquinolin-3-yl)methanamine (6i)

The compound was prepared by general procedure J with 6-chloro-2-methyl-1,2,3,4-tetrahydroisoquinoline-3-carboxamide **6h** (0.355 g, 1.70 mmol) and lithium aluminum hydride (50.7 mg, 1.34 mmol) in anhydrous tetrahydrofuran (8.80 mL), refluxed for 16 h. After workup, the crude product was concentrated to give the desired product (6-chloro-2-methyl-1,2,3,4-tetrahydroisoquinolin-3-yl)methanamine **6i** as a yellow liquid (72.0 mg, 77%). ^1H NMR (300 MHz, $CDCl_3$) δ 7.09–7.03 (m, 2H), 6.92 (d, 1H), 3.77 (d, 1H), 3.61 (d, 1H), 2.80 (dd, 2H), 2.75–2.60 (m, 3H), 2.35 (s, 3H).

7-Methyl-1,2,3,4-tetrahydroisoquinoline-3-carboxylic acid (7b)

A mixture solution of 2-amino-3-(4-methylphenyl)propanoic acid **7a** (0.500 g, 2.79 mmol) in 48% aqueous hydrobromic acid (5 mL) at room temperature was added 37% aqueous formaldehyde (1.68 mL, 22.3 mmol). The reaction mixture was stirred at 75 ˚C for 5 h. After reaction completion, the crude was cooled down to 0 ˚C, filtered and washed with dichloromethane, dried *in vacau* to give the desired intermediate (3*R*)-1,2,3,4-tetrahydroisoquinoline-3-carboxylic acid hydrochloride (1:1) **7b** as a white solid (536 mg, 99%). ^1H NMR (400 MHz, DMSO-d_6) δ 9.51 (br s, 2H), 7.27–6.93 (m, 3H), 4.38 (dd, 1H), 4.32–4.24 (m, 2H), 3.29–3.19 (m, 1H), 3.11–2.96 (m, 1H), 2.27 (s, 3H).

Methyl 7-methyl-1,2,3,4-tetrahydroisoquinoline-3-carboxylate (7c)

The compound was prepared by general procedure G with (3*R*)-1,2,3,4-tetrahydroisoquinoline-3-carboxylic acid hydrochloride (1:1) **7b** (0.536 g, 2.80 mmol) and thionyl chloride (1.73 mL, 23.8 mmol) in anhydrous methanol (30.0 mL), stirred at 50 °C for overnight. The desired product methyl 7-methyl-1,2,3,4-tetrahydroisoquinoline-3-carboxylate **7c** was obtained as a white solid (0.542 g, 94%). ^{1}H NMR (400 MHz, DMSO-d_6) δ 9.84 (s, 1H), 7.21–7.00 (m, 3H), 4.56 (dd, 1H), 4.34–4.23 (m, 2H), 3.82 (s, 3H), 3.26 (dd, 1H), 3.08 (dd, 1H), 2.27 (s, 3H).

7-Methyl-1,2,3,4-tetrahydroisoquinoline-3-carboxamide (7d)

The compound was prepared by general procedure H with methyl 7-methyl-1,2,3,4-tetrahydroisoquinoline-3-carboxylate **7c** (0.542 g, 2.64 mmol) in 28–30% aqueous ammonium hydroxide (18.3 mL), stirred at room temperature for 17 h. The desired product 7-methyl-1,2,3,4-tetrahydroisoquinoline-3-carboxamide **7d** was obtained as a white solid (0.287 g, 57%). ^{1}H NMR (400 MHz, DMSO-d_6) δ 7.31 (br s, 1H), 7.05 (br s, 1H), 7.00–6.87 (m, 2H), 6.83 (s, 1H), 3.96–3.75 (m, 2H), 3.39–3.25 (m overlapped with H_2O, 1H) 2.83 (d, 1H), 2.74–2.60 (m, 1H), 2.22 (s, 3H).

2,7-Dimethyl-1,2,3,4-tetrahydroisoquinoline-3-carboxamide (7e)

The compound was prepared by general procedure I with 7-methyl-1,2,3,4-tetrahydroisoquinoline-3-carboxamide **7d** (0.287 g, 1.51 mmol), 37% aqueous formaldehyde (0.390 mL, 5.28 mmol) and sodium triacetoxyborohydride (1.28 g, 6.03 mmol) in acetonitrile (28.0 mL) and tetrahydrofuran (57.0 mL), stirred at room temperature for 17 h. After workup, the crude product was purified by Isco Combi-Flash Companion column chromatography (0–5% methanol in CH_2Cl_2) to give the desired product 2,7-dimethyl-1,2,3,4-tetrahydroisoquinoline-3-carboxamide **7e** as a white solid (0.242 g, 78.5%). ^{1}H NMR (400 MHz, $CDCl_3$) δ 7.10–6.97 (m, 2H), 6.92–6.87 (m, 2H), 5.32 (br s, 1H), 3.82 (d, 1H), 3.67 (d, 1H), 3.33–3.21 (m, 1H), 3.06–2.99 (m, 2H), 2.43 (s, 3H), 2.31 (s, 3H).

(2,7-Dimethyl-1,2,3,4-tetrahydroisoquinolin-3-yl)methanamine (7f)

The compound was prepared by general procedure J with 2,7-dimethyl-1,2,3,4-tetrahydroisoquinoline-3-carboxamide **7e** (0.242 g, 1.18 mmol) and lithium aluminum hydride (130 mg, 3.55 mmol) in anhydrous tetrahydrofuran (20.0 mL), stirred at 50°C for 17 h. After workup, the crude product was concentrated to give the desired product (2,7-dimethyl-1,2,3,4-tetrahydroisoquinolin-3-yl)methanamine **7f** (0.162 g, 72%).

^{1}H NMR (300 MHz, $CDCl_3$) δ 7.07–6.90 (m, 2H), 6.85 (s, 1H), 3.81 (d, 1H), 3.68 (d, 1H), 2.90–2.79 (m, 2H), 2.78–2.64 (m, 3H), 2.39 (s, 3H), 2.29 (s, 3H).

Methyl 4-fluorophenylalaninate hydrochloride (1:1) (8b)

The compound was prepared by general procedure A with 4-fluoro-dl-phenylalanine **8a** (0.100 g, 0.546 mmol) and thionyl chloride (43.6 µL, 0.601 mmol) in anhydrous methanol (0.8 mL), refluxed for 16 h. The desired product methyl 4-fluorophenylalaninate **8b** was obtained as a as a white solid (0.108 g, quantitative yield). ^{1}H NMR (300 MHz, CD_3OD) δ 7.28 (dd, 2H), 7.11 (dd, 2H), 4.32 (dd, 1H), 3.82 (s, 3H), 3.30–3.12 (m, 2H).

Methyl *N*-(ethoxycarbonyl)-4-fluorophenylalaninate (8c)

The compound was prepared by general procedure C with methyl 4-fluorophenylalaninate **8b** (0.108 g, 0.546 mmol), ethyl chloroformate (57.2 µL, 0.601 mmol) and pyridine (96.8 µL, 1.20 mmol) in dichloromethane (1.79 mL), stirred at rt for 2 h. The desired product methyl *N*-(ethoxycarbonyl)-4-fluorophenylalaninate **8c** was obtained as a yellow liquid (6.88 g, quantitative yield). ^{1}H NMR (300 MHz, $CDCl_3$) δ 7.06 (dd, 1H), 6.88–6.75 (m, 2H), 5.15 (dd, 0.5H), 4.93 (dd, 0.5H), 4.72 (d, 1H), 4.50 (dd, 1H), 4.25–4.10 (m, 2H), 3.59 (s, 3H), 3.25–3.00 (m, 2H), 1.30 (t, 1.5H), 1.23 (t, 1.5H).

2-Ethyl 3-methyl 7-fluoro-3,4-dihydroisoquinoline-2,3(1*H*)-dicarboxylate (8d)

The compound was prepared by general procedure D with methyl *N*-(ethoxycarbonyl)-4-fluorophenylalaninate **8c** (6.59 g, 24.5 mmol) and paraformaldehyde (0.772 g, 25.7 mmol) in a mixture of acetic acid (23.7 mL) and sulfuric acid (7.90 mL), stirred at room temperature for 16 h. After workup, The crude product was concentrated to give the desired product 2-ethyl 3-methyl 7-fluoro-3,4-dihydroisoquinoline-2,3(1*H*)-dicarboxylate **8d** as a yellow liquid (6.88 g, quantitative yield). ^{1}H NMR (300 MHz, $CDCl_3$) δ 7.06 (dd, 1H), 6.88–6.75 (m, 2H), 5.15 (dd, 0.5H), 4.93 (dd, 0.5H), 4.72 (d, 1H), 4.50 (dd, 1H), 4.25–4.10 (m, 2H), 3.59 (s, 3H), 3.25–3.00 (m, 2H), 1.30 (t, 1.5H), 1.23 (t, 1.5H).

7-Fluoro-1,2,3,4-tetrahydroisoquinoline-3-carboxylic acid hydrochloride (1:1) (8e)

The compound was prepared by general procedure E with 2-ethyl 3-methyl 7-fluoro-3,4-dihydroisoquinoline-2,3(1*H*)-dicarboxylate **8d** (0.726 g, 2.58 mmol) in aqueous 6 N hydrogen chloride (10.5 mL), refluxed for 48 h. The desired product 7-fluoro-1,2,3,4-tetrahydroisoquinoline-3-carboxylic acid

hydrochloride (1:1) **8e** was obtained as a brown solid (0.598 g, 98%). ^{1}H NMR (300 MHz, CD_3OD) δ 7.33 (dd, 1H), 7.13–7.02 (m, 2H), 4.52–4.38 (m, 3H), 3.47 (dd, 1H), 3.19 (dd, 1H).

Methyl 7-fluoro-1,2,3,4-tetrahydroisoquinoline-3-carboxylate (8f)

The compound was prepared by general procedure F with 7-fluoro-1,2,3,4-tetrahydroisoquinoline-3-carboxylic acid hydrochloride (1:1) **8e** (0.100 g, 0.432 mmol) and sulfuric acid (50.0 µL, 0.938 mmol) in methanol (2.5 mL), refluxed for 16 h. The desired product methyl 7-fluoro-1,2,3,4-tetrahydroisoquinoline-3-carboxylate **8f** was obtained as a brown liquid (79.8 mg, 88%). ^{1}H NMR (300 MHz, $CDCl_3$) δ 7.06 (dd, 1H), 6.85 (td, 1H), 6.73 (d, 1H), 4.16–4.00 (m, 2H), 3.77 (s, 3H), 3.72 (dd, 1H), 3.05 (dd, 1H), 2.90 (dd, 1H).

7-Fluoro-1,2,3,4-tetrahydroisoquinoline-3-carboxamide (8g)

The compound was prepared by general procedure H with methyl 7-fluoro-1,2,3,4-tetrahydroisoquinoline-3-carboxylate **8f** (3.52 g, 16.8 mmol) in 28–30% aqueous ammonium hydroxide (46.8 mL, 336 mmol), stirred at room temperature for 16 h. The desired product 7-fluoro-1,2,3,4-tetrahydroisoquinoline-3-carboxamide **8g** was obtained as a white solid white solid (2.83 g, 87%). ^{1}H NMR (400 MHz, CD_3OD) δ 7.13 (dd, 1H), 6.87 (td, 1H), 6.82 (dd, 1H), 4.05 (d, 1H), 3.97 (d, 1H), 3.55 (dd, 1H), 3.02 (dd, 1H), 2.83 (dd, 1H).

7-Fluoro-2-methyl-1,2,3,4-tetrahydroisoquinoline-3-carboxamide (8h)

The compound was prepared by general procedure I with 7-fluoro-1,2,3,4-tetrahydroisoquinoline-3-carboxamide **8g** (0.800 g, 4.12 mmol), 37% aqueous formaldehyde (1.08 mL, 14.4 mmol) and sodium triacetoxyborohydride (3.49 g, 16.5 mmol) in acetonitrile (60 mL), stirred at room temperature for 5 h. The desired product 7-fluoro-2-methyl-1,2,3,4-tetrahydroisoquinoline-3-carboxamide **8h** was obtained as a white solid (0.786 g, 92%). ^{1}H NMR (300 MHz, CD_3OD) δ 7.13 (dd, 1H), 6.90 (dd, 1H), 6.84 (d, 1H), 3.95 (d, 1H), 3.57 (d, 1H), 3.17 (dd, 1H), 3.10–2.92 (m, 2H), 2.44 (s, 3H).

1-(7-Fluoro-2-methyl-1,2,3,4-tetrahydroisoquinolin-3-yl)methanamine (8i)

The compound was prepared by general procedure J with 7-fluoro-2-methyl-1,2,3,4-tetrahydroisoquinoline-3-carboxamide **8h** (0.386 g, 1.85 mmol) and lithium aluminum hydride (0.211 g, 5.56 mmol) in anhydrous tetrahydrofuran (36.9 mL), stirred at 60 ˚C for for 18 h. After workup, the crude product was concentrated to give the desired product

1-(7-fluoro-2-methyl-1,2,3,4-tetrahydroisoquinolin-3-yl)methanamine **8i** as a yellow liquid (0.352 g, 98%). ^{1}H NMR (300 MHz, $CDCl_3$) δ 6.82 (dd, 1H), 6.62 (td, 1H), 6.51 (dd, 1H), 3.58 (d, 1H), 3.43 (d, 1H), 2.68–2.37 (m, 5H), 2.16 (s, 3H).

Methyl 2-amino-3-(4-chlorophenyl)propanoate hydrochloride (1:1) (9b)

The compound was prepared by general procedure A with dl-4-chlorophenylalanine **9a** (3.00 g, 15.0 mmol) and thionyl chloride (1.20 mL, 16.5 mmol) in anhydrous methanol (75.0 mL), refluxed for 18 h. The desired product methyl 2-amino-3-(4-chlorophenyl)propanoate hydrochloride (1:1) **9b** was obtained as a white solid (3.76 g, quantitative yield). ^{1}H NMR (300 MHz, DMSO-d_6) δ 8.74 (br s, 2H), 7.39 (d, 2H), 7.28 (d, 2H), 4.27 (t, 1H), 3.68 (s, 3H), 3.23–3.08 (m, 2H).

Methyl 3-(4-chlorophenyl)-2-((ethoxycarbonyl)amino)propanoate (9c)

The compound was prepared by general procedure C with methyl 2-amino-3-(4-chlorophenyl)propanoate hydrochloride (1:1) **9b** (3.46 g, 13.8 mmol), ethyl chloroformate (2.04 mL, 14.5 mmol) and pyridine (2.45 mL, 30.4 mmol) in dichloromethane (46.0 mL), stirred at 0 ˚C for 1 h. The desired product methyl 3-(4-chlorophenyl)-2-((ethoxycarbonyl)amino)propanoate **9c** was obtained as a white solid (3.89 g, 98%). ^{1}H NMR (300 MHz, DMSO-d_6) δ 7.63 (d, 1H), 7.34 (d, 2H), 7.26 (d, 2H), 4.26–4.18 (m, 1H), 3.92 (qd, 2H), 3.62 (s, 3H), 3.02 (dd, 1H), 2.84 (dd, 1H), 1.11 (t, 3H).

2-Ethyl 3-methyl 7-chloro-3,4-dihydroisoquinoline-2,3(1*H*)-dicarboxylate (9d)

The compound was prepared by general procedure D with methyl 3-(4-chlorophenyl)-2-((ethoxycarbonyl)amino)propanoate **9c** (3.89 g, 13.6 mmol) and paraformaldehyde (0.45 g, 15.0 mmol) in a mixture of acetic acid (40.5 mL) and sulfuric acid (13.5 mL), stirred at room temperature for 18 h. After workup, the crude product was purified by flash column chromatography (SiO_2, 20% ethyl acetate in n-hexane) to give the desired product 2-ethyl 3-methyl 7-chloro-3,4-dihydroisoquinoline-2,3(1*H*)-dicarboxylate **9d** as a colorless oil (3.09 g, 76%). ^{1}H NMR (300 MHz, $CDCl_3$) δ 7.27 (d, 1H), 7.15–7.06 (m, 2H), 5.29–4.94 (m, 1H), 4.78–4.39 (m, 2H), 4.29–4.14 (m, 2H), 3.62 (s, 3H), 3.27–3.07 (m, 2H), 1.35–1.23 (m, 3H).

7-Chloro-1,2,3,4-tetrahydroisoquinoline-3-carboxylic acid hydrochloride (1:1) (9e)

The compound was prepared by general procedure E with 2-ethyl 3-methyl 7-chloro-3,4-

dihydroisoquinoline-2,3(1*H*)-dicarboxylate **9d** (3.06 g, 13.6 mmol) in aqueous 6 N hydrogen chloride (51.0 mL), refluxed for 48 h. The desired product 7-chloro-1,2,3,4-tetrahydroisoquinoline-3-carboxylic acid hydrochloride (1:1) **9e** was obtained as a white solid (1.19 g, 84%). ^{1}H NMR (300 MHz, DMSO-d_6) δ 9.99 (br s, 1H), 8.45 (br s, 1H), 7.40–7.37 (m, 1H), 7.37–7.25 (m, 2H), 4.50–4.07 (m, 3H), 3.31 (dd overlapped with H_2O, 1H), 3.16–3.03 (m, 1H).

Methyl 7-chloro-1,2,3,4-tetrahydroisoquinoline-3-carboxylate hydrochloride (1:1) (9f)

The compound was prepared by general procedure G with 7-chloro-1,2,3,4-tetrahydroisoquinoline-3-carboxylic acid hydrochloride (1:1) **9e** (1.19 g, 4.80 mmol), thionyl chloride (1.22 mL, 16.8 mmol) and triethylamine (0.67 mL, 4.80 mmol) in anhydrous methanol (48.0 mL), refluxed for 18 h. The desired product methyl 7-chloro-1,2,3,4-tetrahydroisoquinoline-3-carboxylate hydrochloride (1:1) **9f** was obtained as a white solid (1.05 g, 83%). ^{1}H NMR (300 MHz, DMSO-d_6) δ 9.96 (br s, 2H), 7.40 (d, 1H), 7.38–7.27 (m, 2H), 4.54 (dd, 1H), 4.42–4.23 (m, 2H), 3.81 (s, 3H), 3.42–3.24 (m overlapped with H_2O, 1H), 3.11 (dd, 1H).

7-Chloro-1,2,3,4-tetrahydroisoquinoline-3-carboxamide (9g)

The compound was prepared by general procedure H with methyl 7-chloro-1,2,3,4-tetrahydroisoquinoline-3-carboxylate hydrochloride (1:1) **9f** (1.05 g, 4.00 mmol) in 28–30% aqueous ammonium hydroxide (40.0 mL), stirred at room temperature for 18 h. The desired product 7-chloro-2-methyl-1,2,3,4-tetrahydroisoquinoline-3-carboxamide **9g** was obtained as a white solid (711 mg, 84%). ^{1}H NMR (300 MHz, DMSO-d_6) δ 7.33 (br s, 1H), 7.24–7.11 (m, 3H), 7.08 (br s, 1H), 4.01–3.76 (m, 2H), 3.39–3.27 (m overlapped with H_2O, 1H), 2.87 (dd, 1H), 2.67 (dd, 1H), 2.59 (br s, 1H).

7-Chloro-2-methyl-1,2,3,4-tetrahydroisoquinoline-3-carboxamide (9h)

The compound was prepared by general procedure I with 7-chloro-2-methyl-1,2,3,4-tetrahydroisoquinoline-3-carboxamide **9g** (711 mg, 3.38 mmol), 37% aqueous formaldehyde (0.96 mL, 11.8 mmol), sodium triacetoxyborohydride (2.86 g, 13.5 mmol) and acetic acid (0.2 mL, 3.38 mmol) in acetonitrile (54.0 mL) and tetrahydrofuran (13.0 mL), stirred at room temperature for 5 h. The desired product 7-chloro-2-methyl-1,2,3,4-tetrahydroisoquinoline-3-carboxamide **9h** was obtained as a white solid (459 mg, 60%). ^{1}H NMR (300 MHz, DMSO-d_6) δ 7.41 (br s, 1H), 7.20–7.11 (m, 3H), 7.08 (br s, 1H), 3.87 (d, 1H), 3.50 (d, 1H), 3.10 (dd, 1H), 2.97–2.78 (m, 2H), 2.31 (s, 3H).

(7-Chloro-2-methyl-1,2,3,4-tetrahydroisoquinolin-3-yl)methanamine (9i)

The compound was prepared by general procedure J with 7-chloro-2-methyl-1,2,3,4-tetrahydroisoquinoline-3-carboxamide **9h** (459 mg, 2.04 mmol) and lithium aluminum hydride (232 mg, 6.13 mmol) in anhydrous tetrahydrofuran (70.0 mL), stirred at 60 °C for for 4 h. After workup, the crude product was purified by flash column chromatography (SiO_2, 10% methanol with 1% triethylamine in dichloromethane) to give the desired product (7-chloro-2-methyl-1,2,3,4-tetrahydroisoquinolin-3-yl) methanamine **9i** as a brown syrup (155 mg, 36%). ^{1}H NMR (300 MHz, DMSO-d_6) δ 7.19–6.99 (m, 3H), 3.74 (d, 1H), 3.51 (d, 1H), 2.74–2.68 (m, 3H), 2.57 (q, 1H), 2.52–2.43 (m overlapped with d_6-DMSO, 1H), 2.28 (s, 3H), 1.59 (br s, 1H).

(3*S*)-2-Methyl-1,2,3,4-tetrahydroisoquinoline-3-carboxamide (10b)

The compound was prepared by general procedure I with (3*S*)-1,2,3,4-tetrahydroisoquinoline-3-carboxamide **10a** (1.60 g, 9.10 mmol), 37% aqueous formaldehyde (2.39 mL, 31.8 mmol), sodium triacetoxyborohydride (7.71 g, 36.4 mmol) and acetic acid (0.521 mL, 9.10 mmol) in acetonitrile (91.0 mL) and tetrahydrofuran (91.0 mL), stirred at room temperature for 16 h. The desired product (3*S*)-2-methyl-1,2,3,4-tetrahydroisoquinoline-3-carboxamide **10b** was obtained as a white solid (1.73 g, quantitative yield). ^{1}H NMR (300 MHz, CD_3OD) δ 7.17–7.04 (m, 4H), 3.99 (d, 1H), 3.59 (d, 1H), 3.22–3.13 (m, 1H), 3.08 (d, 1H), 3.00 (dd, 1H), 2.46 (s, 3H).

[(3*S*)-2-Methyl-1,2,3,4-tetrahydroisoquinolin-3-yl]methanamine (10c)

The compound was prepared by general procedure J with (3*S*)-2-methyl-1,2,3,4-tetrahydroisoquinoline-3-carboxamide **10b** (0.800 g, 4.21 mmol) and lithium aluminum hydride (0.120 g, 3.15 mmol) in anhydrous tetrahydrofuran (19.7 mL), refluxed for 16 h. After workup, the crude product was purified by flash column chromatography (SiO_2, dichloromethane/methanol/ammonium hydroxide solution = 92/7.6/0.4) to give the desired product [(3*R*)-2-methyl-1,2,3,4-tetrahydroisoquinolin-3-yl]methanamine **10c** as a yellow liquid (0.621 g, 84%). ^{1}H NMR (400 MHz, $CDCl_3$) δ 7.16–7.06 (m, 3H), 7.04–6.99 (m, 1H), 3.84 (d, 1H), 3.70 (d, 1H), 2.84 (dd, 2H), 2.78 (d, 2H), 2.71–2.64 (m, 1H), 2.40 (s, 3H).

Methyl (3*R*)-1,2,3,4-tetrahydroisoquinoline-3-carboxylate (11b)

The compound was prepared by general procedure G with (3*R*)-1,2,3,4-tetrahydroisoquinoline-3-carboxylic

acid hydrochloride (1:1) **11a** (0.260 g, 1.22 mmol), thionyl chloride (0.309 mL, 4.26 mmol) and triethylamine (0.187 mL, 1.34 mmol) in anhydrous methanol (12.0 mL), refluxed for 16 h. After workup, the crude product was purified by flash column chromatography (1% methanol in dichloromethane) to give the desired product methyl (3*R*)-1,2,3,4-tetrahydroisoquinoline-3-carboxylate **11b** as a colorless liquid (0.209 g, 90%). ^{1}H NMR (400 MHz, $CDCl_3$) δ 7.18–7.09 (m, 3H), 7.06–7.01 (m, 1H), 4.15 (d, 1H), 4.09 (d, 1H), 3.81–3.74 (m, 4H), 3.10 (dd, 1H), 2.97 (dd, 1H).

(3*R*)-1,2,3,4-Tetrahydroisoquinoline-3-carboxamide (11c)

The compound was prepared by general procedure H with (3*R*)-1,2,3,4-tetrahydroisoquinoline-3-carboxylate **11b** (3.06 g, 16.0 mmol) in 28–30% aqueous ammonium hydroxide (32.0 mL), stirred at room temperature for 16 h. The desired product (3*R*)-1,2,3,4-tetrahydroisoquinoline-3-carboxamide **11c** was obtained as a white solid (2.58 g, 92%). ^{1}H NMR (400 MHz, CD_3OD) δ 7.15–7.09 (m, 3H), 7.08–7.03 (m, 1H), 4.08–3.95 (m, 2H), 3.57 (dd, 1H), 3.04 (dd, 1H), 2.89 (dd, 1H).

(3*R*)-2-Methyl-1,2,3,4-tetrahydroisoquinoline-3-carboxamide (11d)

The compound was prepared by general procedure I with (3*R*)-1,2,3,4-tetrahydroisoquinoline-3-carboxamide **11c** (0.118 g, 0.670 mmol), 37% aqueous formaldehyde (75.3 µL, 1.01 mmol), sodium triacetoxyborohydride (0.284 g, 1.34 mmol) and acetic acid (38.4 µL, 0.670 mmol) in acetonitrile (3.40 mL) and tetrahydrofuran (3.40 mL), stirred at room temperature for 16 h. The desired product (3*R*)-2-methyl-1,2,3,4-tetrahydroisoquinoline-3-carboxamide **11d** was obtained as a white solid (0.122 g, 96%). ^{1}H NMR (400 MHz, CD_3OD) δ 7.16–7.05 (m, 4H), 3.96 (d, 1H), 3.56 (d, 1H), 3.17–3.05 (m, 2H), 2.99 (dd, 1H), 2.44 (s, 3H).

[(3*R*)-2-Methyl-1,2,3,4-tetrahydroisoquinolin-3-yl]methanamine (11e)

The compound was prepared by general procedure J with (3*R*)-2-methyl-1,2,3,4-tetrahydroisoquinoline-3-carboxamide **11d** (0.800 g, 4.21 mmol) and lithium aluminum hydride (0.479 g, 12.6 mmol) in anhydrous tetrahydrofuran (21.0 mL), refluxed for 36 h. After workup, the crude product was purified by flash column chromatography (SiO_2, dichloromethane/methanol/ammonium hydroxide solution = 92/7.6/0.4) to give the desired product [(3*R*)-2-methyl-1,2,3,4-tetrahydroisoquinolin-3-yl]methanamine **11e** as a yellow liquid (0.621 g, 84%). ^{1}H NMR (400 MHz, $CDCl_3$) δ 7.16–7.06 (m, 3H), 7.04–6.99 (m, 1H), 3.84 (d, 1H), 3.70 (d, 1H),

2.84 (dd, 2H), 2.78 (d, 2H), 2.71–2.64 (m, 1H), 2.40 (s, 3H).

Methyl (2*S*)-2-amino-3-(3-chlorophenyl)propanoate hydrochloride (1:1) (12b)

The compound was prepared by general procedure A with (2*S*)-2-amino-3-(3-chlorophenyl)propanoic acid **12a** (10.0 g, 50.1 mmol) and thionyl chloride (4.00 mL, 55.1 mmol) in anhydrous methanol (150 mL), refluxed for 18 h. The desired product methyl (2*S*)-2-amino-3-(3-chlorophenyl)propanoate hydrochloride (1:1) **12b** was obtained as a white solid (3.66 g, 34%). ^{1}H NMR (600 MHz, d_6-DMSO) δ 7.31–7.28 (m, 1H), 7.28–7.25 (m, 2H), 7.15 (dt, 1H), 3.58 (s, 3H), 3.56 (dd, 1H), 2.87 (dd, 1H), 2.75 (dd, 1H), 1.79 (s, 2H).

Methyl (2*S*)-3-(3-chlorophenyl)-2-[(ethoxycarbonyl)amino]propanoate (12c)

The compound was prepared by general procedure C with methyl (2*S*)-2-amino-3-(3-chlorophenyl)propanoate hydrochloride (1:1) **12b** (3.66 g, 17.1 mmol), ethyl chloro-formate (2.52 mL, 18 mmol) and pyridine (3.03 mL, 37.7 mmol) in dichloromethane (54.9 mL), stirred at 0 ˚C for 1 h. The desired product methyl (2*S*)-3-(3-chlorophenyl)-2-[(ethoxycarbonyl)amino]propanoate **12c** was obtained as a yellow oil (4.89 g, quantitative yield). ^{1}H NMR (600 MHz, d_6-DMSO) δ 7.66 (d, 1H), 7.33 (br t, 1H), 7.31 (d, 1H), 7.28 (dt, 1H), 7.21 (dt, 1H), 4.24 (ddd, 1H), 3.98–3.87 (m, 2H), 3.63 (s, 3H), 3.04 (dd, 1H), 2.84 (dd, 1H), 1.10 (t, 3H).

2-Ethyl 3-methyl (3*S*)-6-chloro-3,4-dihydroisoquinoline-2,3(1*H*)-dicarboxylate (12d)

The compound was prepared by general procedure D with methyl (2*S*)-3-(3-chlorophenyl)-2-[(ethoxycarbonyl)amino]propanoate **12c** (4.89 g, 17.1 mmol) and paraformaldehyde (566 mg, 18.8 mmol) in a mixture of acetic acid (25.5 mL) and sulfuric acid (8.5 mL), stirred at room temperature for 18 h. After workup, the crude was purified by flash column chromatography (SiO_2, 20% ethyl acetate in n-hexane) to give an inseparable mixture of 2-ethyl 3-methyl (3*S*)-6-chloro-3,4-dihydroisoquinoline-2,3(1*H*)-dicarboxylate and 2-ethyl 3-methyl (3*S*)-8-chloro-3,4-dihydroisoquinoline-2,3(1*H*)-dicarboxylate **12d** as a colorless oil (3.61 g, 71%). ^{1}H NMR (600 MHz, d_6-DMSO) δ 7.38–7.32 (m, 1H), 7.30–7.21 (m, 2H), 5.12–4.85 (m, 1H), 4.70–4.56 (m, 1H), 4.43 (dd, 1H), 4.20–4.03 (m, 2H), 3.59–3.53 (m, 3H), 3.23–3.10 (m, 2H), 1.28–1.22 (m, 1.5H), 1.20–1.13 (m, 1.5H).

(3*S*)-6-Chloro-1,2,3,4-tetrahydroisoquinoline-3-carboxylic acid hydrochloride (1:1) (12e)

The compound was prepared by general procedure E with 2-ethyl 3-methyl (3*S*)-6-chloro-3,4-dihydroisoquinoline-2,3(1*H*)-dicarboxylate and 2-ethyl 3-methyl (3*S*)-8-chloro-3,4-dihydroisoquinoline-2,3(1*H*)-dicarboxylate **12d** (3.61 g, 12.1 mmol) in aqueous 8 N hydrogen chloride (121 mL), refluxed for 48 h. The desired product (3*S*)-6-chloro-1,2,3,4-tetrahydroisoquinoline-3-carboxylic acid hydrochloride (1:1) **12e** as a white solid (2.18 g, 51%). ^{1}H NMR (600 MHz, d_6-DMSO) δ 9.81 (br s, 1H), 7.41 (d, 1H), 7.34 (dd, 1H), 7.30 (d, 1H), 4.41–4.32 (m, 2H), 4.26 (d, 1H), 3.33–3.24 (m overlapped with H_2O, 2H), 3.10 (dd, 1H).

Methyl (3*S*)-6-chloro-1,2,3,4-tetrahydroisoquinoline-3-carboxylate (12f)

The compound was prepared by general procedure G with (3*S*)-6-chloro-1,2,3,4-tetrahydroisoquinoline-3-carboxylic acid hydrochloride **12e** (2.18 g, 8.79 mmol), thionyl chloride (2.23 mL, 30.8 mmol) and triethylamine (1.35 mL, 9.66 mmol) in anhydrous methanol (44 mL), refluxed for 18 h. After workup, the crude mixture was concentrated to give the desired product methyl (3*S*)-6-chloro-1,2,3,4-tetrahydroisoquinoline-3-carboxylate **12f** as a colorless oil (720 mg, 36%). ^{1}H NMR (600 MHz, d_6-DMSO) δ 7.20 (d, 1H), 7.16 (dd, 1H), 7.07 (d, 1H), 3.92 (d, 1H), 3.84 (d, 1H), 3.69–3.65 (m, 4H), 2.95 (dd, 1H), 2.84–2.78 (m, 2H).

(3*S*)-6-Chloro-1,2,3,4-tetrahydroisoquinoline-3-carboxamide (12g)

The compound was prepared by general procedure H with methyl (3*S*)-6-chloro-1,2,3,4-tetrahydroisoquinoline-3-carboxylate **12f** (720 mg, 3.19 mmol) in 28–30% aqueous ammonium hydroxide (32 mL), stirred at room temperature for 18 h, to give the desired product (3*S*)-6-chloro-1,2,3,4-tetrahydroisoquinoline-3-carboxamide **12g** as a white solid (520 mg, 77%). ^{1}H NMR (600 MHz, d_6-DMSO) δ 7.33 (br s, 1H), 7.19 (d, 1H), 7.15 (dd, 1H), 7.08 (br s, 1H), 7.06 (d, 1H), 3.90 (d, 1H), 3.82 (d, 1H), 3.38–3.32 (m, 1H), 2.89 (dd, 1H), 2.71 (dd, 1H), 2.59 (br s, 1H).

(3*S*)-6-Chloro-2-methyl-1,2,3,4-tetrahydroisoquinoline-3-carboxamide (12h)

The compound was prepared by general procedure I with (3*S*)-6-chloro-1,2,3,4-tetrahydroisoquinoline-3-carboxamide **12g** (520 mg, 2.47 mmol), 37% aqueous formaldehyde (0.300 mL, 3.70 mmol), sodium triacetoxyborohydride (1.05 g, 4.94 mmol) and acetic acid (0.140 mL, 2.47 mmol) in tetrahydrofuran (12 mL), stirred at room temperature for 2 h. The desired product (3*S*)-6-chloro-2-methyl-1,2,3,4-tetrahydroisoquinoline-3-carboxamide **12h** was obtained as a white solid

(599 mg, quantitative yield). ^{1}H NMR (400 MHz, d_6-DMSO) δ 7.41 (br s, 1H), 7.21 (d, 1H), 7.16 (dd, 1H), 7.11–7.05 (m, 2H), 3.86 (d, 1H), 3.47 (d, 1H), 3.07 (dd, 1H), 2.97–2.82 (m, 2H), 2.31 (s, 3H).

[(3*S*)-6-Chloro-2-methyl-1,2,3,4-tetrahydroisoquinolin-3-yl]methanamine (12i)

The compound was prepared by general procedure J with (3*S*)-6-chloro-2-methyl-1,2,3,4-tetrahydroisoquinoline-3-carboxamide **12h** (120 mg, 0.534 mmol) and 1M lithium aluminium hydride in THF (1.60 mL, 1.60 mmol) in anhydrous tetrahydrofuran (10.6 mL), stirred at 60 ˚C for for 18 h. After workup, the crude was purified by flash column chromatography (SiO_2, 10–20% methanol with 1% triethylamine in dichloromethane) to give the desired product [(3*S*)-6-chloro-2-methyl-1,2,3,4-tetrahydroisoquinolin-3-yl]methanamine **12i** as a colorless oil (77.0 mg, 69%). ^{1}H NMR (600 MHz, d_6-DMSO) δ 7.16–7.13 (m, 2H), 7.07 (d, 1H), 3.72 (d, 1H), 3.48 (d, 1H), 2.74 (d, 2H), 2.70 (dd, 1H), 2.55 (dd, 1H), 2.49–2.45 (m overlapped with DMSO, 1H), 2.29 (s, 3H), 1.48 (br s, 2H).

Methyl (2*R*)-2-amino-3-(3-chlorophenyl)propanoate hydrochloride (1:1) (13b)

The compound was prepared by general procedure A with (2*R*)-2-amino-3-(3-chlorophenyl)propanoic acid **13a** (5g, 25 mmol) and thionyl chloride (2 mL, 27.6 mmol) in anhydrous methanol (62.6 mL), refluxed for 16 h. The desired product methyl (2*R*)-2-amino-3-(3-chlorophenyl)propanoate hydrochloride (1:1) **13b** was obtained as a white solid (6.26 g, quantitative yield). ^{1}H NMR (CD_3OD, 400 MHz) δ 7.40–7.32 (m, 3H), 7.21 (dt, 1H), 4.36 (t, 1H), 3.82 (s, 3H), 3.27 (dd, overlapped with CD_3OD, 1H), 3.17 (dd, 1H).

Methyl (2*R*)-3-(3-chlorophenyl)-2-[(methoxycarbonyl)amino]propanoate (13c)

The compound was prepared by general procedure C with methyl (2*R*)-2-amino-3-(3-chlorophenyl)propanoate hydrochloride (1:1) **13b** (6.42 g, 25.7 mmol), methyl chloroformate (2.08 mL, 27 mmol) and sodium bicarbonate (4.74 g, 56.5 mmol) in dichloromethane (64.2 mL), stirred at room temperature for 16 h. The desired product methyl (2*R*)-3-(3-chlorophenyl)-2-[(methoxycarbonyl)amino]propanoate **13c** was obtained as a colorless liquid (6.76 g, 97%). ^{1}H NMR ($CDCl_3$, 400 MHz) δ 7.23–7.20 (m, 2H), 7.11 (s, 1H), 7.02–6.98 (m, 1H), 5.23 (br d, 1H), 4.63 (br q, 1H), 3.72 (s, 3H), 3.66 (s, 3H), 3.07 (ddd, 2H).

Dimethyl (3*R*)-6-chloro-3,4-dihydroisoquinoline-2,3(1*H*)-dicarboxylate (13d)

The compound was prepared by general procedure D with methyl

(2*R*)-3-(3-chlorophenyl)-2-[(methoxycarbonyl)amino]propanoate **13c** (1.06 g, 4.17 mmol) and paraformaldehyde (1.12 g, 37.3 mmol) in a mixture of acetic acid (31.1 mL) and sulfuric acid (3.98 mL), stirred at room temperature for 16 h. An inseparable mixture of dimethyl (3*R*)-6-chloro-3,4-dihydroisoquinoline-2,3(1*H*)-dicarboxylate and dimethyl (3*R*)-8-chloro-3,4-dihydroisoquinoline-2,3(1*H*)-dicarboxylate **13d** was obtained as a yellow liquid (6.45 g, 88%). ^{1}H NMR ($CDCl_3$, 400 MHz) δ 7.25–7.00 (m, 3H), 5.17 (dd, 0.5H), 4.97 (dd, 0.5H), 4.73 (dd, 1H), 4.51 (dd, 1H), 3.85–3.72 (m, 3H), 3.63 (s, 3H), 3.34–3.09 (m, 2H).

(3*R*)-6-Chloro-1,2,3,4-tetrahydroisoquinoline-3-carboxylic acid hydrochloride (1:1) (13e)

The compound was prepared by general procedure E with dimethyl (3*R*)-6-chloro-3,4-dihydroisoquinoline-2,3(1*H*)-dicarboxylate and dimethyl (3*R*)-8-chloro-3,4-dihydroisoquinoline-2,3(1*H*)-dicarboxylate **13d** (4.85 g, 17.1 mmol) in aqueous 12 N hydrogen chloride (33.4 mL), refluxed for 16 h. The desired product (3*R*)-6-chloro-1,2,3,4-tetrahydroisoquinoline-3-carboxylic acid hydrochloride (1:1) **13e** was obtained as a white solid (3.05 g, 72%). ^{1}H NMR (CD_3OD, 400 MHz) δ 7.37 (s, 1H), 7.32 (dd, 1H), 7.26 (d, 1H), 4.50–4.36 (m, 3H), 3.49 (dd, 1H), 3.23 (dd, 1H).

Methyl (3*R*)-6-chloro-1,2,3,4-tetrahydroisoquinoline-3-carboxylate (13f)

The compound was prepared by general procedure G with (3*R*)-6-chloro-1,2,3,4-tetrahydroisoquinoline-3-carboxylic acid hydrochloride (1:1) **13e** (3.05 g, 12.3 mmol), thionyl chloride (3.12 mL, 43.0 mmol) and triethylamine (1.89 mL, 13.5 mmol) in anhydrous methanol (61.5 mL), stirred at room temperature for 16 h. After workup, the crude was purified by flash column chromatography (SiO_2, dichloromethane/methanol = 98/2) to give the desired product methyl (3*R*)-6-chloro-1,2,3,4-tetrahydroisoquinoline-3-carboxylate **13f** as a colorless liquid (2.42 g, 87%). ^{1}H NMR ($CDCl_3$, 400 MHz) δ 7.13–7.09 (m, 2H), 6.96 (d, 1H), 4.10 (d, 1H), 4.02 (d, 1H), 3.78 (s, 3H), 3.72 (dd, 1H), 3.06 (dd, 1H), 2.94 (dd, 1H).

(3*R*)-6-Chloro-1,2,3,4-tetrahydroisoquinoline-3-carboxamide (13g)

The compound was prepared by general procedure H with methyl (3*R*)-6-chloro-1,2,3,4-tetrahydroisoquinoline-3-carboxylate **13f** (1.00 g, 4.43 mmol) in 28–30% aqueous

ammonium hydroxide (22.2 mL), stirred at room temperature for 16 h, to give the desired product (3*R*)-6-chloro-1,2,3,4-tetrahydroisoquinoline-3-carboxamide **13g** as a white solid (0.794 g, 85%). ^{1}H NMR (CD_3OD, 400 MHz) δ 7.17–7.12 (m, 2H), 7.05 (d, 1H), 4.03 (d, 1H), 3.95 (d, 1H), 3.55 (dd, 1H), 3.02 (dd, 1H), 2.87 (dd, 1H).

(3*R*)-6-Chloro-2-methyl-1,2,3,4-tetrahydroisoquinoline-3-carboxamide (13h)

The compound was prepared by general procedure I with (3*R*)-6-chloro-1,2,3,4-tetrahydroisoquinoline-3-carboxamide **13g** (0.794 g, 3.77 mmol), 37% aqueous formaldehyde (0.424 mL, 5.65 mmol), sodium triacetoxyborohydride (1.6 g, 7.53 mmol) and acetic acid (0.216 mL, 3.77 mmol) in acetonitrile (18.8 mL) and tetrahydrofuran (18.8 mL), stirred at room temperature for 16 h. The desired product (3*R*)-6-chloro-2-methyl-1,2,3,4-tetrahydroisoquinoline-3-carboxamide **13h** was obtained as a white solid (0.825 g, 97%). ^{1}H NMR (CD_3OD, 400 MHz) δ 7.17–7.12 (m, 2H), 7.07 (d, 1H), 3.95 (d, 1H), 3.54 (d, 1H), 3.16 (dd, 1H), 3.12–2.94 (m, 2H), 2.44 (s, 3H).

[(3*R*)-6-Chloro-2-methyl-1,2,3,4-tetrahydroisoquinolin-3-yl]methanamine (13i)

The compound was prepared by general procedure J with (3*R*)-6-chloro-2-methyl-1,2,3,4-tetrahydroisoquinoline-3-carboxamide **13h** (0.355 g, 1.7 mmol) and lithium aluminium hydride (0.101 g, 2.67 mmol) in anhydrous tetrahydrofuran (8.9 mL), refluxed for 24 h. After workup, the crude was purified by flash column chromatography (SiO_2, dichloromethane/methanol/ammonium hydroxide solution = 92/7.6/0.4) to give the desired product [(3*R*)-6-chloro-2-methyl-1,2,3,4-tetrahydroisoquinolin-3-yl]methanamine **13i** as a yellow liquid (0.127 g, 64%). ^{1}H NMR ($CDCl_3$, 400 MHz) δ 7.03–6.99 (m, 2H), 6.87 (d, 1H), 3.72 (d, 1H), 3.55 (d, 1H), 2.80–2.69 (m, 2H), 2.67 (d, 2H), 2.60–2.52 (m, 1H), 2.31 (s, 3H).

Methyl (2*S*)-2-amino-3-(3-methylphenyl)propanoate hydrochloride (1:1) (14b)

The compound was prepared by general procedure A with (2*S*)-2-amino-3-(3-methyl phenyl)propanoic acid **14a** (8.00 g, 44.6 mmol) and thionyl chloride (3.57 mL, 49.1 mmol) in anhydrous methanol (120 mL), refluxed for 18 h. The desired product methyl (2*S*)-2-amino-3-(3-methylphenyl)propanoate hydrochloride (1:1) **14b** was obtained as a white solid (10.2 g,

quantitative yield). ^{1}H NMR (600 MHz, DMSO-d_6) δ 8.50 (br s, 2H), 7.22 (t, 1H), 7.10 (d, 1H), 7.04 (s, 1H), 7.01 (d, 1H), 4.26 (t, 1H), 3.67 (s, 3H), 3.10 (dd, 1H), 3.04 (dd, 1H), 2.29 (s, 3H).

Methyl (2*S*)-2-[(ethoxycarbonyl)amino]-3-(3-methylphenyl)propanoate (14c)

The compound was prepared by general procedure C with methyl (2*S*)-2-amino-3-(3-methylphenyl)propanoate hydrochloride (1:1) **14b** (10.2 g, 44.6 mmol), ethyl chloroformate (4.49 mL, 46.9 mmol) and pyridine (7.91 mL, 98.2 mmol) in dichloromethane (153 mL), stirred at 0 ˚C for 1 h. The desired product methyl (2*S*)-2-[(ethoxycarbonyl)amino]-3-(3-methylphenyl)propanoate **14c** was obtained as a brown oil (11.8 g, quantitative yield). ^{1}H NMR (600 MHz, DMSO-d_6) δ 7.62 (d, 1H), 7.16 (t, 1H), 7.06–7.00 (m, 3H), 4.19 (ddd, 1H), 3.97–3.88 (m, 2H), 3.61 (s, 3H), 2.96 (dd, 1H), 2.80 (dd, 1H), 2.26 (s, 3H), 1.11 (t, 3H).

2-Ethyl 3-methyl (3*S*)-6-methyl-3,4-dihydroisoquinoline-2,3(1*H*)-dicarboxylate and 2-ethyl 3-methyl (3*S*)-8-methyl-3,4-dihydroisoquinoline-2,3(1*H*)-dicarboxylate (14d)

The compound was prepared by general procedure D with methyl (2*S*)-2-((ethoxycarbonyl)amino)-3-(3-methylphenyl)propanoate **14c** (11.8 g, 44.6 mmol) and paraformaldehyde (1.47 g, 49.1 mmol) in a mixture of acetic acid (67.5 mL) and sulfuric acid (22.5 mL), stirred at room temperature for 18 h. After workup, the crude product was purified by flash column chromatography (SiO_2, 20% ethyl acetate in n-hexane) to give an inseparable mixture of 2-ethyl 3-methyl (3*S*)-6-methyl-3,4-dihydroisoquinoline-2,3(1*H*)-dicarboxylate and 2-ethyl 3-methyl (3*S*)-8-methyl-3,4-dihydroisoquinoline-2,3(1*H*)-dicarboxylate **14d** as a colorless oil (8.17 g, 66%). ^{1}H NMR (400 MHz, DMSO-d_6) δ 7.13–6.89 (m, 3H), 5.06–4.80 (m, 1H), 4.65–4.48 (m, 1H), 4.47–4.24 (m, 1H), 4.20–3.99 (m, 2H), 3.53 (s, 1.5H), 3.52 (s, 1.5H), 3.15–3.05 (m, 2H), 2.25–2.14 (m, 3H), 1.27–1.11 (m, 3H).

(3*S*)-6-Methyl-1,2,3,4-tetrahydroisoquinoline-3-carboxylic acid hydrochloride (1:1) and (3*S*)-8-methyl-1,2,3,4-tetrahydroisoquinoline-3-carboxylic acid hydrochloride (1:1) (14e)

The compound was prepared by general procedure E with 2-ethyl 3-methyl (3*S*)-6-methyl-3,4-dihydroisoquinoline-2,3(1*H*)-dicarboxylate and 2-ethyl 3-methyl (3*S*)-8-methyl-3,4-dihydroisoquinoline-2,3(1*H*)-dicarboxylate **14d** (8.17 g, 29.5 mmol) in aqueous 8 N hydrogen chloride (118 mL), refluxed for 48 h. The inseparable mixture of

(3*S*)-6-methyl-1,2,3,4-tetrahydroisoquinoline-3-carboxylic acid hydrochloride (1:1) and

(3*S*)-8-methyl-1,2,3,4-tetrahydroisoquinoline-3-carboxylic acid hydrochloride (1:1) **14e** was obtained as a white solid (the ratio is 88:12, 3.59 g, 54%). The ^{1}H NMR spectrum of

(3*S*)-6-methyl-1,2,3,4-tetrahydroisoquinoline-3-carboxylic acid hydrochloride (1:1) is listed. ^{1}H NMR (600 MHz, DMSO-d_6) δ 14.14 (br s, 1H), 9.77 (br s, 1H), 7.13 (d, 1H), 7.09–7.06 (m, 2H), 4.37 (dd, 1H), 4.31–4.22 (m, 2H), 3.25 (dd, 1H), 3.08 (dd, 1H), 2.27 (s, 3H).

Methyl (3*S*)-6-methyl-1,2,3,4-tetrahydroisoquinoline-3-carboxylate and Methyl (3*S*)-8-methyl-1,2,3,4-tetrahydroisoquinoline-3-carboxylate (14f)

The compound was prepared by general procedure G with

(3*S*)-6-methyl-1,2,3,4-tetrahydroisoquinoline-3-carboxylic acid hydrochloride (1:1) and

(3*S*)-8-methyl-1,2,3,4-tetrahydroisoquinoline-3-carboxylic acid hydrochloride (1:1) **14e** (3.57 g, 15.7 mmol), thionyl chloride (3.99 mL, 54.9 mmol) and triethylamine (2.40 mL, 17.2 mmol) in anhydrous methanol (157 mL), refluxed for 18 h. The crude product was purified by flash column chromatography (SiO_2, 0.5−1% methnol in dichloromethane) to give an inseparable mixture of methyl

(3*S*)-6-methyl-1,2,3,4-tetrahydroisoquinoline-3-carboxylate and methyl

(3*S*)-8-methyl-1,2,3,4-tetrahydroisoquinoline-3-carboxylate **14f** as a colorless oil (the ratio is 90:10, 1.44 g, 45%). The spectrum of methyl (3*S*)-6-methyl-1,2,3,4-tetrahydroisoquinoline-3-carboxylate is listed. ^{1}H NMR (600 MHz, DMSO-d_6) δ 6.93–6.89 (m, 3H), 3.90 (d, 1H), 3.83 (d, 1H), 3.67–3.62 (m, 4H), 2.89 (dd, 1H), 2.77 (dd, 1H), 2.71 (br s, 1H), 2.23 (s, 3H).

(3*S*)-6-Methyl-1,2,3,4-tetrahydroisoquinoline-3-carboxamide (14g)

The compound was prepared by general procedure H with methyl

(3*S*)-6-methyl-1,2,3,4-tetrahydroisoquinoline-3-carboxylate and

(3*S*)-8-methyl-1,2,3,4-tetrahydroisoquinoline-3-carboxylate **14f** (1.43 g, 7.00 mmol) in 28–30% aqueous ammonium hydroxide (140 mL), stirred at room temperature for 18 h. The desired product

(3*S*)-6-methyl-1,2,3,4-tetrahydroisoquinoline-3-carboxamide **14g** was obtained as a white solid (803 mg, 60%). ^{1}H NMR (600 MHz, DMSO-d_6) δ 7.31 (br s, 1H), 7.05 (br s, 1H), 6.92–6.88 (m, 3H), 3.87 (d, 1H), 3.81 (d, 1H), 2.83 (dd, 1H), 2.67 (dd, 1H), 2.53–2.51 (m overlapped with DMSO, 1H), 2.23 (s, 3H).

(3*S*)-2,6-Dimethyl-1,2,3,4-tetrahydroisoquinoline-3-carboxamide (14h)

The compound was prepared by general procedure I with (3*S*)-6-methyl-1,2,3,4-tetrahydroisoquinoline-3-carboxamide **14g** (803 mg, 4.22 mmol), 37% aqueous formaldehyde (0.510 mL, 6.33 mmol), sodium triacetoxyborohydride (1.79 g, 8.44 mmol) and acetic acid (0.240 mL, 4.22 mmol) in tetrahydrofuran (21 mL), stirred at room temperature for 5 h. The desired product (3*S*)-2,6-dimethyl-1,2,3,4-tetrahydroisoquinoline-3-carboxamide **14h** was obtained as a white solid (813 mg, 94%). ^{1}H NMR (400 MHz, DMSO-d_6) δ 7.37 (br s, 1H), 7.04 (br s, 1H), 6.94–6.89 (m, 3H), 3.83 (d, 1H), 3.42 (d, 1H), 3.01 (dd, 1H), 2.89 (dd, 1H), 2.79 (dd, 1H), 2.30 (s, 3H), 2.23 (s, 3H).

((3*S*)-2,6-Dimethyl-1,2,3,4-tetrahydroisoquinolin-3-yl)methanamine (14i)

The compound was prepared by general procedure J with (3*S*)-2,6-dimethyl-1,2,3,4-tetrahydroisoquinoline-3-carboxamide **14h** (150 mg, 0.730 mmol) and 1 M lithium aluminum hydride solution in THF (2.20 mL, 2.20 mmol) in anhydrous tetrahydrofuran (15 mL), stirred at 60 ˚C for 18 h. After workup, the crude product was purified by flash column chromatography (SiO_2, 20% methanol with 1% triethylamine in dichloromethane) to give the desired product ((3*S*)-2,6-dimethyl-1,2,3,4-tetrahydroisoquinolin-3-yl)methanamine **14i** as a yellow crystal (110 mg, 81%). ^{1}H NMR (600 MHz, DMSO-d_6) δ 6.92–6.89 (m, 2H), 6.88 (s, 1H), 3.68 (d, 1H), 3.46 (d, 1H), 2.72–2.66 (m, 3H), 2.55 (dd, 1H), 2.47–2.43 (m, 1H), 2.28 (s, 3H), 2.23 (s, 3H), 1.43 (br s, 2H).

Methyl (2*R*)-2-amino-3-(3-methylphenyl)propanoate hydrochloride (1:1) (15b)

The compound was prepared by general procedure A with (2*R*)-2-amino-3-(3-methylphenyl)propanoic acid **15a** (5.00 g, 27.9 mmol) and thionyl chloride (2.23 mL, 30.7 mmol) in anhydrous methanol (40 mL), refluxed for 16 h. The desired product methyl (2*R*)-2-amino-3-(3-methylphenyl)propanoate hydrochloride (1:1) **15b** was obtained as a white solid (6.41 g, quantitative yield). ^{1}H NMR (400 MHz, CD_3OD) δ 7.26 (t, 1H), 7.15 (d, 1H), 7.08 (s, 1H), 7.04 (d, 1H), 4.31 (dd, 1H), 3.81 (s, 3H), 3.23 (dd, 1H), 3.11 (dd, 1H), 2.35 (s, 3H).

Methyl (2*R*)-2-[(methoxycarbonyl)amino]-3-(3-methylphenyl)propanoate (15c)

The compound was prepared by general procedure B with methyl (2*R*)-2-amino-3-(3-methylphenyl) propanoate hydrochloride (1:1) **15b** (7.45 g, 32.5 mmol), methyl chloroformate (2.63 mL, 34.1 mmol) and sodium bicarbonate (6.00 g, 71.4 mmol) in dichloromethane (81.1 mL), stirred at room temperature for 16

h. The desired product methyl (2*R*)-2-[(methoxycarbonyl)amino]-3-(3-methylphenyl)propanoate **15c** was obtained as a yellow liquid (7.39 g, 91%). ^{1}H NMR (400 MHz, $CDCl_3$) δ 7.18 (t, 1H), 7.06 (d, 1H), 6.94–6.89 (m, 2H), 5.12 (br d, 1H), 4.62 (br q, 1H), 3.72 (s, 3H), 3.66 (s, 3H), 3.12–2.98 (m, 2H), 2.32 (s, 3H).

Dimethyl (3*R*)-6-methyl-3,4-dihydroisoquinoline-2,3(1*H*)-dicarboxylate and dimethyl (3*R*)-6-methyl-3,4-dihydroisoquinoline-2,3(1*H*)-dicarboxylate (15d)

The compound was prepared by general procedure D with methyl (2*R*)-2-[(methoxycarbonyl)amino]-3-(3-methylphenyl)propanoate **15c** (7.39 g, 29.4 mmol) and paraformaldehyde (1.33 g, 44.1 mmol) in a mixture of acetic acid (36.8 mL) and sulfuric acid (3.14 mL), stirred at room temperature for 16 h. The inseparable mixture of dimethyl (3*R*)-6-methyl-3,4-dihydroisoquinoline-2,3(1*H*)-dicarboxylate and dimethyl (3*R*)-6-methyl-3,4-dihydroisoquinoline-2,3(1*H*)-dicarboxylate **15d** was obtained as a yellow liquid (5.20 g, 67%). ^{1}H NMR (400 MHz, $CDCl_3$) δ 7.11–6.93 (m, 3H), 5.23–5.11 (m, 0.6H), 5.03–4.90 (m, 0.4H), 4.78–4.63 (m, 1H), 4.55–4.37 (m, 1H), 3.83–3.71 (m, 3H), 3.62 (s, 3H), 3.30–3.08 (m, 2H), 2.33–2.21 (m, 3H).

(3*R*)-6-Methyl-1,2,3,4-tetrahydroisoquinoline-3-carboxylic acid hydrochloride (1:1) (15e)

The compound was prepared by general procedure E with dimethyl (3*R*)-6-methyl-3,4-dihydroisoquinoline-2,3(1*H*)-dicarboxylate and dimethyl (3*R*)-6-methyl-3,4-dihydroisoquinoline-2,3(1*H*)-dicarboxylate **15d** (5.20 g, 19.8 mmol) in aqueous 6 N hydrogen chloride (65.8 mL), refluxed for 48 h. The suspension was filtered at 60 ˚C, the precipitate was dried under high vacuum to afford the desired product (3*R*)-6-methyl-1,2,3,4-tetrahydroisoquinoline-3-carboxylic acid hydrochloride (1:1) **15e** as a white solid (1.68 g, 37%). ^{1}H NMR (400 MHz, CD_3OD) δ 7.14–7.10 (m, 3H), 4.45–4.34 (m, 3H), 3.43 (dd, 1H), 3.19 (dd, 1H), 2.33 (s, 3H).

Methyl (3*R*)-6-methyl-1,2,3,4-tetrahydroisoquinoline-3-carboxylate (15f)

The compound was prepared by general procedure G with (3*R*)-6-methyl-1,2,3,4-tetrahydroisoquinoline-3-carboxylic acid hydrochloride (1:1) **15e** (1.68 g, 7.38 mmol), thionyl chloride (1.87 mL, 25.8 mmol) and triethylamine (1.13 mL, 8.12 mmol) in anhydrous methanol (24.6 mL), stirred at 50 °C for 16 h. After workup, the crude product was purified by flash column chromatography (SiO_2, dichloromethane/

methanol = 99/1) to give the desired product methyl (3*R*)-6-methyl-1,2,3,4-tetrahydroisoquinoline-3-carboxylate **15f** as a yellow liquid (1.04 g, 69%). ^{1}H NMR (400 MHz, $CDCl_3$) δ 6.99–6.91 (m, 3H), 4.10 (d, 1H), 4.04 (d, 1H), 3.78 (s, 3H), 3.73 (dd, 1H), 3.04 (dd, 1H), 2.92 (dd, 1H), 2.30 (s, 3H).

(3*R*)-6-Methyl-1,2,3,4-tetrahydroisoquinoline-3-carboxamide (15g)

The compound was prepared by general procedure H with methyl (3*R*)-6-methyl-1,2,3,4-tetrahydroisoquinoline-3-carboxylate **15f** (1.04 g, 5.09 mmol) in 28–30% aqueous ammonium hydroxide (50.9 mL), stirred at room temperature for 16 h. The desired product (3*R*)-6-methyl-1,2,3,4-tetrahydroisoquinoline-3-carboxamide **15g** was obtained as a white solid (0.830 g, 86%). ^{1}H NMR (400 MHz, CD_3OD) δ 6.97–6.91 (m, 3H), 4.01 (d, 1H), 3.94 (d, 1H), 3.54 (dd, 1H), 2.99 (dd, 1H), 2.85 (dd, 1H), 2.27 (s, 3H).

(3*R*)-2,6-Dimethyl-1,2,3,4-tetrahydroisoquinoline-3-carboxamide (15h)

The compound was prepared by general procedure I with (3*R*)-6-methyl-1,2,3,4-tetrahydroisoquinoline-3-carboxamide **15g** (0.830 g, 4.36 mmol), 37% aqueous formaldehyde (0.490 mL, 6.54 mmol), sodium triacetoxyborohydride (1.85 g, 8.72 mmol) and acetic acid (0.250 mL, 4.36 mmol) in acetonitrile (21.8 mL) and tetrahydrofuran (21.8 mL), stirred at room temperature for 16 h. The desired product (3*R*)-2,6-dimethyl-1,2,3,4-tetrahydroisoquinoline-3-carboxamide **15h** was obtained as a white solid (0.884 g, 99%). ^{1}H NMR (400 MHz, $CDCl_3$) δ 7.05–6.94 (m, 4H), 5.57 (br s, 1H), 3.84 (d, 1H), 3.68 (d, 1H), 3.28 (t, 1H), 3.04 (d, 2H), 2.44 (s, 3H), 2.31 (s, 3H).

[(3*R*)-2,6-Dimethyl-1,2,3,4-tetrahydroisoquinolin-3-yl]methanamine (15i)

The compound was prepared by general procedure J with (3*R*)-2,6-dimethyl-1,2,3,4-tetrahydroisoquinoline-3-carboxamide **15h** (0.748 g, 3.66 mmol) and lithium aluminum hydride (0.417 g, 11.0 mmol) in anhydrous tetrahydrofuran (36.6 mL), refluxed for 48 h. After workup, the crude product was purified by flash column chromatography (SiO_2, dichloromethane/methanol/ammonium hydroxide solution = 92/7.6/0.4) to give the desired product [(3*R*)-2,6-dimethyl-1,2,3,4-tetrahydroisoquinolin-3-yl]methanamine **15i** as a yellow liquid (0.430 g, 62%). ^{1}H NMR (400 MHz, $CDCl_3$) δ 6.97–6.88 (m, 3H), 3.81 (d, 1H), 3.67 (d, 1H), 2.89–2.79 (m, 2H), 2.76–2.64 (m, 3H), 2.39 (s, 3H), 2.29 (s, 3H).

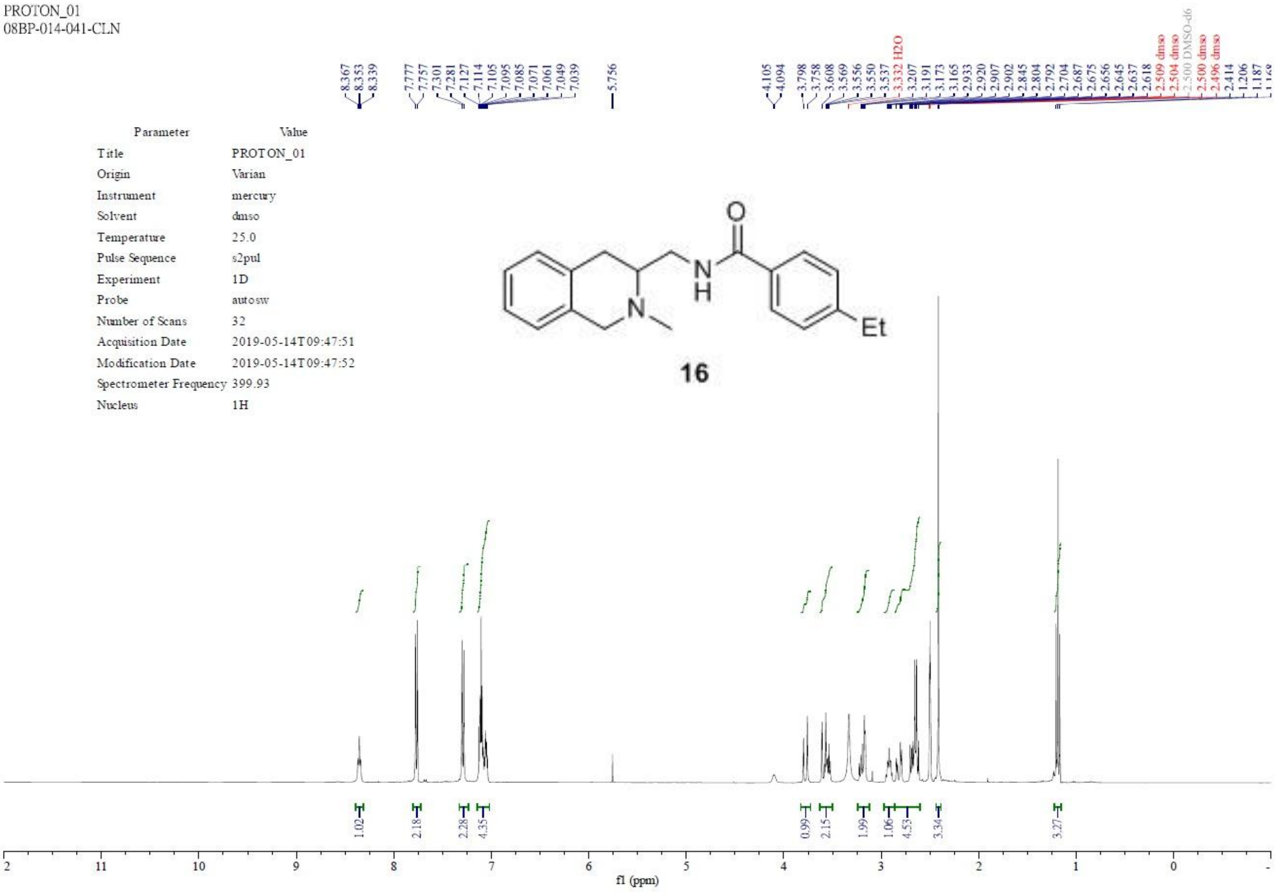

^{1}H NMR spectrum of compound **16**

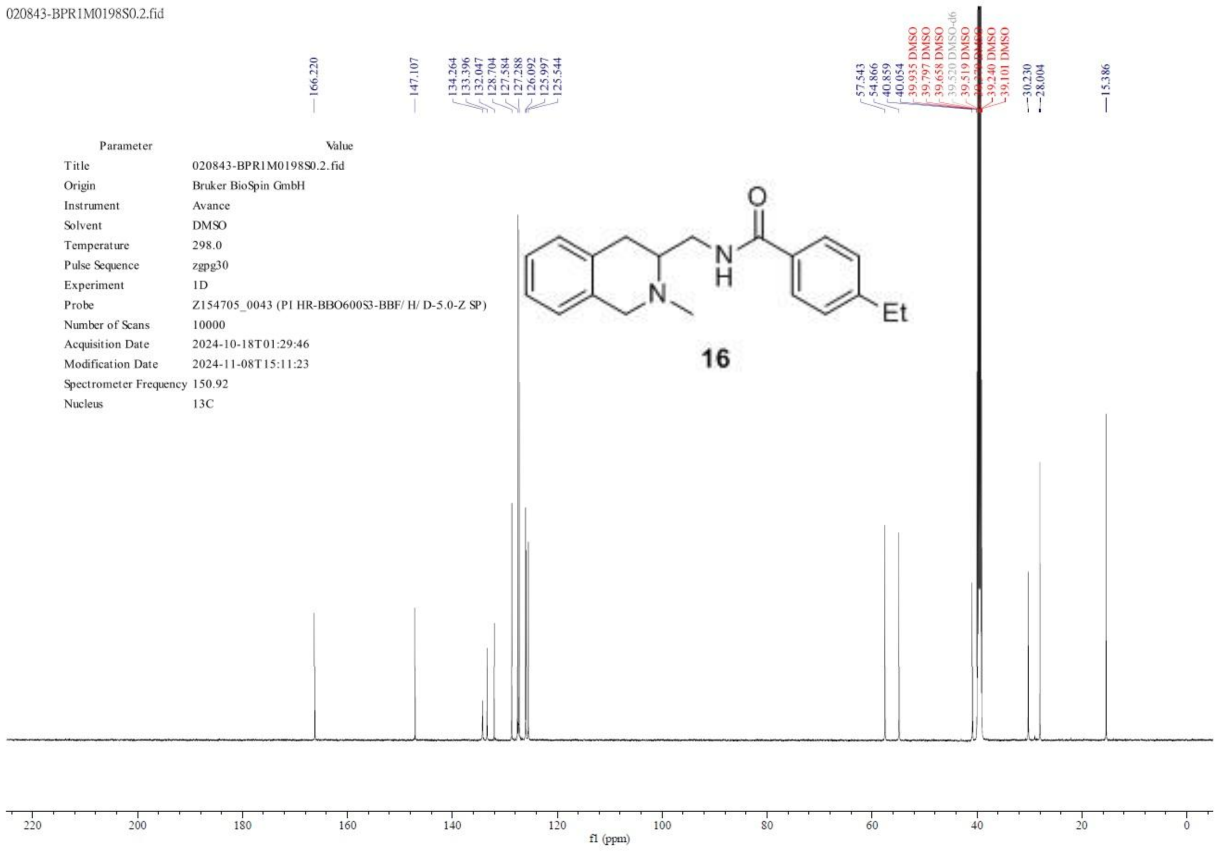

^{13}C NMR spectrum of compound **16**

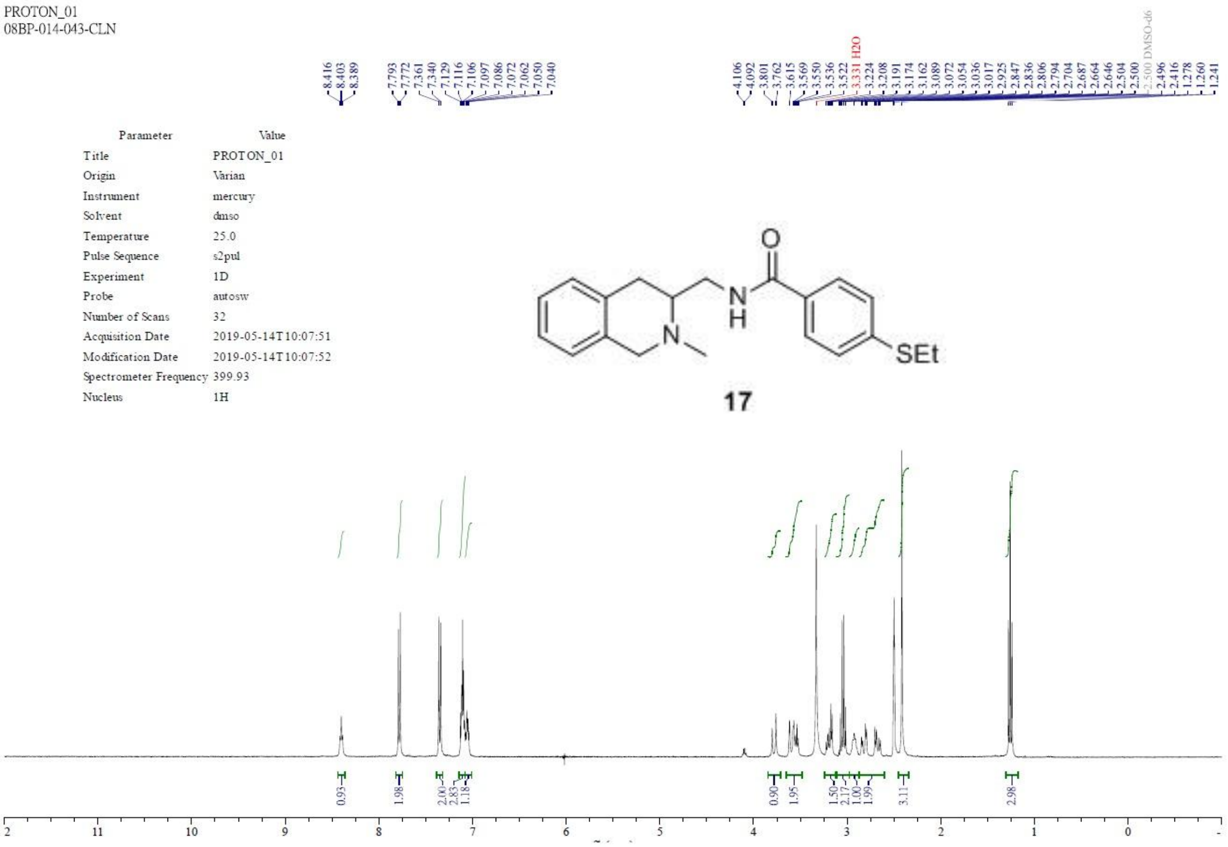

^{1}H NMR spectrum of compound **17**

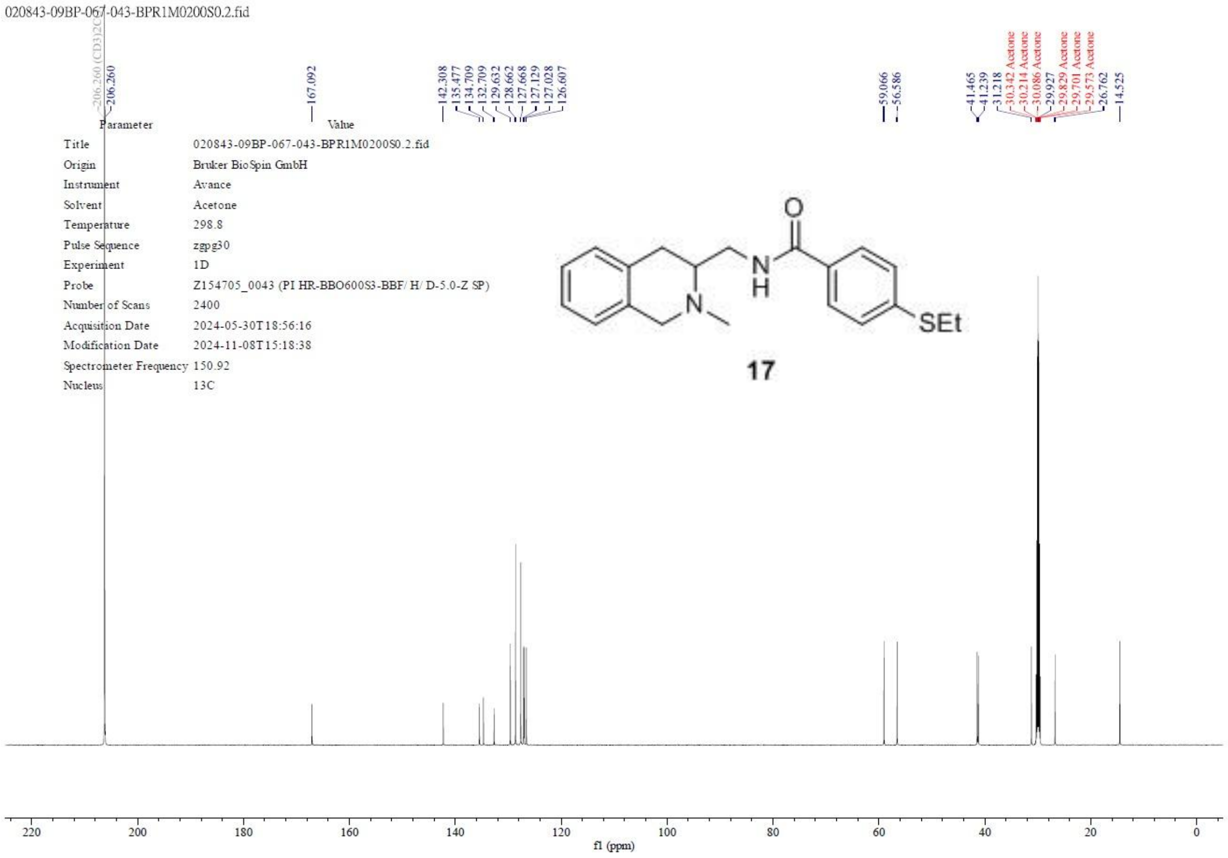

^{13}C NMR spectrum of compound **17**

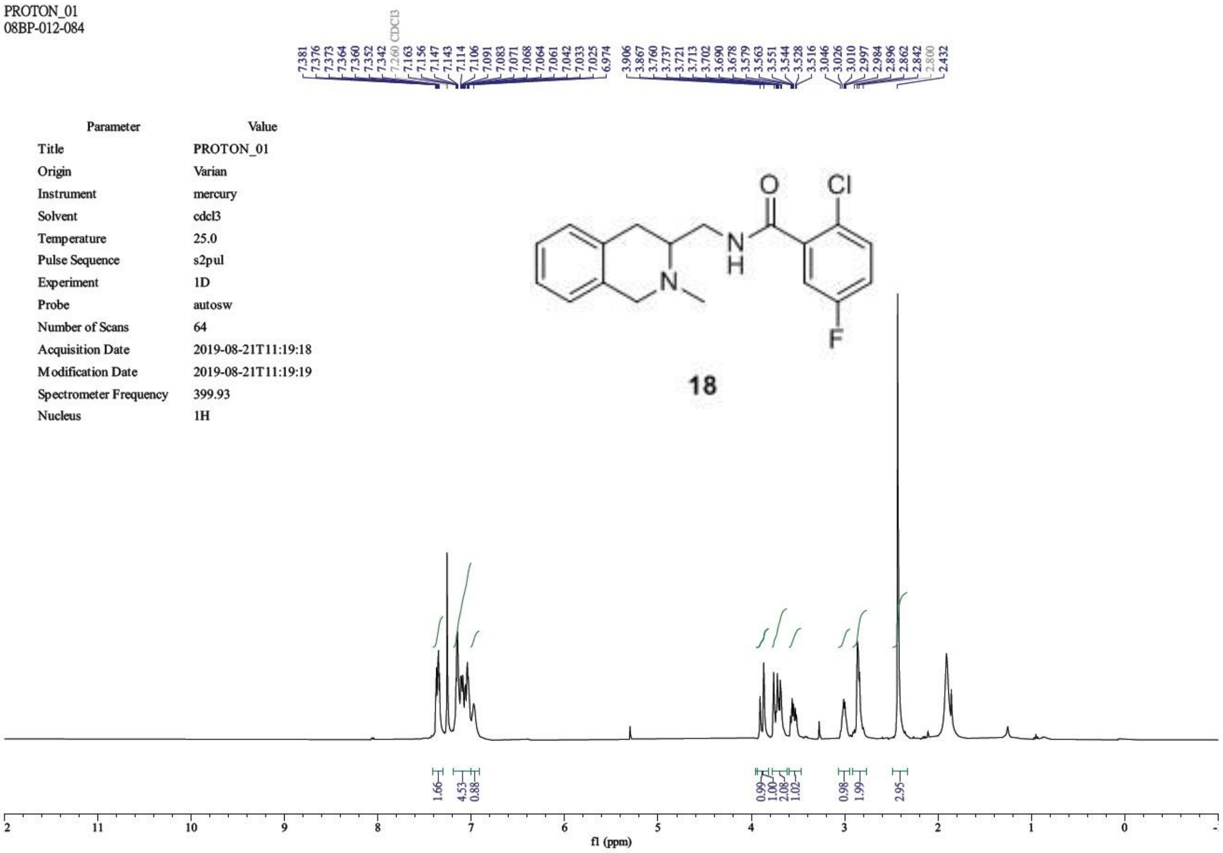

^{1}H NMR spectrum of compound **18**

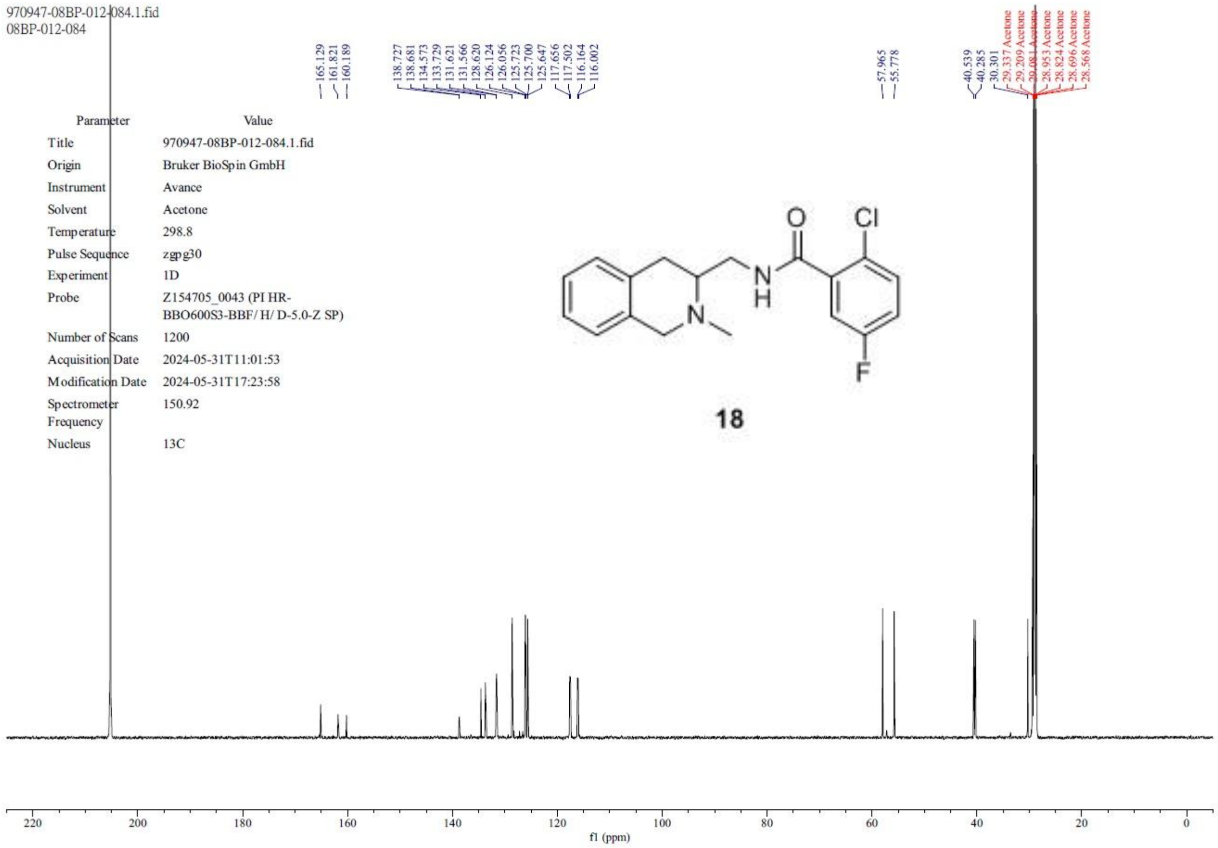

^{13}C NMR spectrum of compound **18**

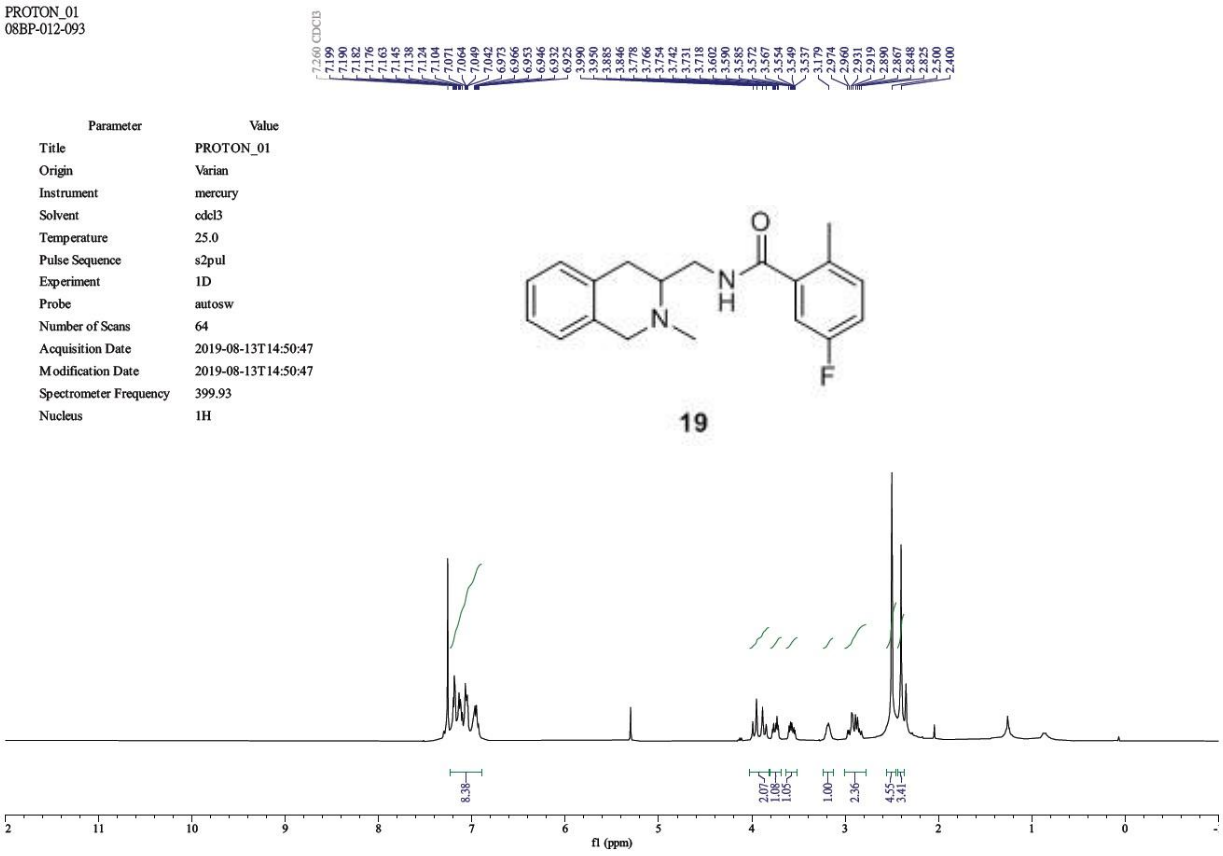

^{1}H NMR spectrum of compound **19**

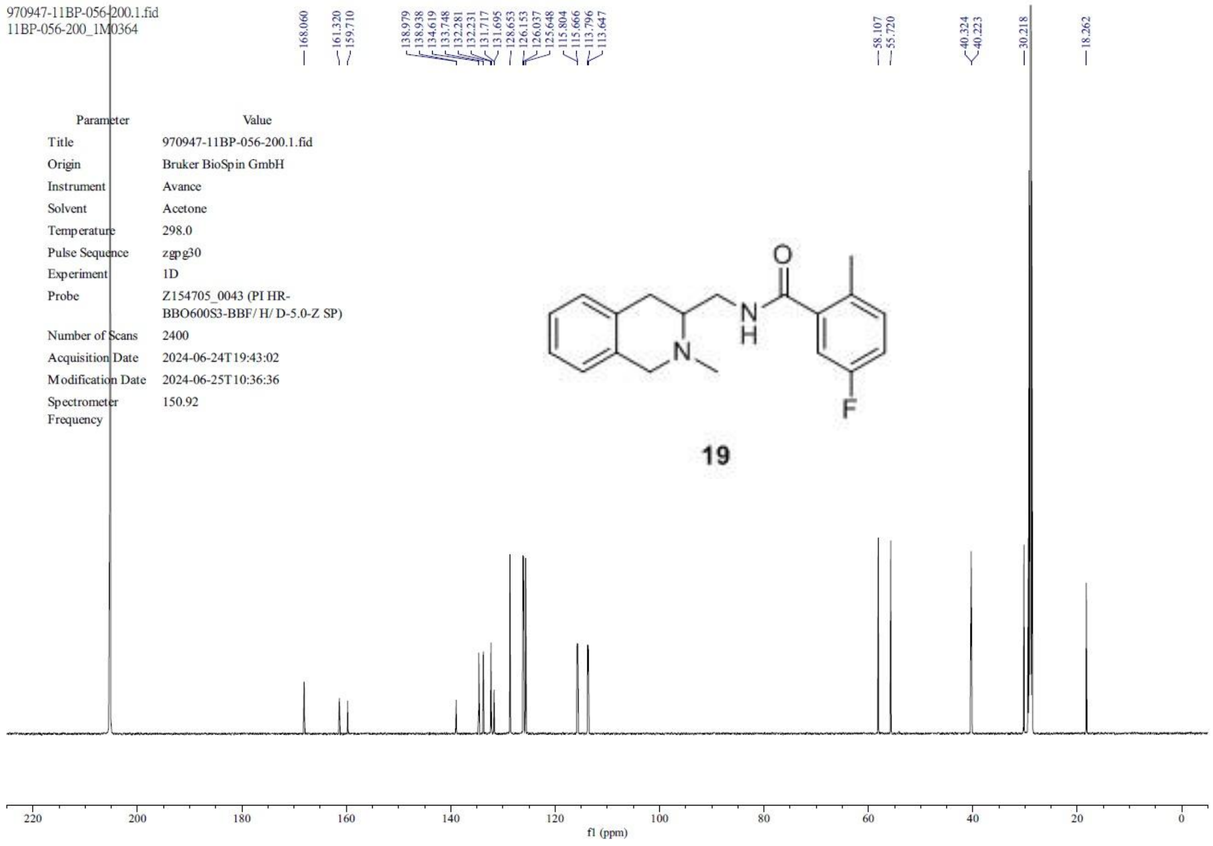

^{13}C NMR spectrum of compound **19**

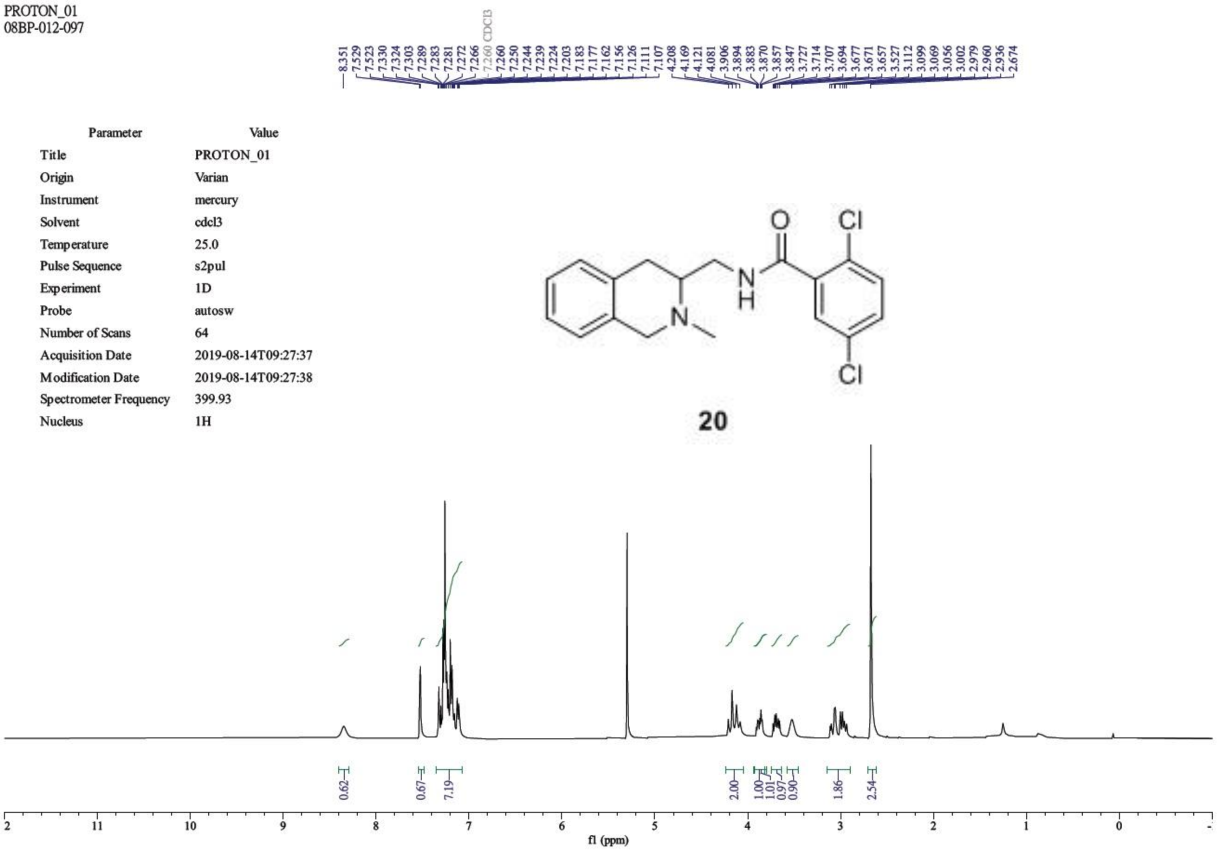

^{1}H NMR spectrum of compound **20**

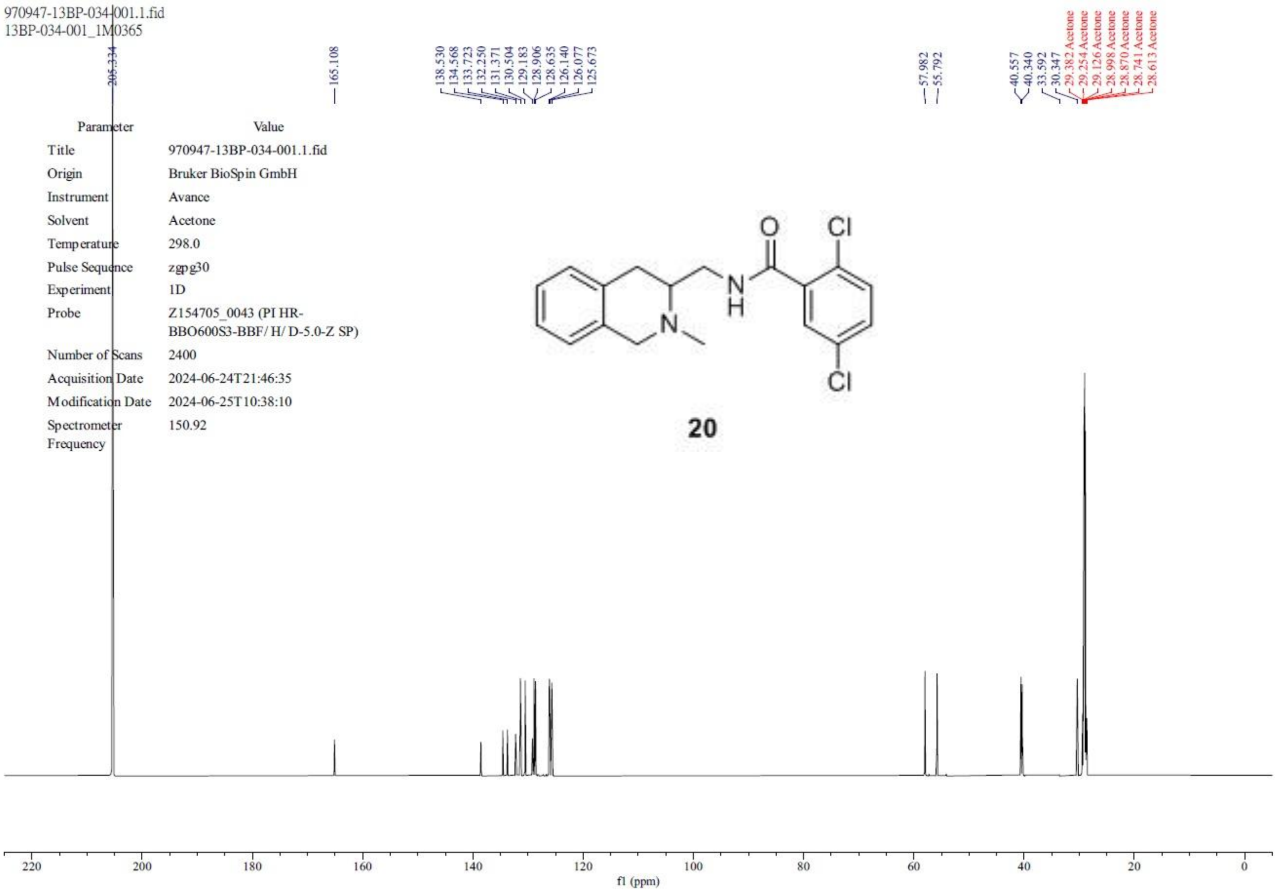

^{13}C NMR spectrum of compound **20**

PROTON_01
08BP-012-100

Parameter	Value
Title	PROTON_01
Origin	Varian
Instrument	mercury
Solvent	cdcl3
Temperature	25.0
Pulse Sequence	s2pul
Experiment	1D
Probe	autosw
Number of Scans	64
Acquisition Date	2019-08-19T09:24:38
Modification Date	2019-08-19T09:24:38
Spectrometer Frequency	399.93
Nucleus	1H

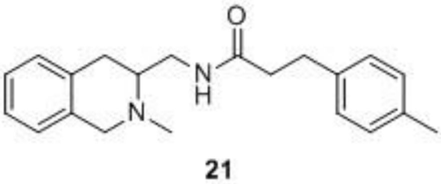

21

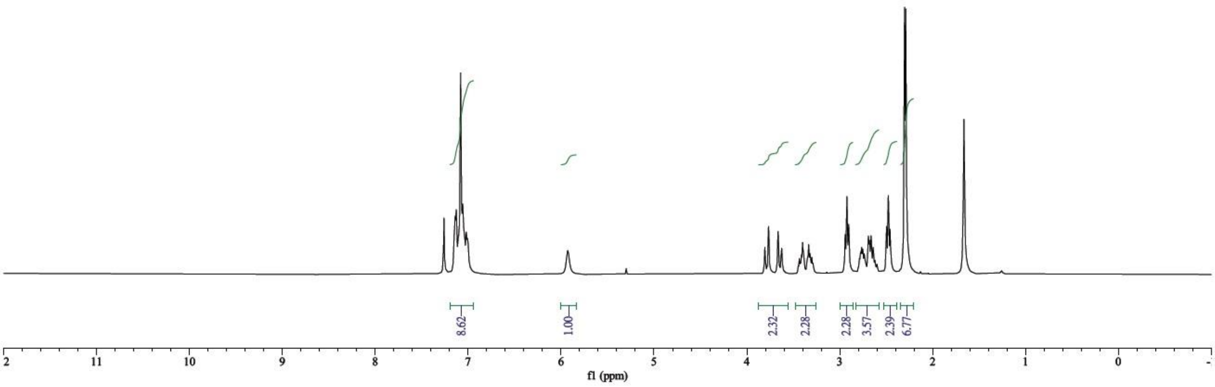

^{1}H NMR spectrum of compound **21**

970947-08BP-012-100.1.fid
08BP-012-100_1M0367

Parameter	Value
Title	970947-08BP-012-100.1.fid
Origin	Bruker BioSpin GmbH
Instrument	Avance
Solvent	Acetone
Temperature	298.0
Pulse Sequence	zgpg30
Experiment	1D
Probe	Z154705_0043 (PI HR-BBO600S3-BBF/ H/ D-5.0-Z SP)
Number of Scans	2400
Acquisition Date	2024-06-27T19:48:26
Modification Date	2024-06-28T09:19:11
Spectrometer Frequency	150.92
Nucleus	13C

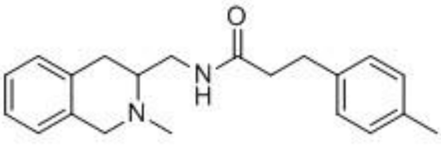

21

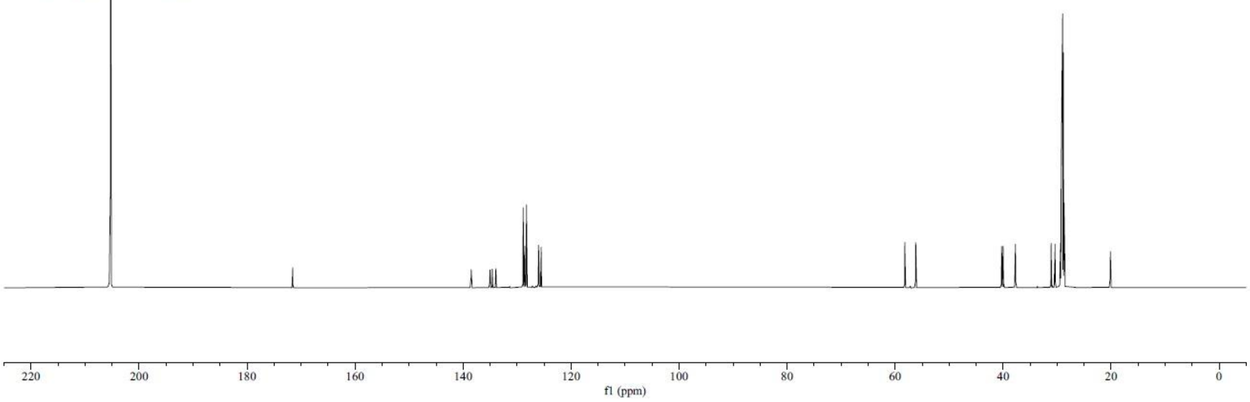

^{13}C NMR spectrum of compound **21**

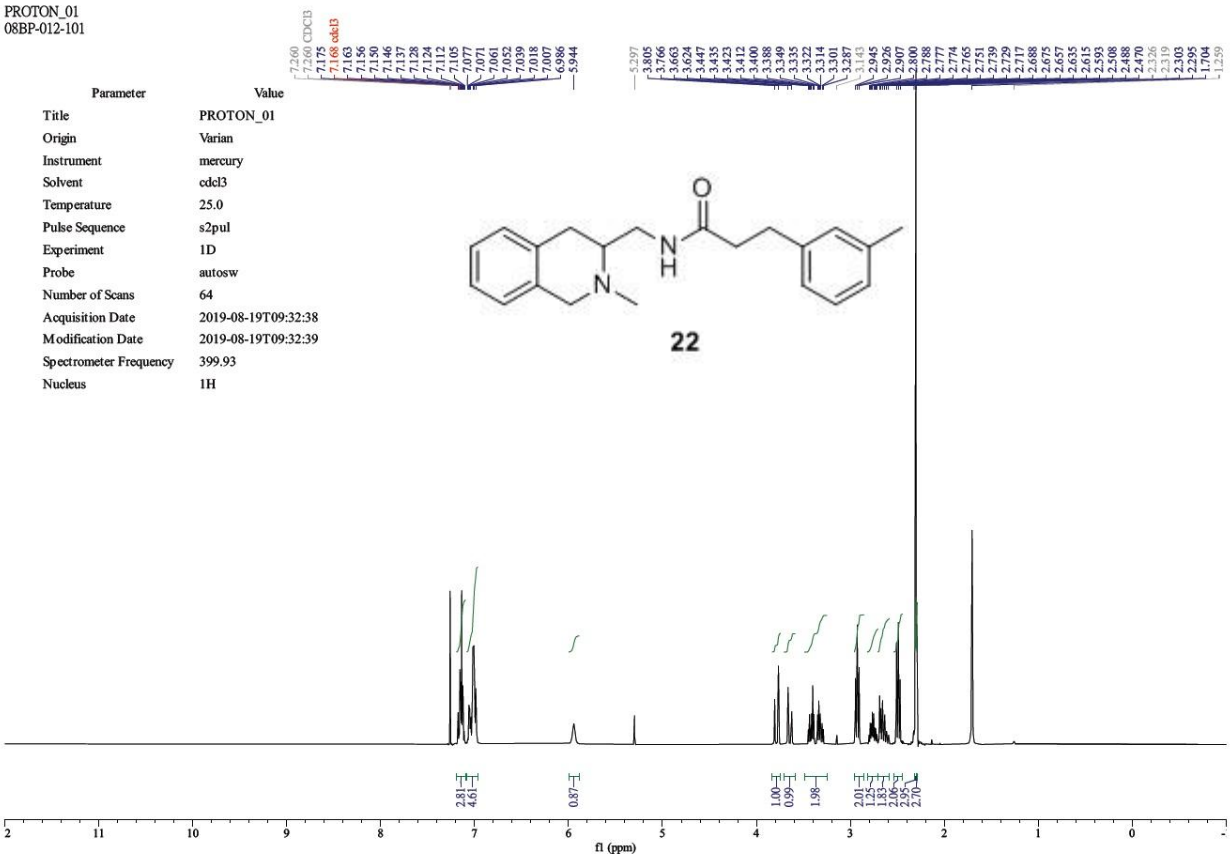

^{1}H NMR spectrum of compound **22**

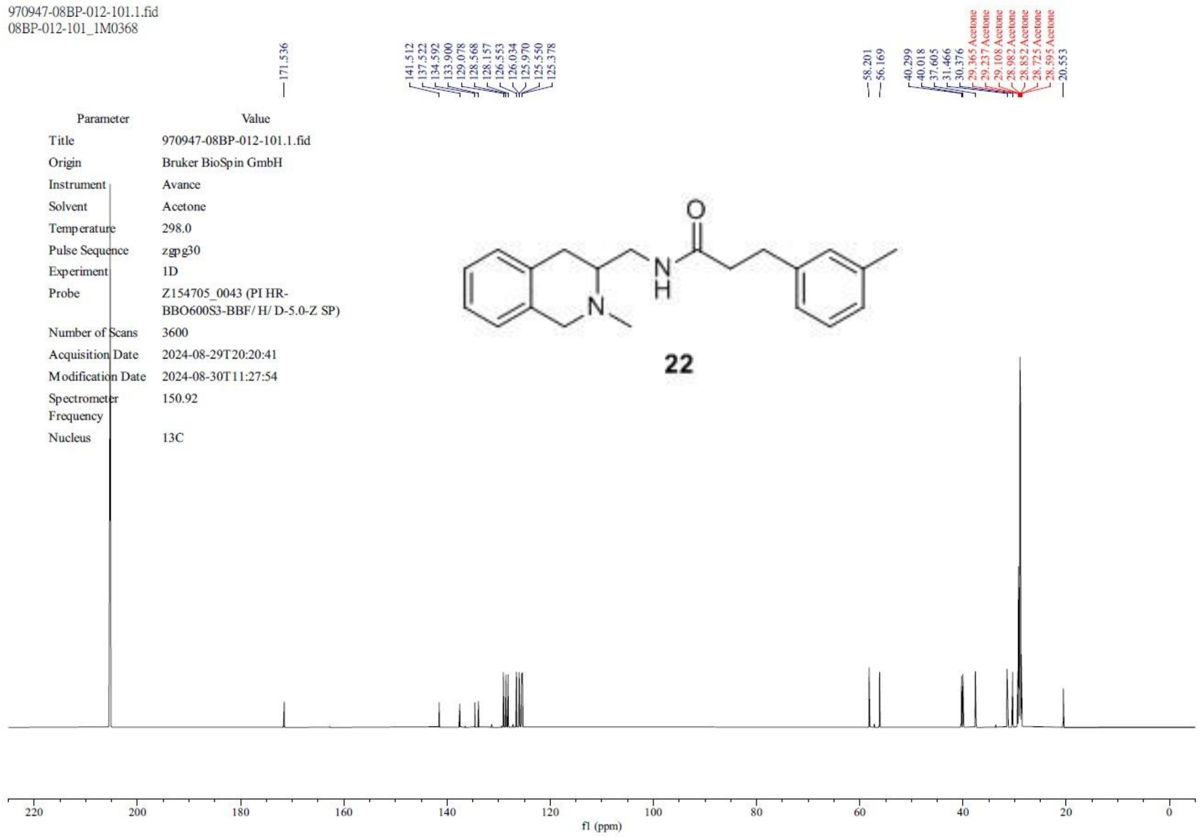

^{13}C NMR spectrum of compound **22**

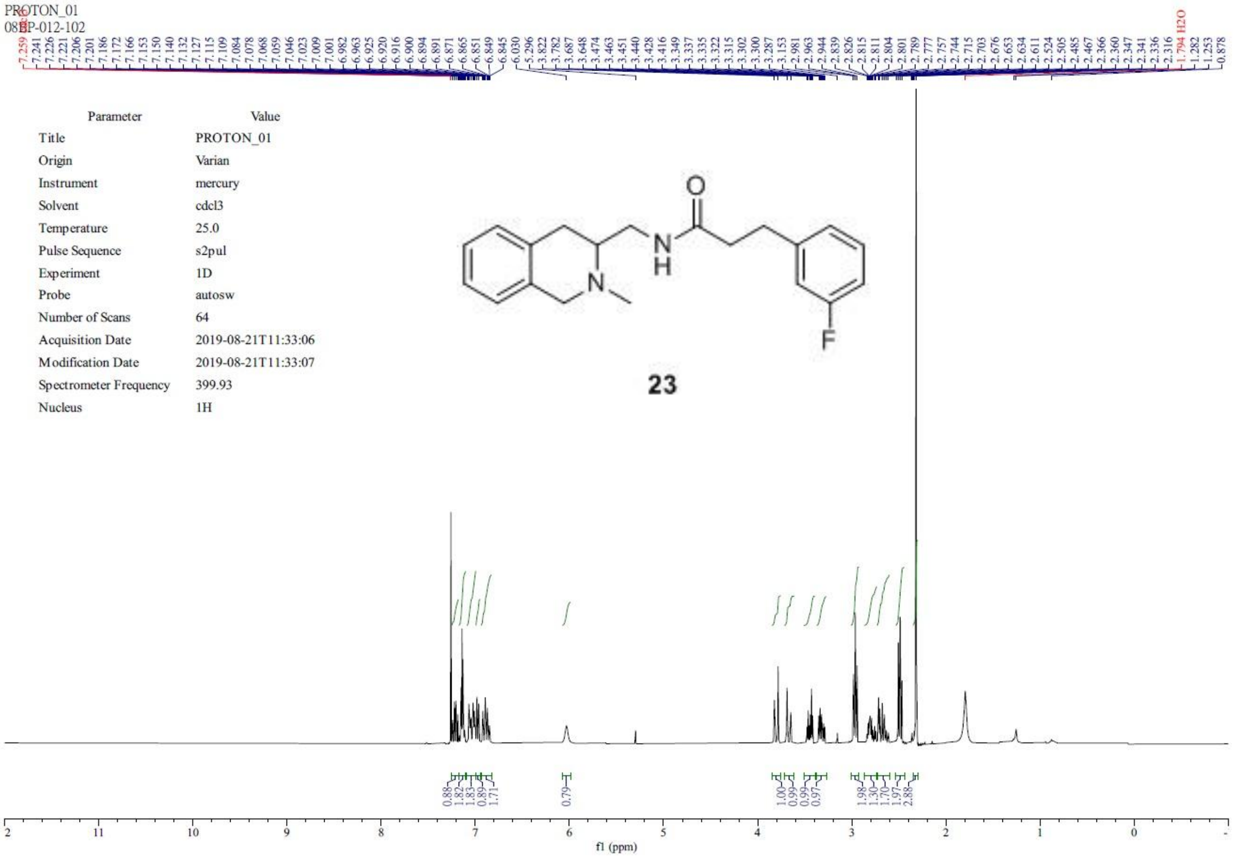

^{1}H NMR spectrum of compound **23**

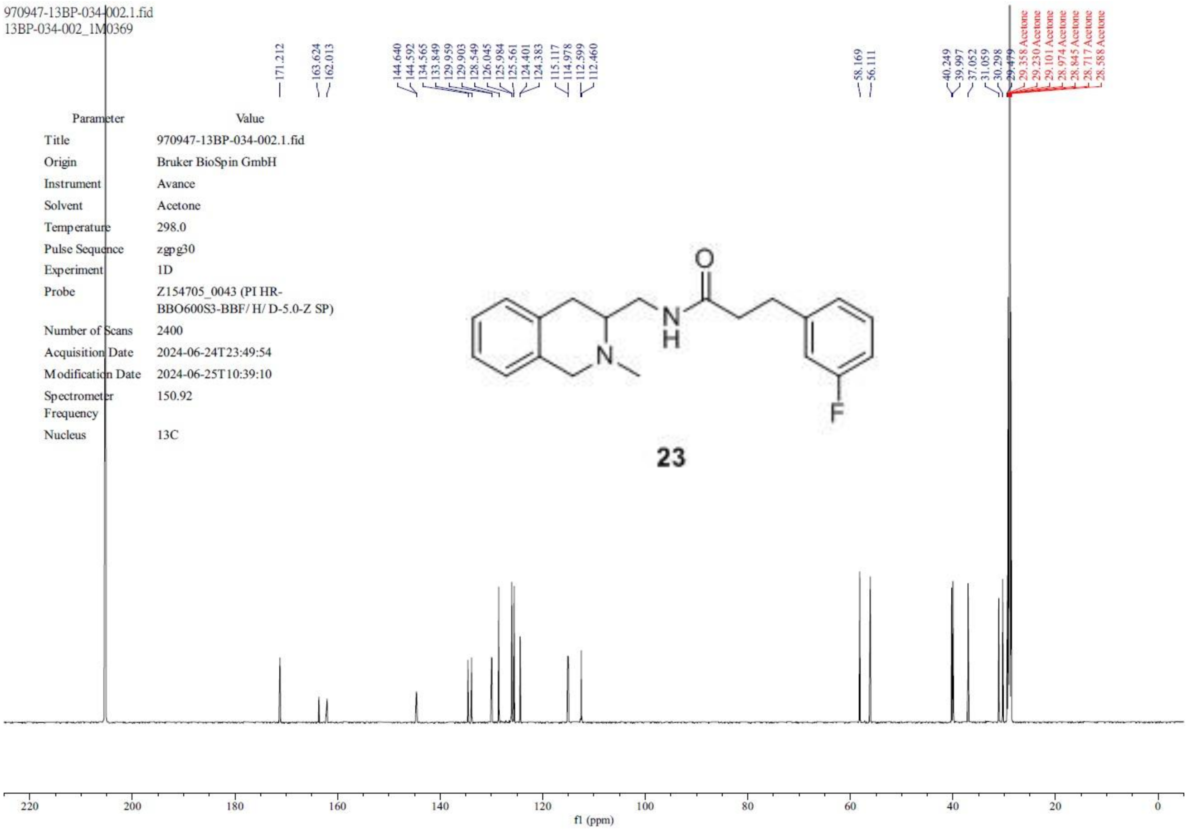

^{13}C NMR spectrum of compound **23**

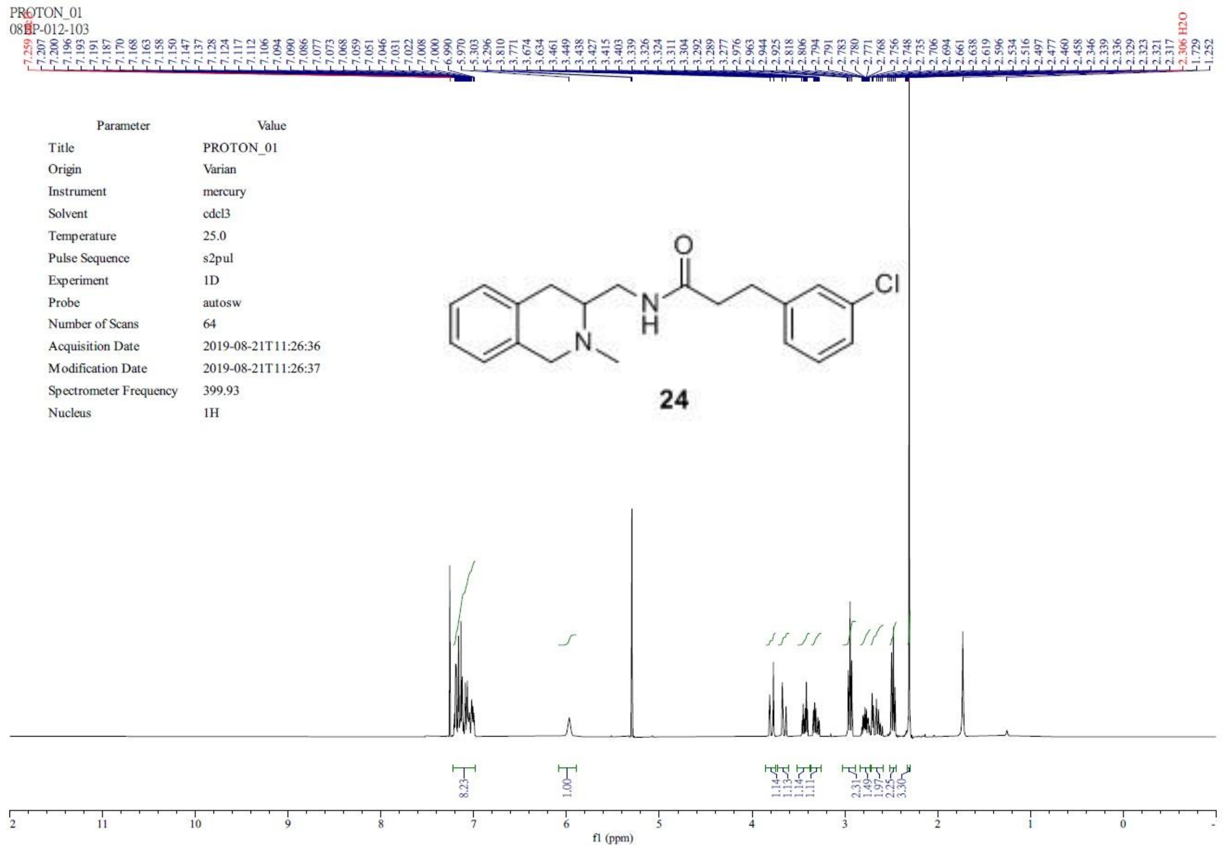

^{1}H NMR spectrum of compound **24**

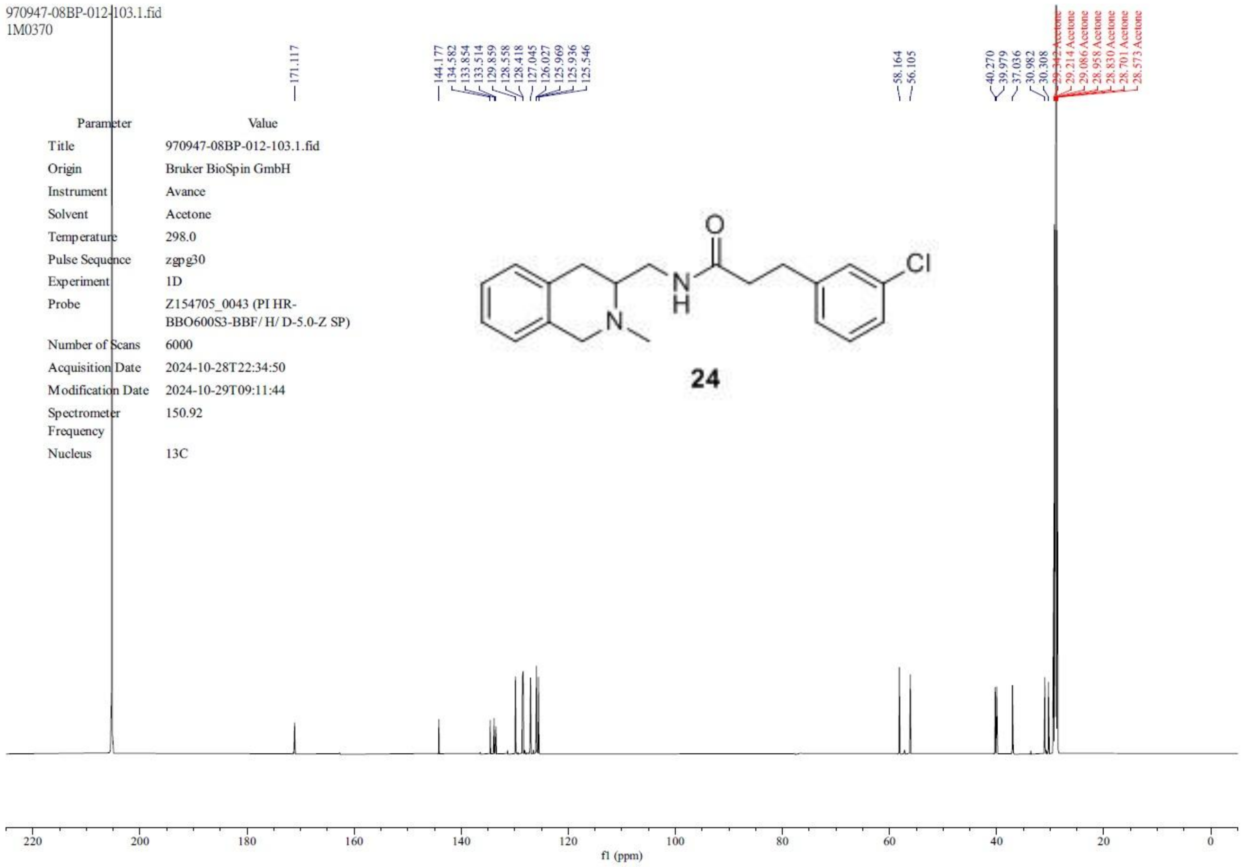

^{13}C NMR spectrum of compound **24**

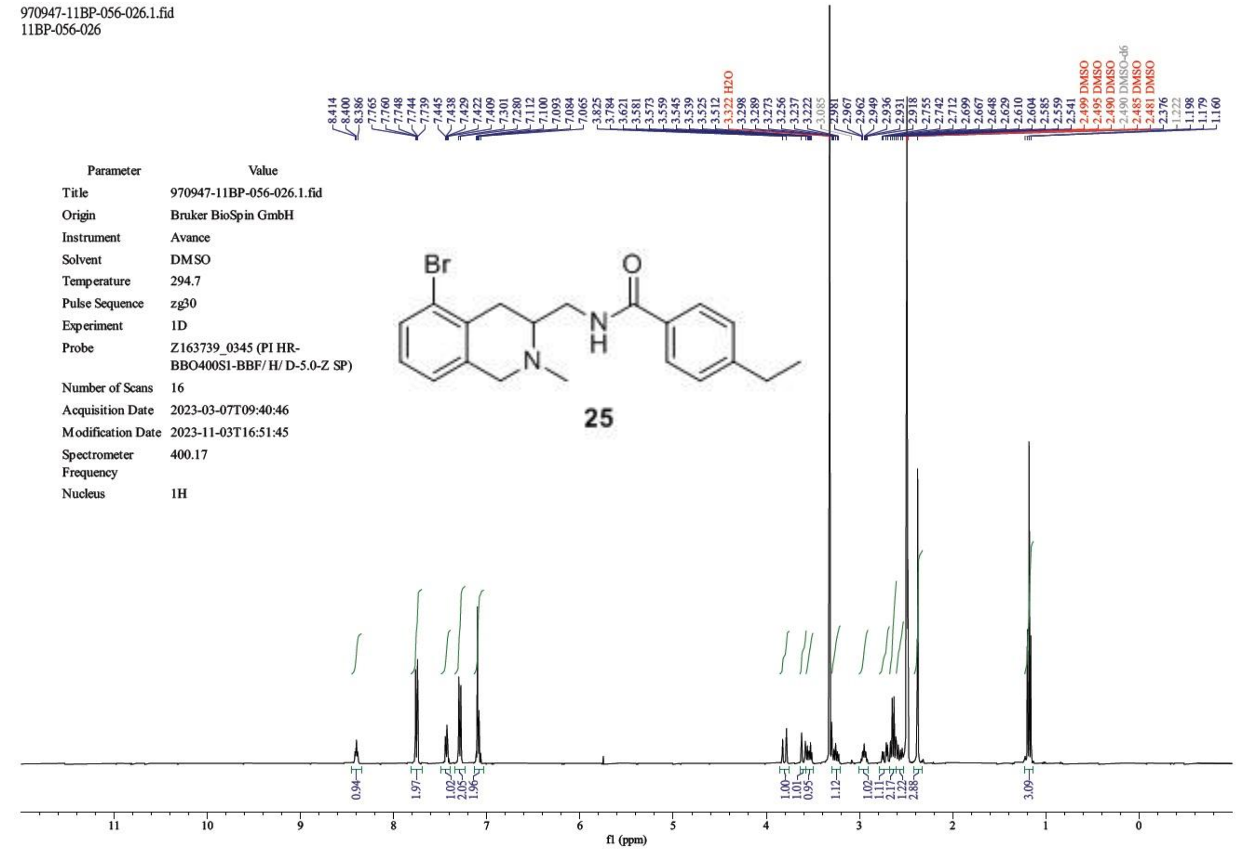

^{1}H NMR spectrum of compound **25**

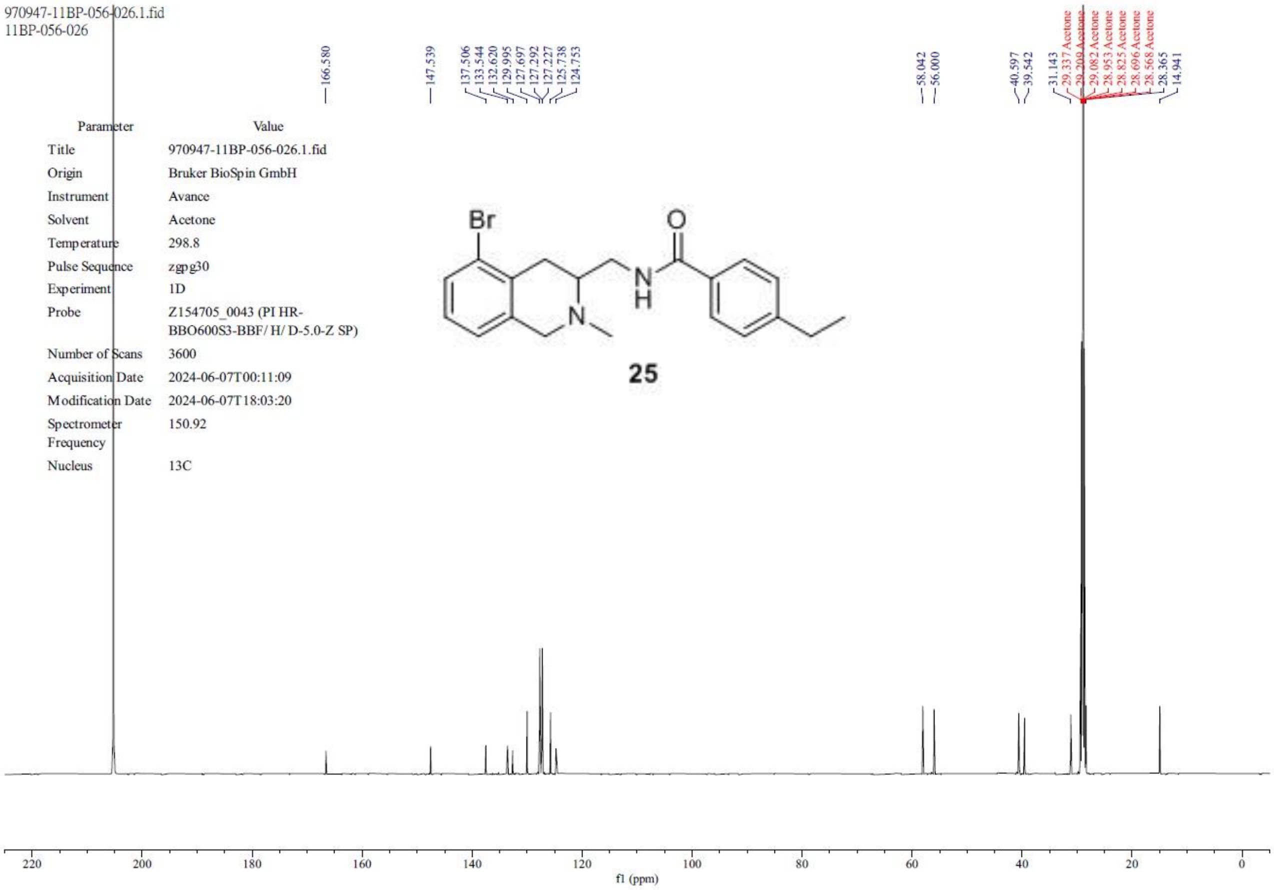

^{13}C NMR spectrum of compound **25**

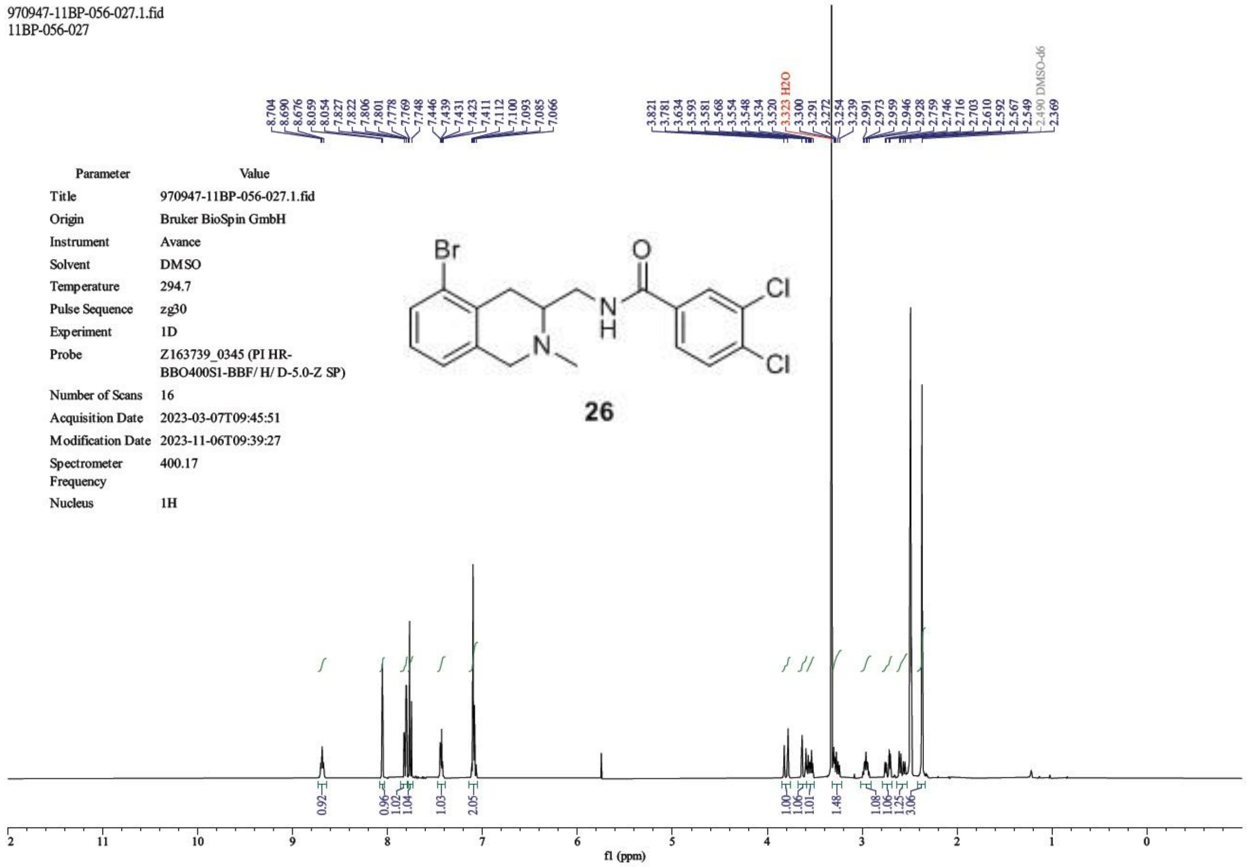

^{1}H NMR spectrum of compound **26**

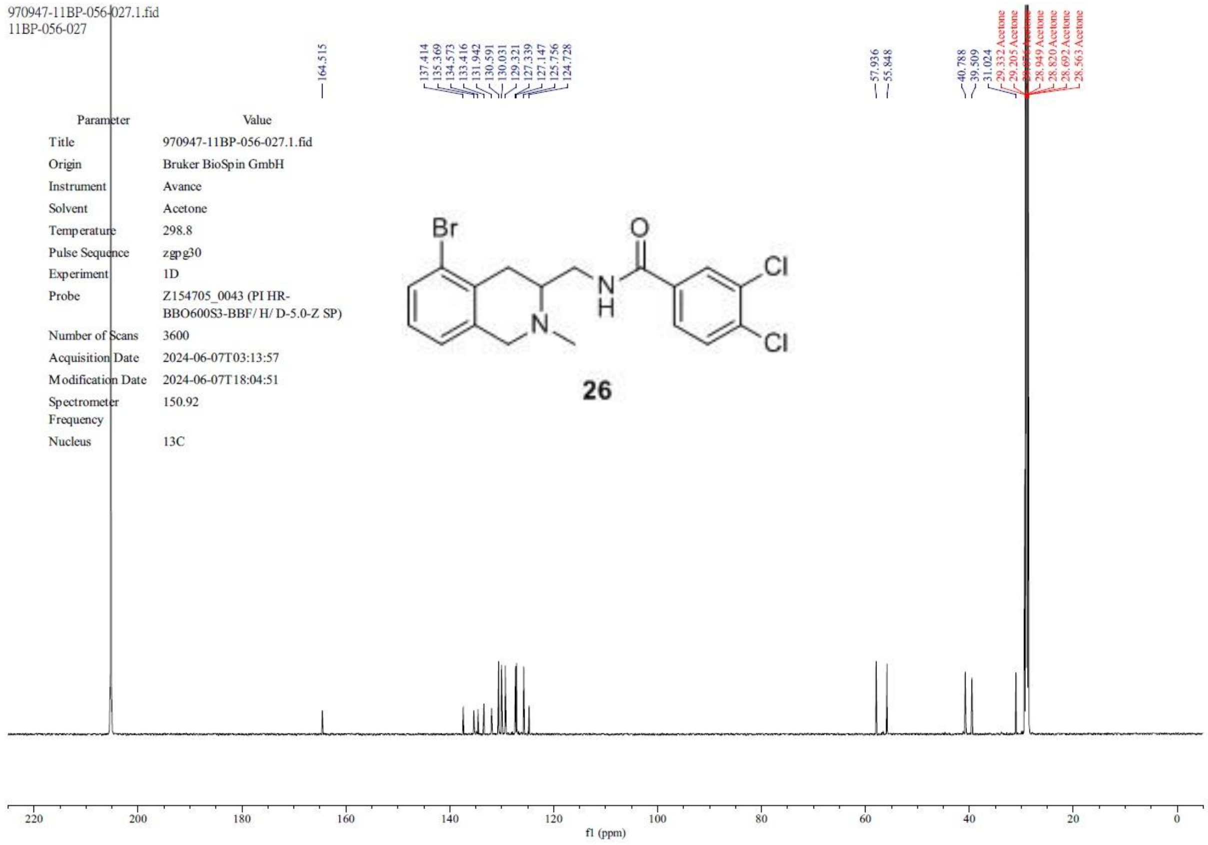

^{13}C NMR spectrum of compound **26**

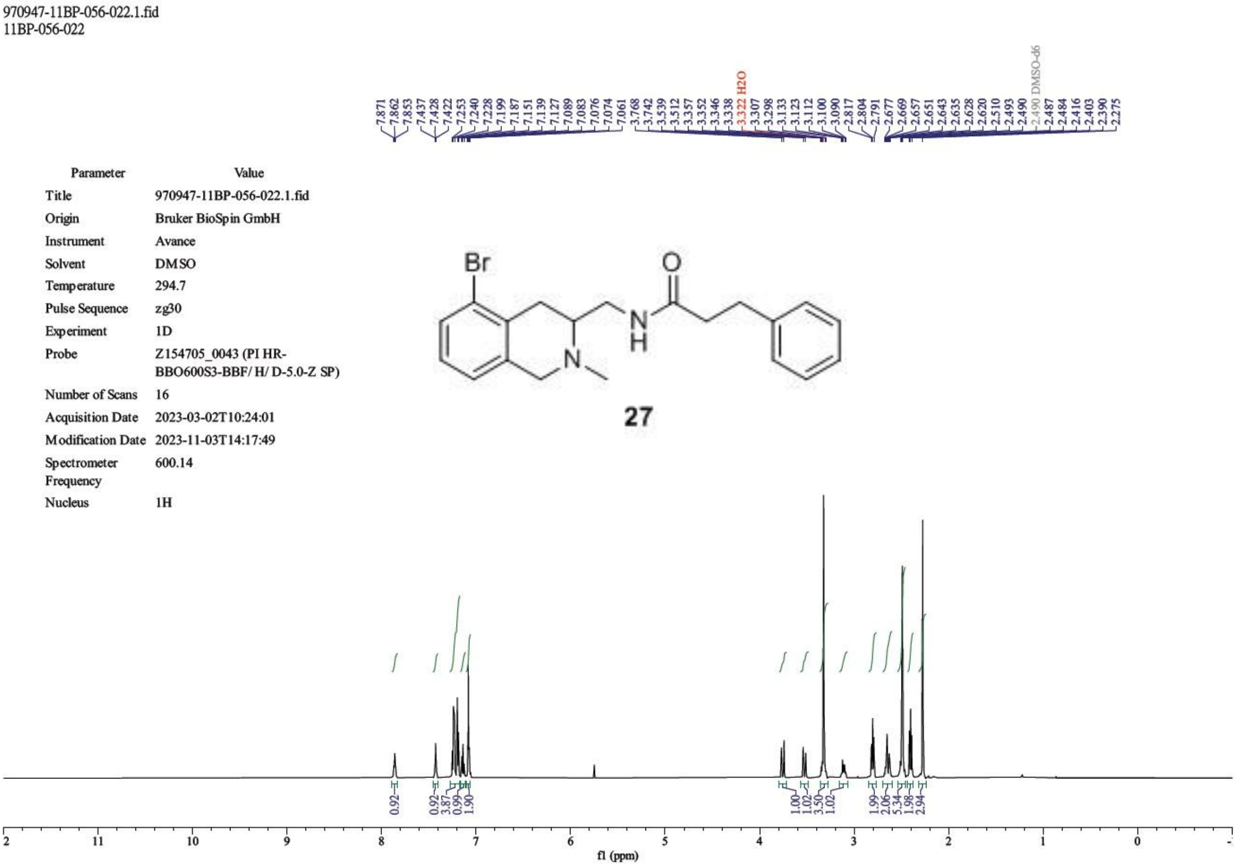

^{1}H NMR spectrum of compound **27**

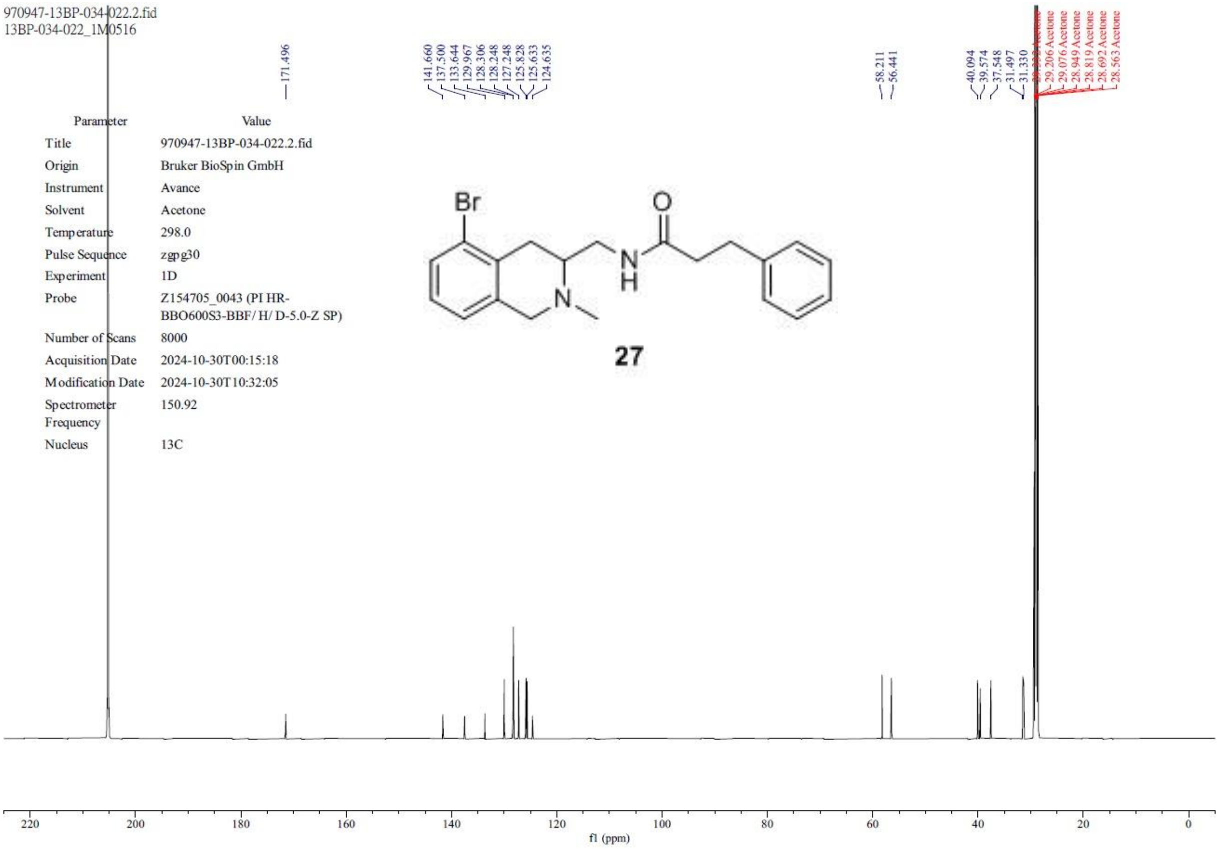

^{13}C NMR spectrum of compound **27**

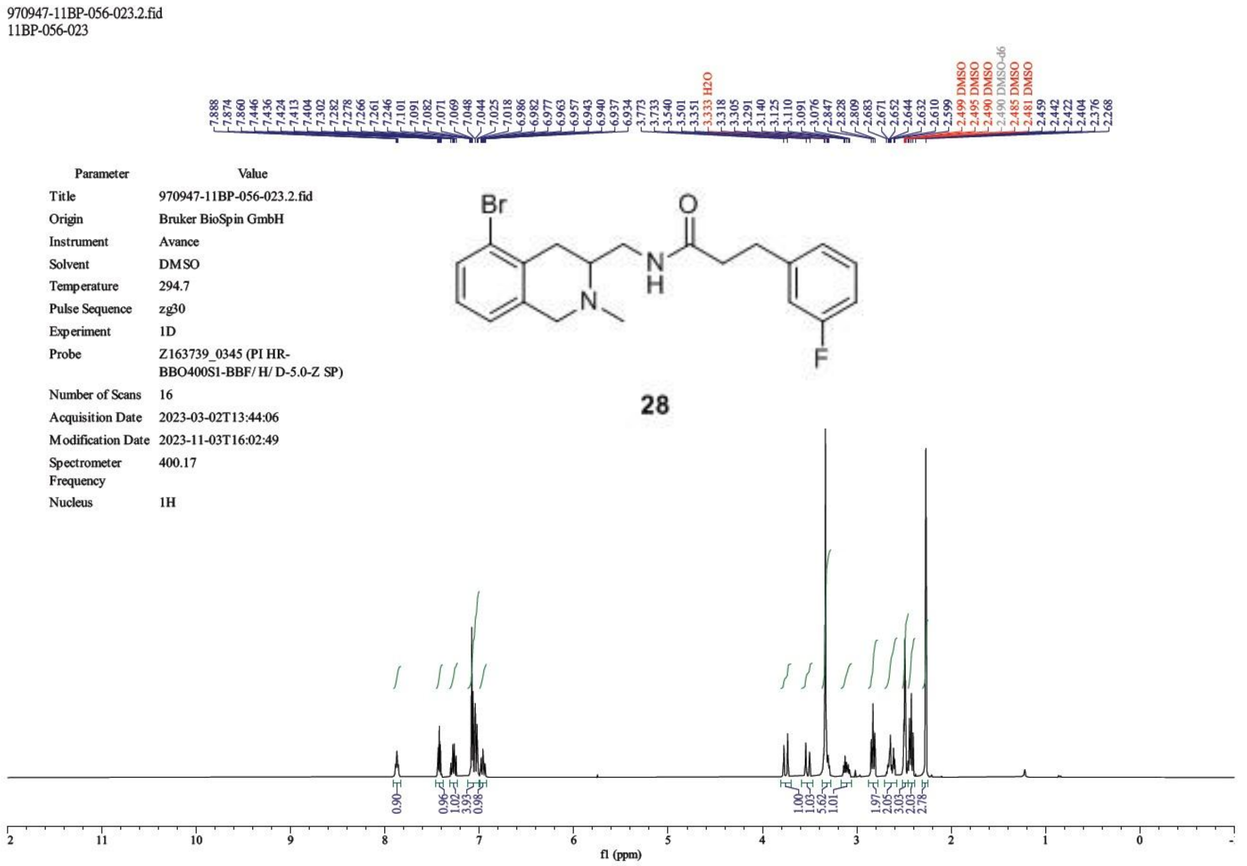

^{1}H NMR spectrum of compound **28**

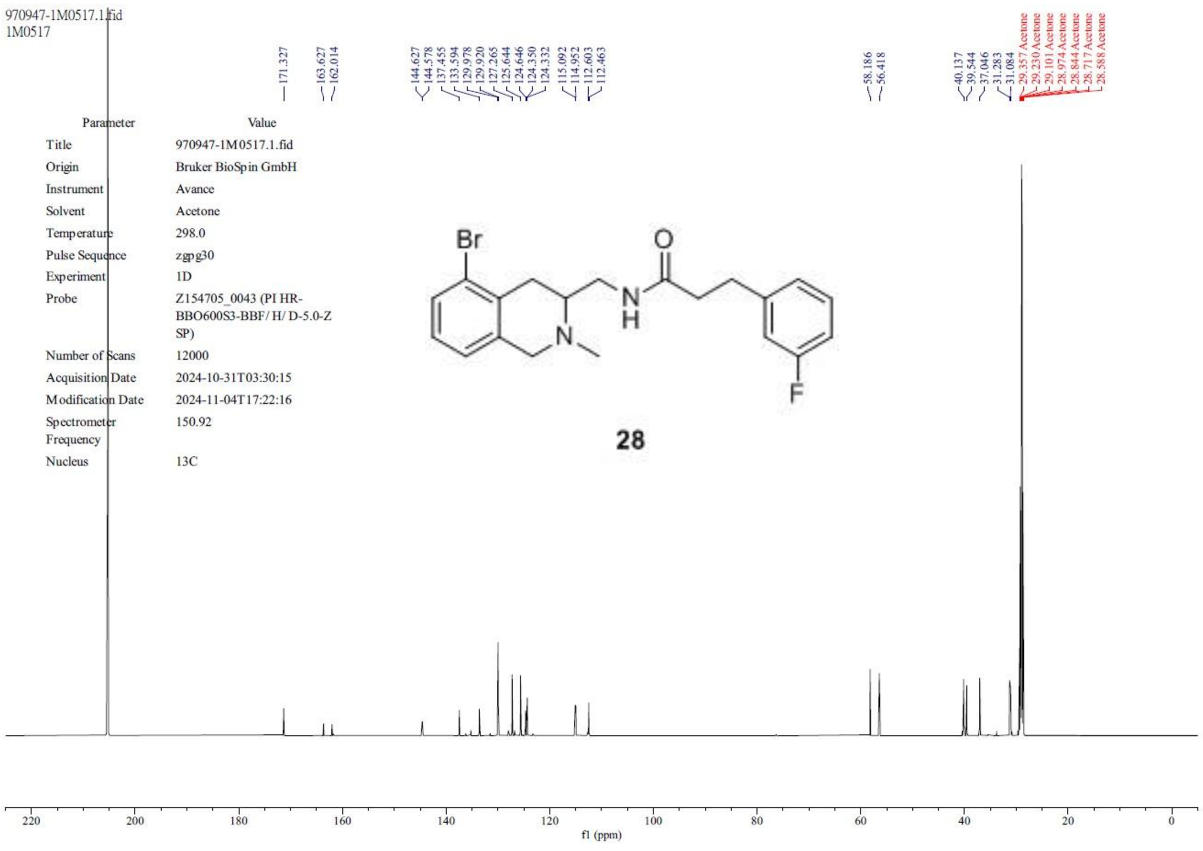

^{13}C NMR spectrum of compound **28**

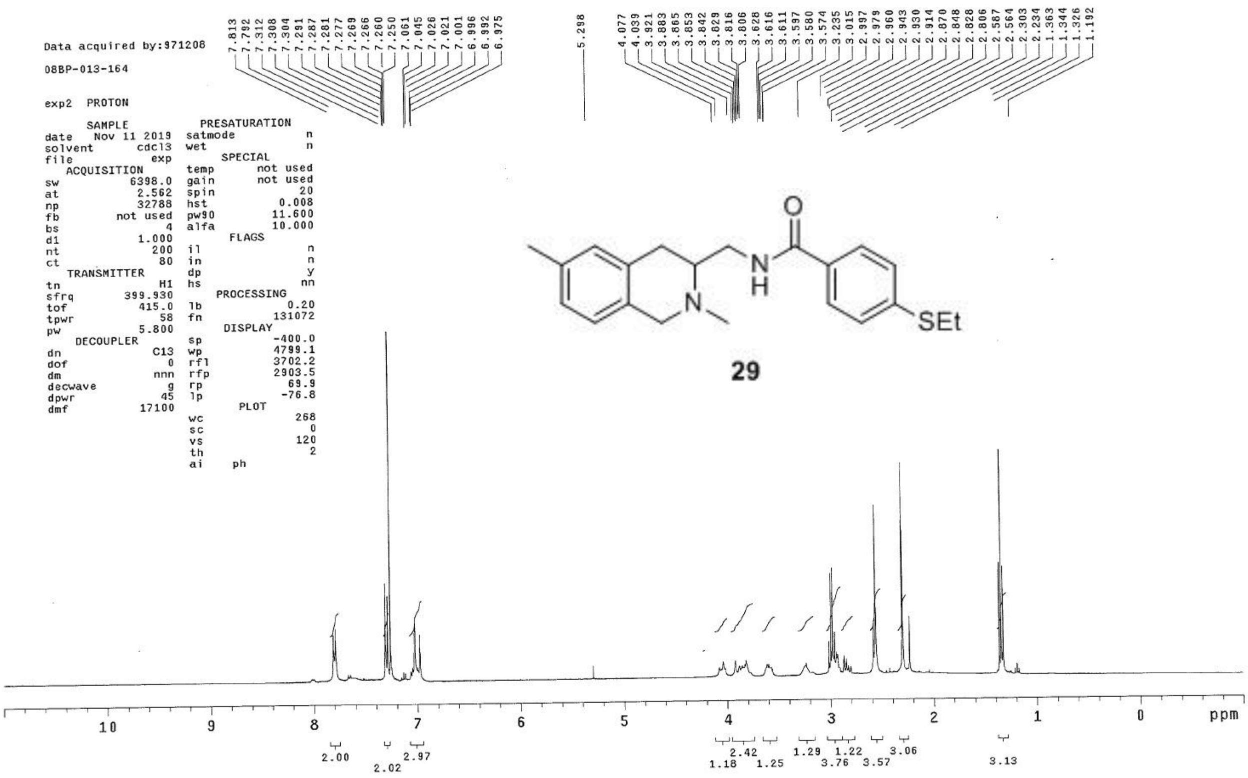

^1H NMR spectrum of compound **29**

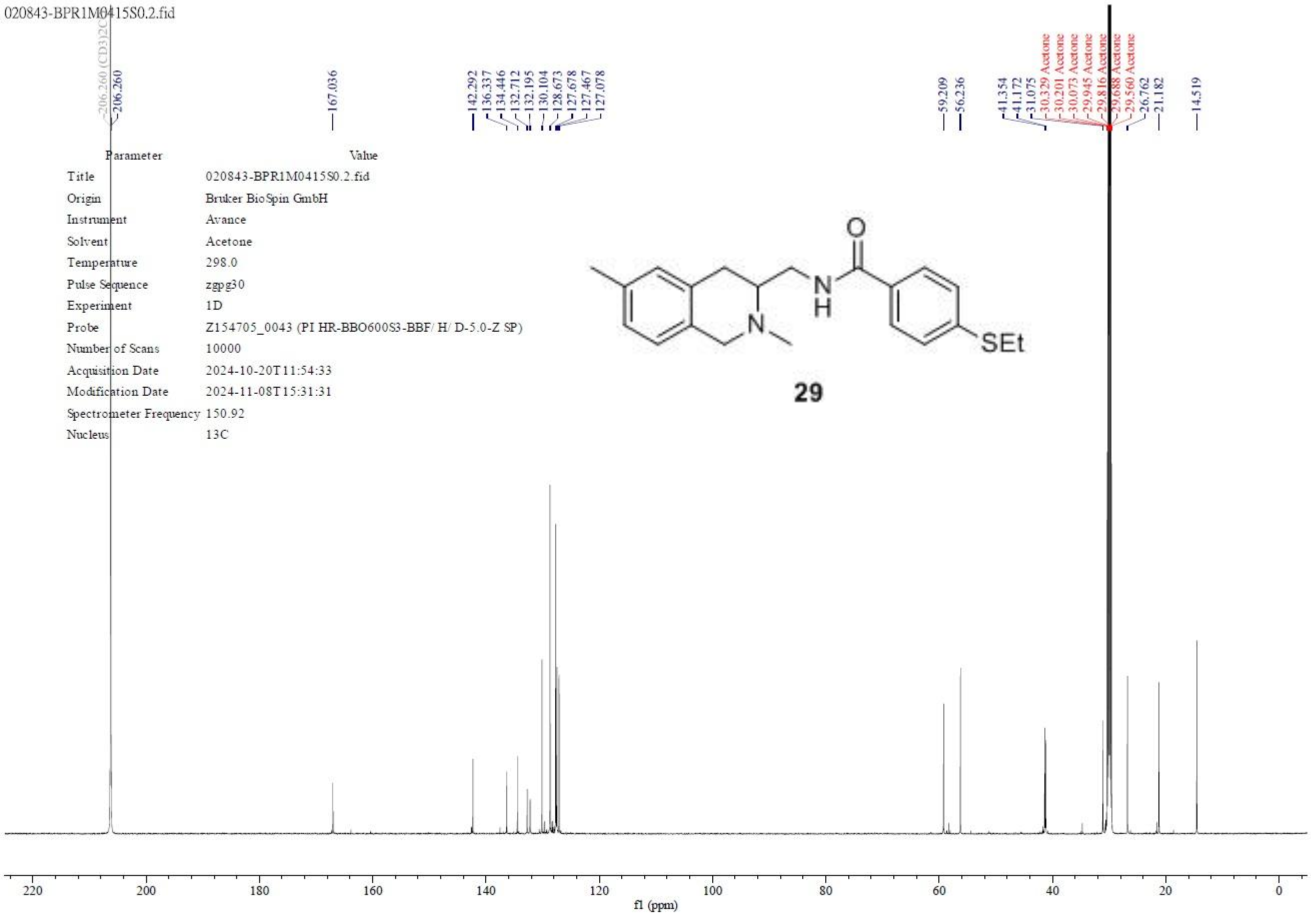

^{13}C NMR spectrum of compound **29**

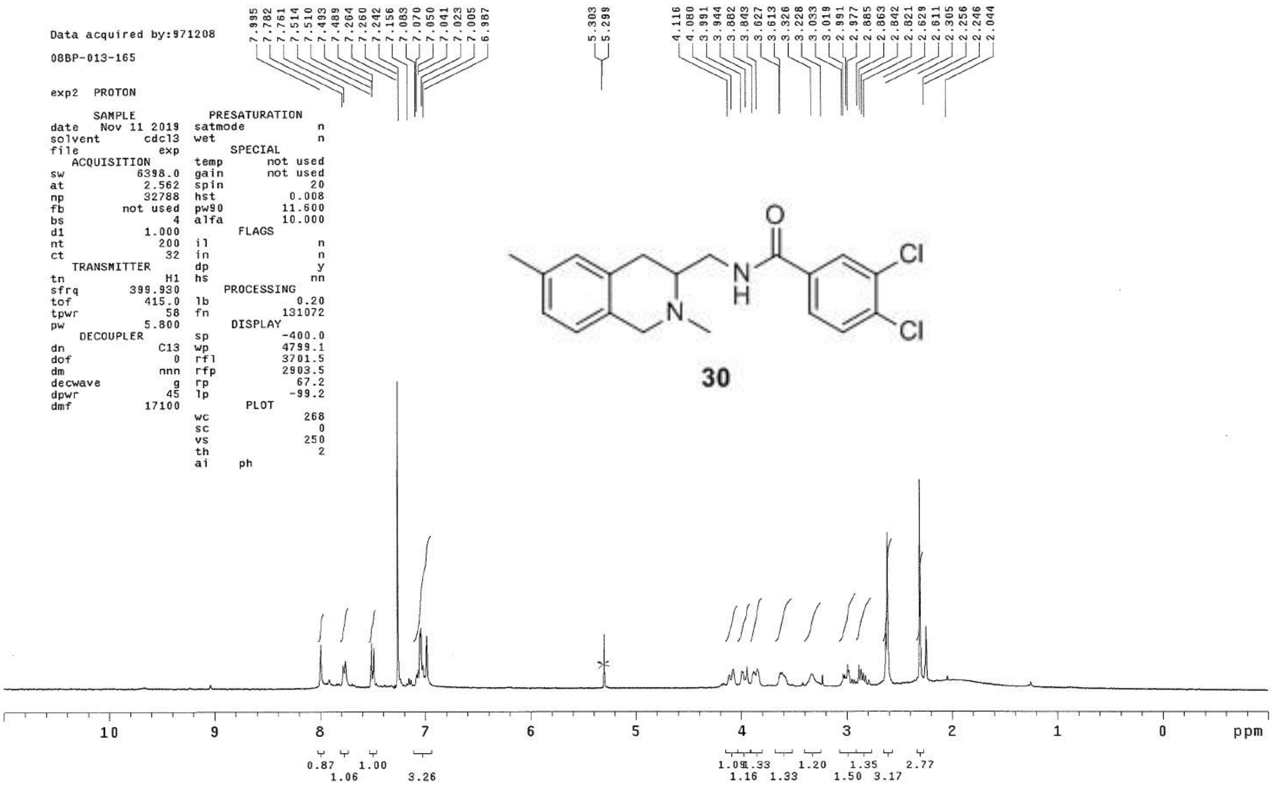

^{1}H NMR spectrum of compound **30**

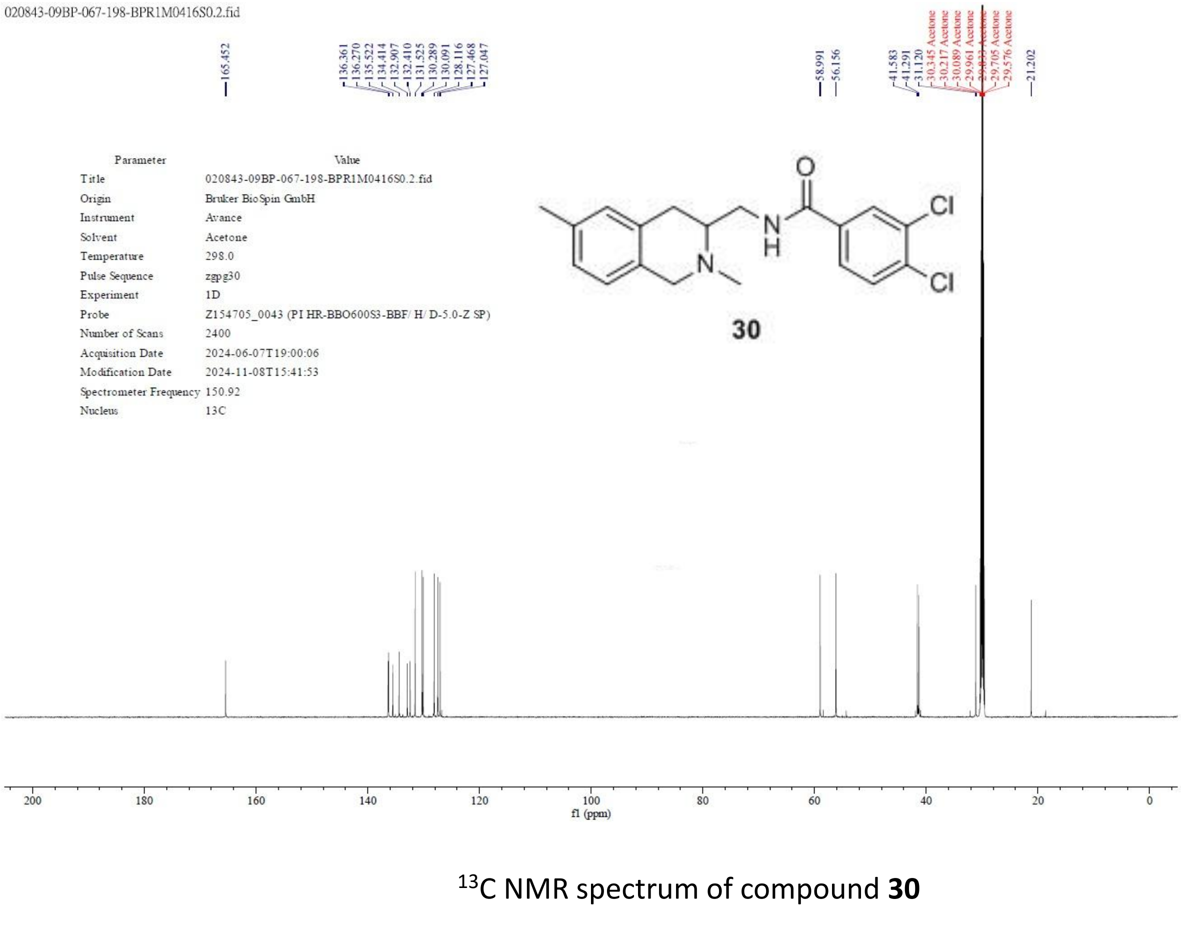

^{13}C NMR spectrum of compound **30**

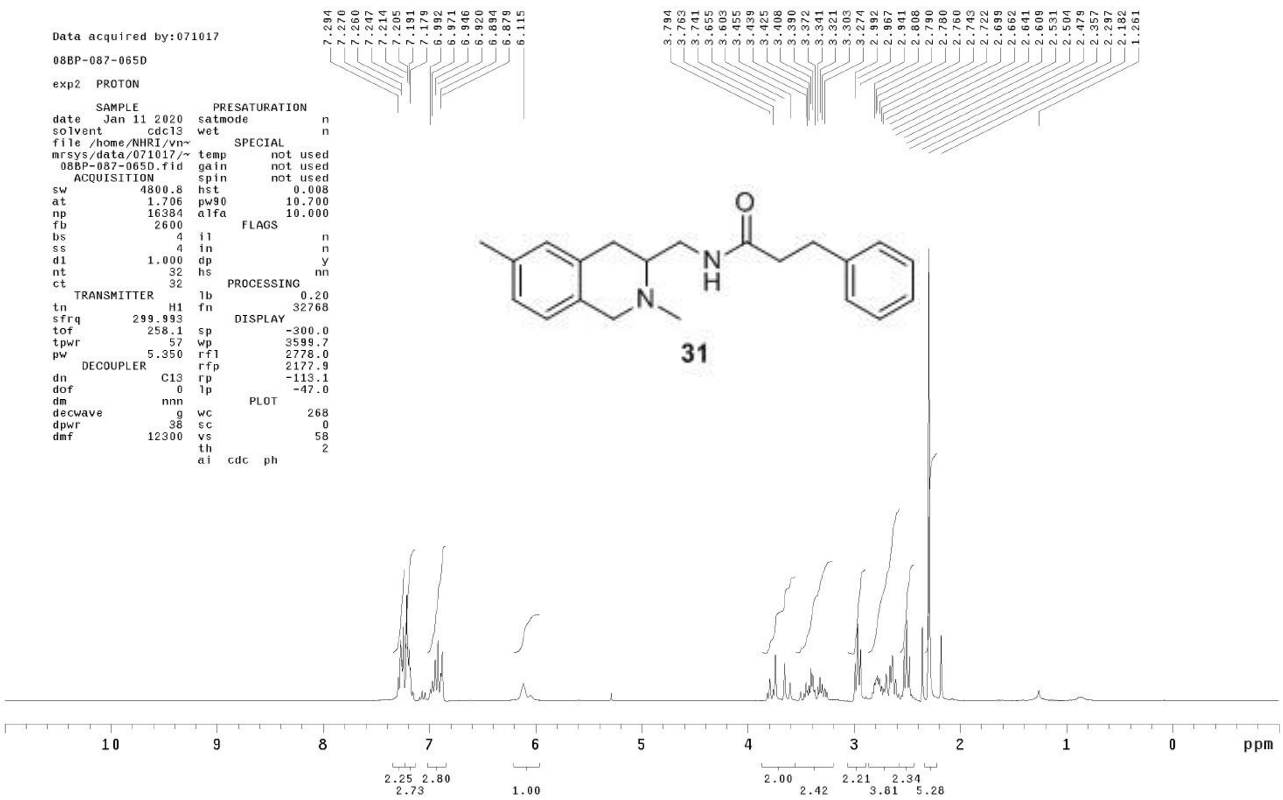

^1H NMR spectrum of compound **31**

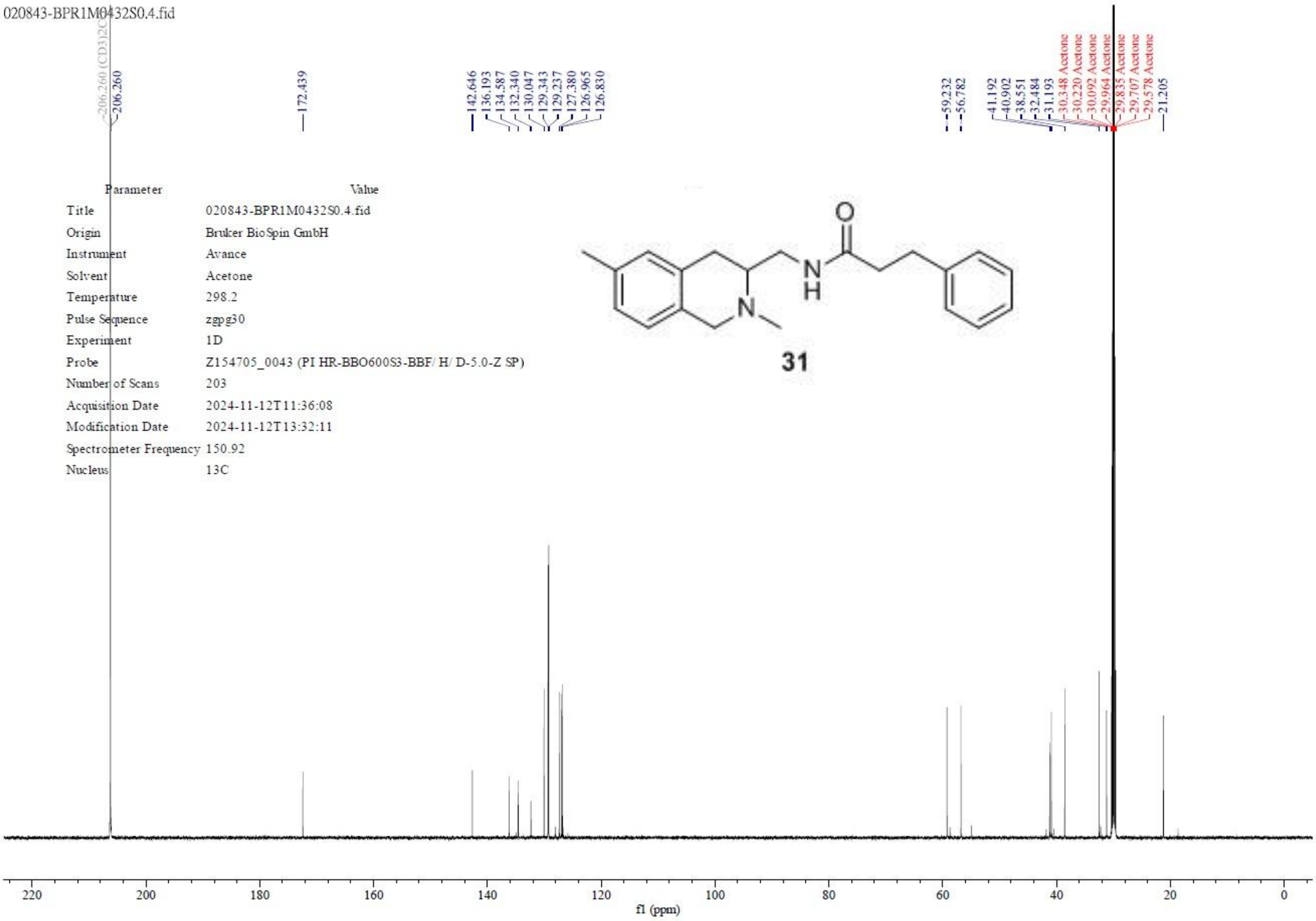

131

^{13}C NMR spectrum of compound **31**

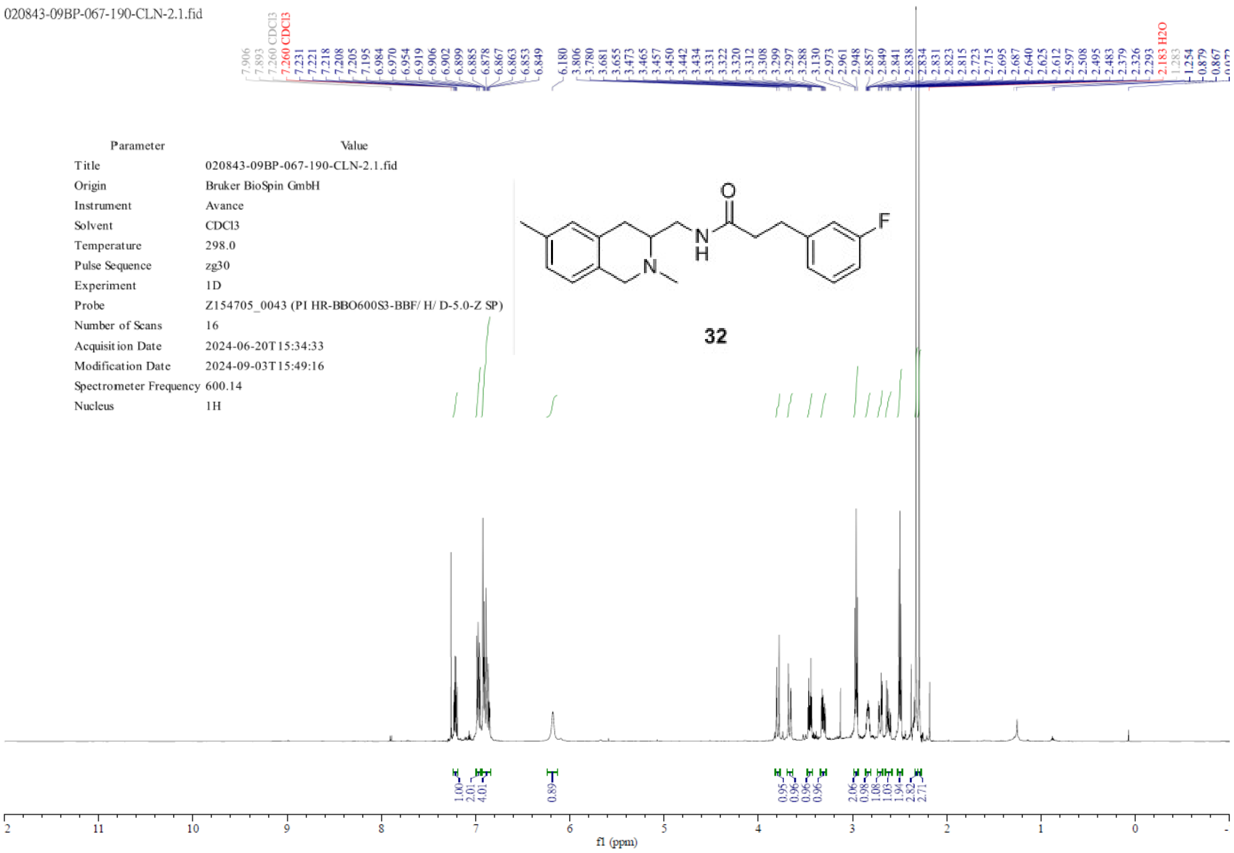

^{1}H NMR spectrum of compound **32**

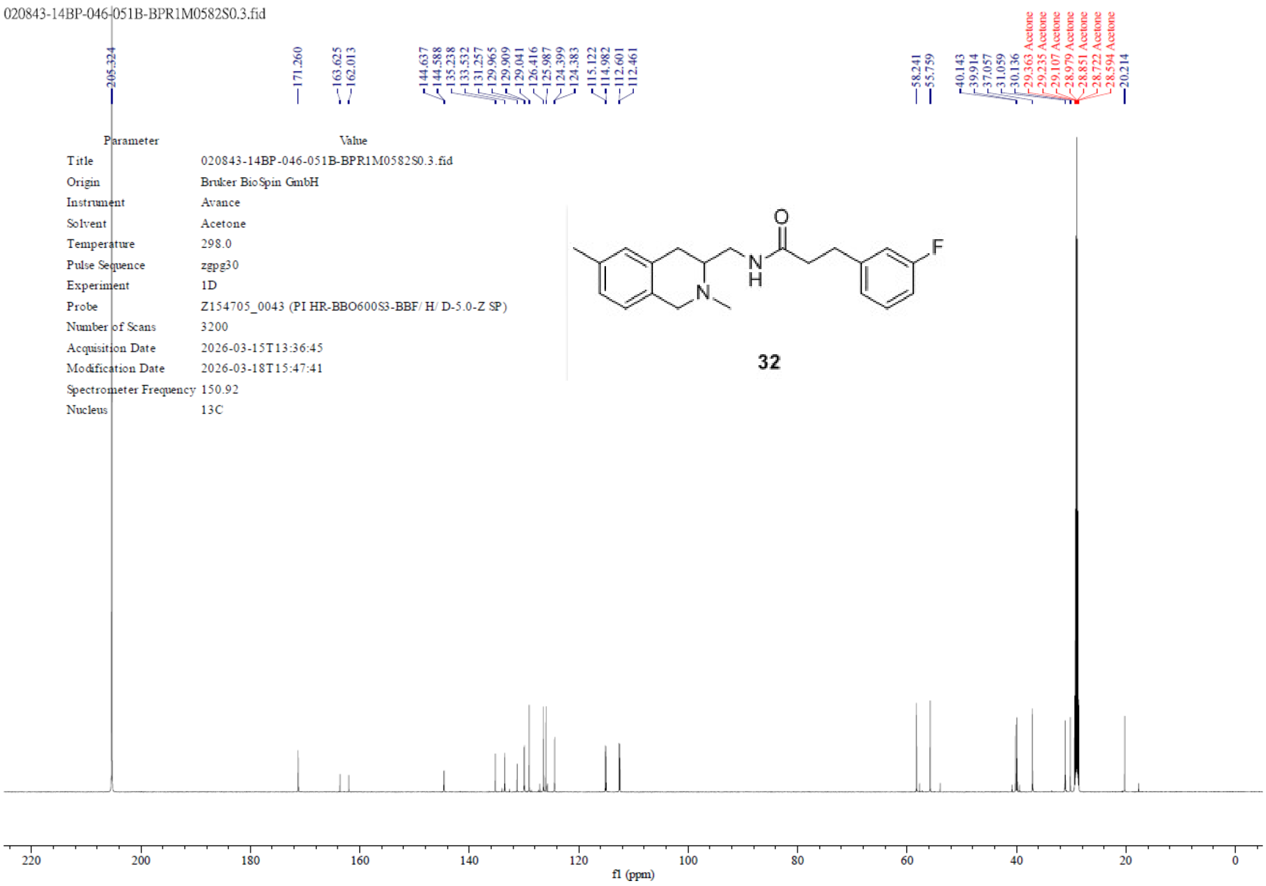

^{13}C NMR spectrum of compound **32**

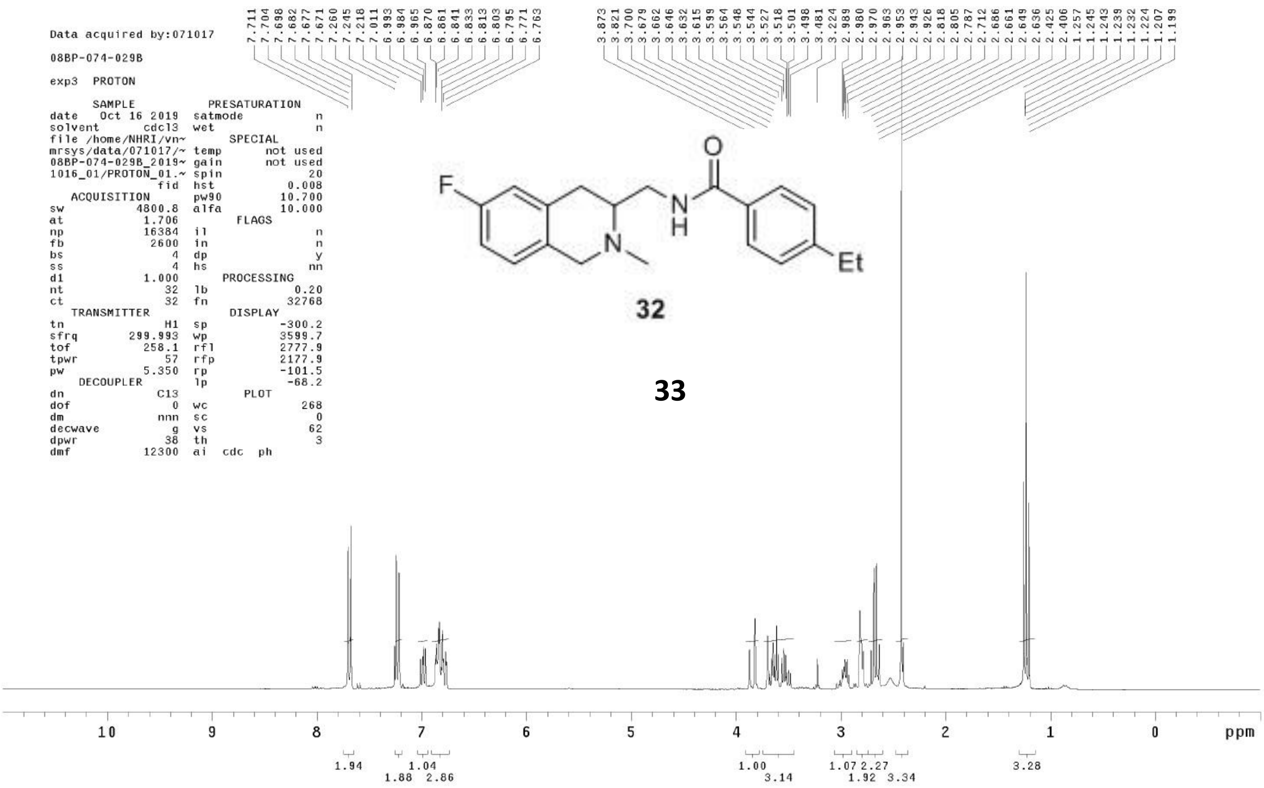

^{1}H NMR spectrum of compound **33**

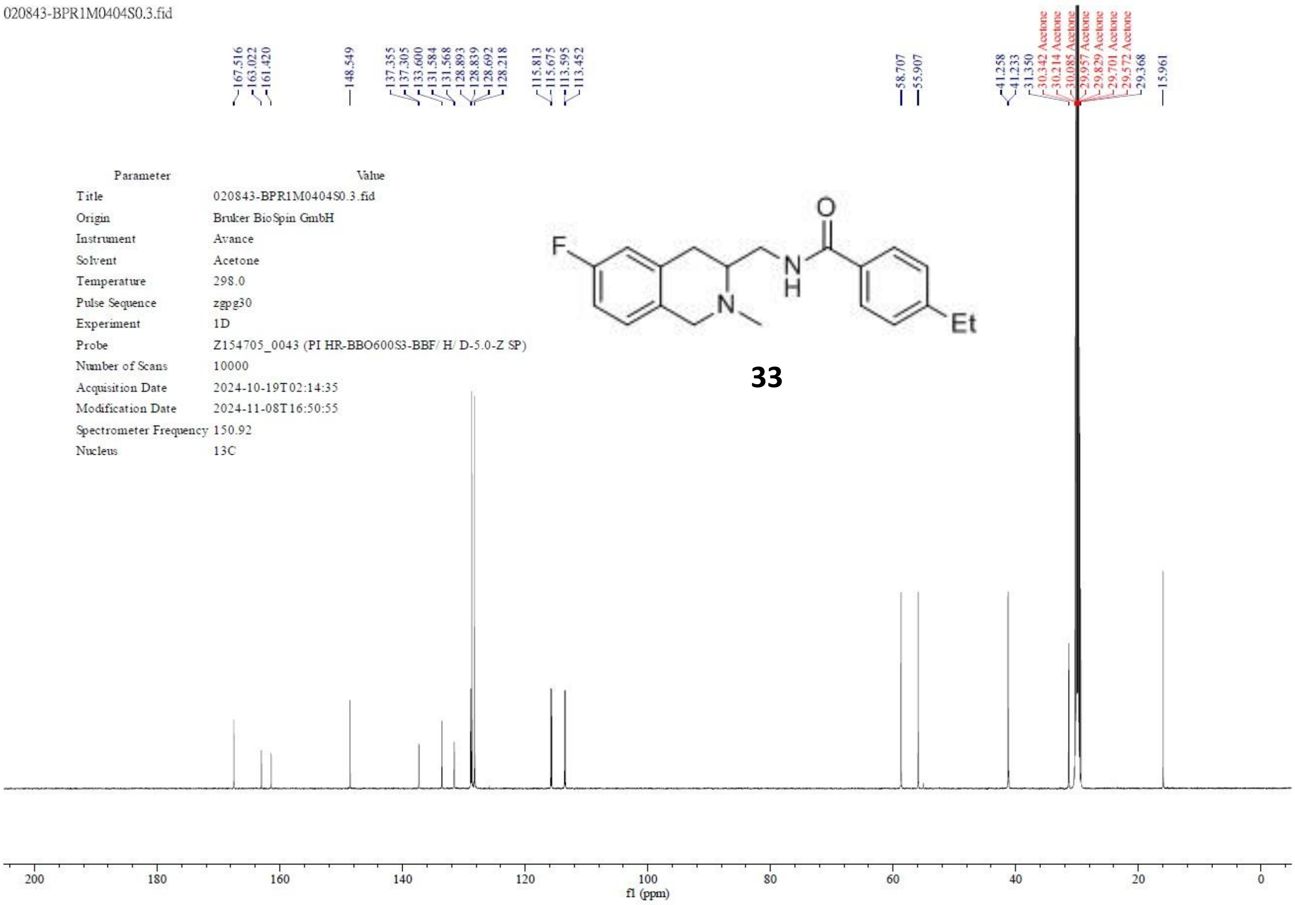

^{13}C NMR spectrum of compound **33**

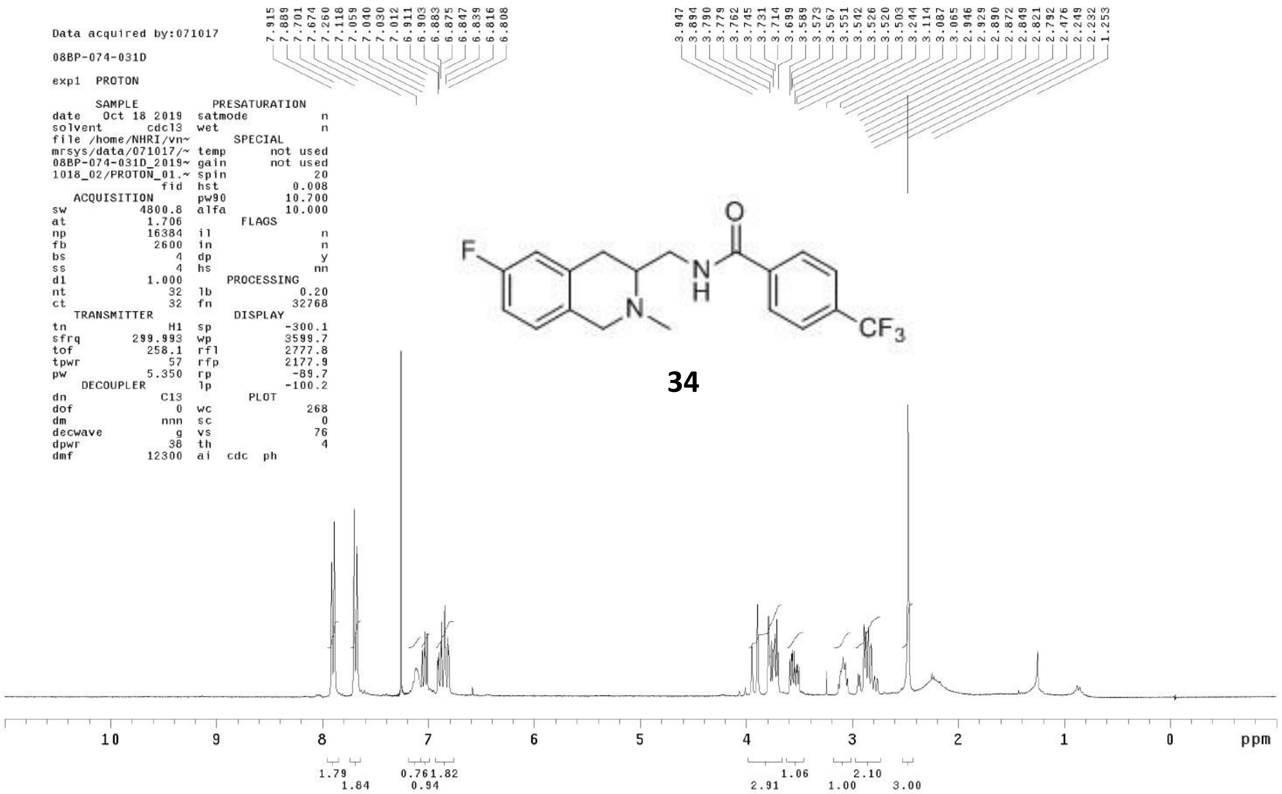

^{1}H NMR spectrum of compound **34**

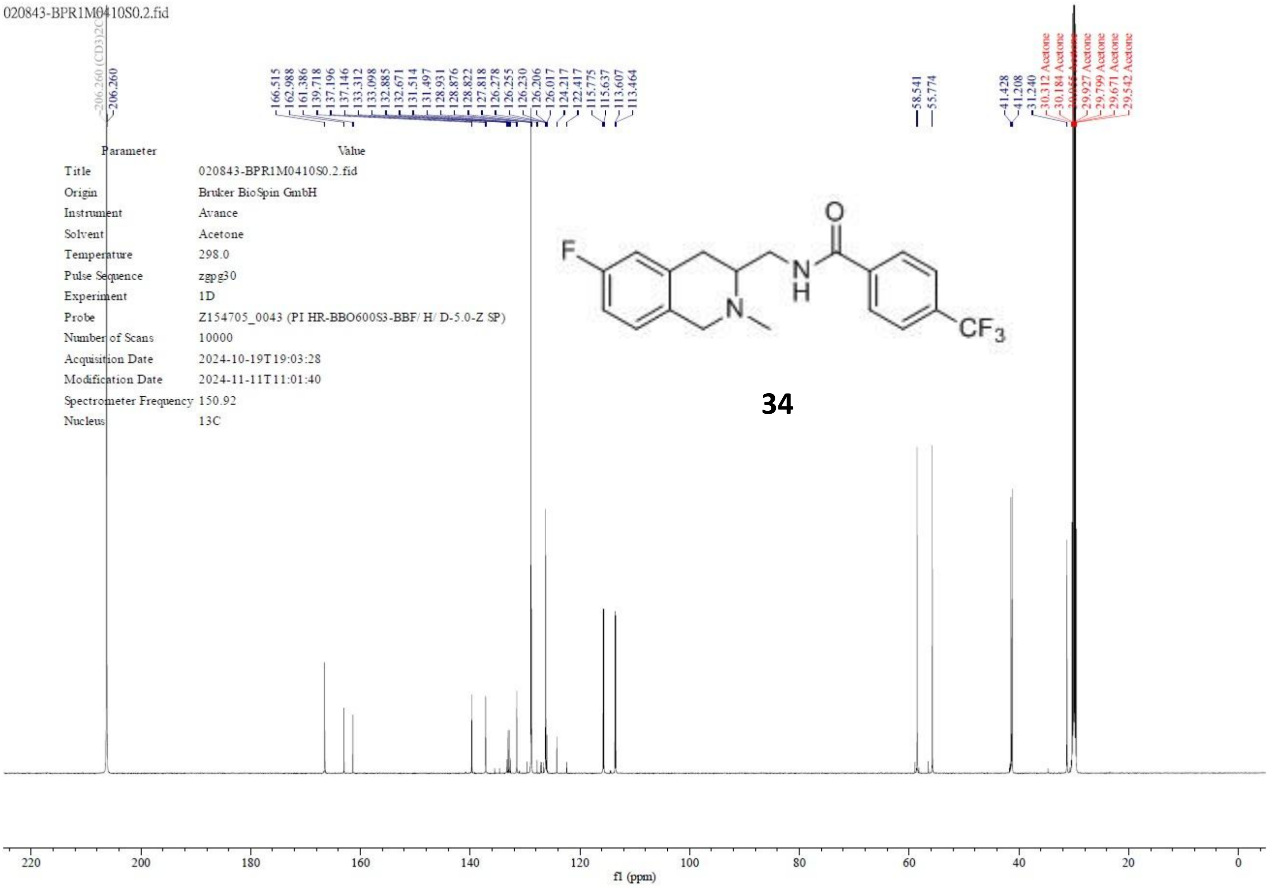

^{13}C NMR spectrum of compound **34**

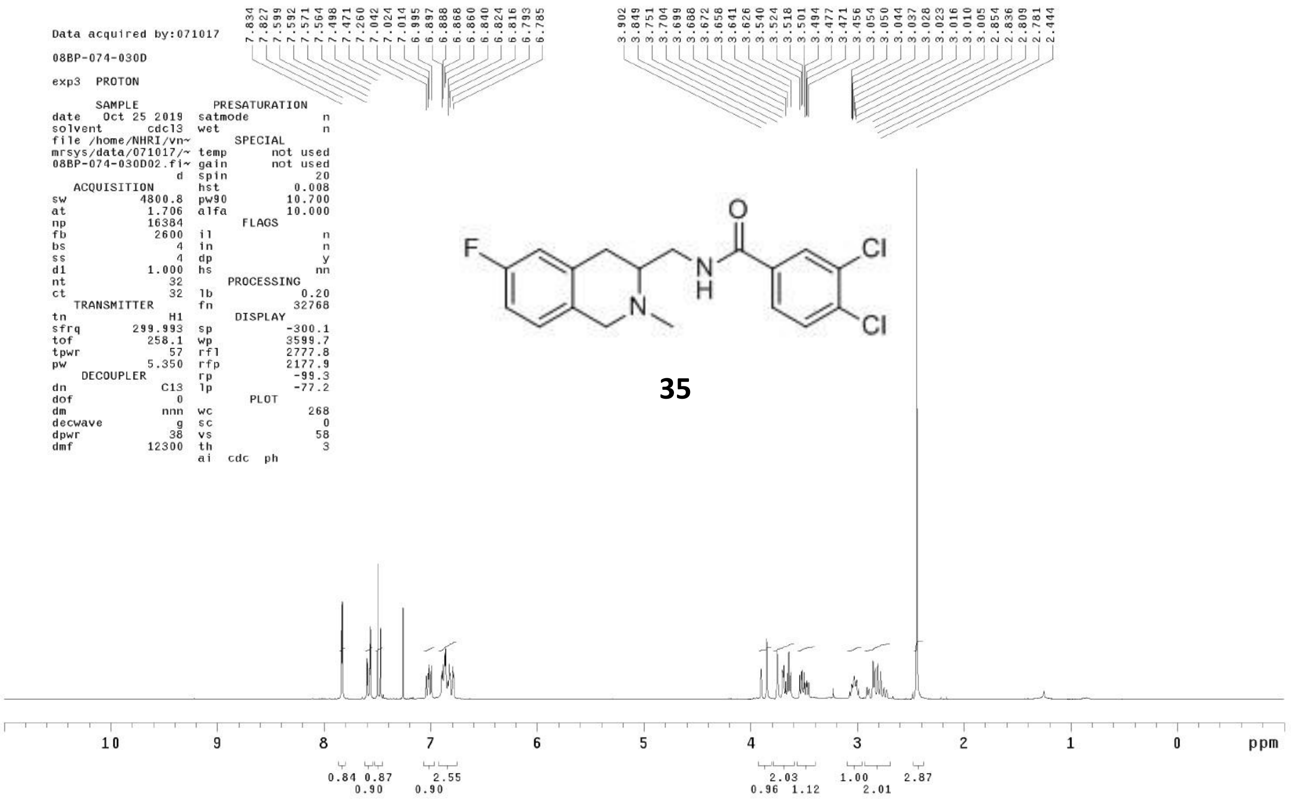

^{1}H NMR spectrum of compound **35**

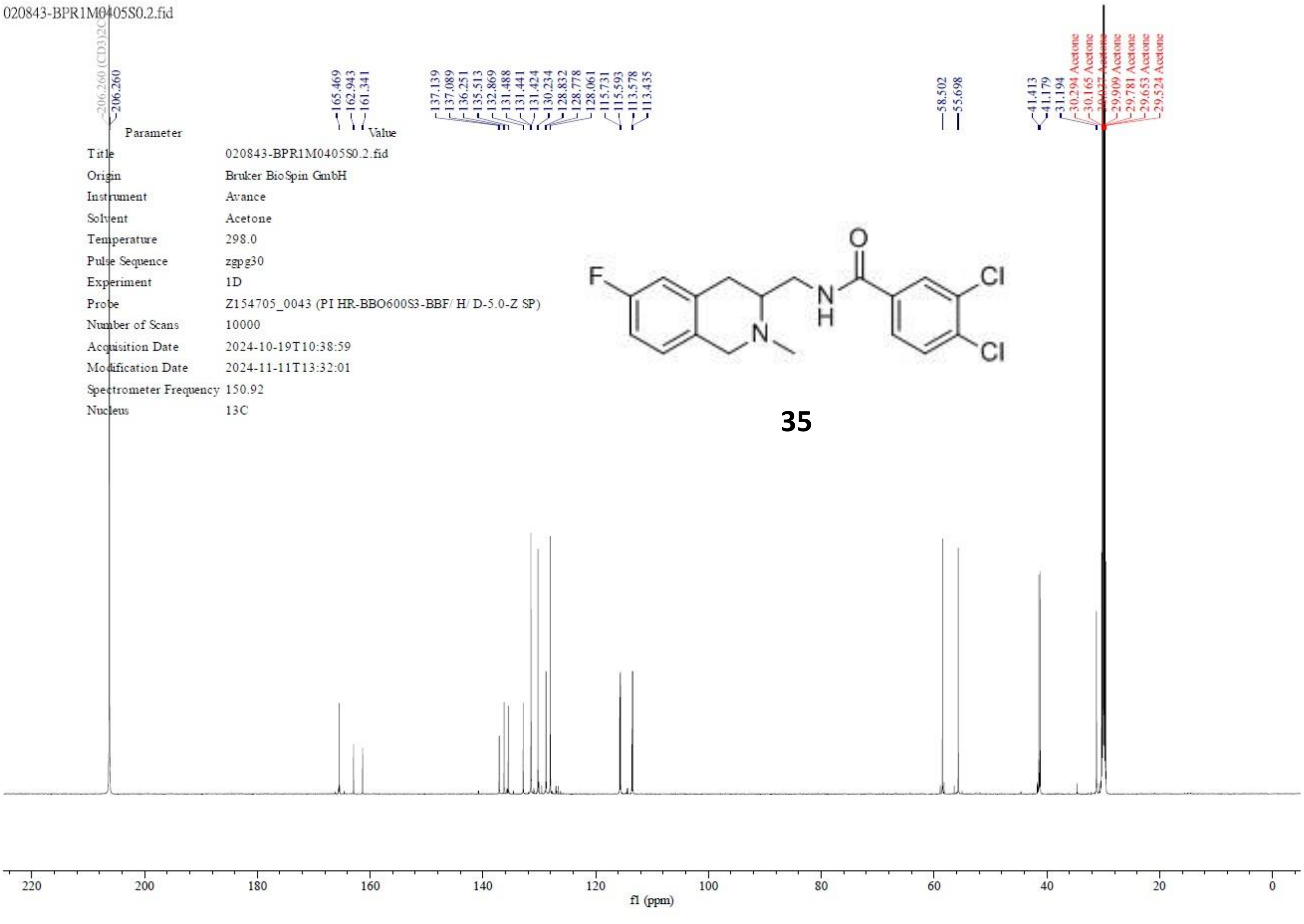

^{13}C NMR spectrum of compound **35**

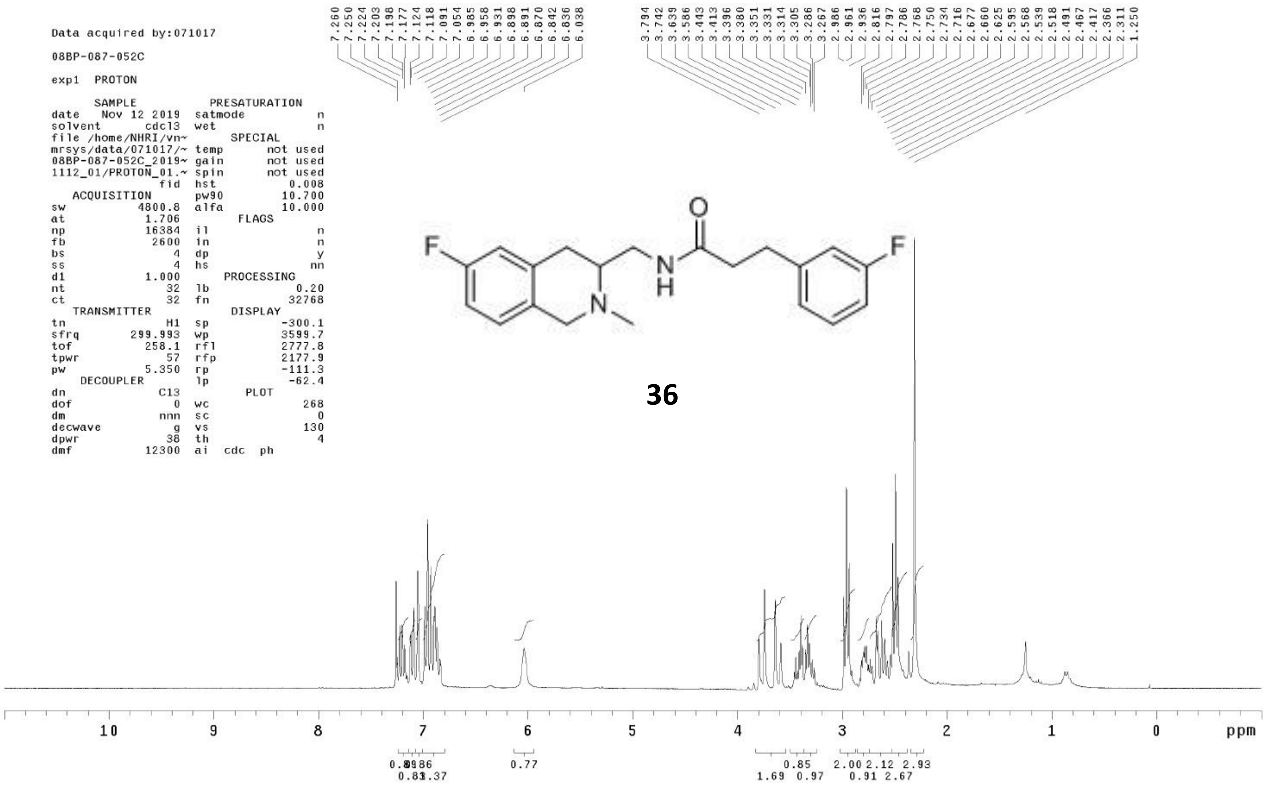

¹H NMR spectrum of compound **36**

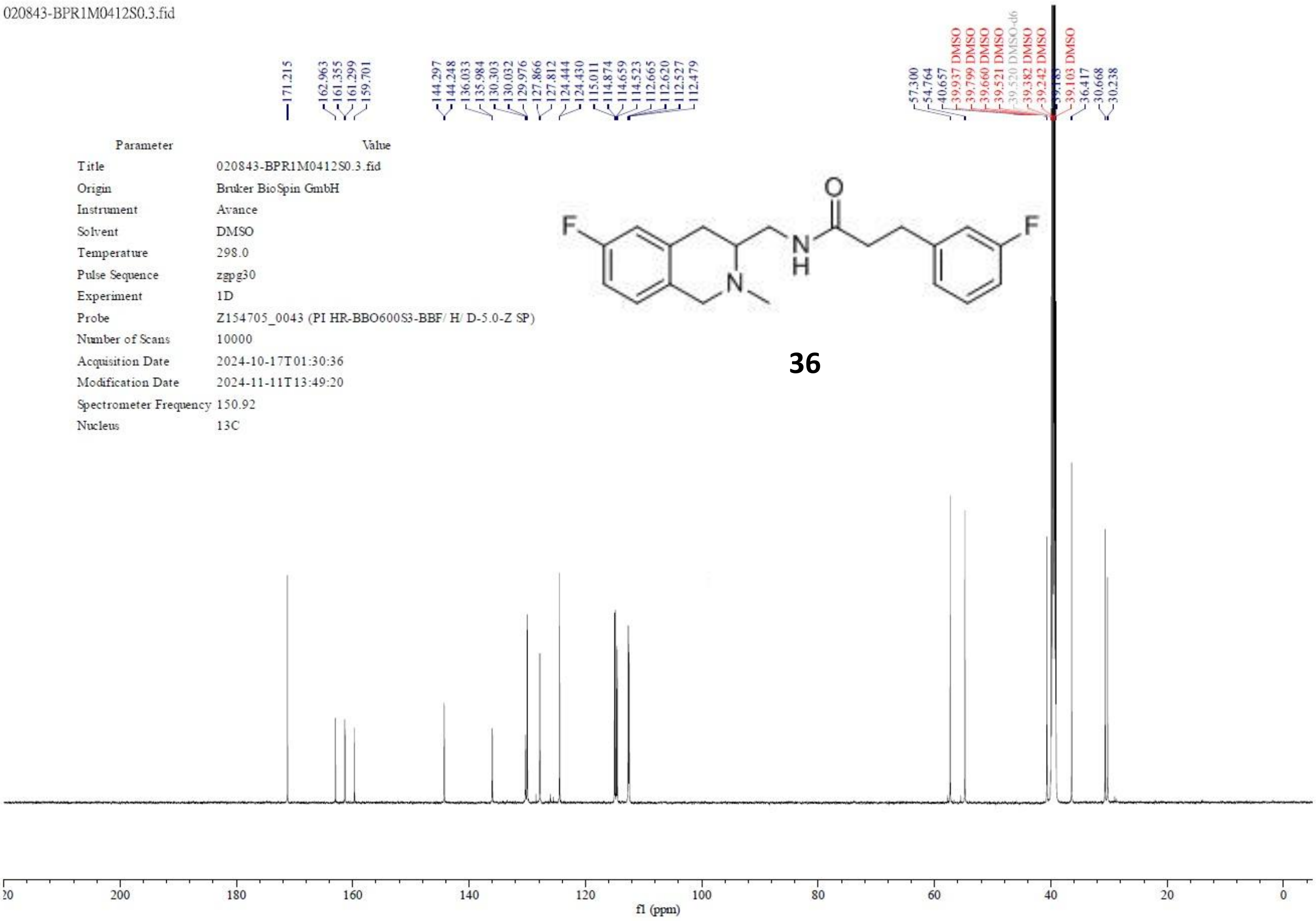

¹³C NMR spectrum of compound **36**

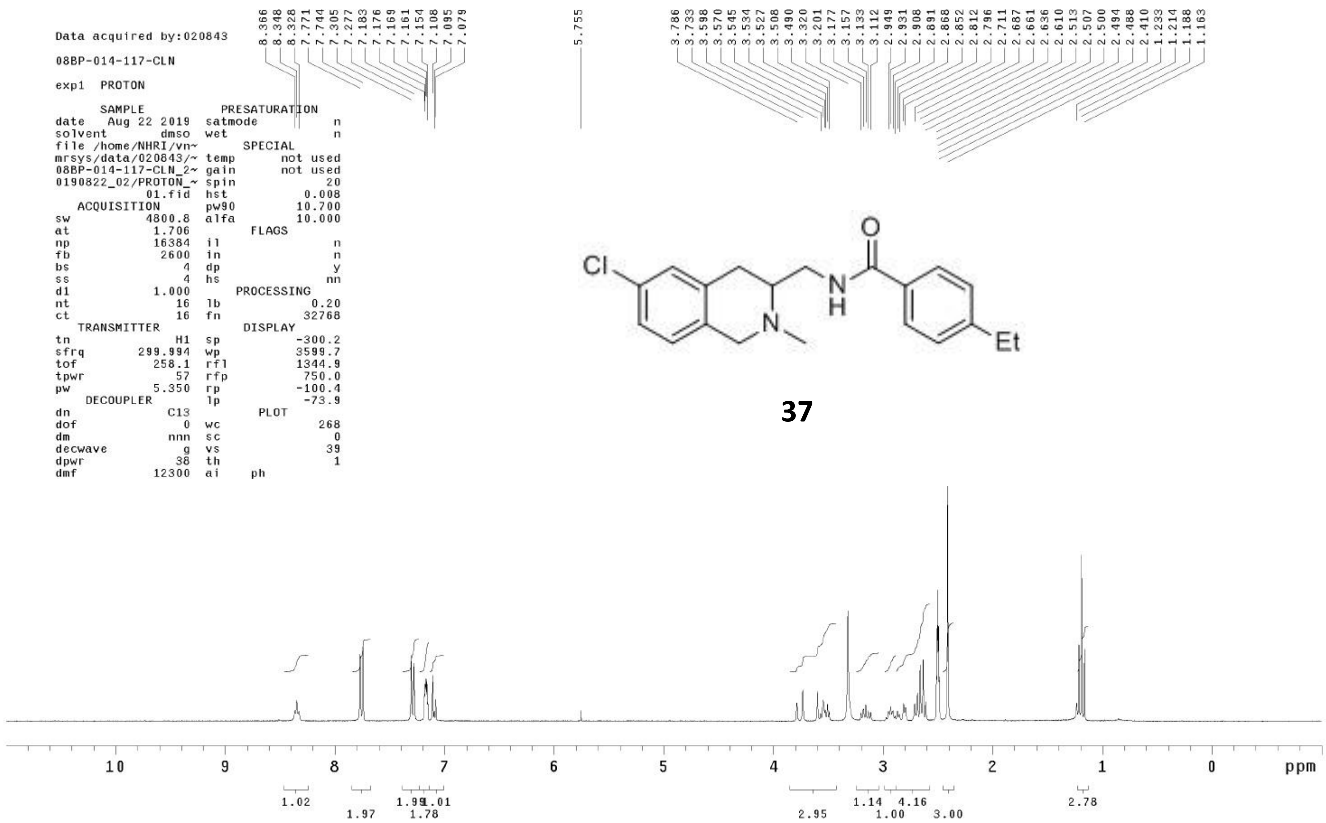

^{1}H

^{1}H NMR spectrum of compound **37**

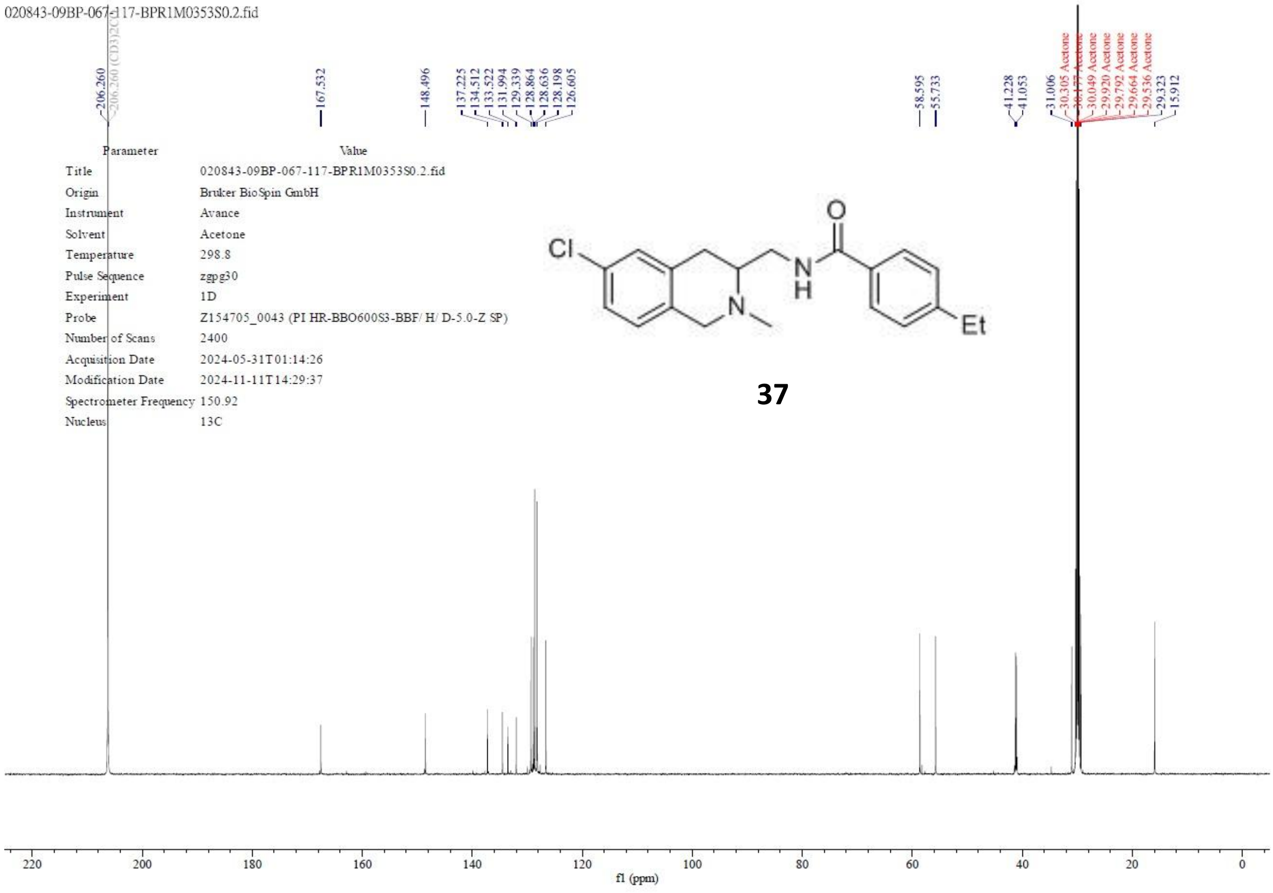

^{13}C NMR spectrum of compound **37**

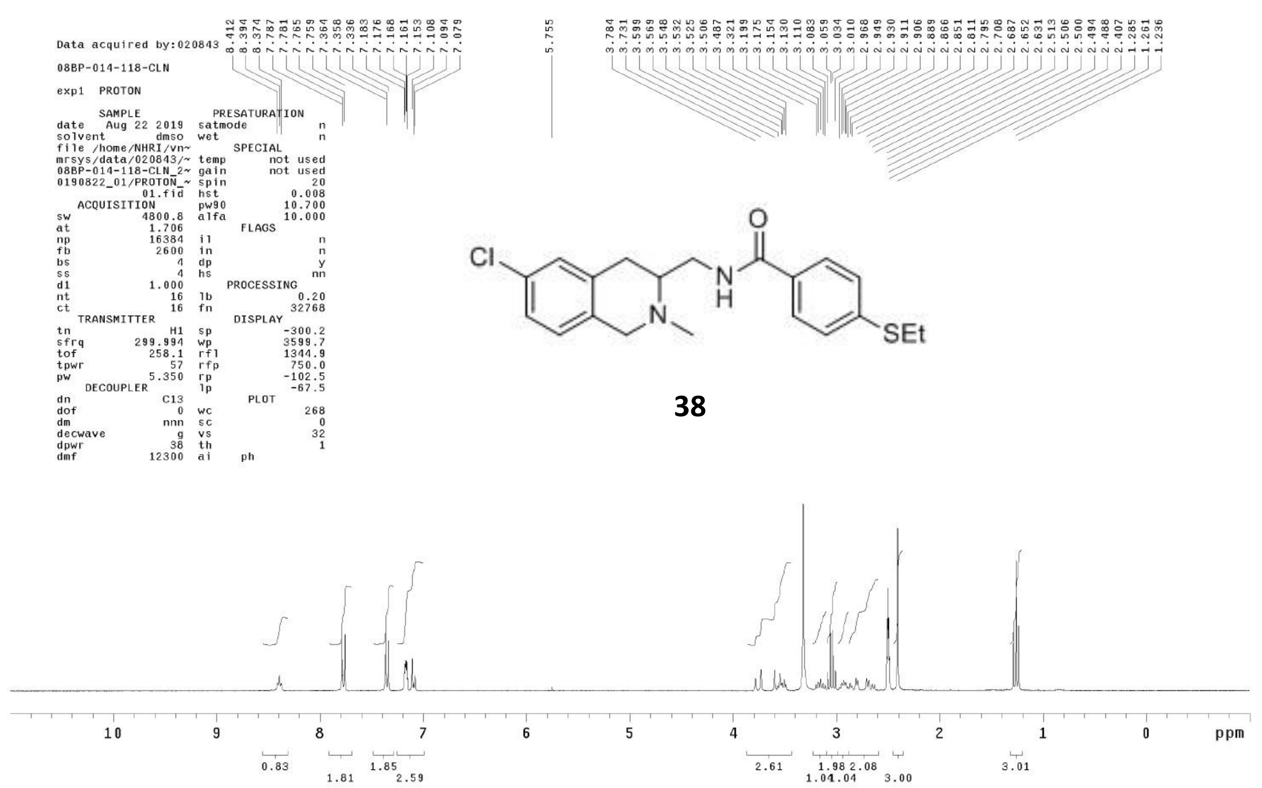

^{1}H NMR spectrum of compound **38**

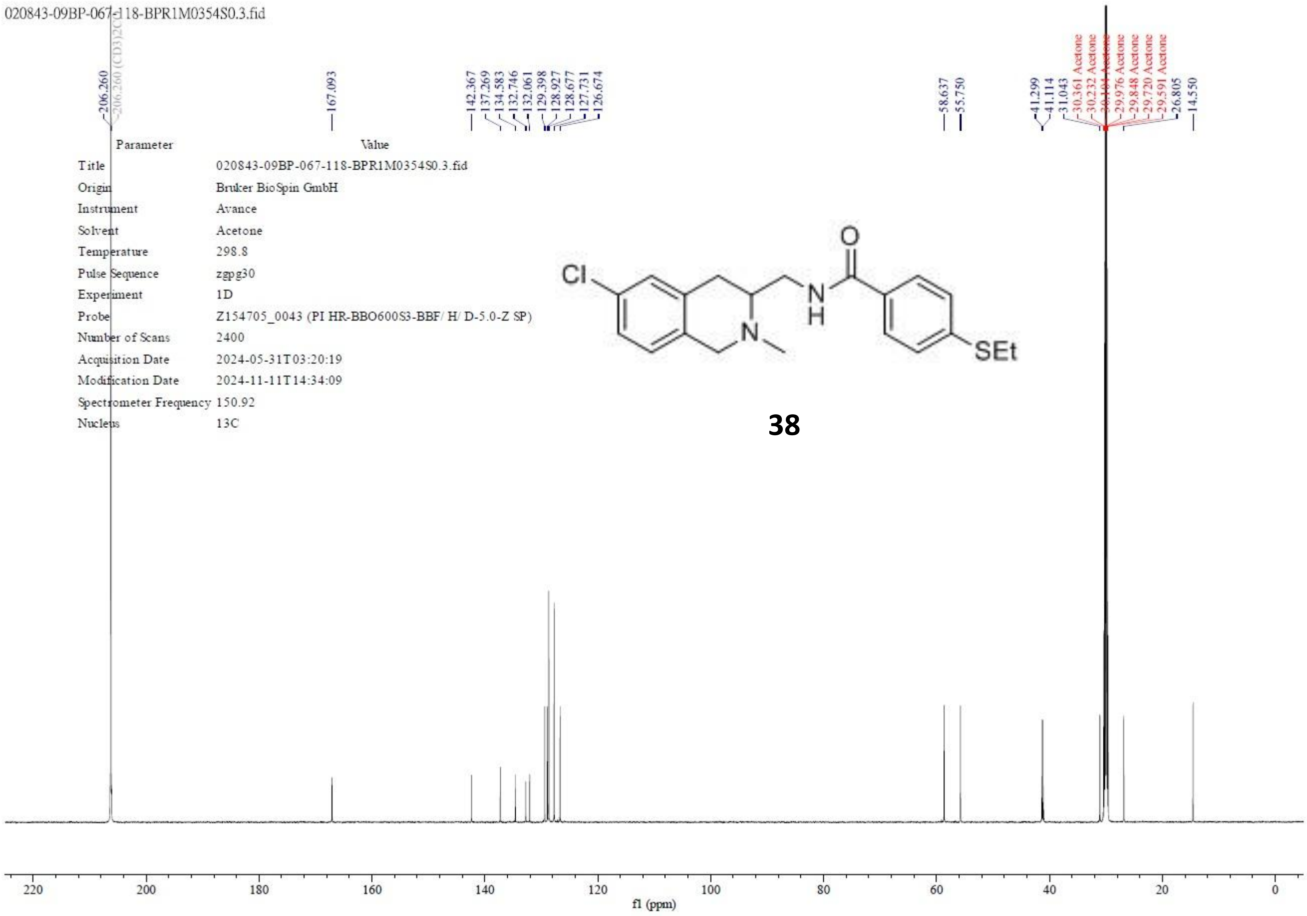

^{13}C NMR spectrum of compound **38**

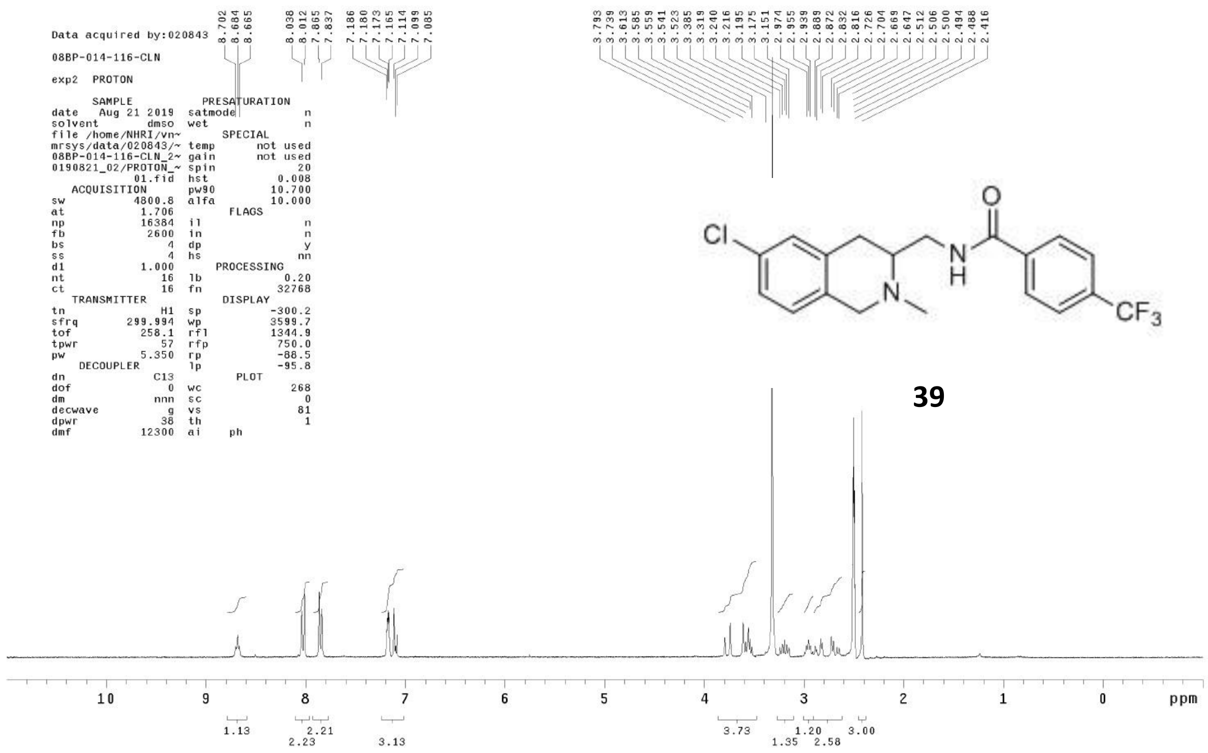

^{1}H NMR spectrum of compound **39**

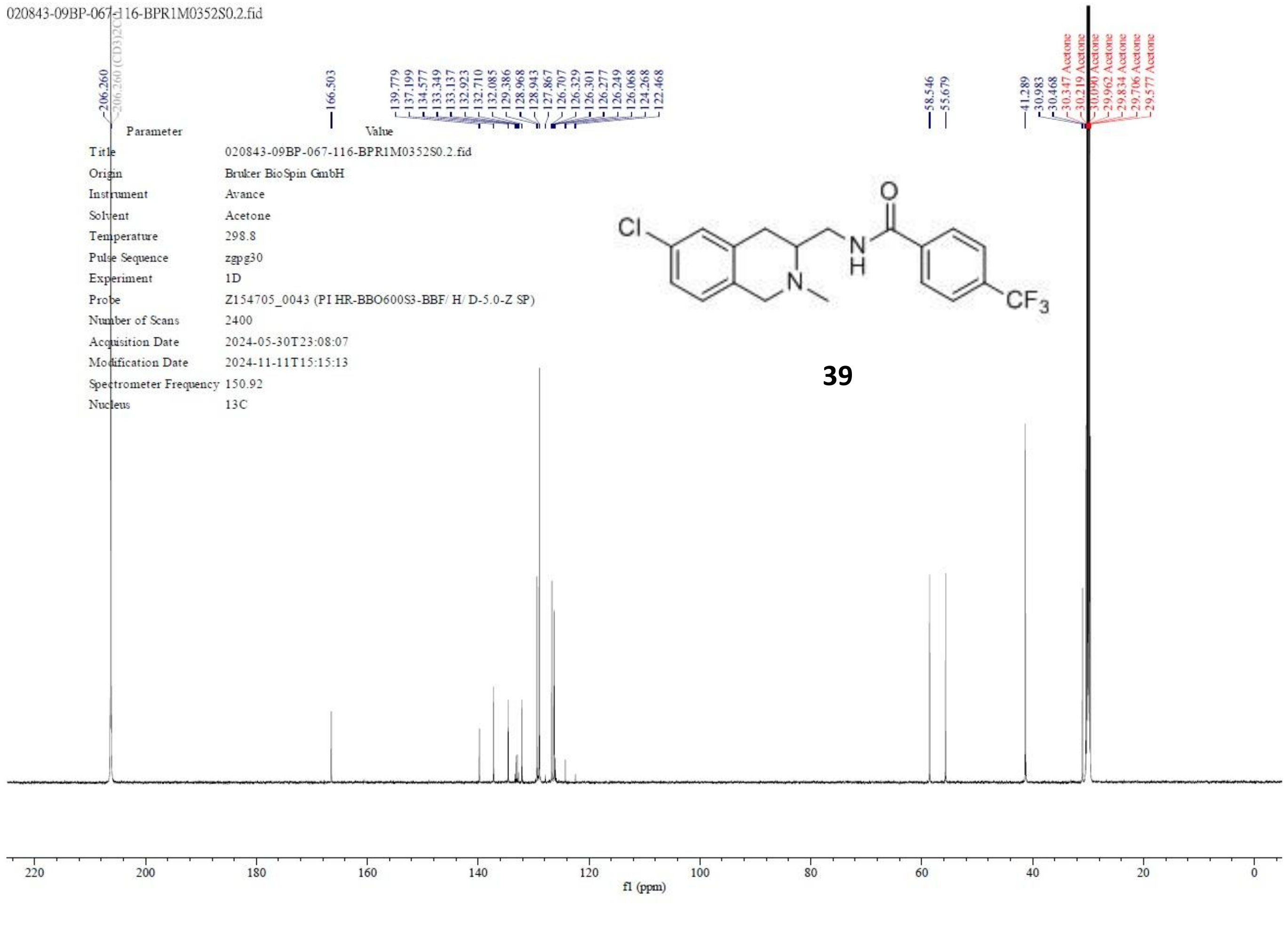

^{13}C NMR spectrum of compound **39**

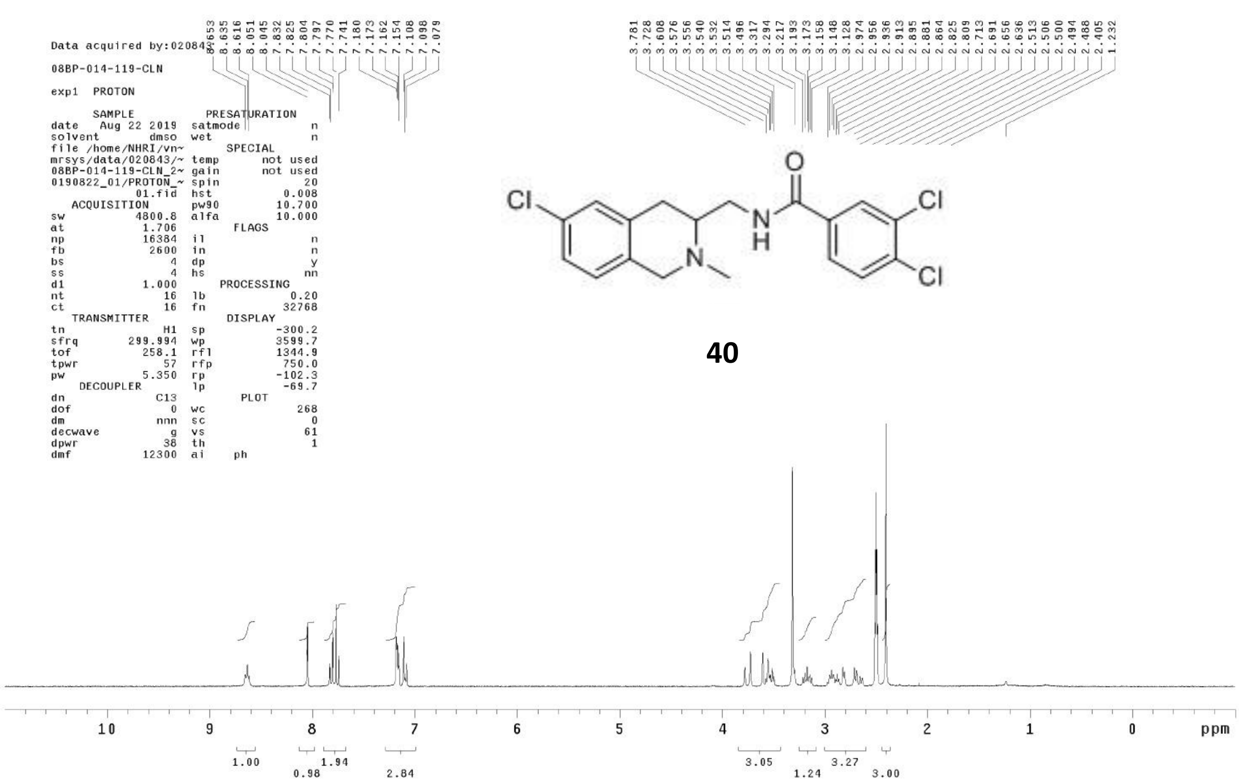

^{1}H NMR spectrum of compound **40**

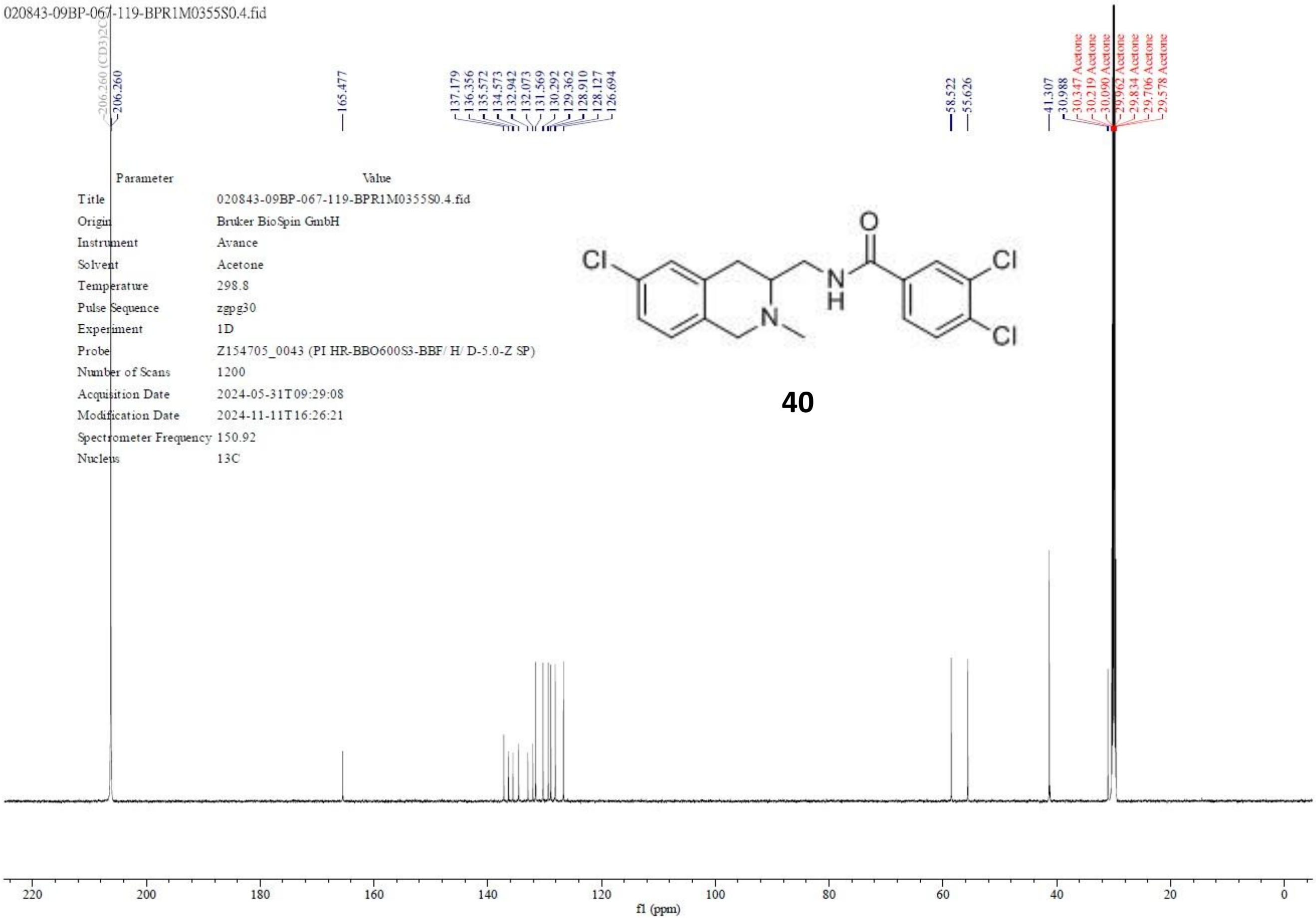

^{13}C NMR spectrum of compound **40**

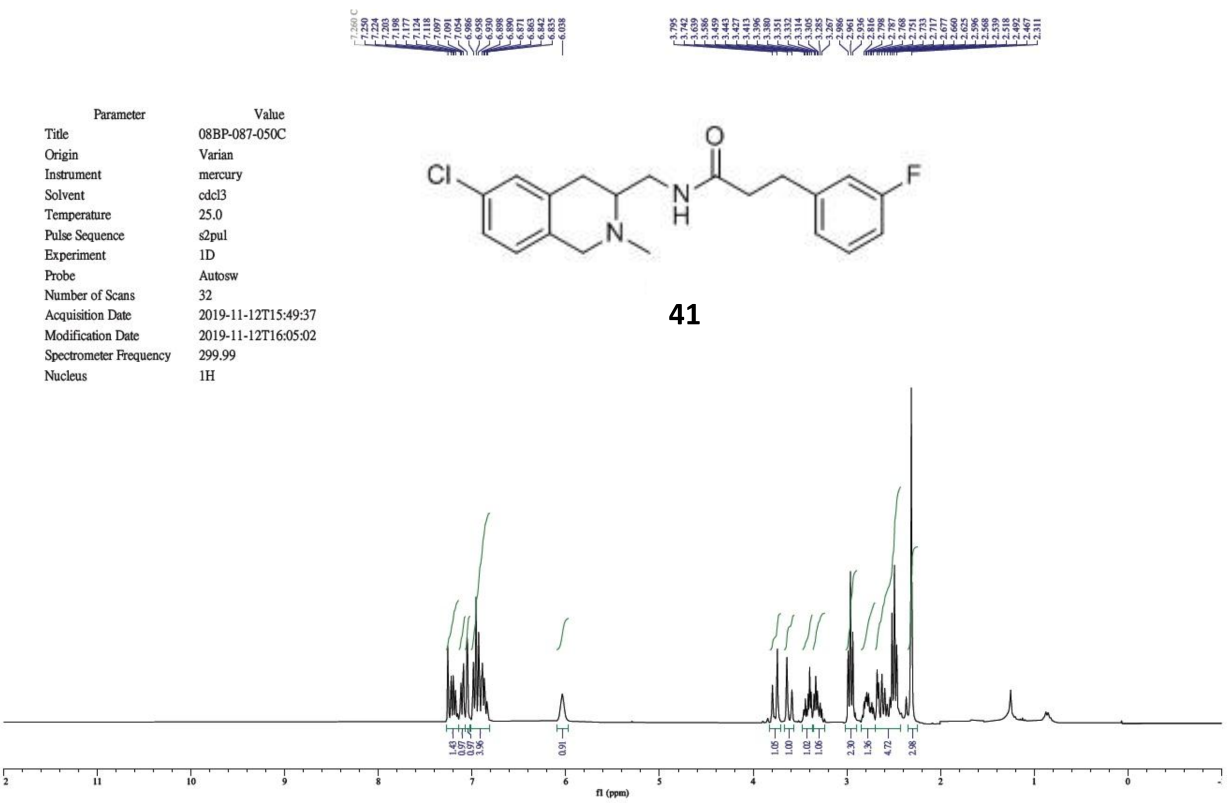

^{1}H NMR spectrum of compound **41**

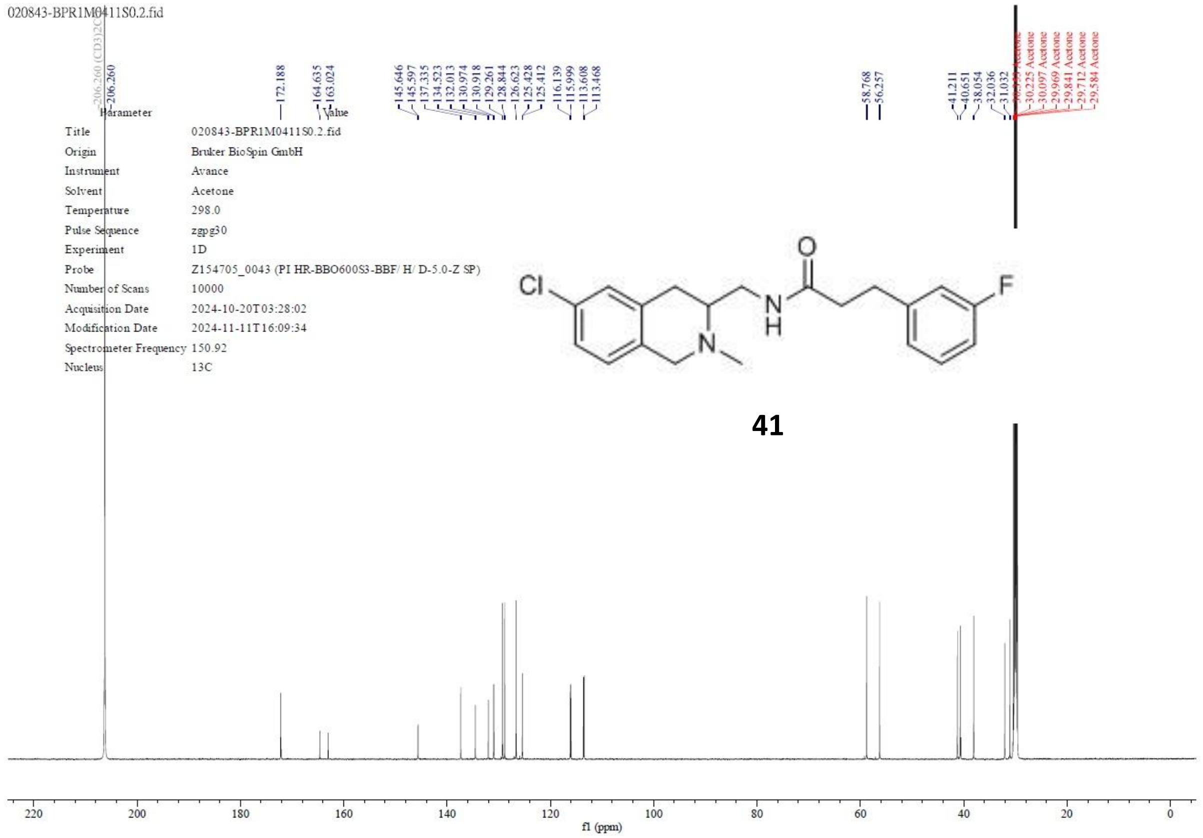

^{13}C NMR spectrum of compound **41**

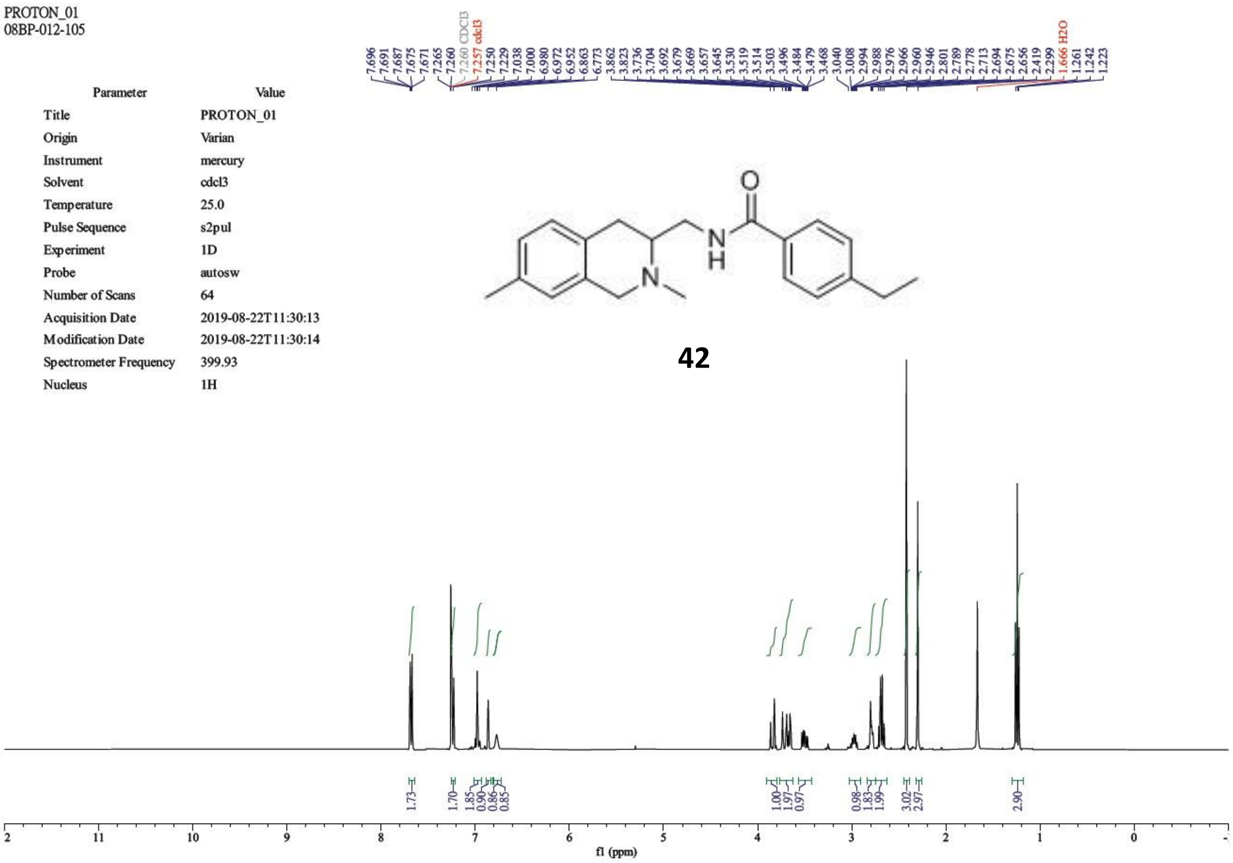

^{1}H NMR spectrum of compound **42**

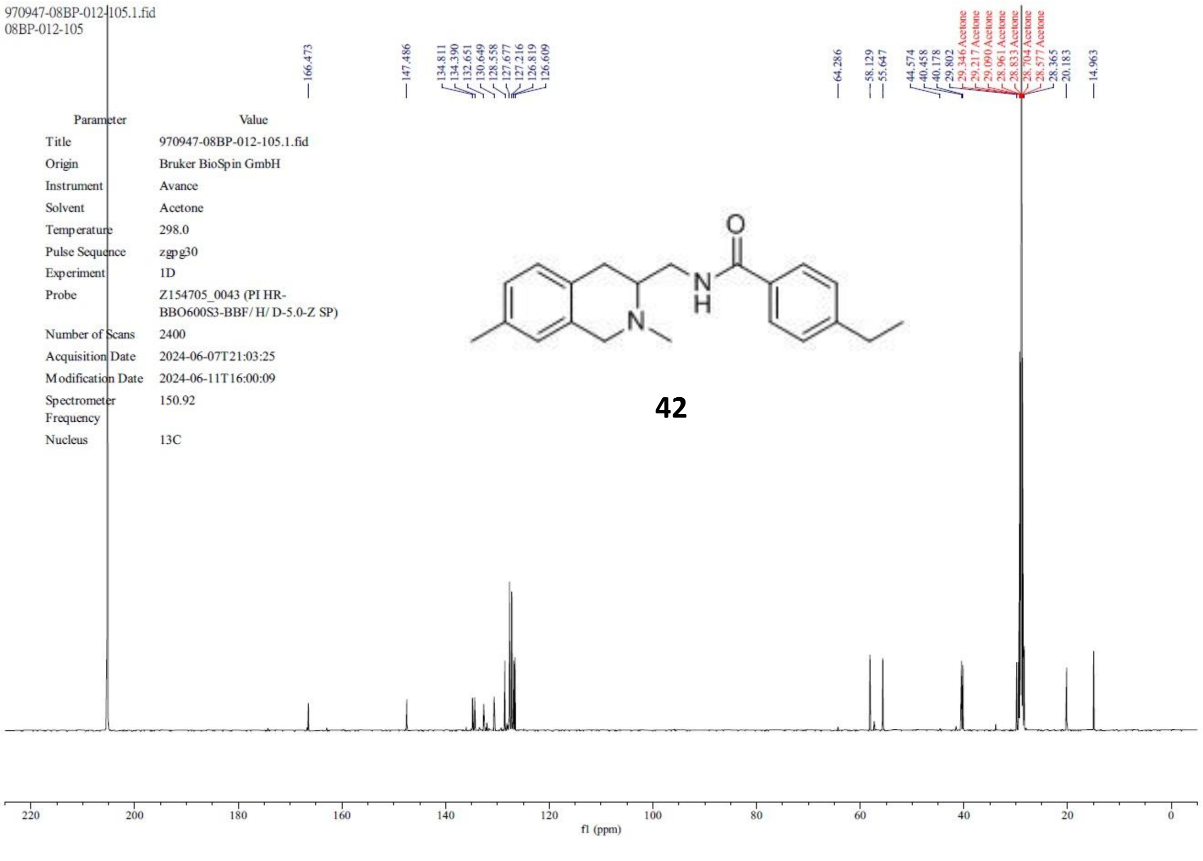

^{13}C NMR spectrum of compound **42**

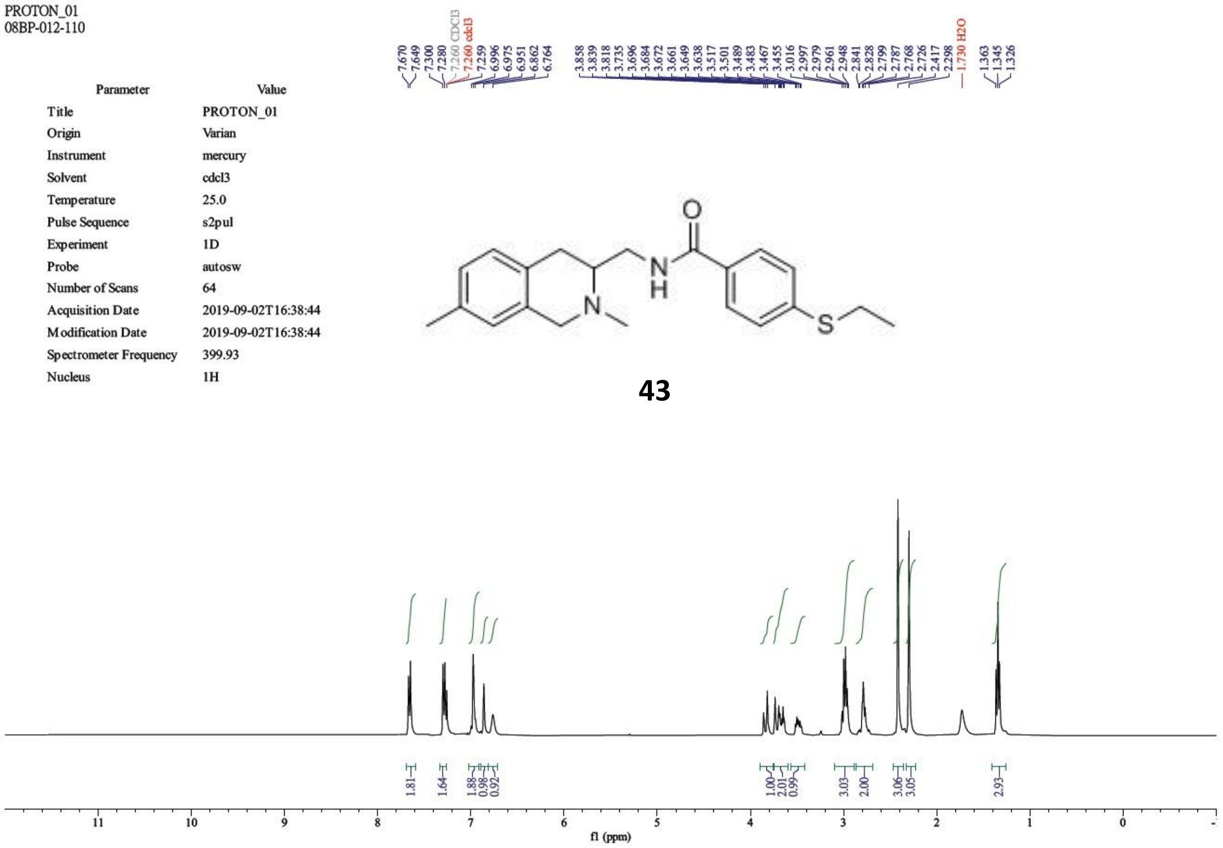

^{1}H NMR spectrum of compound **43**

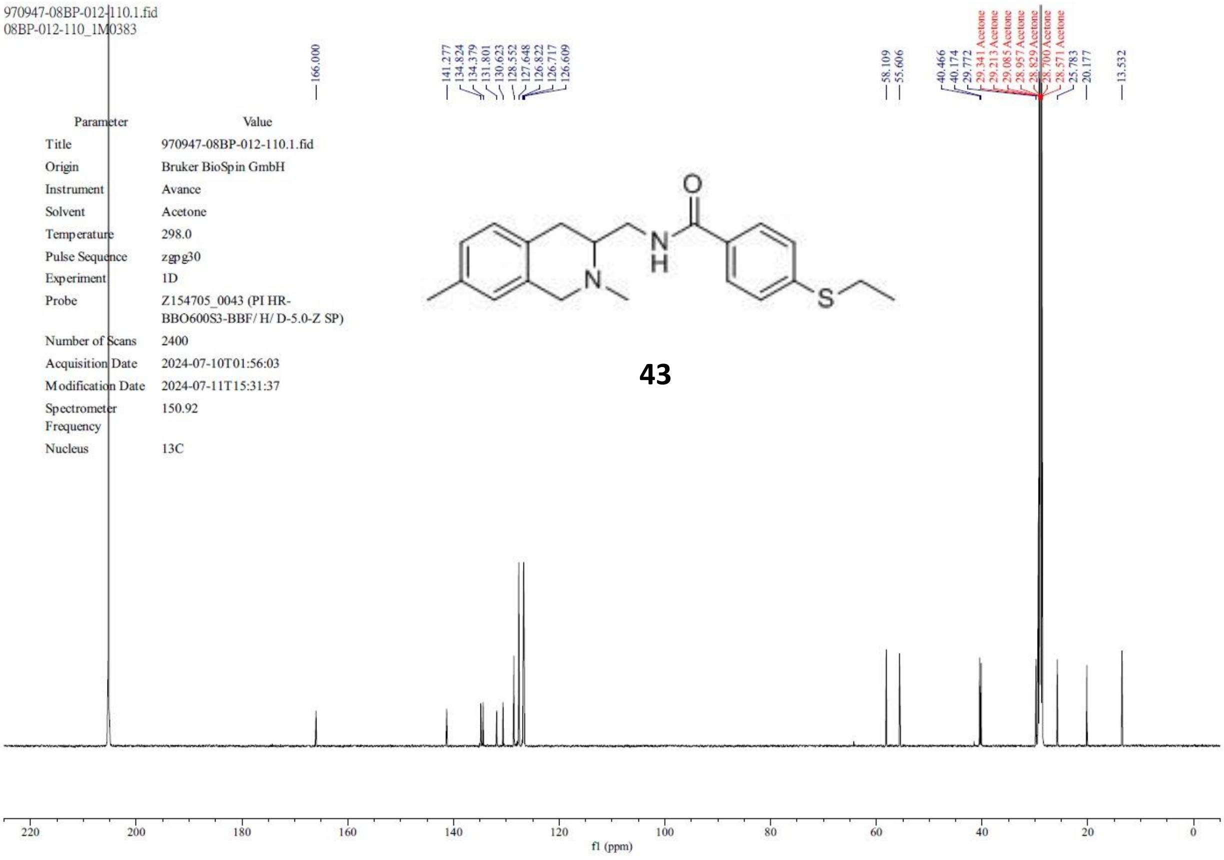

^{13}C NMR spectrum of compound **43**

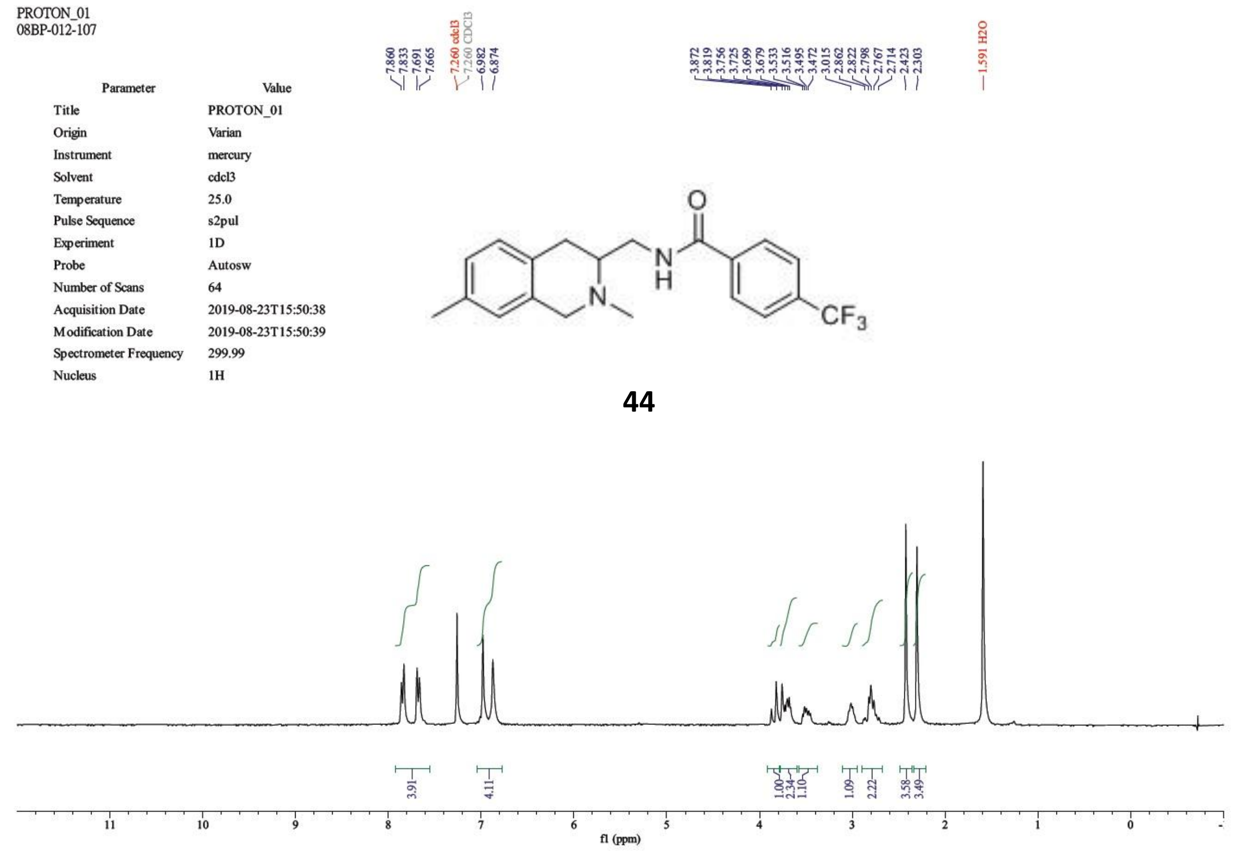

^{1}H NMR spectrum of compound **44**

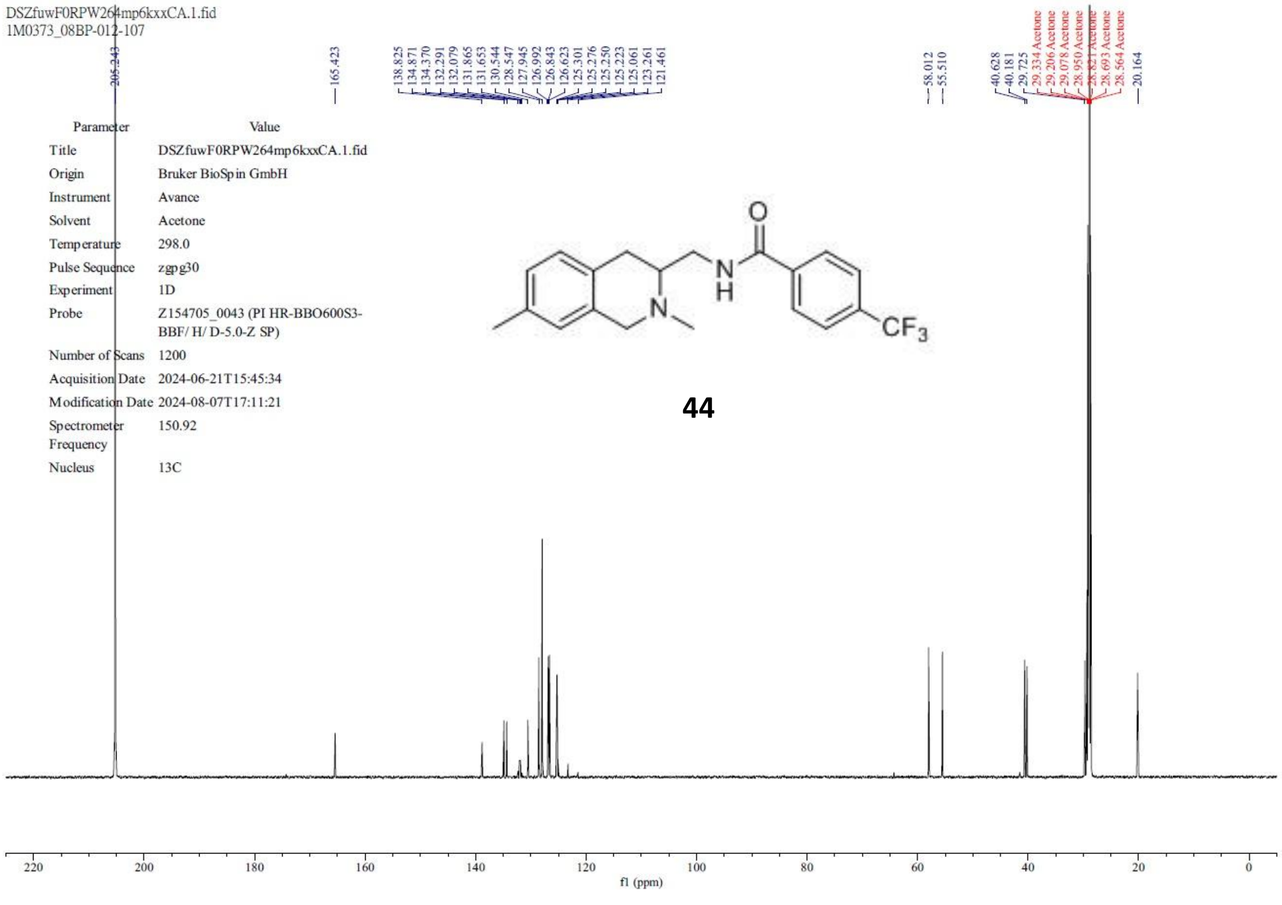

^{13}C NMR spectrum of compound **44**

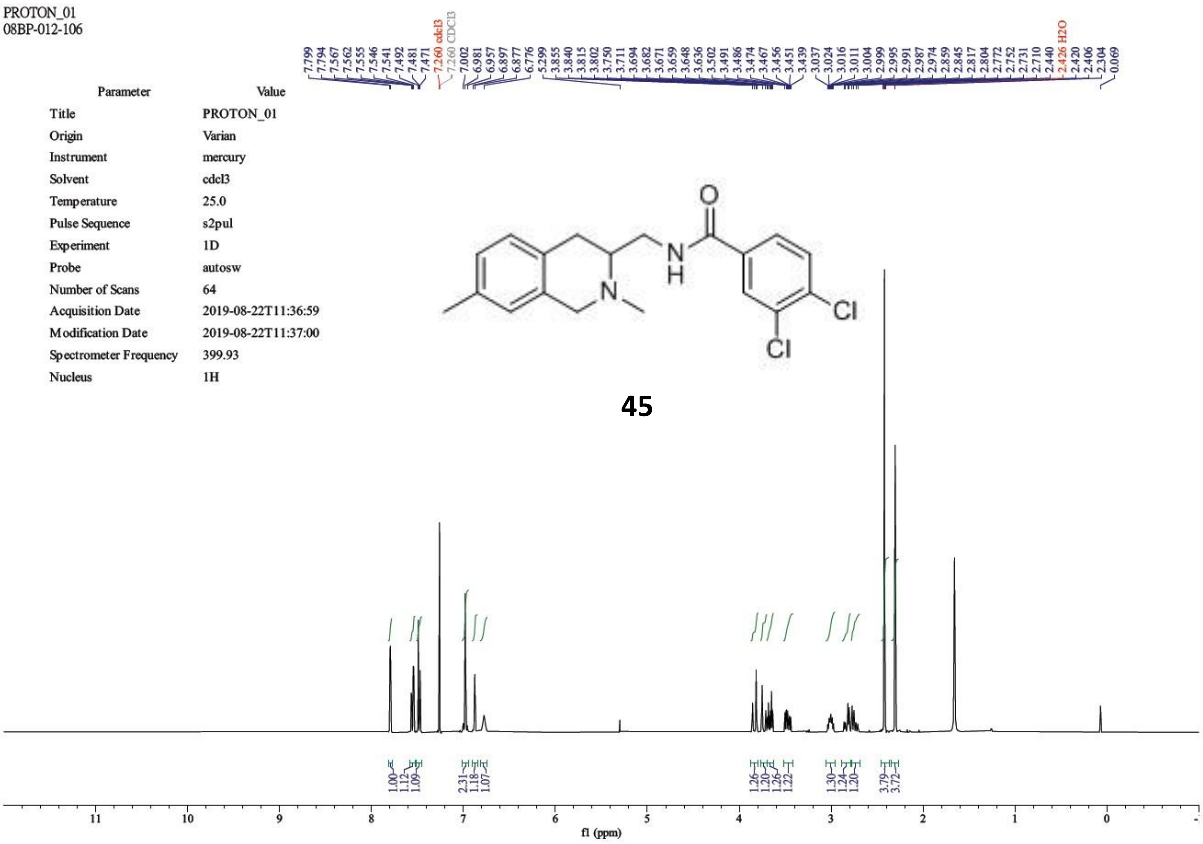

^{1}H NMR spectrum of compound **45**

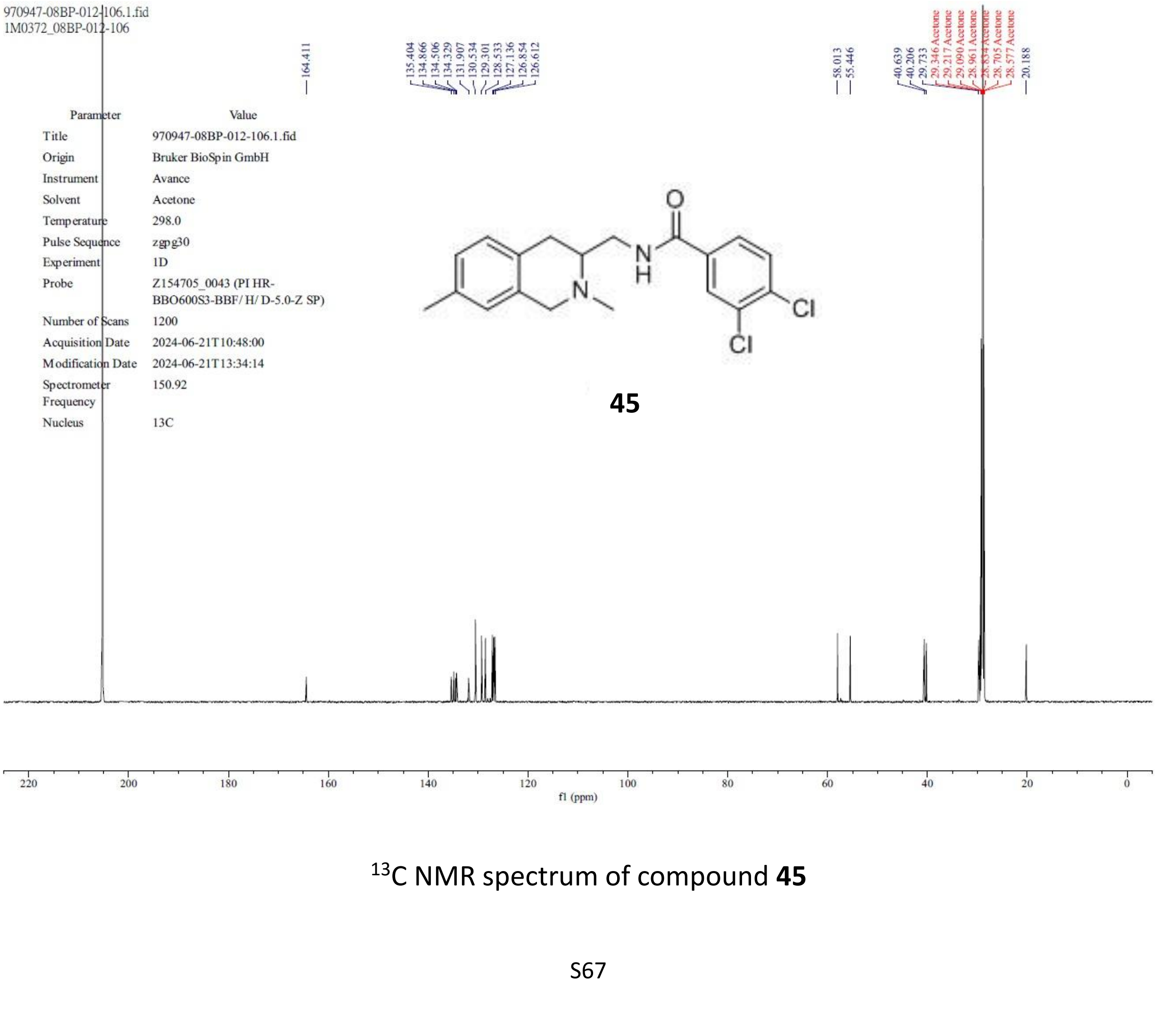

^{13}C NMR spectrum of compound **45**

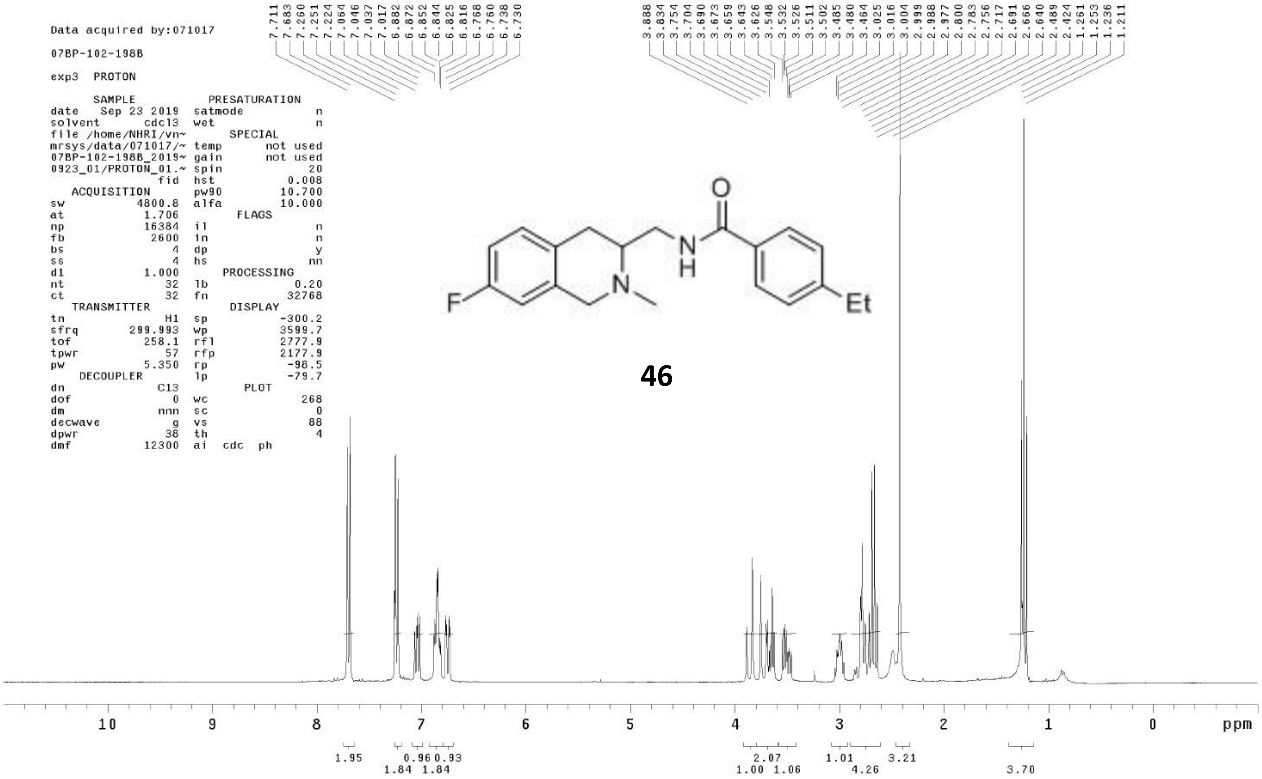

^{1}H NMR spectrum of compound **46**

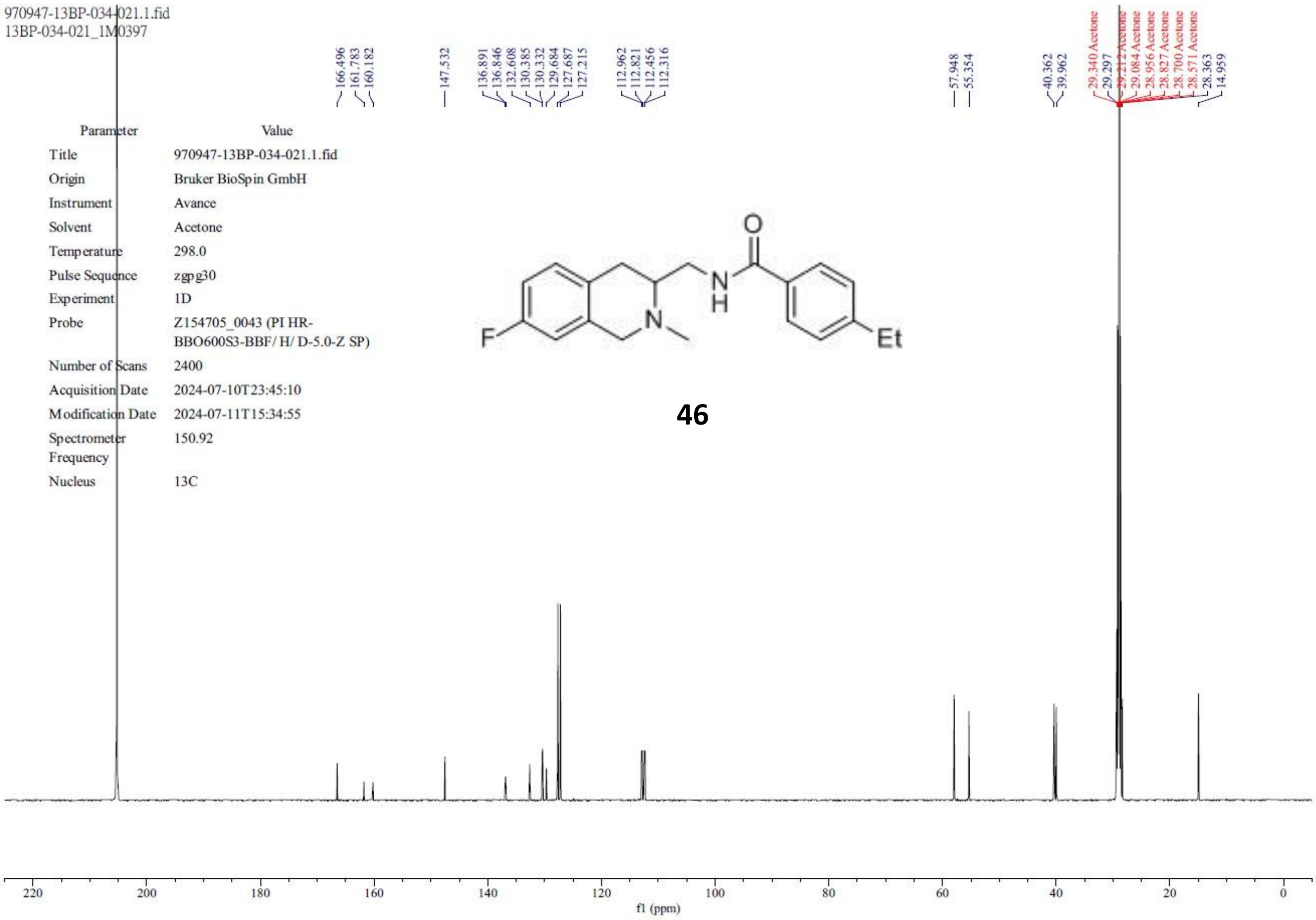

^{13}C NMR spectrum of compound **46**

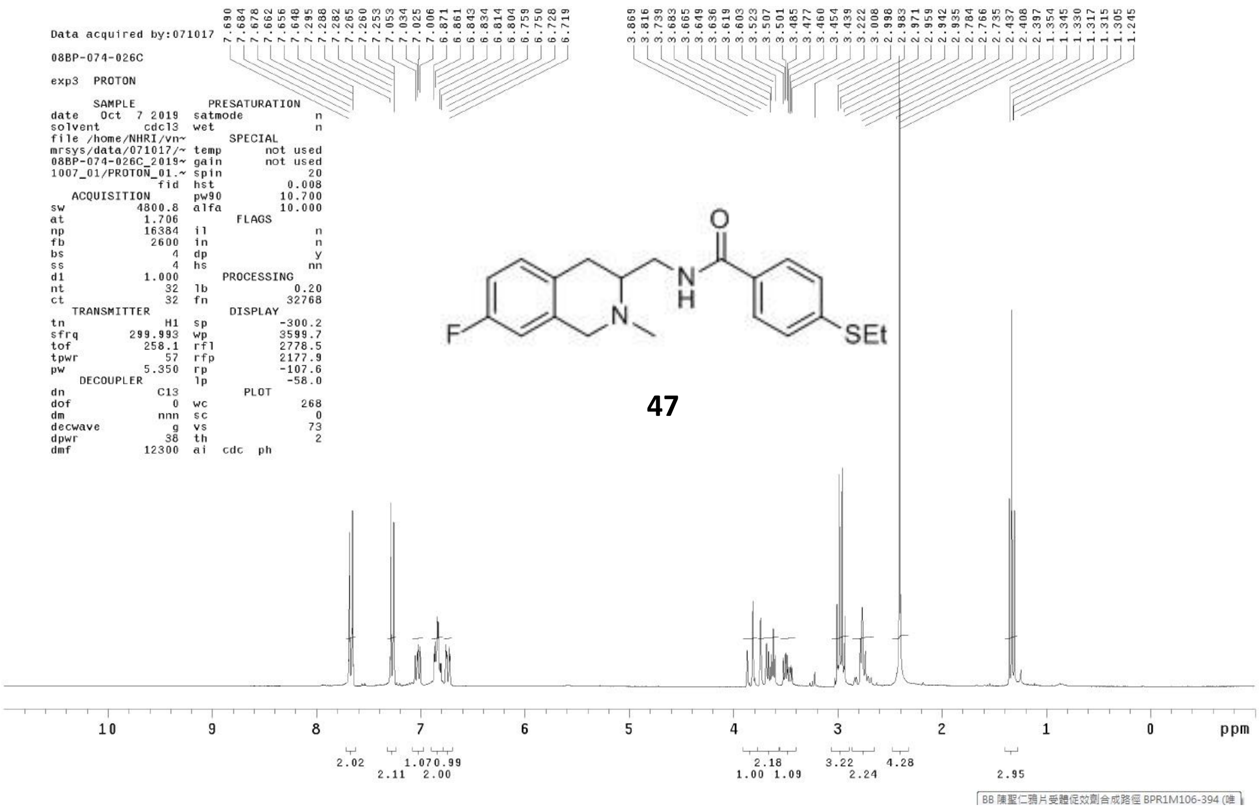

^{1}H NMR spectrum of compound **47**

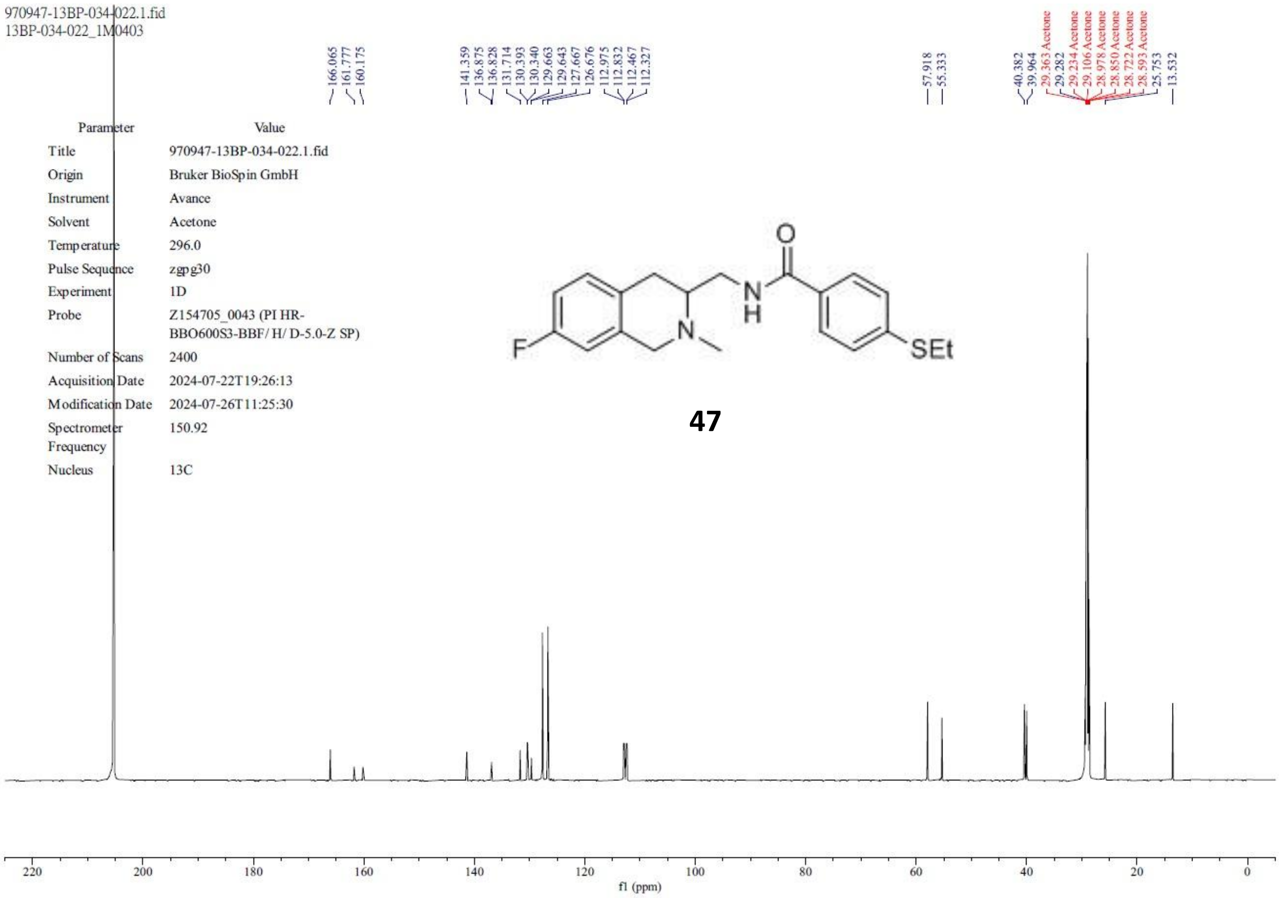

^{13}C NMR spectrum of compound **47**

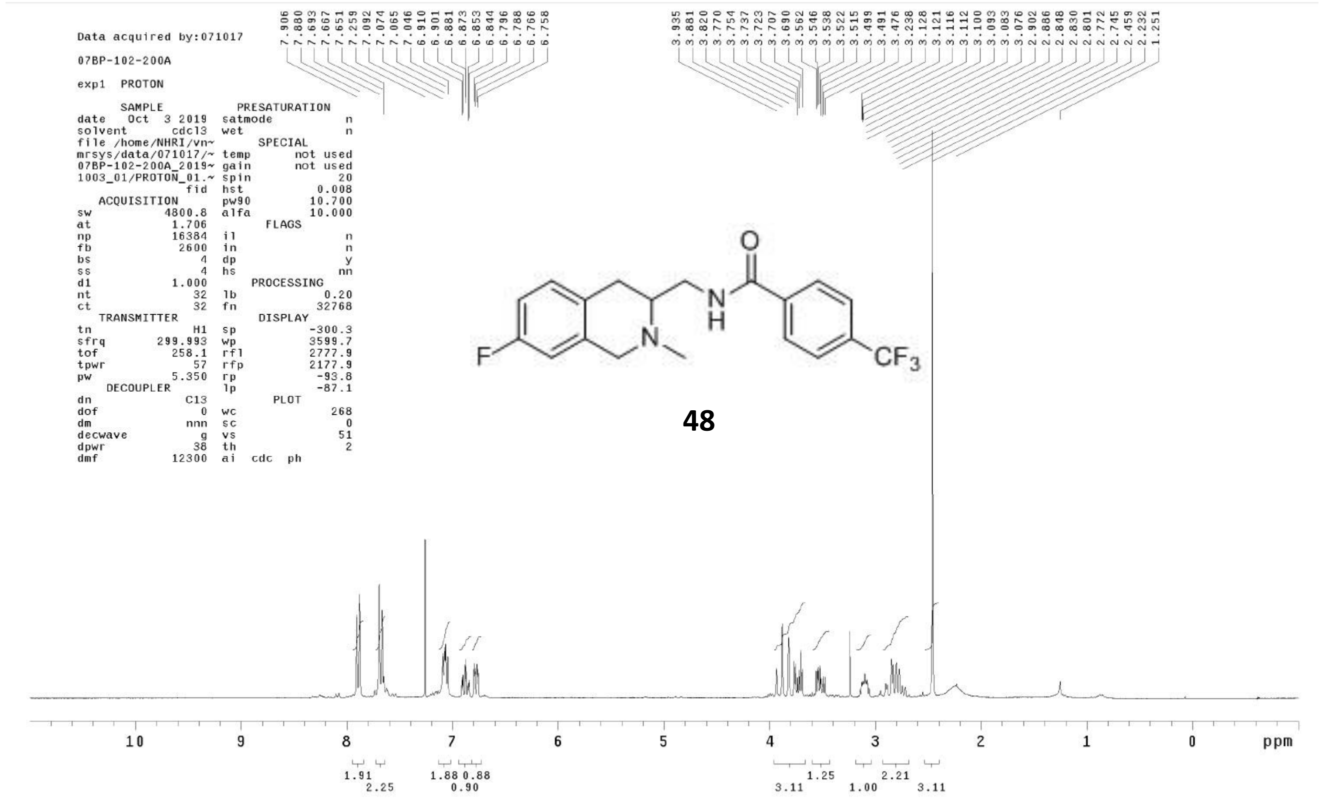

^{1}H NMR spectrum of compound **48**

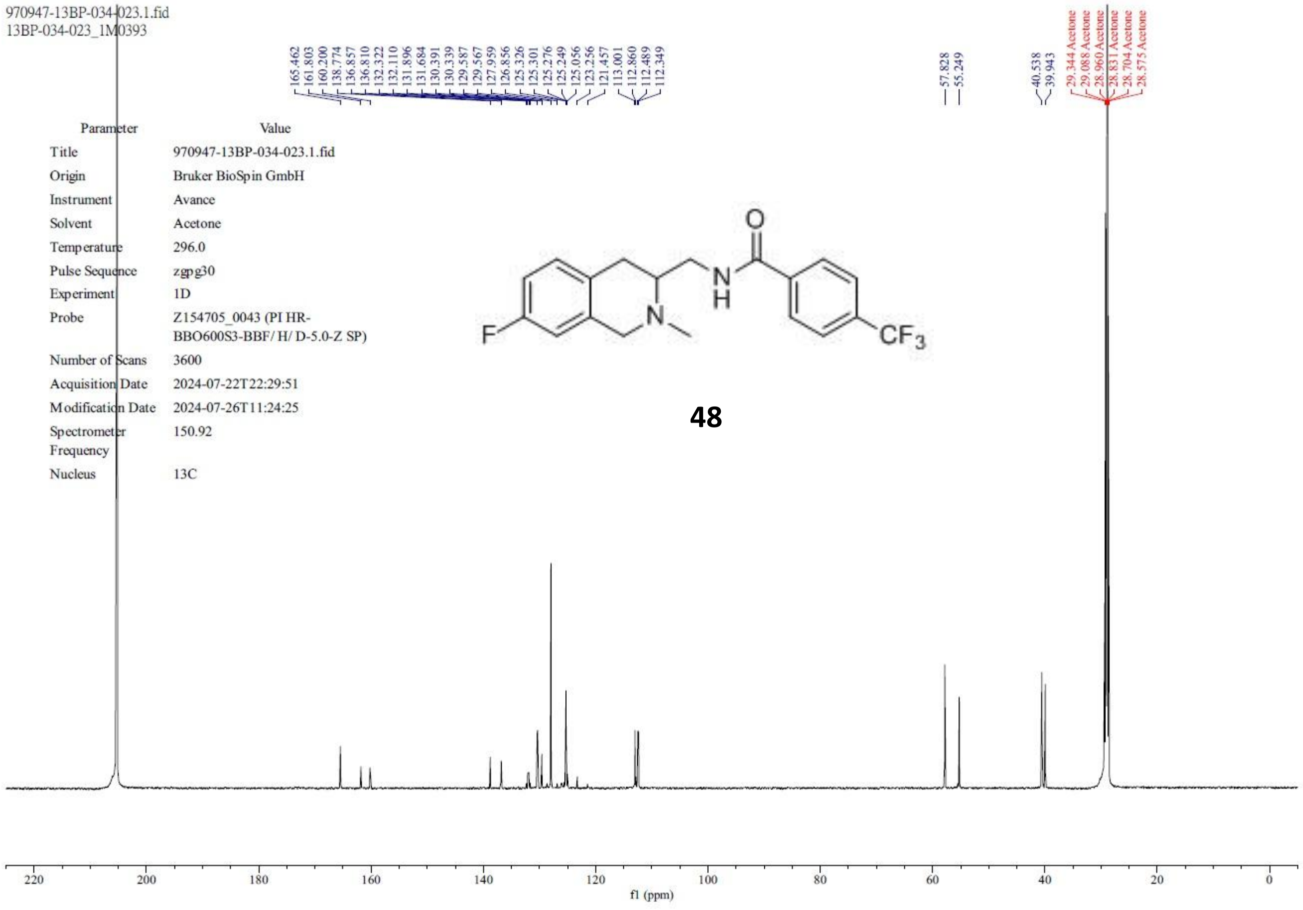

^{13}C NMR spectrum of compound **48**

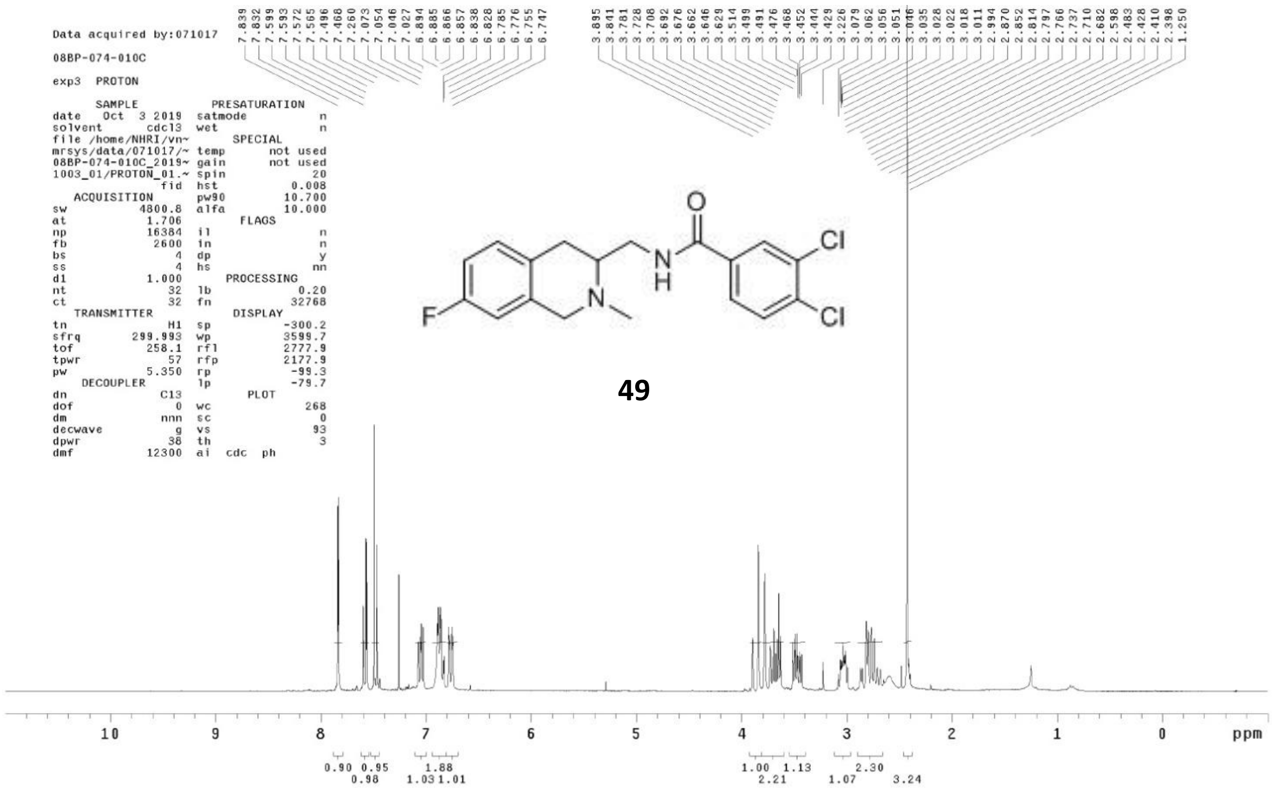

^{1}H NMR spectrum of compound **49**

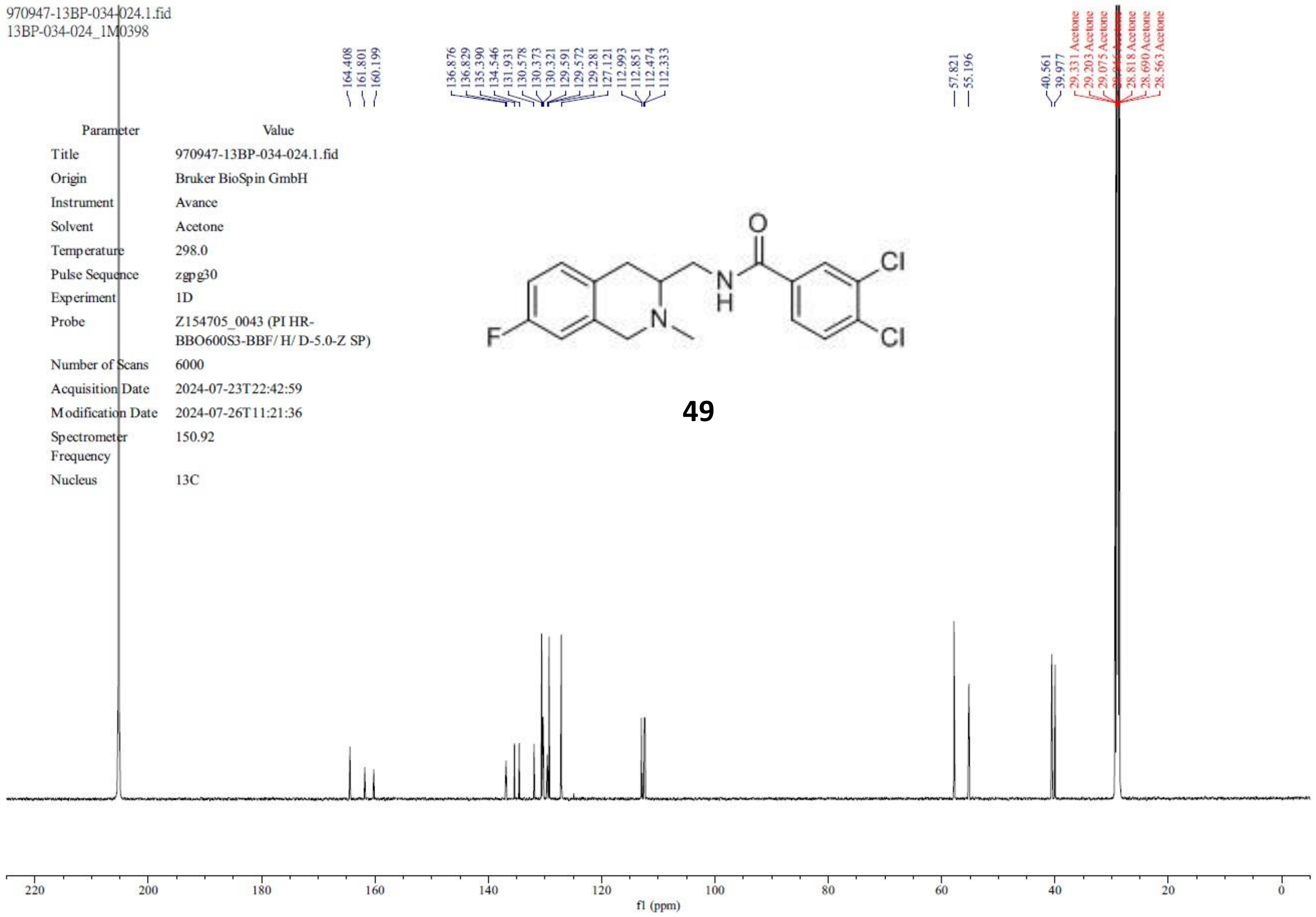

^{13}C NMR spectrum of compound **49**

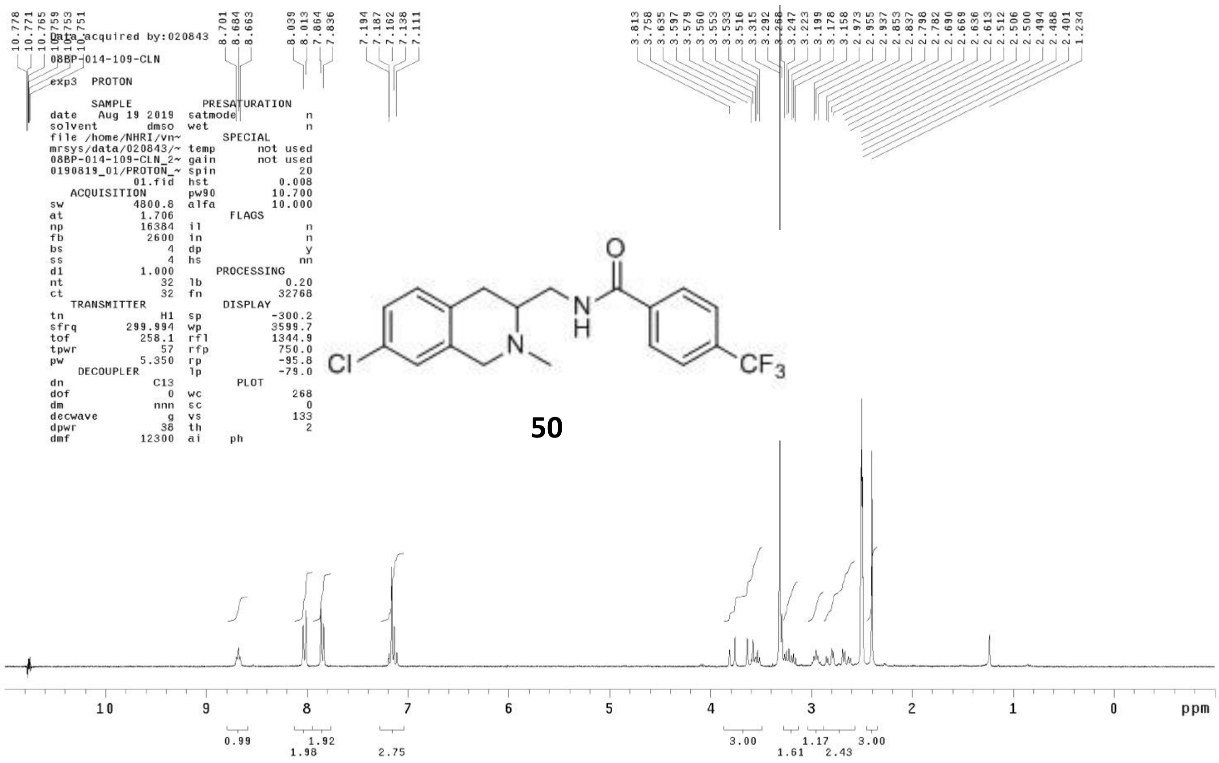

^{1}H NMR spectrum of compound **50**

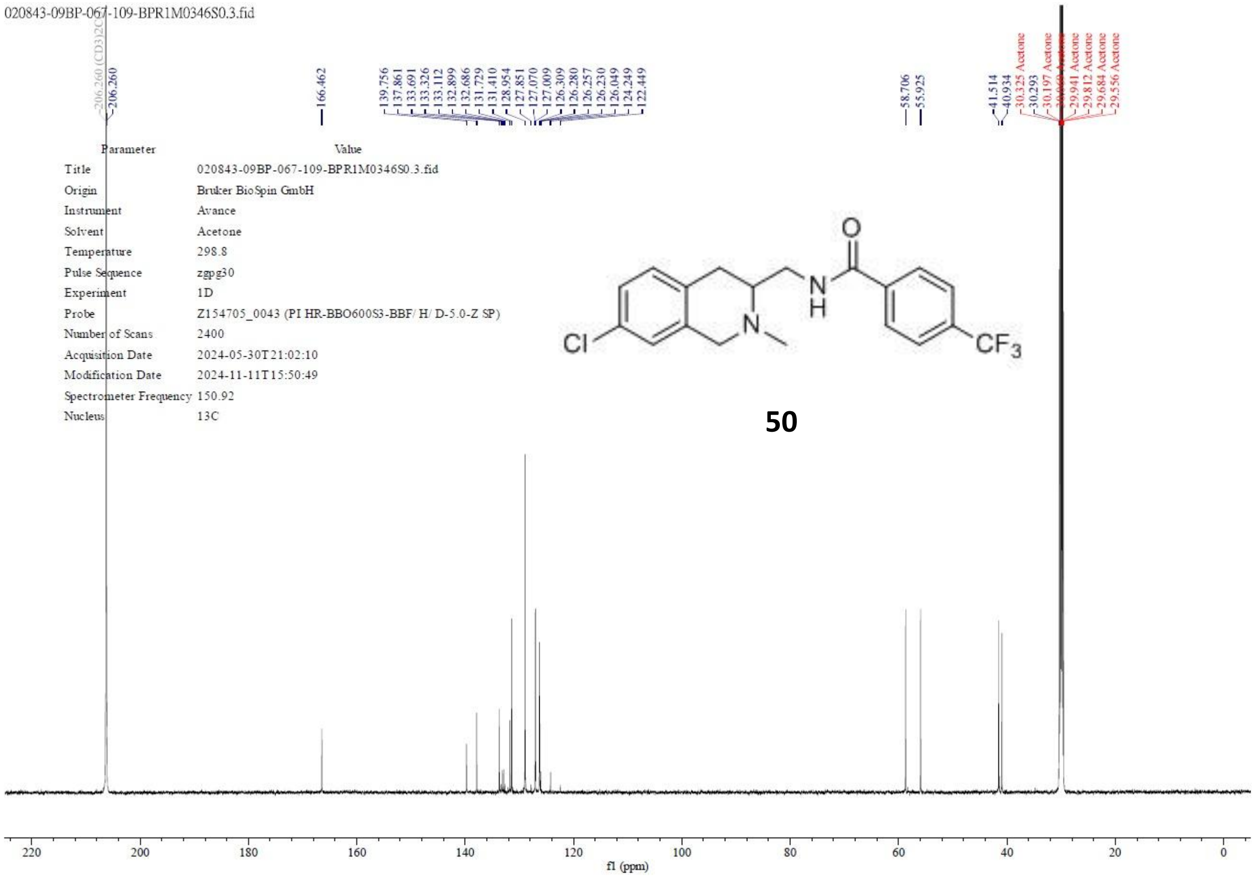

^{13}C NMR spectrum of compound **50**

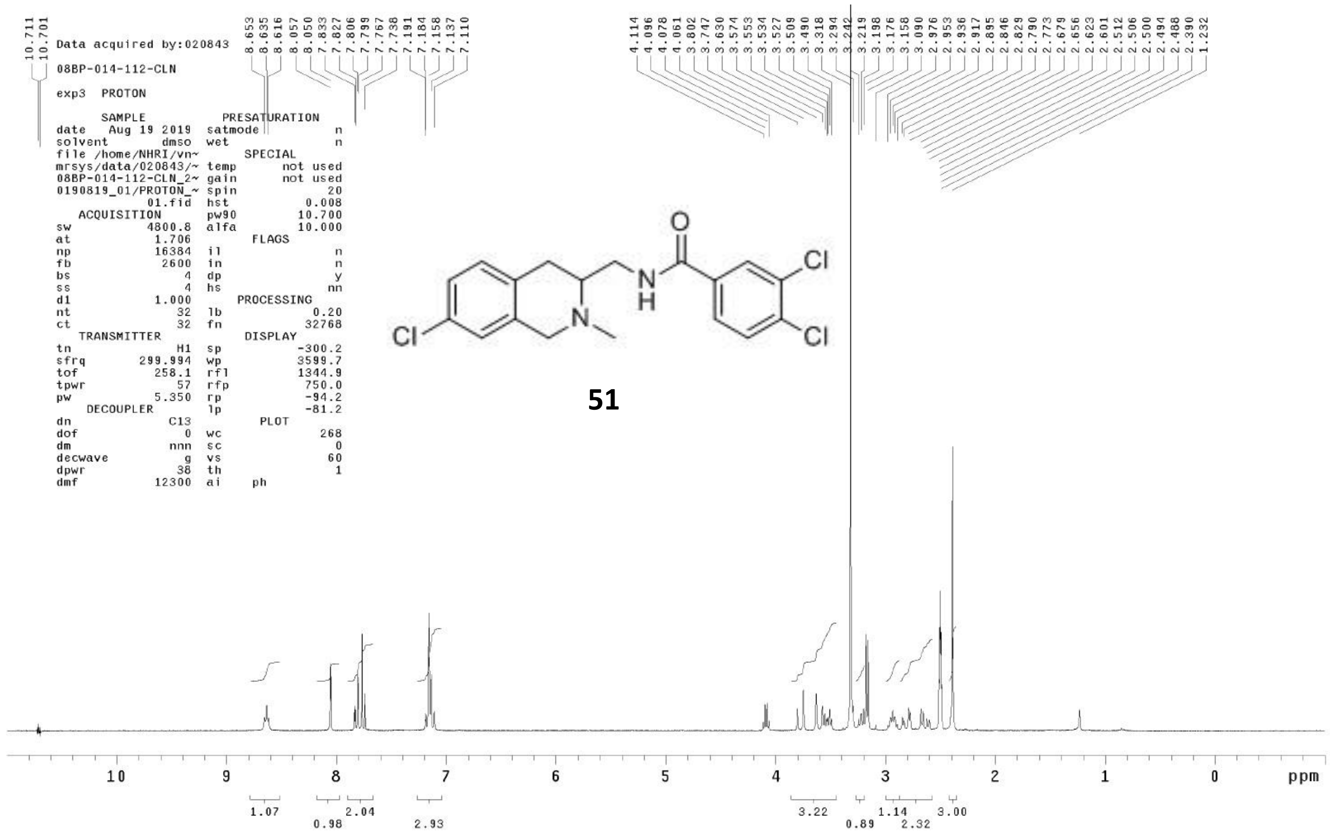

51

^1H NMR spectrum of compound **51**

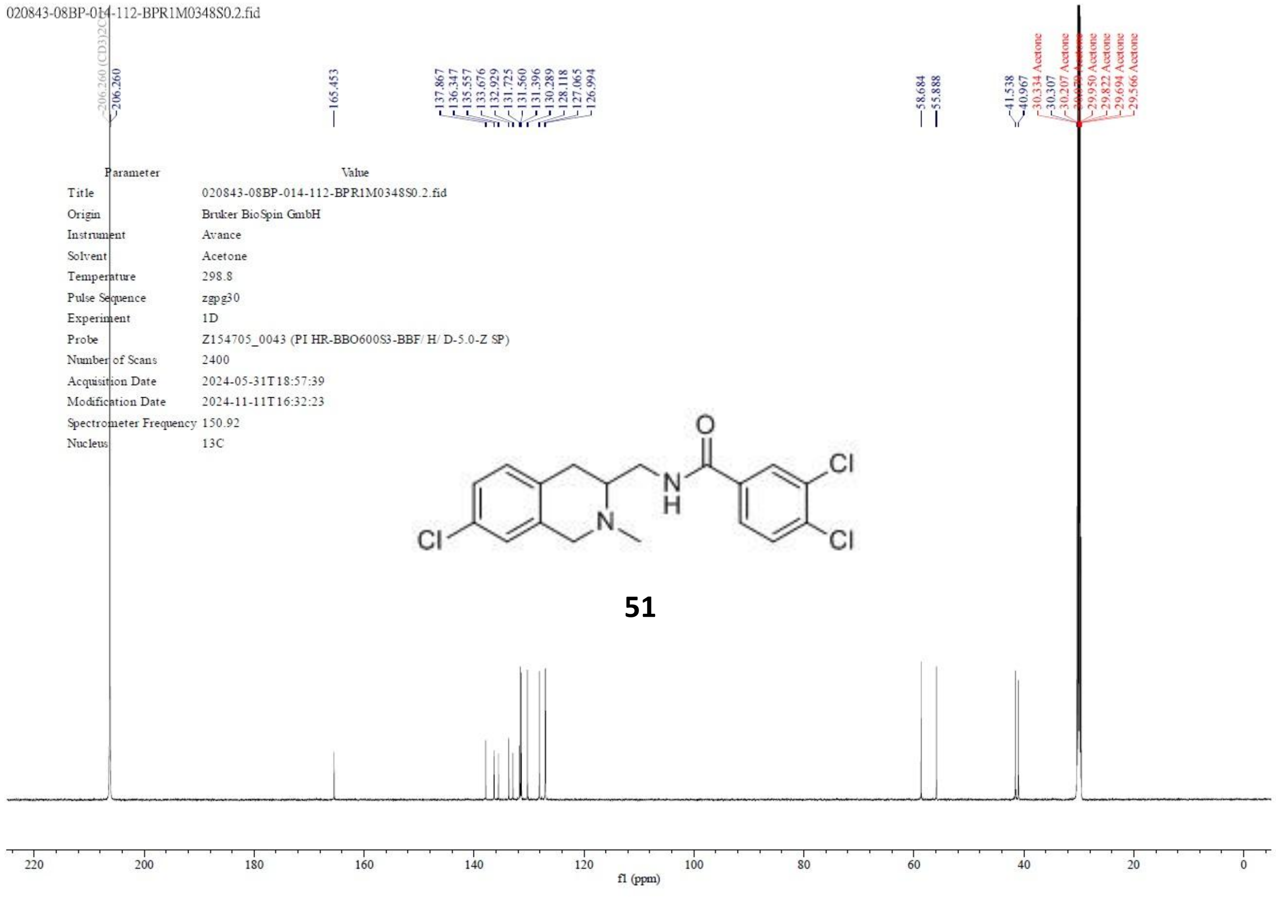

51

^{13}C NMR spectrum of compound **51**

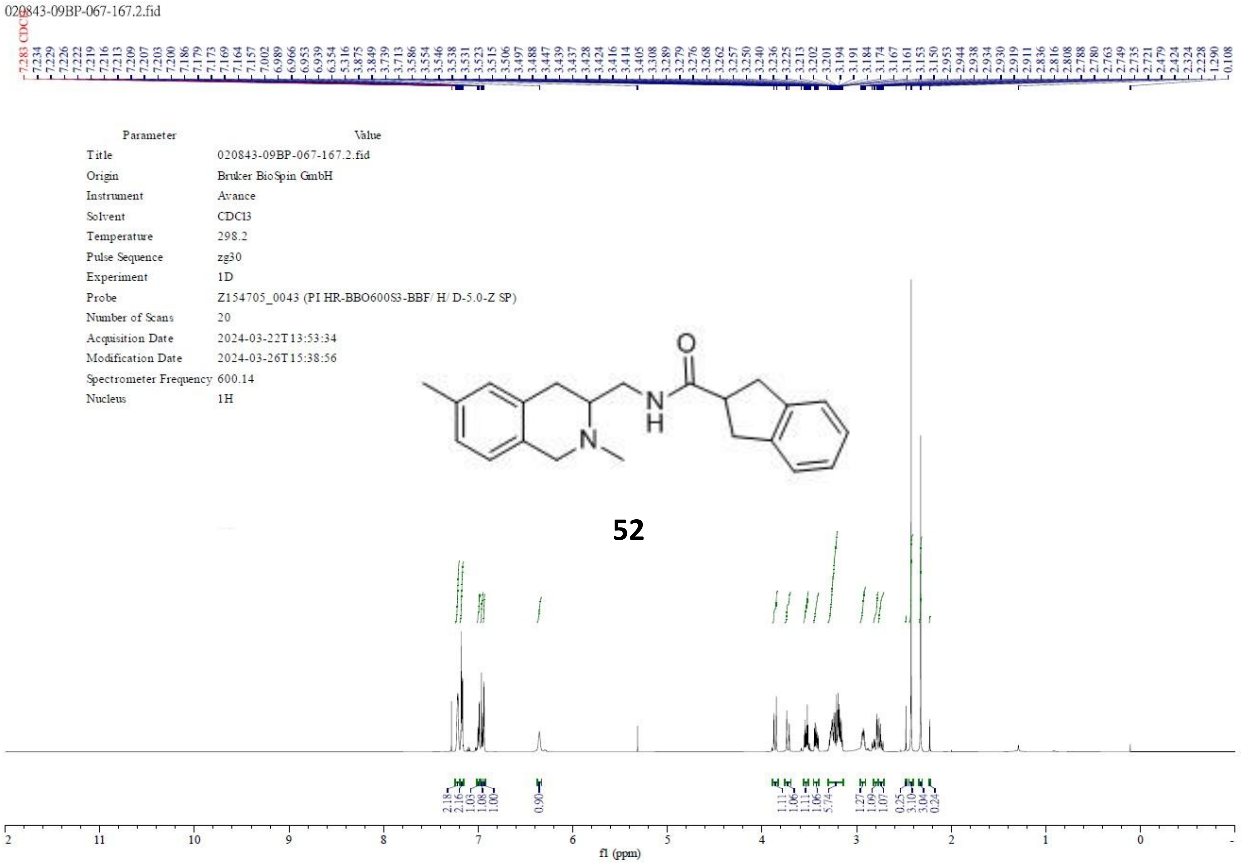

^{1}H NMR spectrum of compound **52**

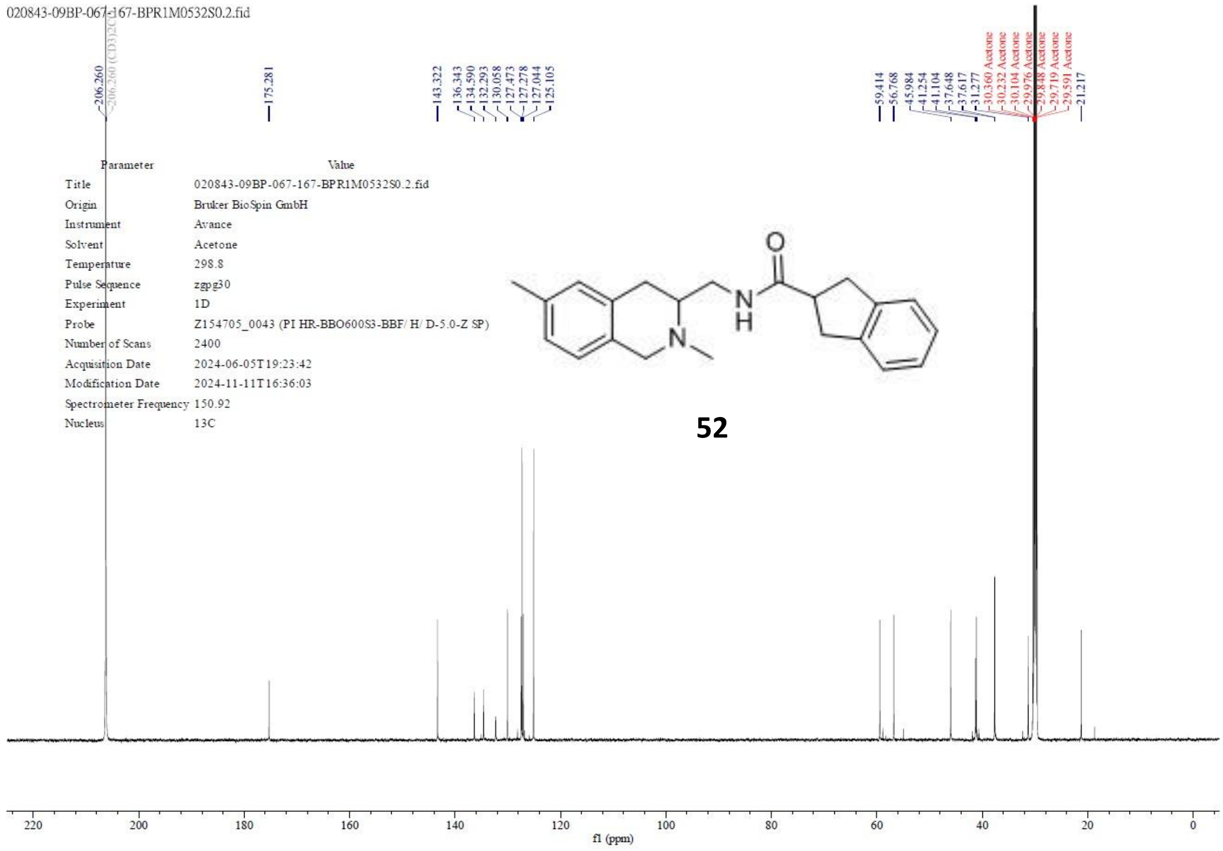

^{13}C NMR spectrum of compound **52**

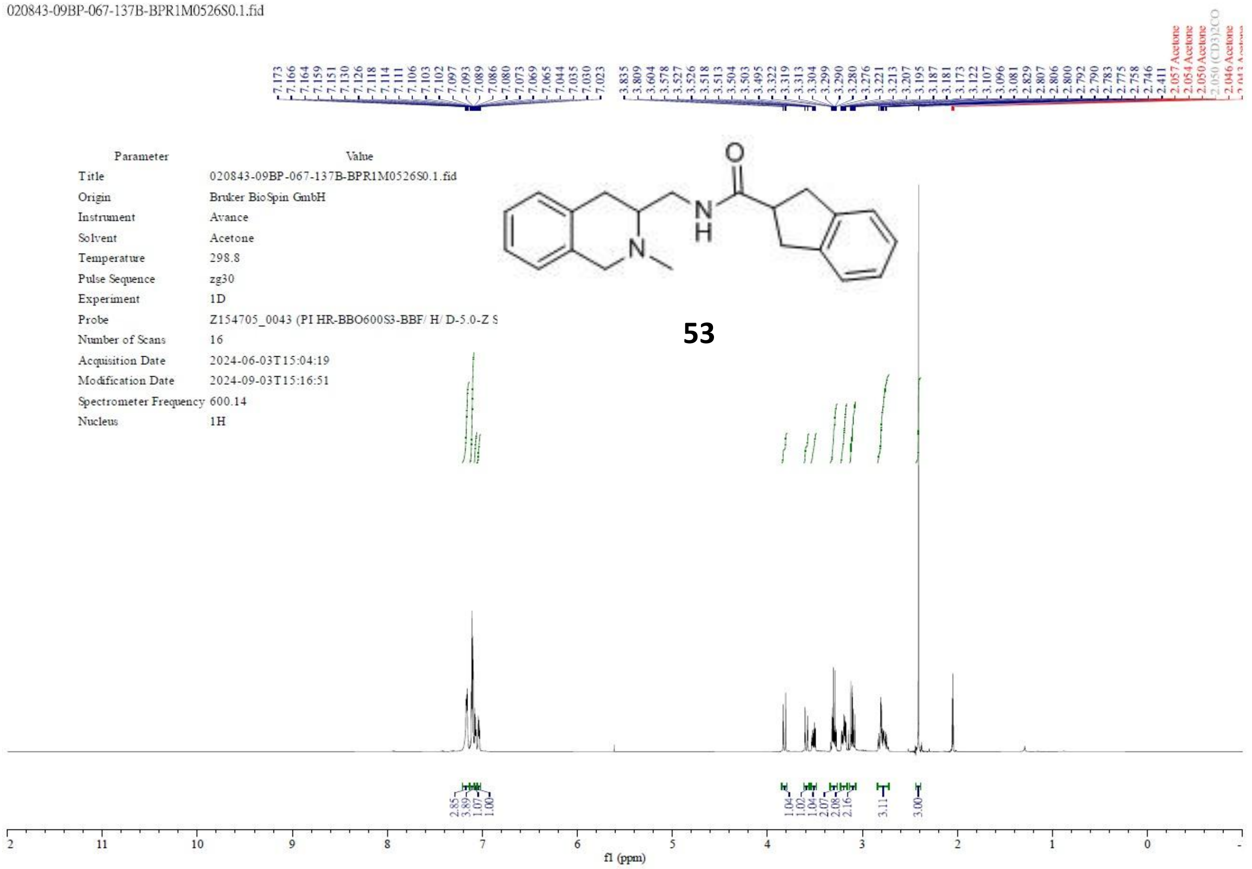

^{1}H NMR spectrum of compound **53**

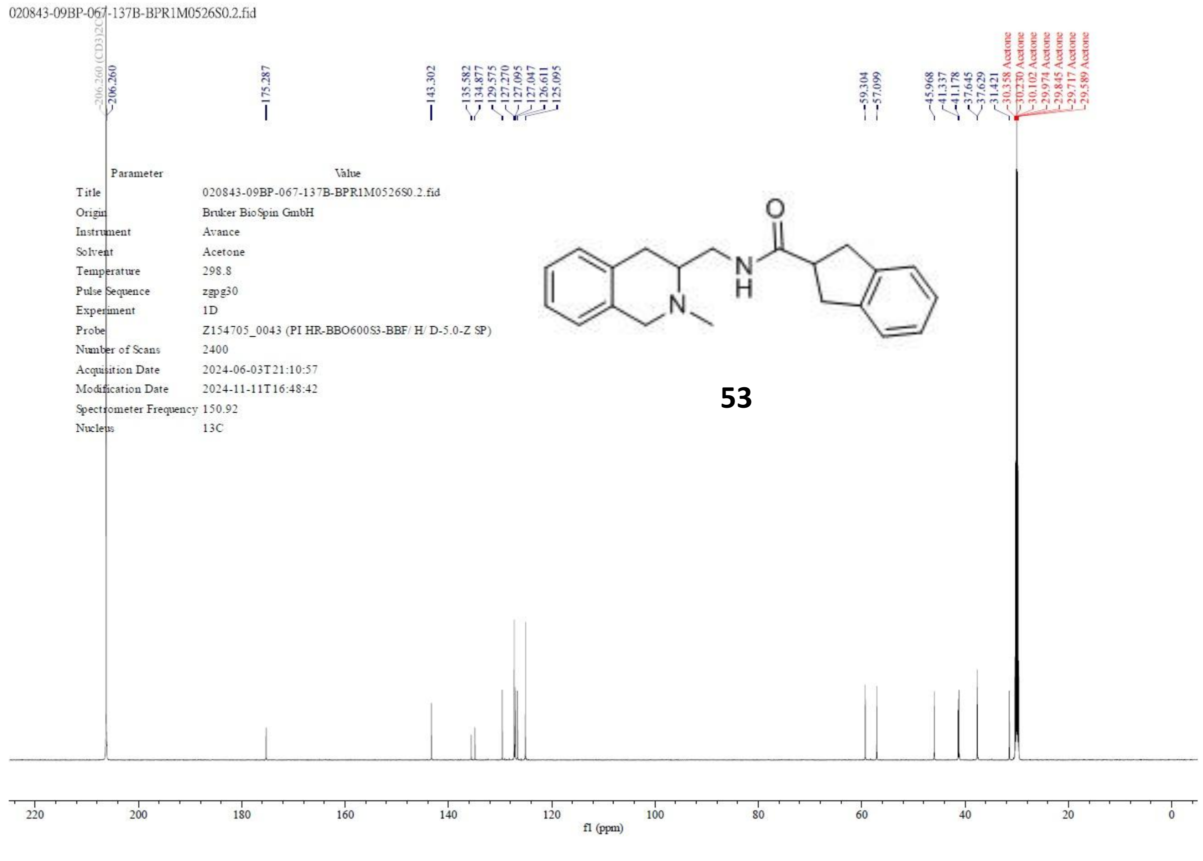

^{13}C NMR spectrum of compound **53**

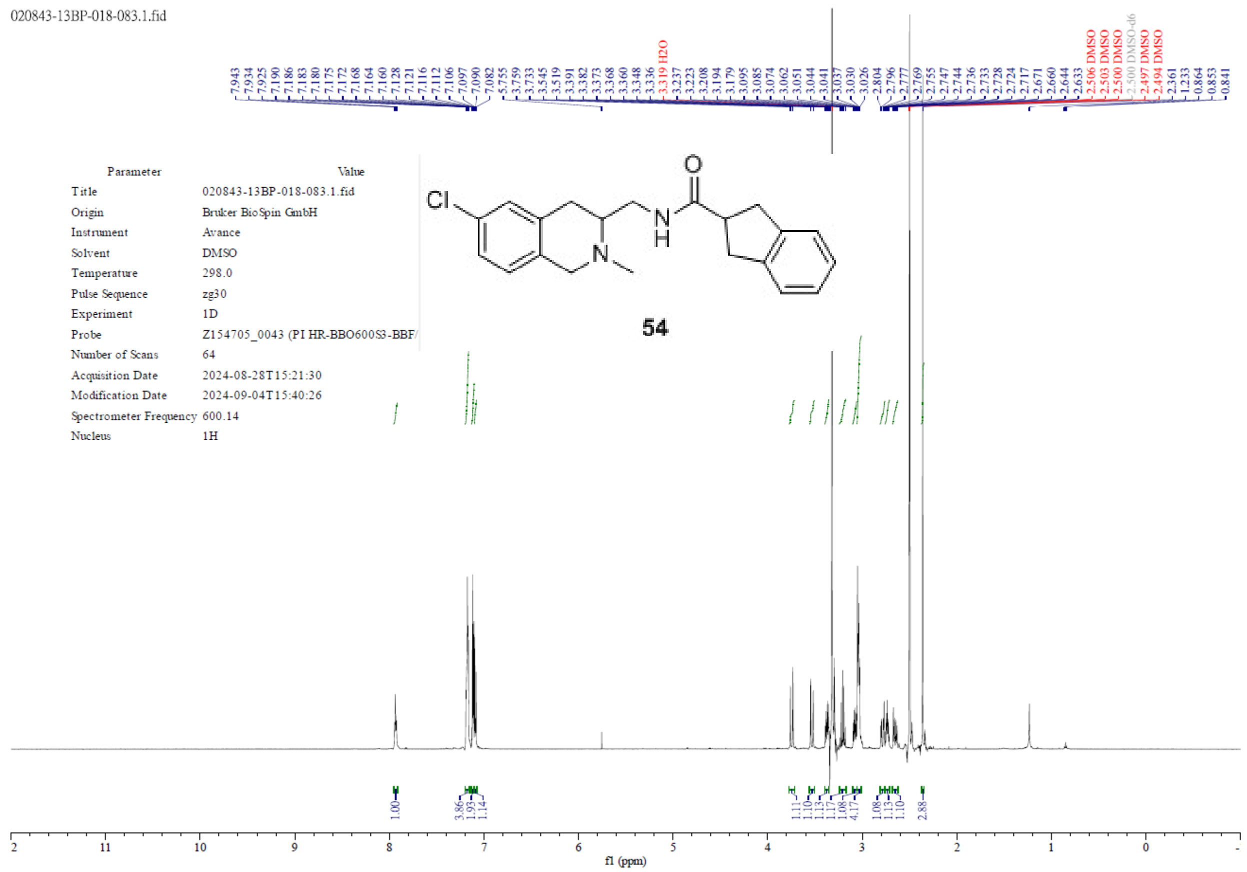

^1H NMR spectrum of compound **54**

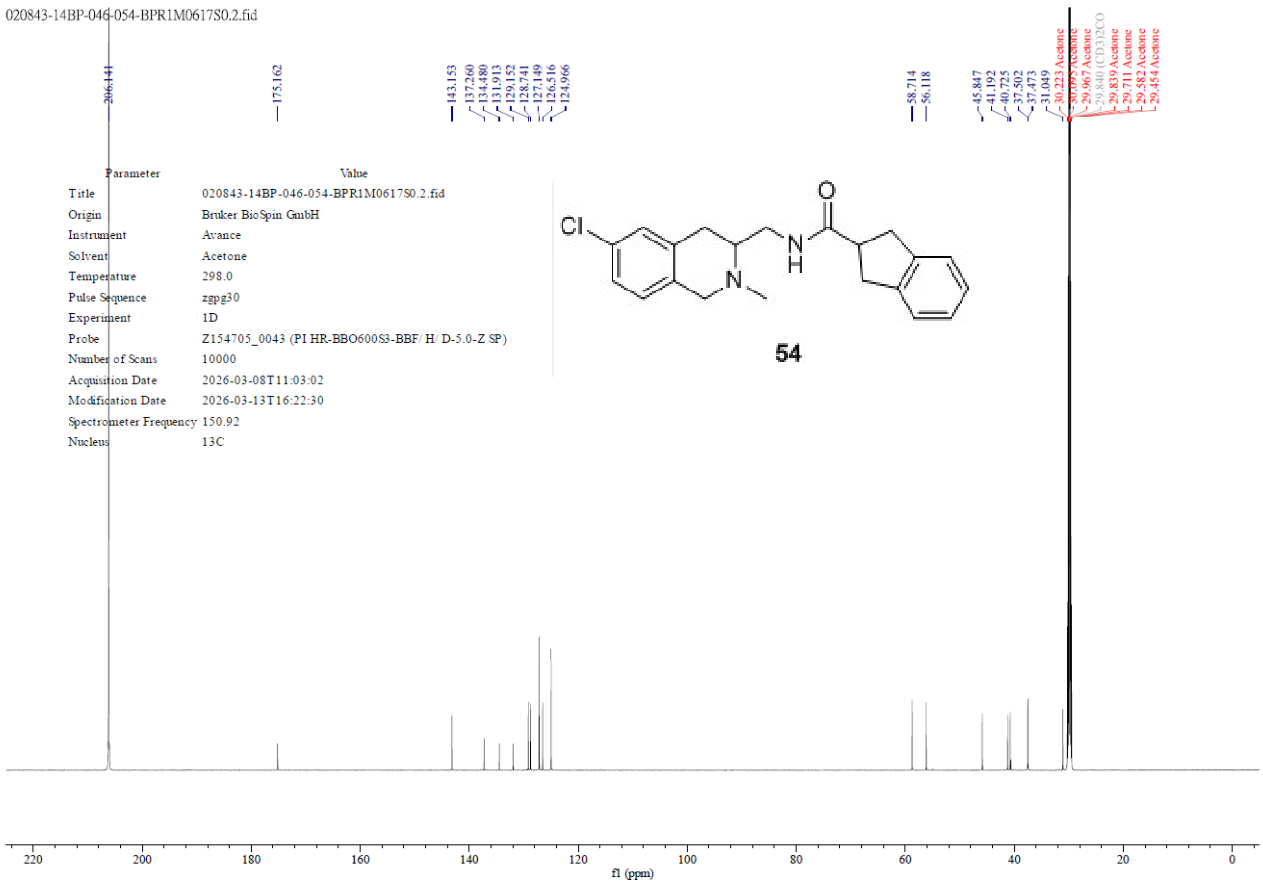

^{13}C NMR spectrum of compound **54**

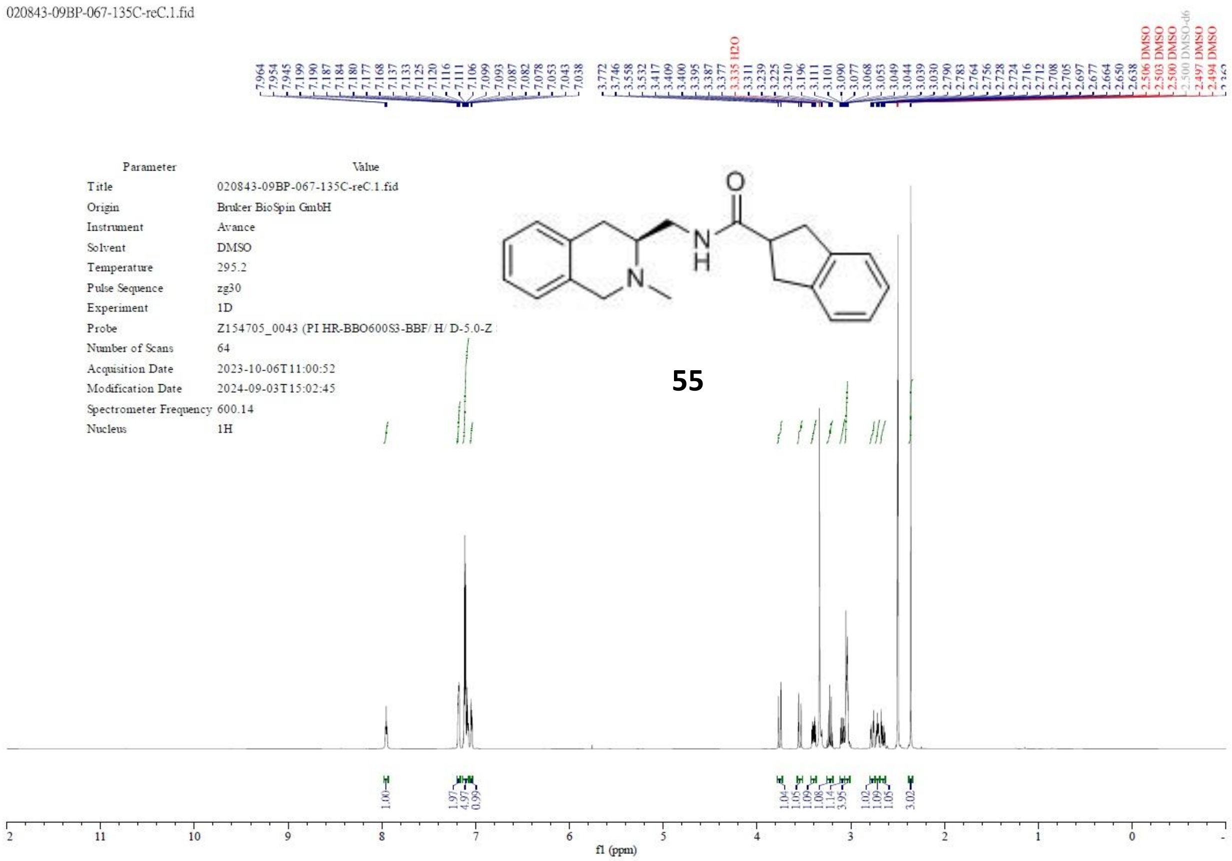

^{1}H NMR spectrum of compound **55**

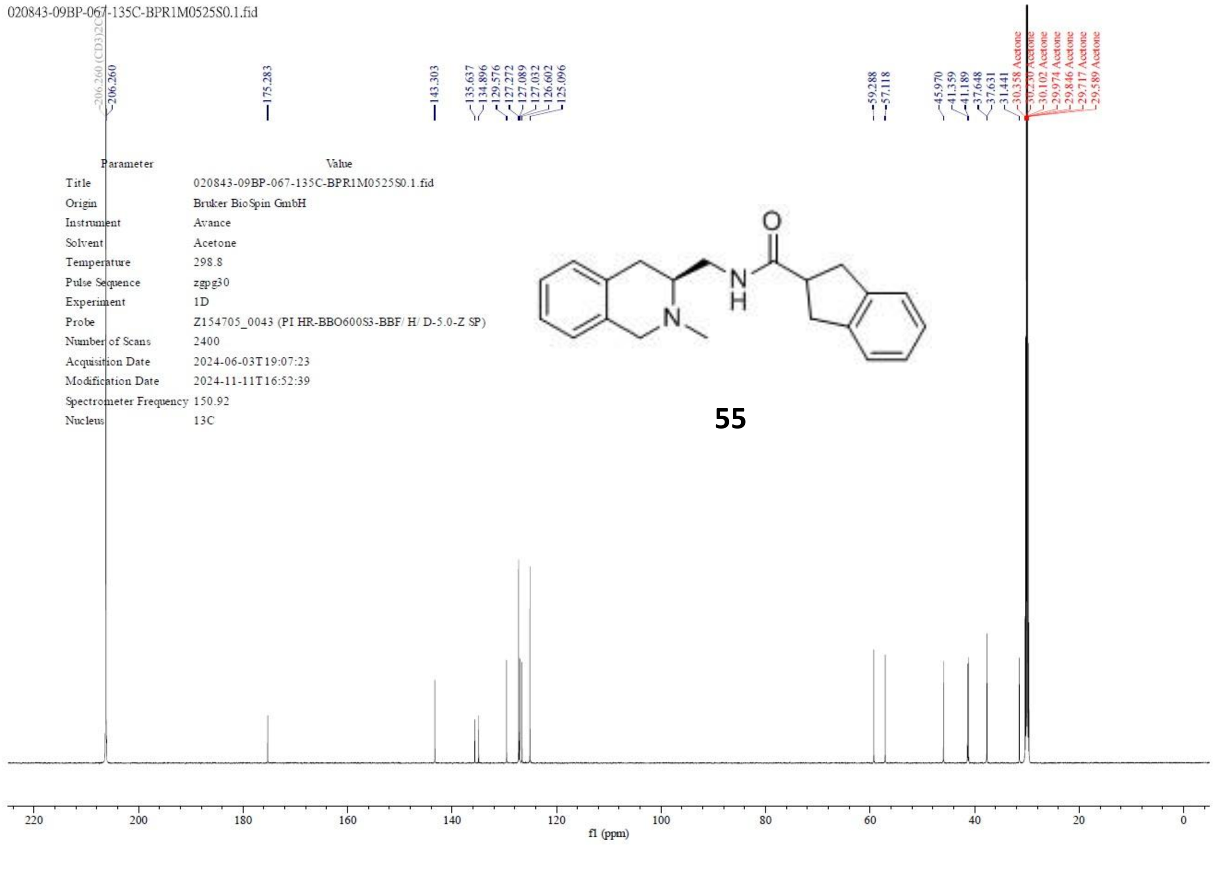

^{13}C NMR spectrum of compound **55**

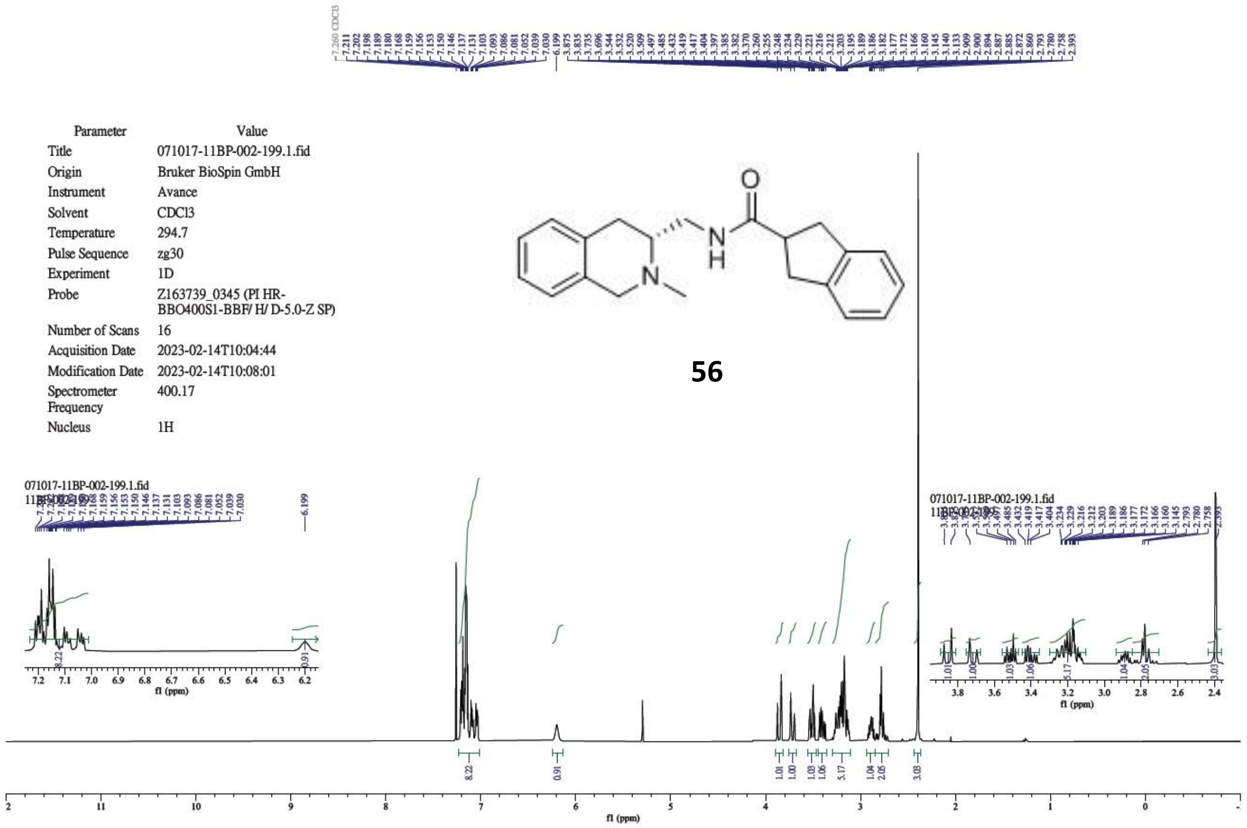

^{1}H NMR spectrum of compound **56**

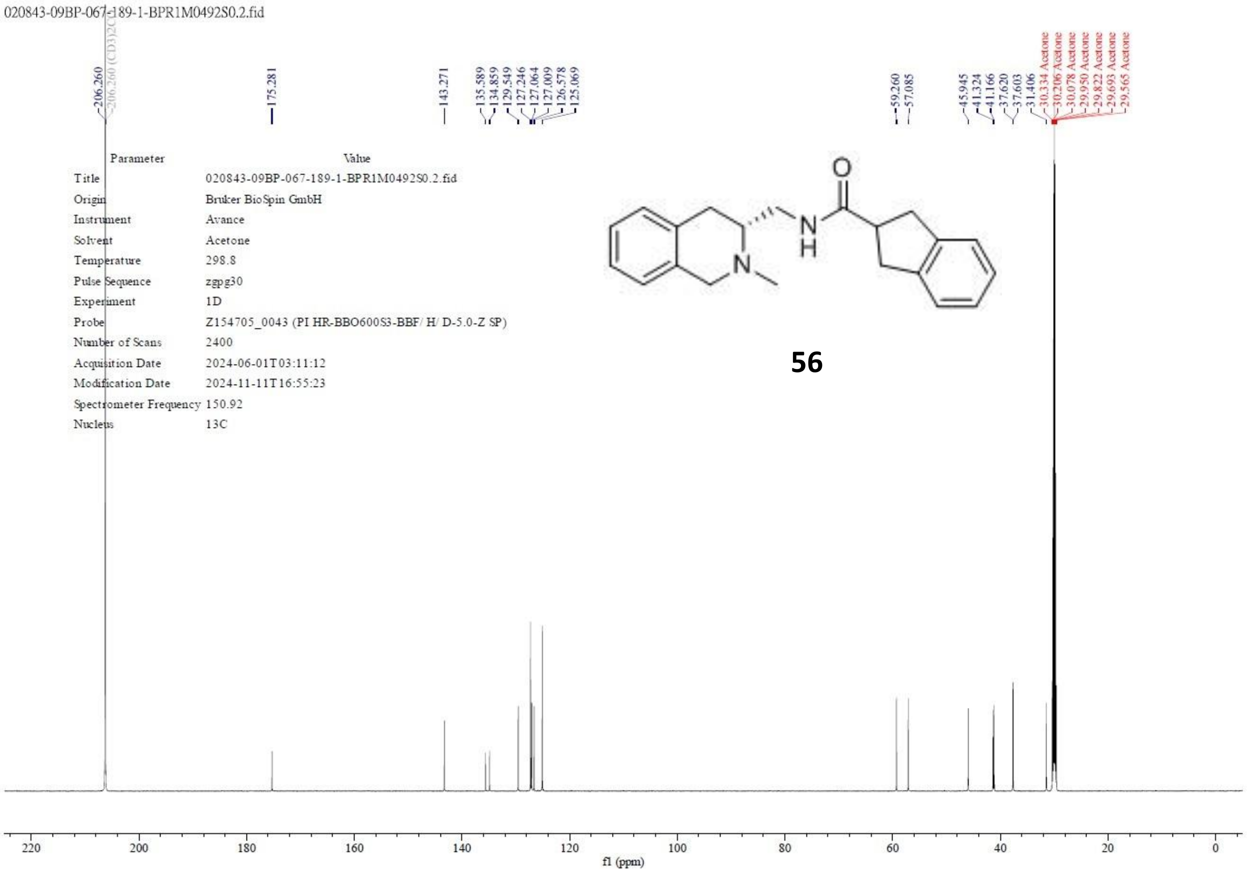

^{13}C NMR spectrum of compound **56**

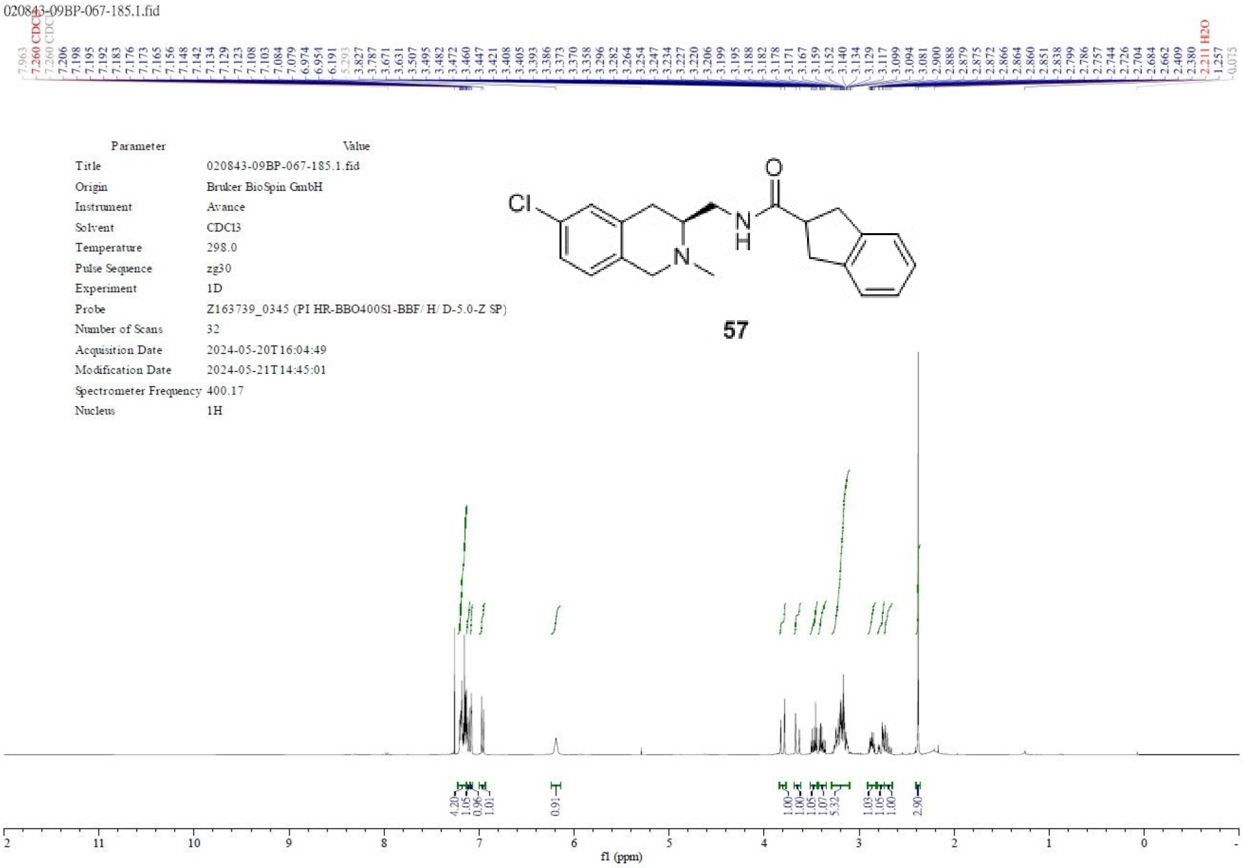

^{1}H NMR spectrum of compound **57**

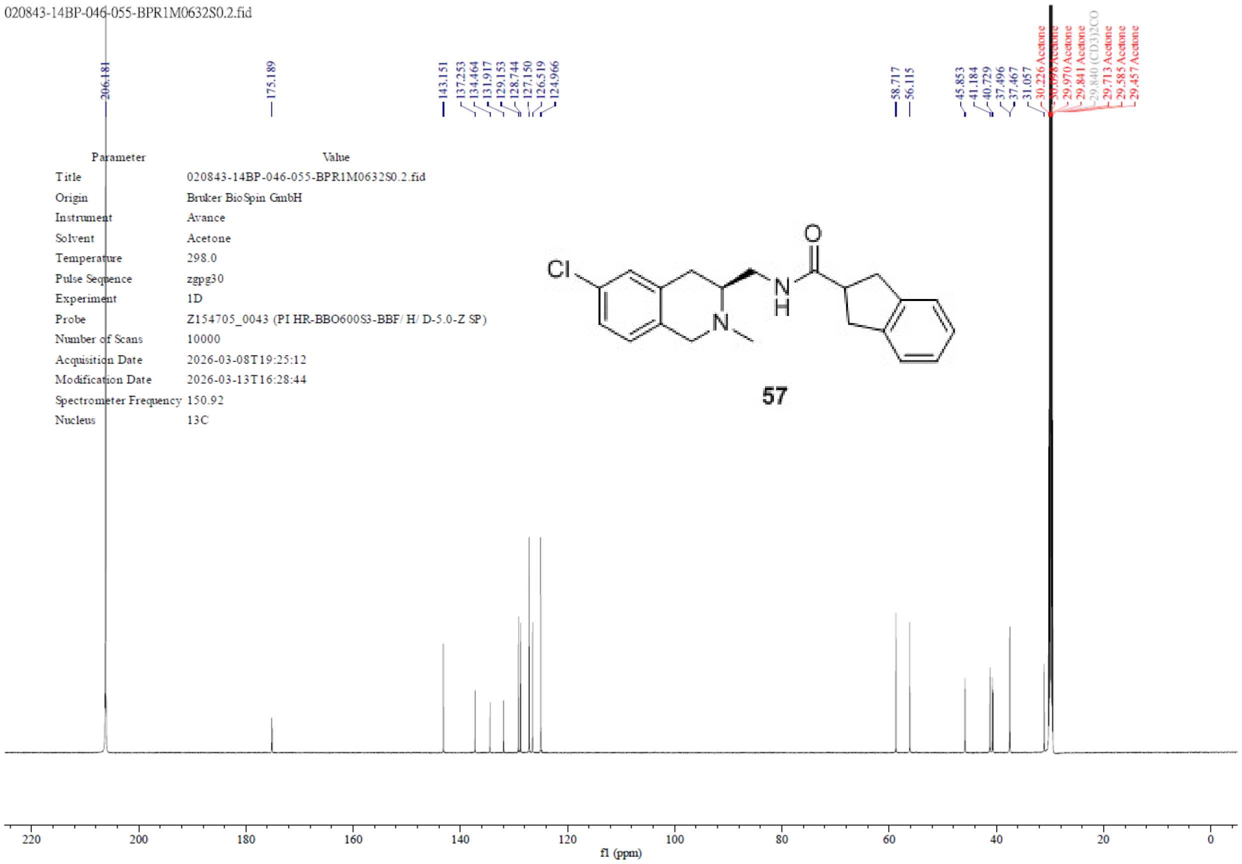

^{13}C NMR spectrum of compound **57**

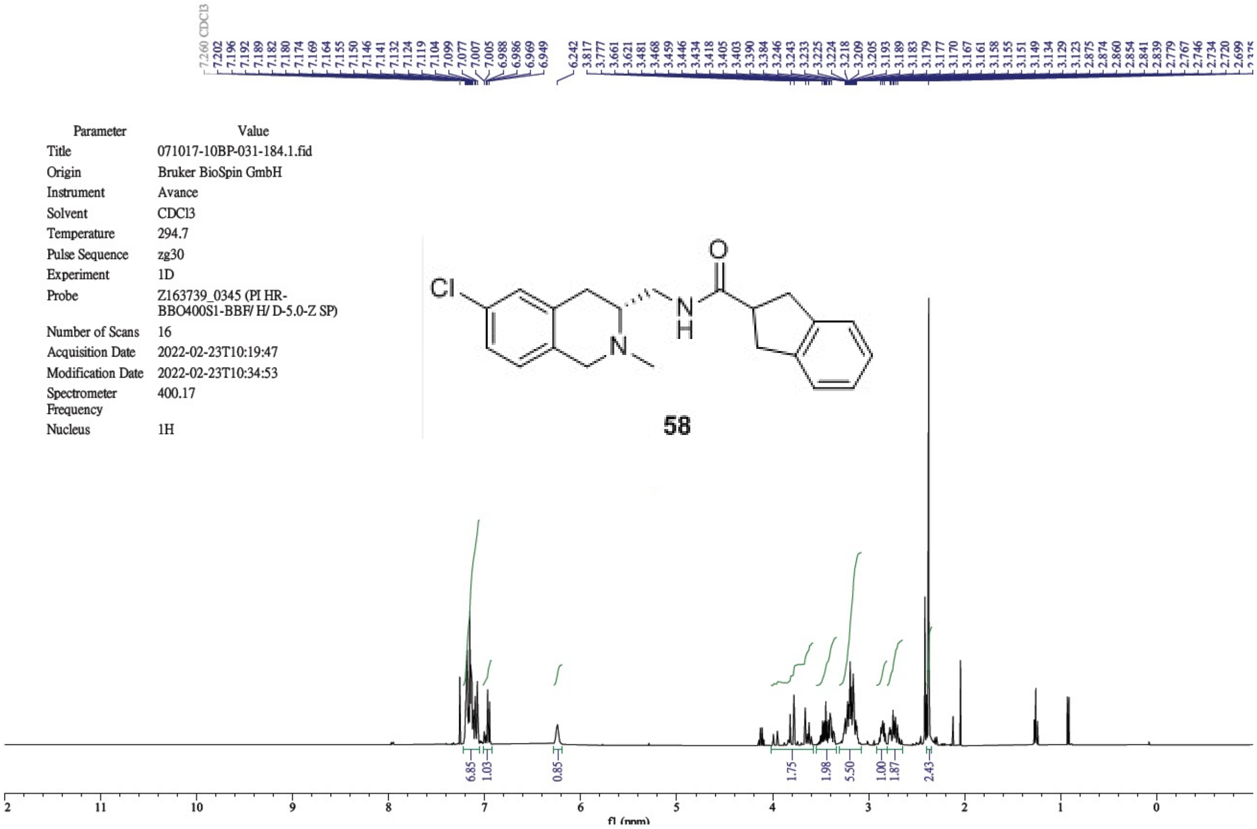

^{1}H NMR spectrum of compound **58**

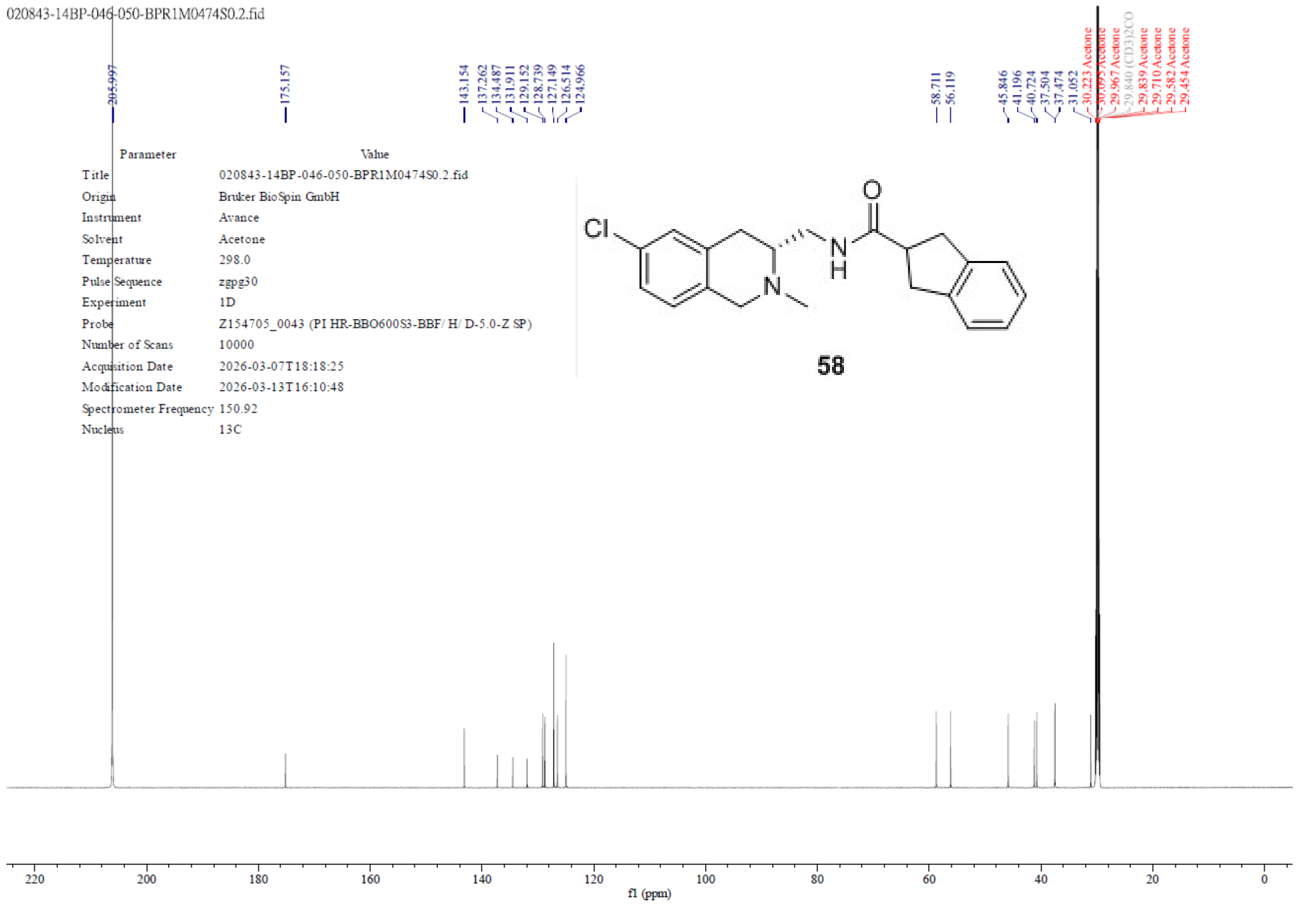

^{13}C NMR spectrum of compound **58**

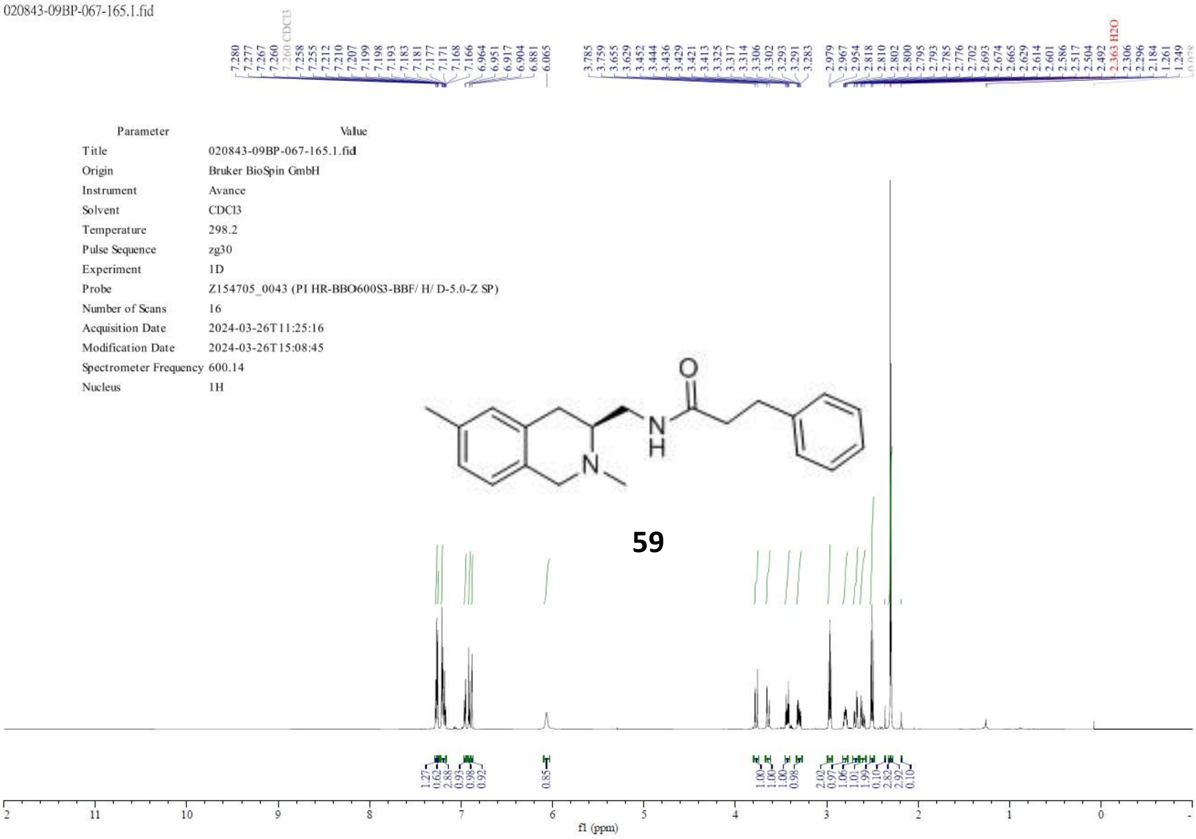

^{1}H NMR spectrum of compound **59**

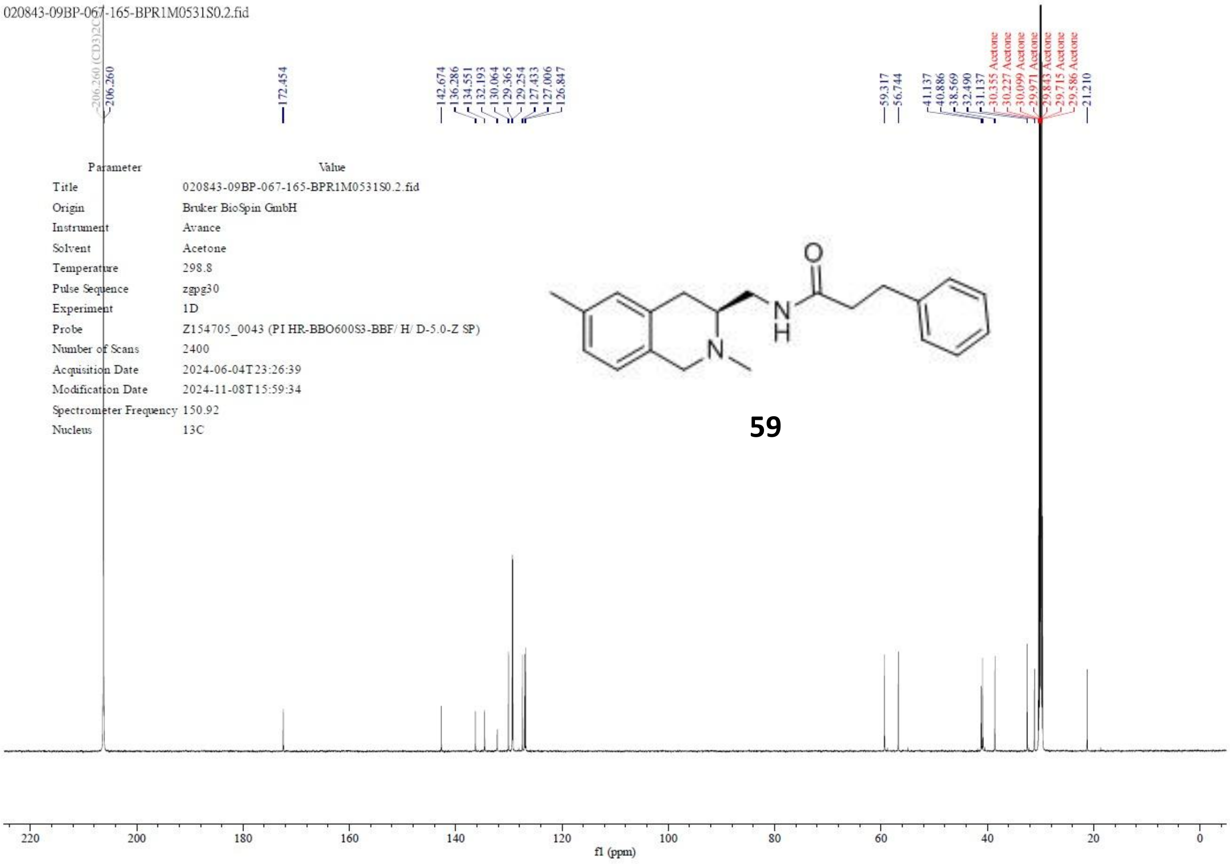

^{13}C NMR spectrum of compound **59**

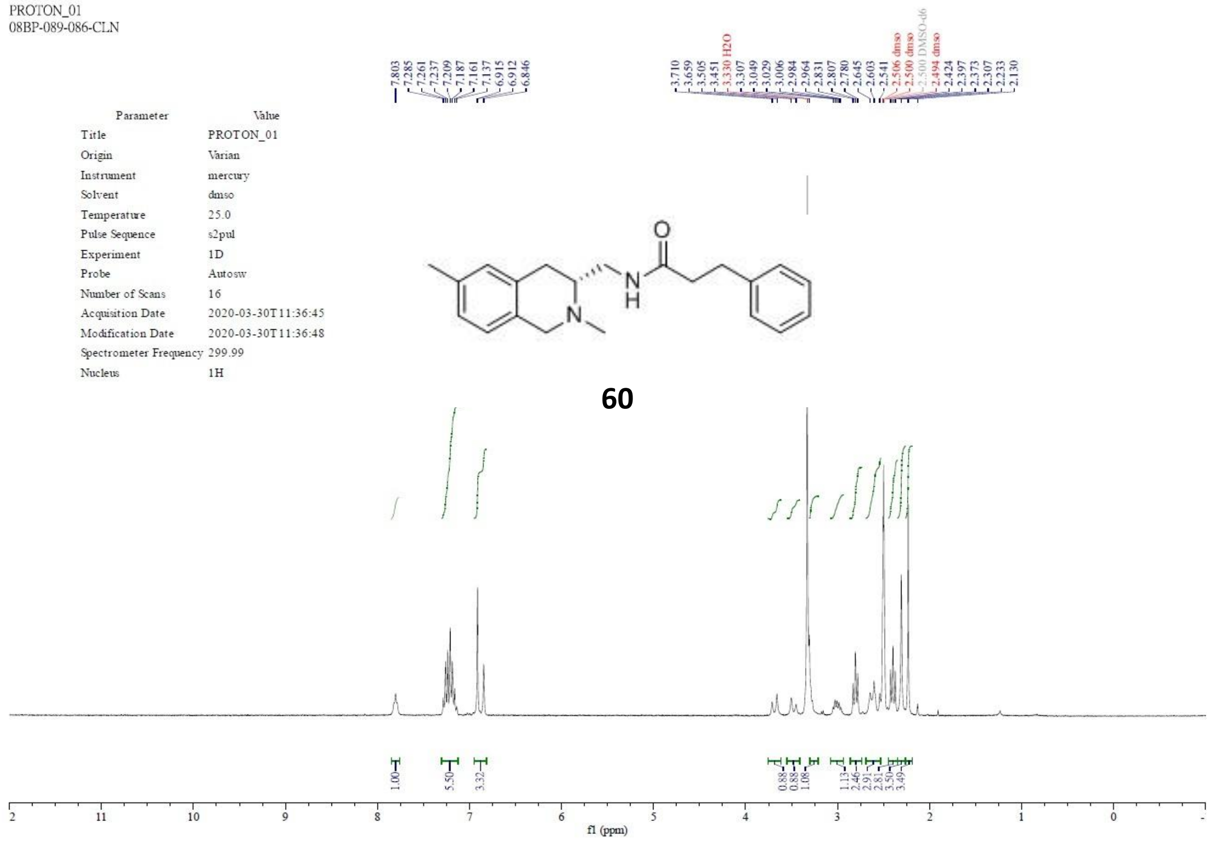

60

^1H NMR spectrum of compound **60**

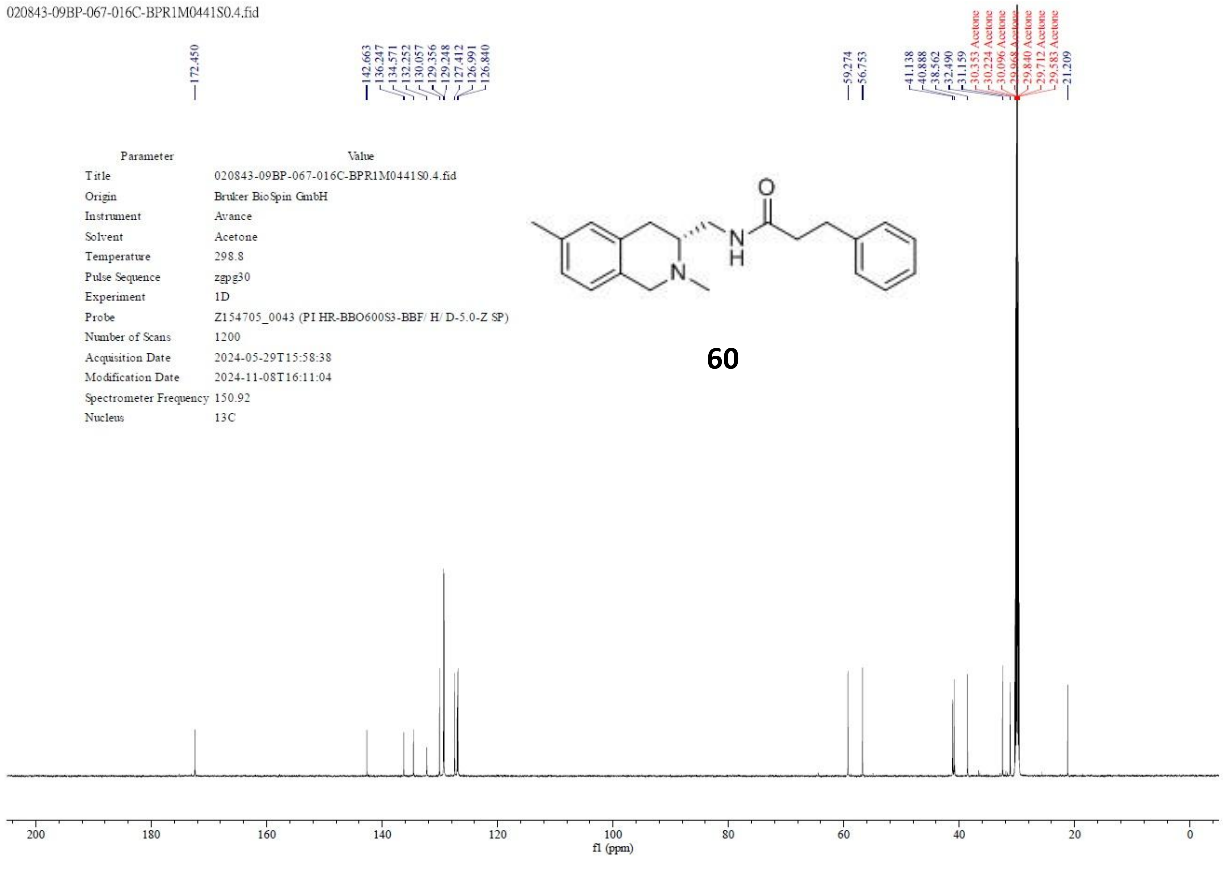

60

^{13}C NMR spectrum of compound **60**

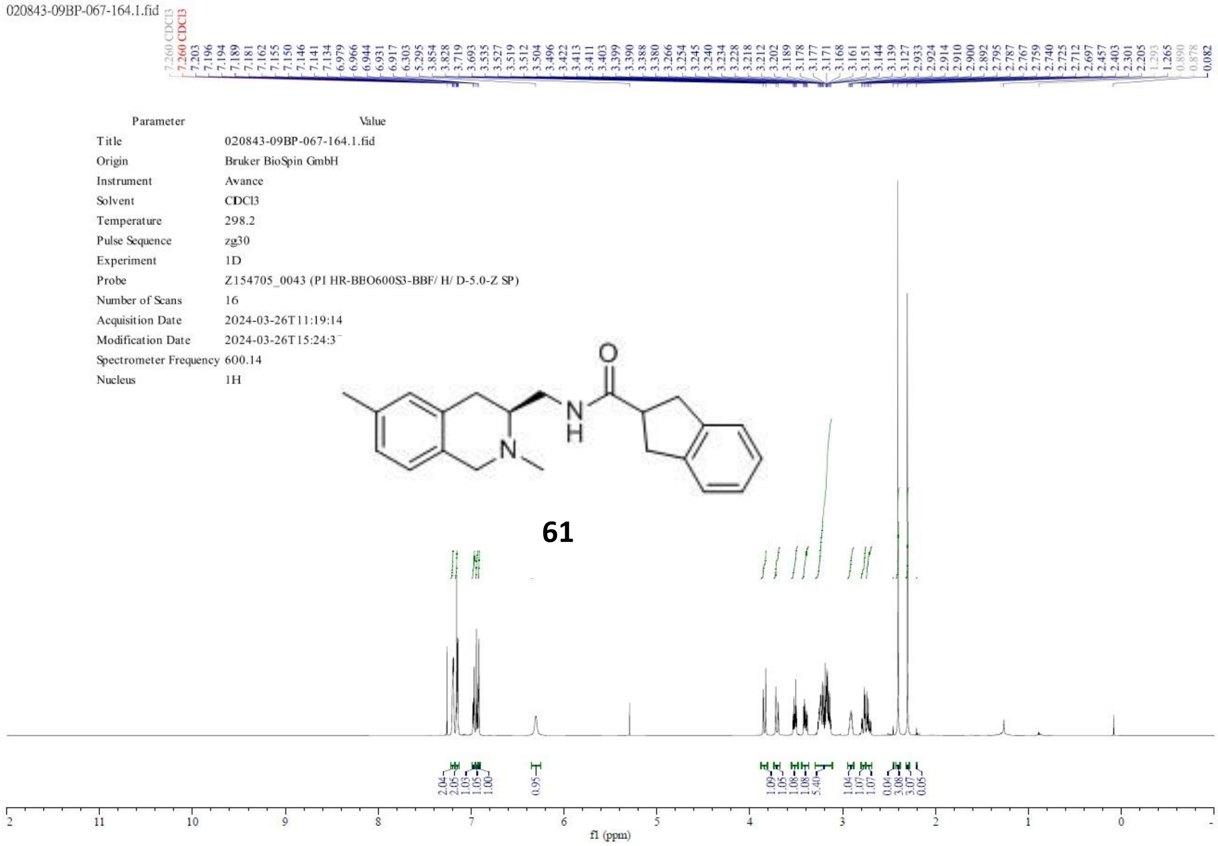

^{1}H NMR spectrum of compound **61**

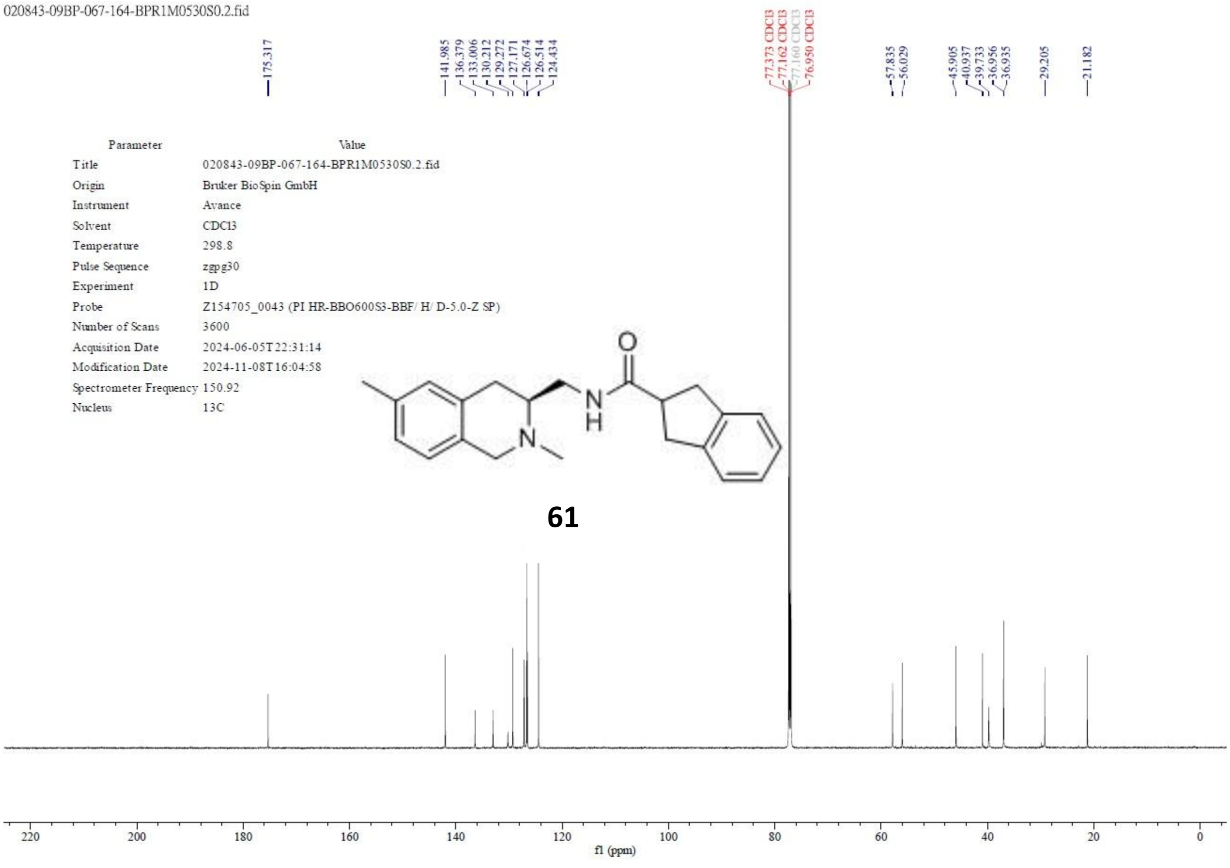

^{13}C NMR spectrum of compound **61**

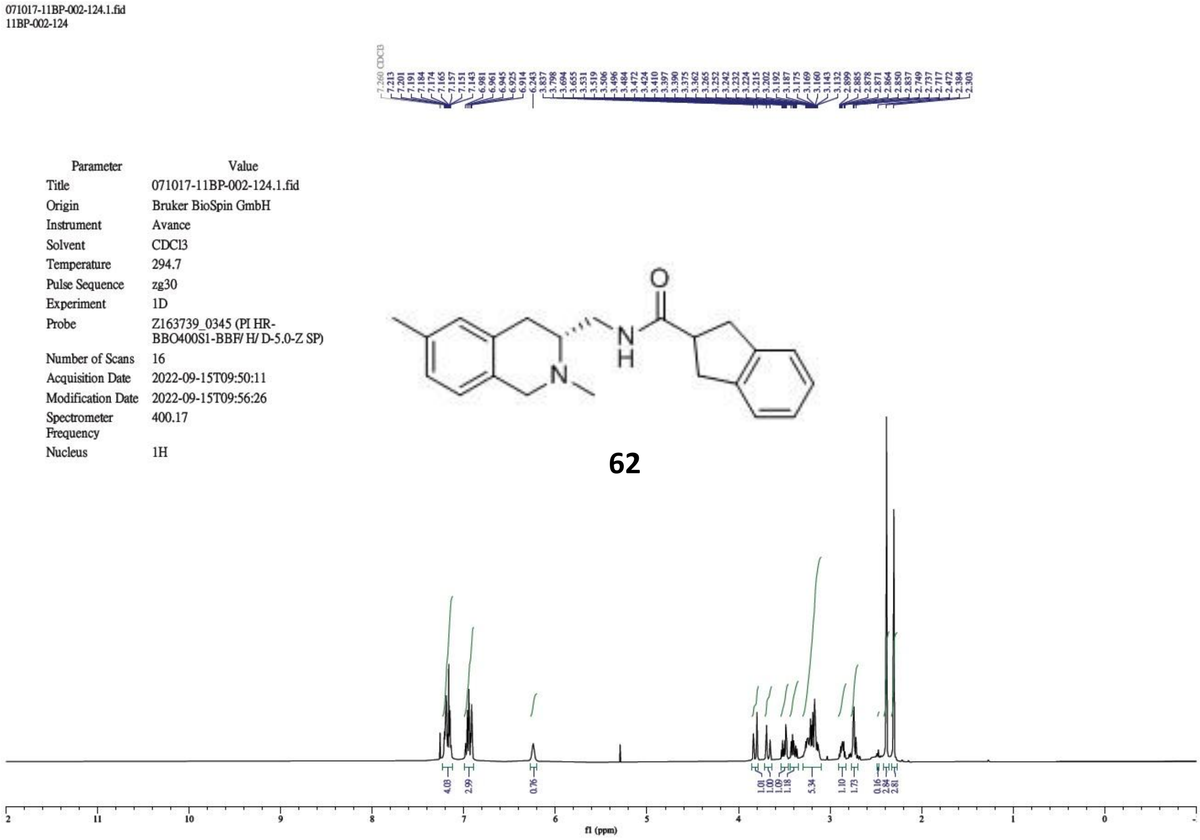

^{1}H NMR spectrum of compound **62**

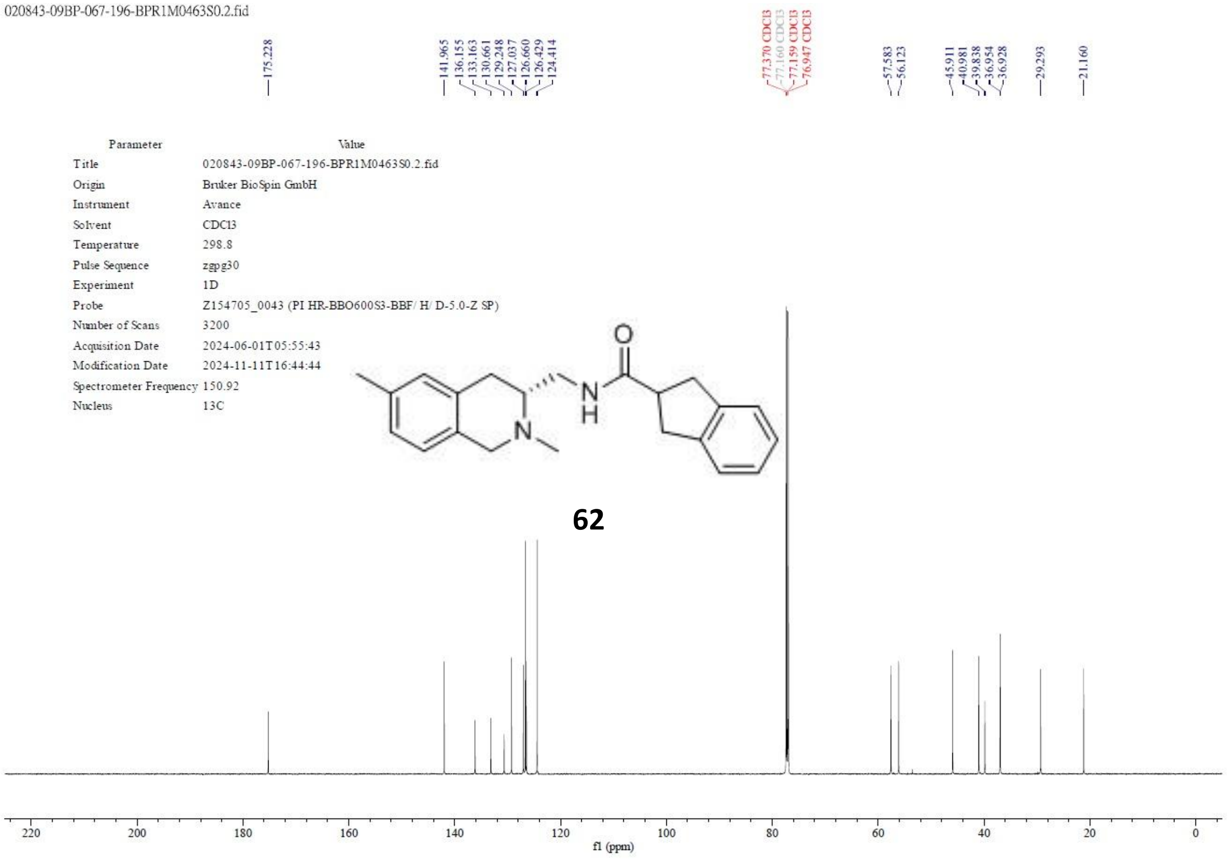

^{13}C NMR spectrum of compound **62**

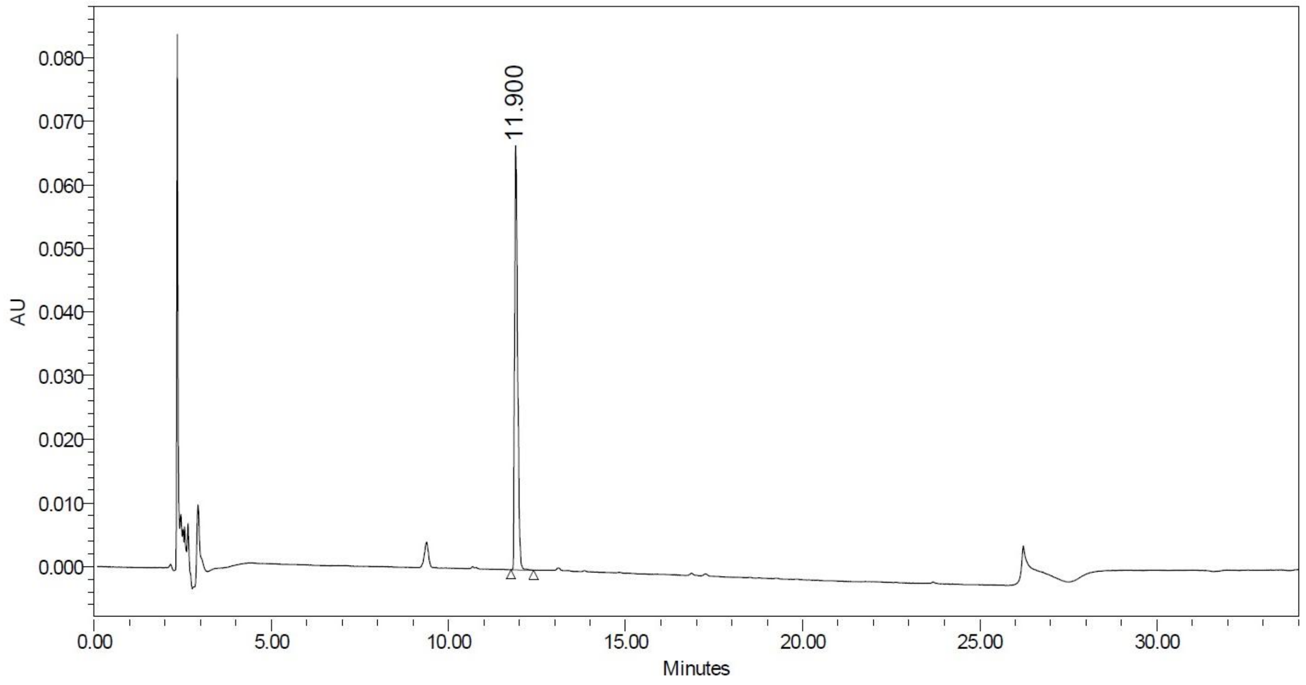

	RT	Area	% Area	Height
1	11.900	392850	100.00	66732

HPLC chromatogram of compound **56**

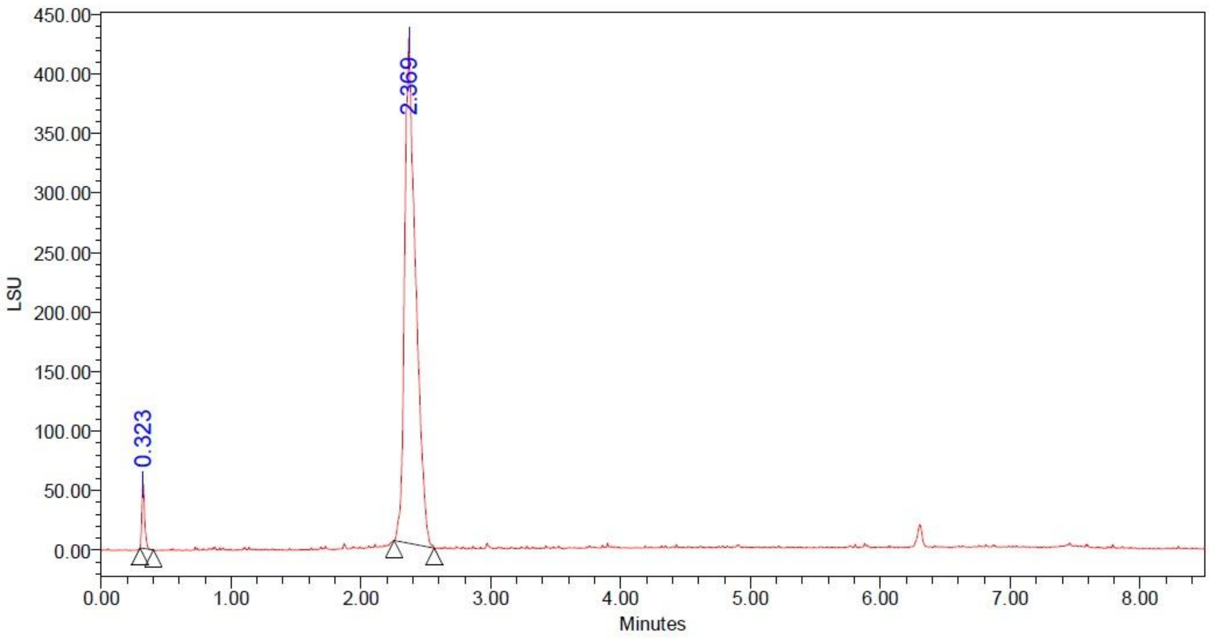

Peak Results

	Name	RT	Height	Area	% Area
1		0.323	54520	83206	3.22
2		2.369	424051	2501381	96.78

UPLC chromatogram of compound **60**

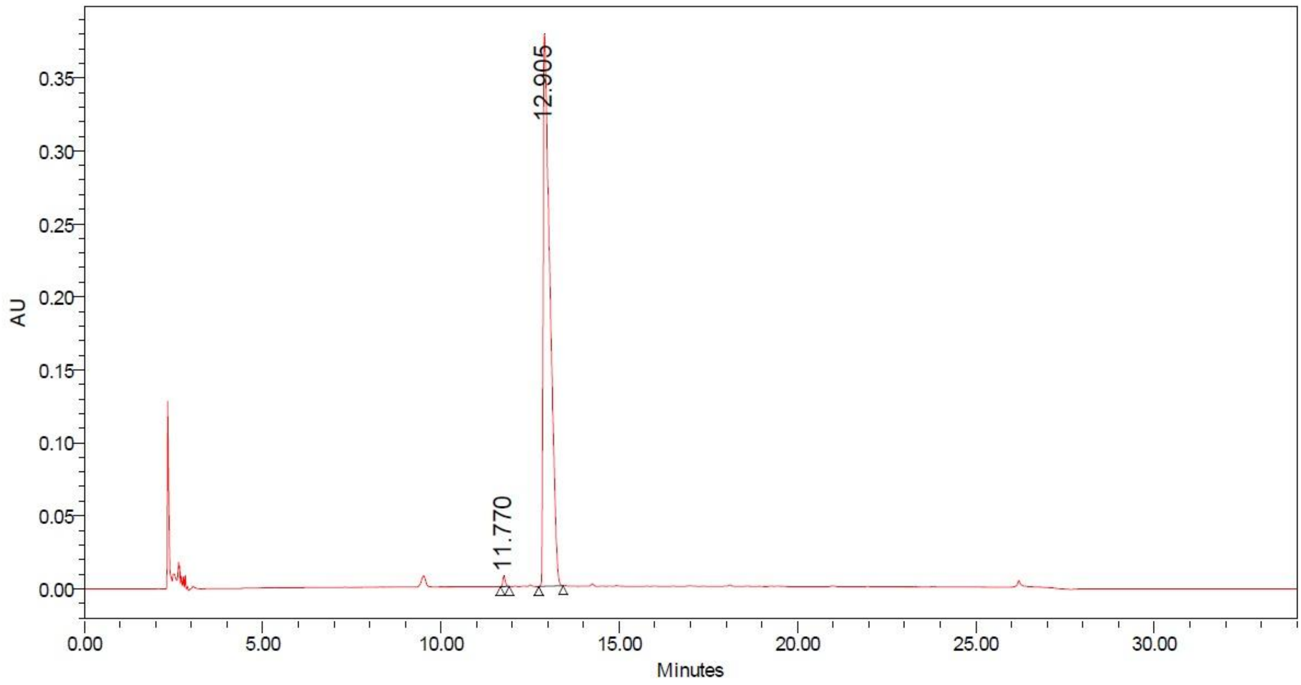

	RT	Area	% Area	Height
1	11.770	33396	0.67	7070
2	12.905	4976570	99.33	378447

HPLC chromatogram of compound **62**

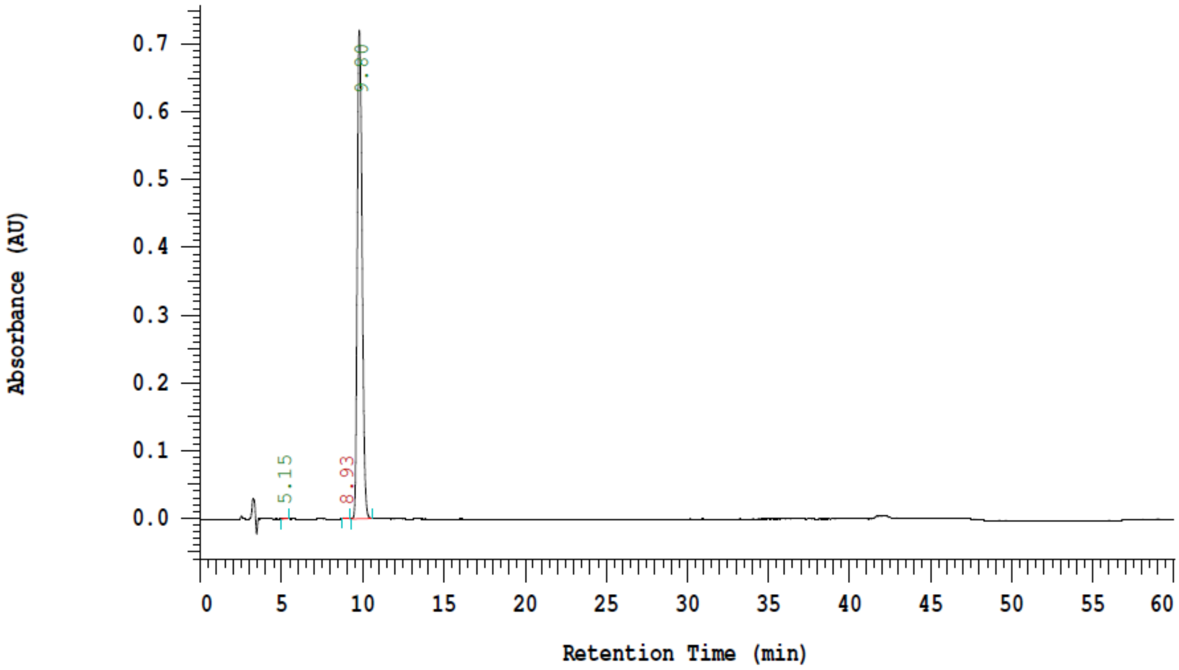

No.	RT	Area	Height	Conc 1
1	5.15	4219	454	0.059
2	8.93	12029	778	0.169
3	9.80	7090300	360393	99.771
		7106548	361625	100.000

Chiral HPLC chromatogram of compound **55**

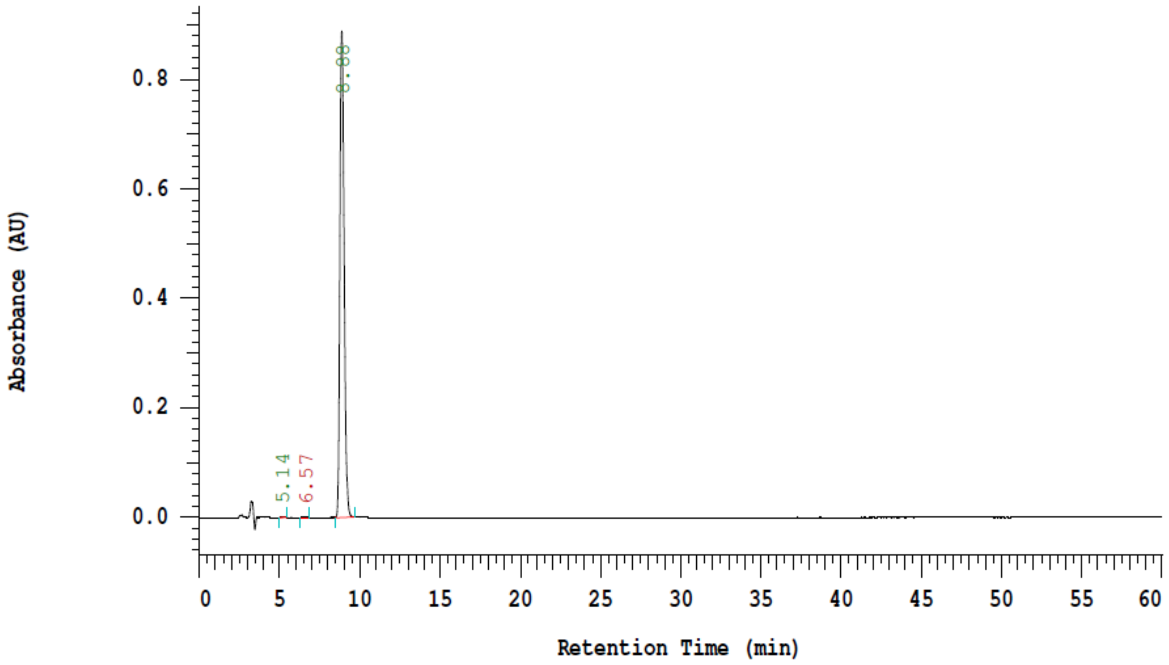

No.	RT	Area	Height	Conc 1
1	5.14	4855	473	0.060
2	6.57	8406	624	0.105
3	8.88	8025560	443842	99.835
		8038821	444939	100.000

Chiral HPLC chromatogram of compound **56**

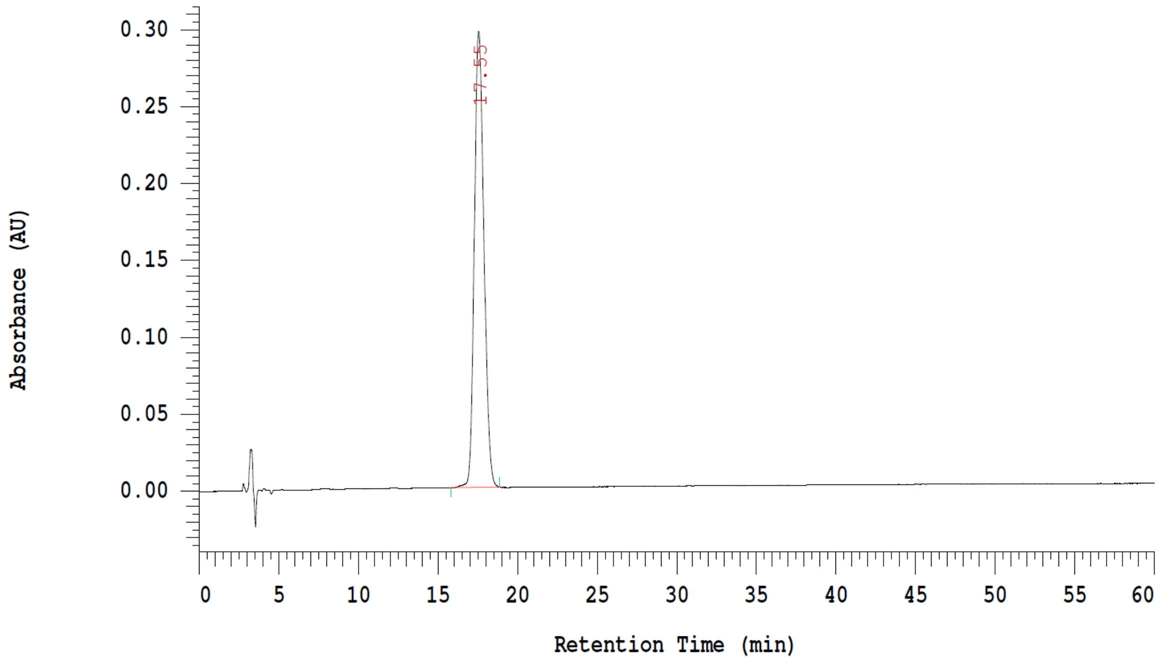

No.	RT	Area	Height	Conc 1
1	17.55	6095582	148043	100.000
		6095582	148043	100.000

Chiral HPLC chromatogram of compound **57**

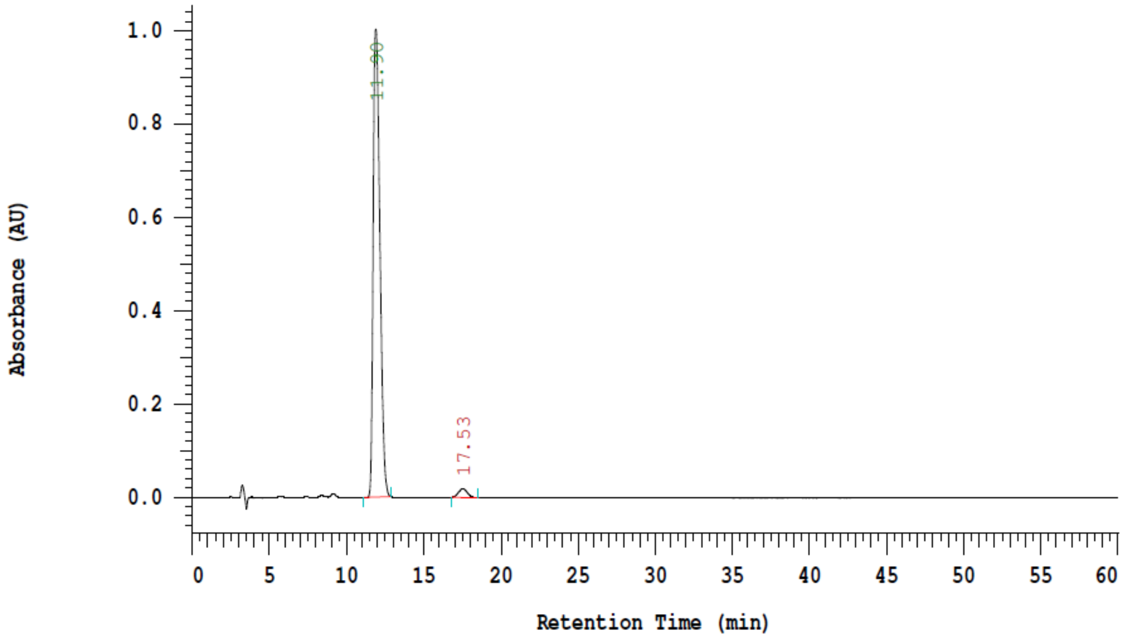

No.	RT	Area	Height	Conc 1
1	11.90	14632201	500973	97.521
2	17.53	371931	9373	2.479
		15004132	510346	100.000

Chiral HPLC chromatogram of compound **58**

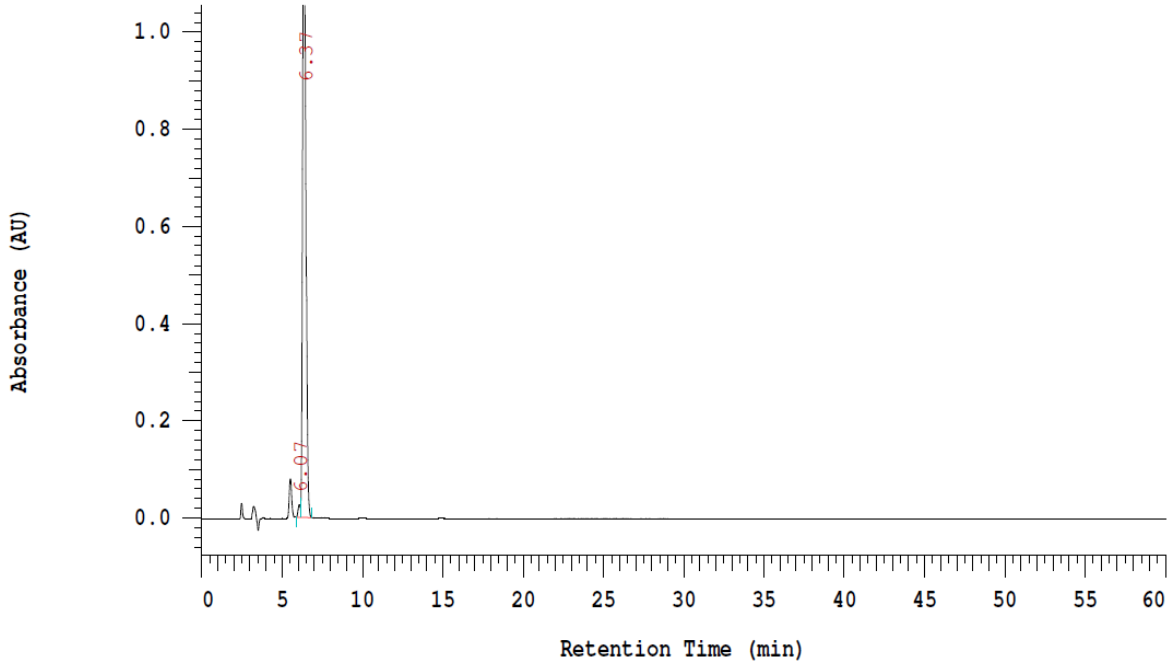

No.	RT	Area	Height	Conc 1
1	6.07	112811	12988	1.280
2	6.37	8699504	646774	98.720

Chiral HPLC chromatogram of compound **59**

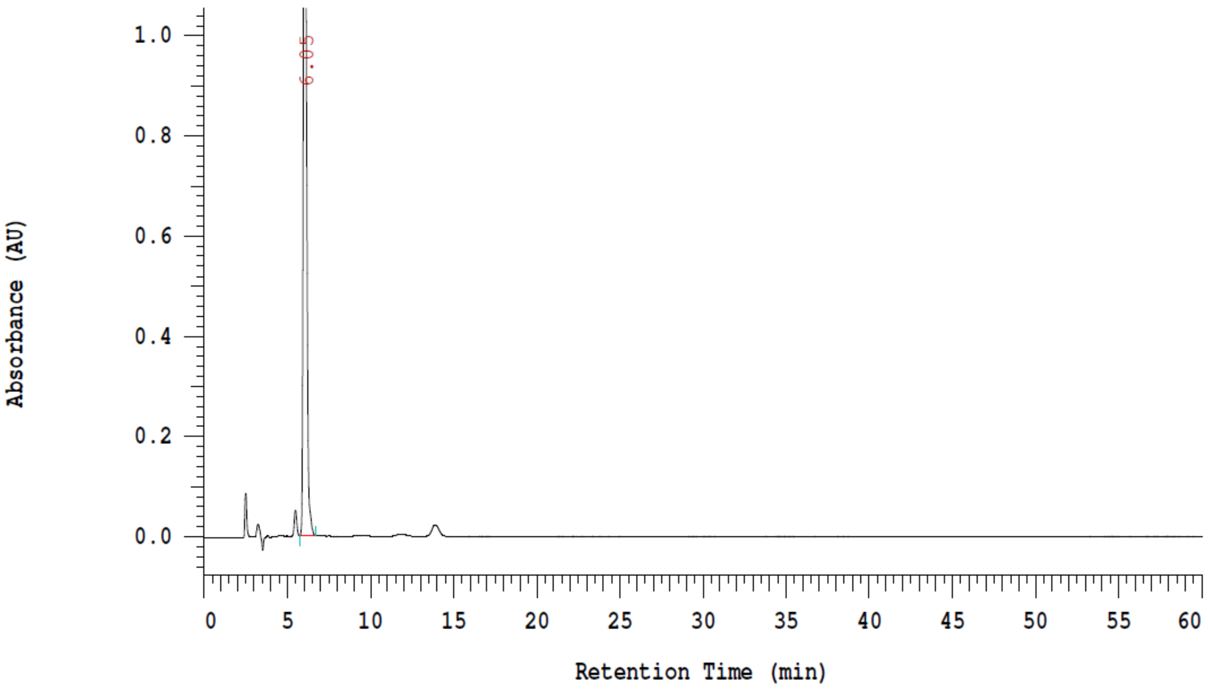

No.	RT	Area	Height	Conc 1
1	6.05	10191828	735706	100.000
		10191828	735706	100.000

Chiral HPLC chromatogram of compound **60**

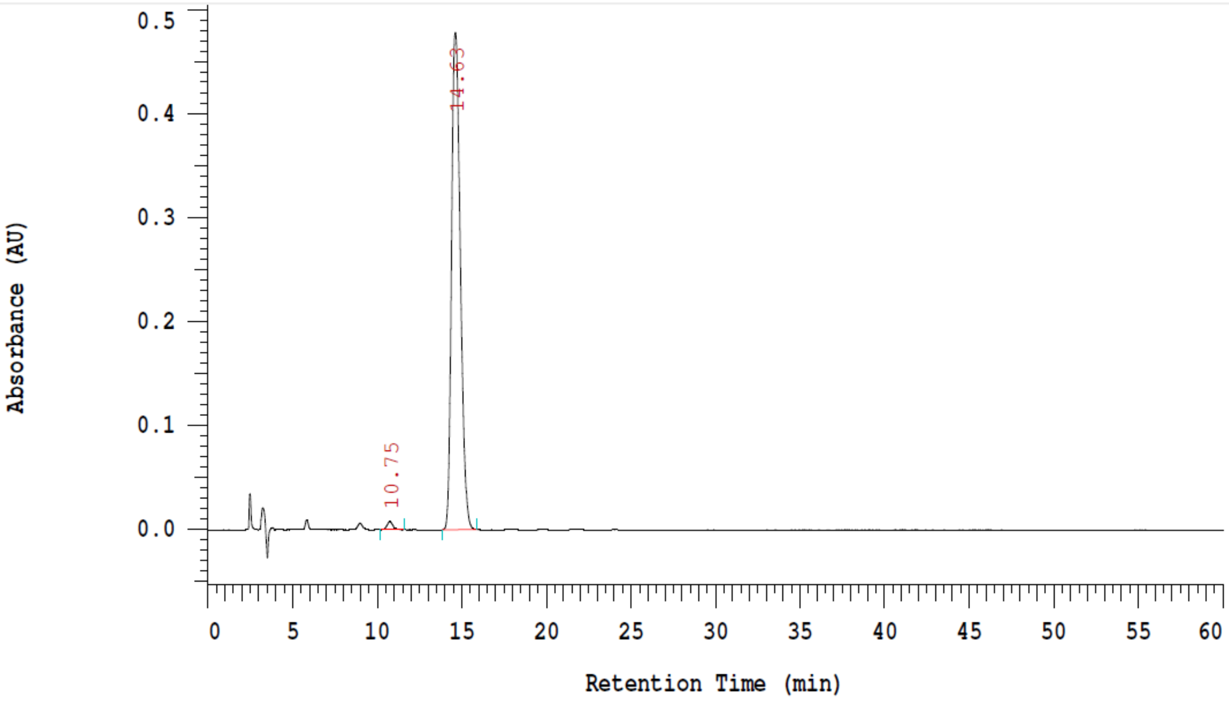

No.	RT	Area	Height	Conc 1
1	10.75	86340	3825	1.017
2	14.63	8402756	239104	98.983
		8489096	242929	100.000

Chiral HPLC chromatogram of compound **61**

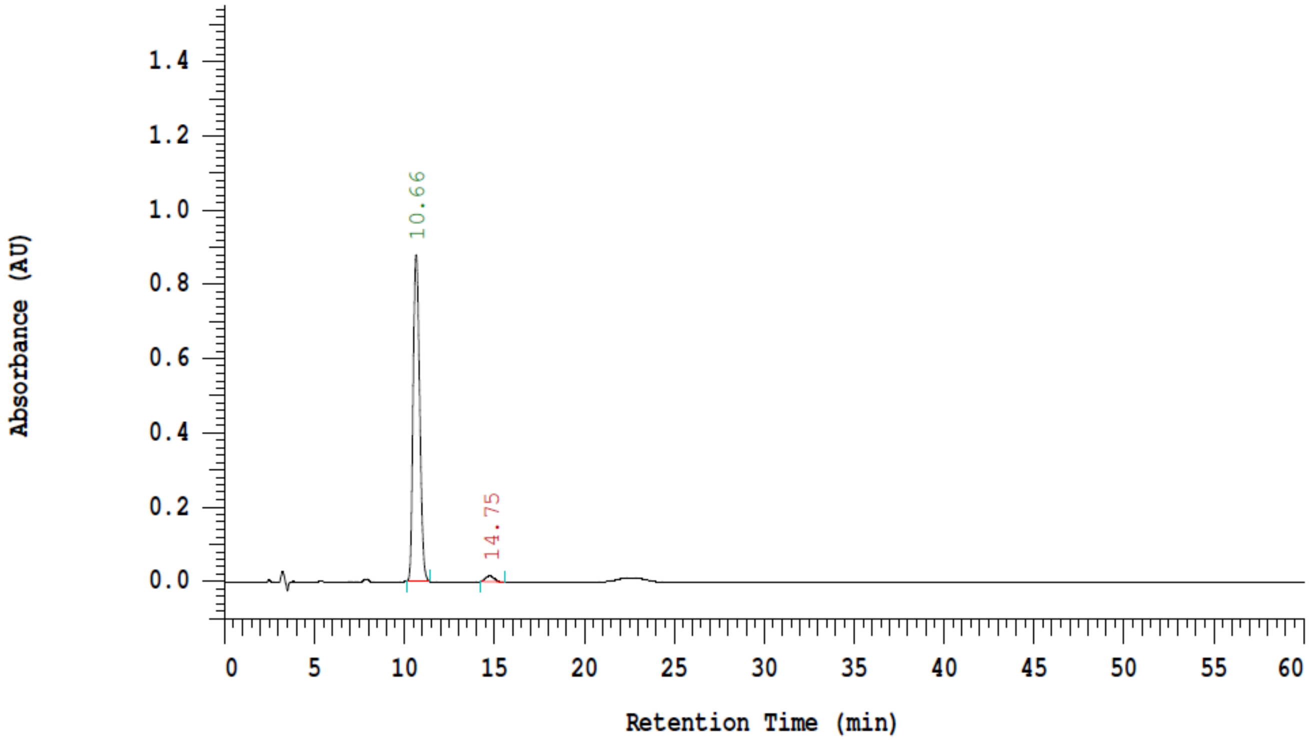

No.	RT	Area	Height	Conc 1
1	10.66	10695545	439309	97.730
2	14.75	248469	7824	2.270
		10944014	447133	100.000

Chiral HPLC chromatogram of compound **62**
